# Photoredox catalysed reductive cleavage of dibenzothiophene dioxides enabled by a temperature-controlled photoreactor

**DOI:** 10.1039/d5sc05889a

**Published:** 2025-10-23

**Authors:** Siyuan Wang, Quang Truong Le, Yoshiteru Shishido, Ismail Y. Kokculer, Ken Yamazaki, Gregory J. P. Perry, Adrian M. Nightingale, Hideki Yorimitsu

**Affiliations:** a School of Chemistry and Chemical Engineering, University of Southampton Southampton SO17 1BJ UK gregory.perry@soton.ac.uk; b Mechanical Engineering, Faculty of Engineering and Physical Sciences, University of Southampton Southampton SO17 1BJ UK A.Nightingale@soton.ac.uk; c Department of Chemistry, Graduate School of Science, Kyoto University Sakyo-ku Kyoto 606-8502 Japan yori@kuchem.kyoto-u.ac.jp; d Division of Applied Chemistry, Okayama University Tsushimanaka Okayama 700-8530 Japan

## Abstract

The cleavage of C–S bonds in dibenzothiophene dioxides under reductive photoredox catalysed conditions is reported. The reactions afford sulfinates, which can be used in a variety of subsequent transformations for diversification. When using unsymmetrical dibenzothiophene substrates, reductive cleavage occurs preferentially at one C–S bond. Experimental and computational studies provide insight into this interesting selectivity. The process tolerates the presence of oil (dodecane), highlighting a possible application in the removal of dibenzothiophene impurities from crude oil. The reactivity of some substrates is highly dependent on reaction temperature, hence the development of a versatile and inexpensive 3D printed photoreactor that allows for the precise control of reaction temperature is also reported.

## Abstract 2

Photoredox catalysed reductive cleavage of C–S bonds in dibenzothiophene dioxides enabled by a 3D-printed photoreactor with temperature control.

## Graphical abstract

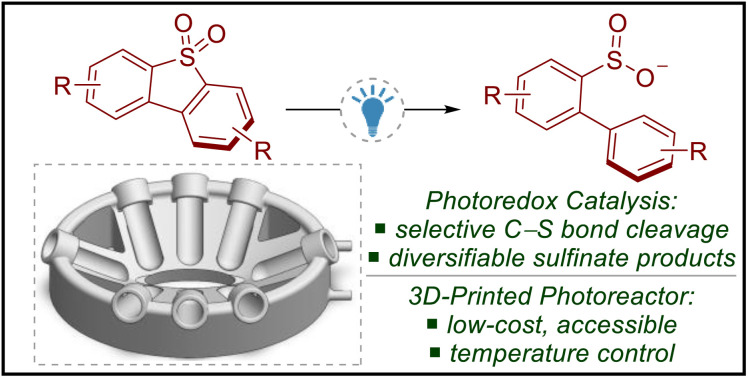

Sulfinate salts (RSO_2_^−^) are highly versatile compounds in synthesis.^1^ They are routinely used to access a range of important functional groups such as sulfonamides, sulfones and sulfonyl fluorides, with applications in various fields, for example biology and medicine.^2^ They can also be used in a variety of valuable cross-coupling reactions by extrusion of SO_2_ to access important C–C and C–heteroatom bond linkages.^1^

Desulfonylation *via* reductive C–S bond cleavage of sulfones is highly useful in organic synthesis but generally requires strongly reducing metals.^3^ Milder photocatalytic methods for C–S bond cleavage are known, but these routes often require activated substrates, for example sulfones containing heteroaromatic (azoles, pyridines *etc.*), alkenyl/alkynyl, polyfluoroalkyl, cyano or keto groups (Scheme 1A).^4^

**Scheme 1:**
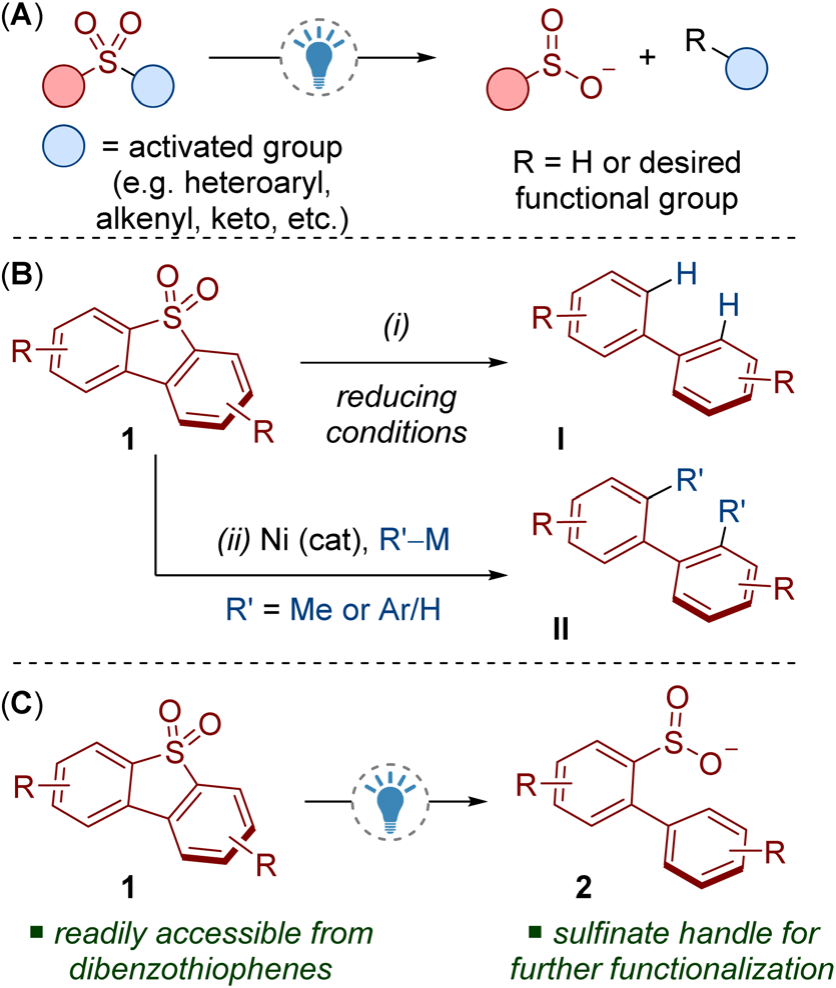
(A) Photocatalytic cleavage of activated sulfones. (B) Reductive cleavage of dibenzothiophene dioxides. (C) This work: photoredox catalysed reductive cleavage of dibenzothiophene dioxides.

Dibenzothiophene dioxides 1 are a class of relatively unactivated sulfones that can be easily accessed through routine oxidation of dibenzothiophenes. Recent investigations into the deconstruction of dibenzothiophene dioxides has resulted in a variety of useful C–S bond cleaving transformations that have found applications in synthesis and neighbouring fields.^5,6^ Investigating the reactivity of dibenzothiophene dioxides may also deliver benefits in oxidative desulfurization for removing dibenzothiophene impurities from petroleum.^7^

Methods for the reductive cleavage of dibenzothiophene dioxides 1 are limited, especially from a synthetic perspective. For example, many methods lead to unfunctionalized biaryls I and require relatively forcing conditions (Scheme 1B, i).^8^ More attractive cross-coupling methods have been demonstrated, but they suffer from low yields, product mixtures and/or poor generality (Scheme 1B, ii).^9^ Both strategies also lead to the complete removal of the sulfur functionality. A complementary cleavage process that retains the sulfur moiety would deliver products with a synthetic handle for accessing various modes of reactivity and valuable functional groups.

Here we report a photocatalytic reductive C–S bond cleavage of dibenzothiophene dioxides 1 (Scheme 1C). The reaction produces sulfinates 2 which can be used as a platform for diversification, for example *via* fluorination, alkylation and cross-coupling reactions. Computational and experimental results provide insight into this unique mode of reactivity. To aid these studies, we also introduce a 3D printed photoreactor that will prove useful to others in need of precise temperature control for photochemical reactions.

Our previous work on sulfonamide functionalization provided a photoredox catalysed reduction of sulfonyl pyrroles.^10^ With these conditions, our initial results were encouraging, showing that sulfinate 2a (analysed as the corresponding sulfonyl fluoride 3a) was formed in 38% yield from dibenzothiophene dioxide 1a (Table 1, Entry 1). Interestingly, the sulfur functionality was retained under our conditions, providing a complementary approach to previous reductions in which the sulfone 1 is either reduced to the dibenzothiophene^11^ or the SO_2_ unit is lost completely (*c.f.*Scheme 1B),^8,9^ and opens the possibility for further derivatization (*vide infra*, Scheme 5A). Further optimisation revealed that the removal of water and addition of formic acid had a beneficial effect on the reaction yield (Entry 2). Optimized conditions were achieved by switching the photoredox catalyst from Ir1 to the more strongly reducing Ir2 (Entry 3, Ir1: *E*_red_ [Ir^III^/Ir^II^] = −1.51 V *vs.* SCE in MeCN; Ir2: *E*_red_ [Ir^III^/Ir^II^] = −2.19 V *vs.* SCE in MeCN).^12^ Finally, the reaction did not proceed in the absence of the light source or the photoredox catalyst.^13^

**Table 1:** Optimisation of the photoredox catalysed reductive cleavage of dibenzothiophene dioxides^a^

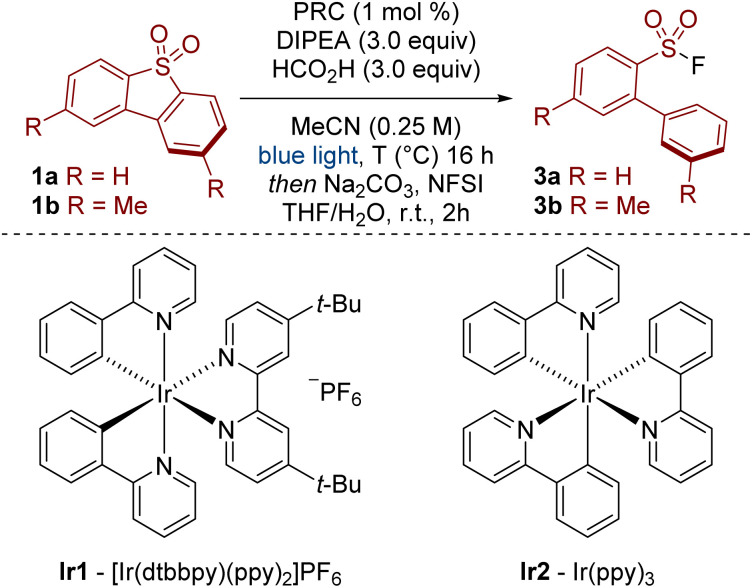				
Entry	R	PRC	*T* (°C)	3a/3b^b^ (%)
1^c^	H	Ir1	25	38
2^d^	H	Ir1	25	44
3	H	Ir2	25	95 (93)^e^
4	Me	Ir2	25	18
5	Me	PTH^f^	25	36
6^g^	Me	Ir2	25	32
7	Me	Ir2	50	85 (82)^e^

a Reaction conditions: 1 (0.5 mmol), photoredox catalyst (PRC, 1 mol%), DIPEA (3.0 equiv.), HCO_2_H (3.0 equiv.), MeCN (0.25 M), light = Kessil PR160L 440 nm, *T* = 25 °C or 50 °C, 16 h. Remove volatiles then Na_2_CO_3_ (1.5 equiv.), *N*-fluorobenzenesulfonimide (NFSI, 2.0 equiv.), THF/H_2_O (9 : 1, 0.25 M).

b ^1^H NMR yields using CHCl_2_CHCl_2_ as an internal standard.

c DIPEA = 4.0 equiv., HCO_2_H = 0 equiv., solvent = MeCN/H_2_O (6 : 1, 0.25 M).

d DIPEA = 4.0 equiv., HCO_2_H = 4.0 equiv.

e Yield in parenthesis is of isolated material.

f 10-Phenylphenothiazine (PTH, 5 mol%), light = Kessil PR160L 390 nm.

g MeCN (0.125 M).

With a mild and efficient process in hand, we began to explore the scope of the reaction but found some substrates gave significantly lower yields. For example, 2,8-dimethyldibenzothiophene dioxide 1b gave only 18% yield of product 3b under the standard reaction conditions (Entry 4). We considered two possibilities for this decrease in reactivity: susceptibility towards reduction and solubility. Firstly, substrate 1b may be less reactive as it is harder to reduce. This was reflected in the measured cathodic peak potentials (1a: *E*_pc_ = −1.7 V *vs.* SCE in MeCN, 1b: *E*_pc_ = −2.2 V *vs.* SCE in MeCN).^13^ A more strongly reducing photodeox catalyst, 10-phenylphenothiazine (PTH), showed an improvement in reactivity, but the yield was still unsatisfactory (Entry 5).^14^ Secondly, substrate 1b is relatively insoluble. For example, whereas ∼18 mg mL^−1^ of 1a was soluble in MeCN, only ∼2 mg mL^−1^ of 1b was soluble in MeCN.^13^ In this regard, diluting the reaction mixture to 0.125 M showed a small improvement in yield (Entry 6). More polar solvents (DMF and DMSO) did not improve reactivity.^13^ Ultimately, to better improve the solubility of the substrate and the overall reactivity of the system, we opted to increase the temperature to 50 °C, which, gratifyingly, provided the product in high yield (Entry 7).

Although increasing the temperature of a reaction is usually a simple task, this proves challenging under photochemical conditions due to a lack of suitable equipment. For example, when first conducting the photoredox reactions with 1b at 50 °C we had to part submerge the vials in a water bath, which was inconvenient, hazardous and impractical. Others have also been forced to use elaborate experimental setups when performing photochemical reactions at elevated or reduced temperatures.^15^ Previously reported photoreactors were found to be unsuitable as they either do not allow for accurate temperature control and/or were prohibitively expensive bespoke models.^16^ We therefore designed our own low-cost photoreactors that conveniently enable photochemical reactions at a range of temperatures (Fig. 1A).^17^ The importance of temperature control in photochemical reactions has recently been highlighted by Cañellas and co-workers.^16*j*^ Our photoreactors are 3D printed in polycarbonate using standard fused filament printers, making them low cost and easily accessible. The photoreactor can hold up to 8 vials (Fig. 1B), is designed to sit on a standard laboratory stirrer plate (Fig. 1D) and is oval shaped to match the illumination area for commonly used PR160L Kessil Lamps (ensuring each vial receives equal light intensity, Fig. 1E). Temperature-control is achieved by connection to a refrigerated/heating circulator. Liquid from the circulator is flowed through channels in the photoreactor body (Fig. 1C) located immediately behind each vial (Fig. 1C inset) allowing heat exchange with the vials without affecting the light path. Here we used water as the recirculating liquid to set the temperature at 25 or 50 °C, however we found temperatures between 10–80 °C were achievable. We envisage that the temperature range could be further extended using other recirculating liquids and reactor materials.

**Fig. 1:**
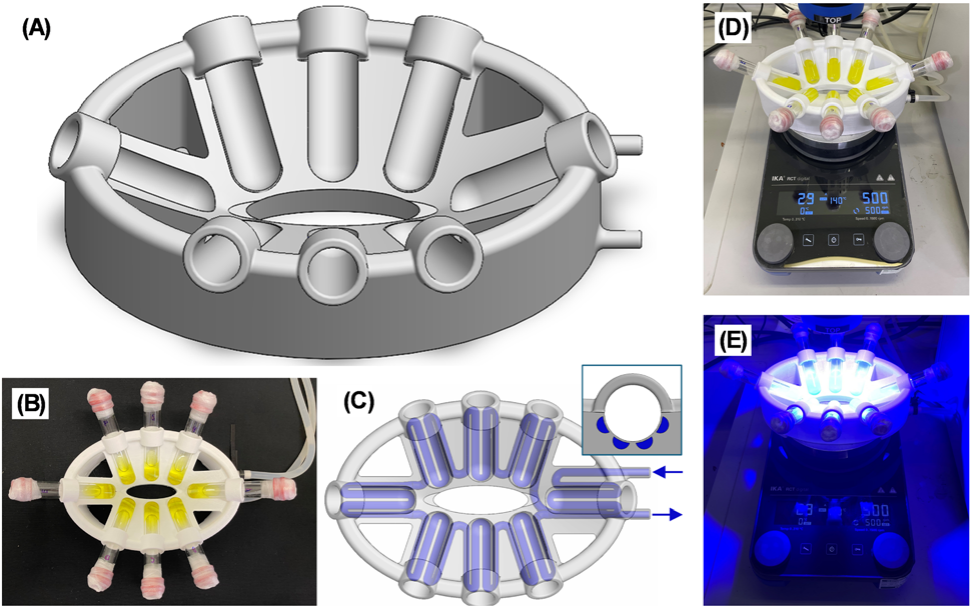
(A) CAD image of the 3D printed photoreactor. (B) 3D Printed photoreactor with reaction vials. (C) CAD image of the 3D printed photoreactor demonstrating the recirculating fluid. (C, inset) cross section showing the position of the recirculating fluid channels relative to a vial (D) reaction set up. (E) Reaction set up under irradiation.

With conditions and set-up optimised, we then tested the method on a range of substrates. Screening was made easier with our 3D printed photoreactor as we were able to simply switch between temperatures when required. For example, products 3a, 3d, 3e and 3h formed in appreciable yields at 25 °C, whereas 3b, 3c and 3g required heating. Substituents at various positions around the dibenzothiophene dioxide were tolerated (*e.g.* see 3b, 3c, 3d). Products bearing phenyl (3e, 3f) and methoxy substituents (3g, 3h) also formed in respectable yield. Some heteroaromatics were also tolerated, as demonstrated in the formation of the pyrazole-containing product 3i. Reactivity was also observed with trifluoromethoxy and fluoro substituents (3j and 3k). A substrate bearing an ester substituent gave none of the desired product 3l. The poor reactivity in this case may be due to the low solubility of the compound (<1 mg mL^−1^ in MeCN).^13^ Other unsuccessful substrates and details on the outcome of these reactions are provided in the SI (Table S4).

When testing unsymmetrical dibenzothiophene dioxides, we observed some impressive selectivity (Scheme 3). For example, substrate 1m was cleaved to give product 3m preferentially over isomer 3m′. Similarly, reductive cleavage of 1n provided 3n exclusively; the other isomer 3n′ was not observed. The structure of 3n was unequivocally determined by X-ray crystallography.^18^

**Scheme 2:**
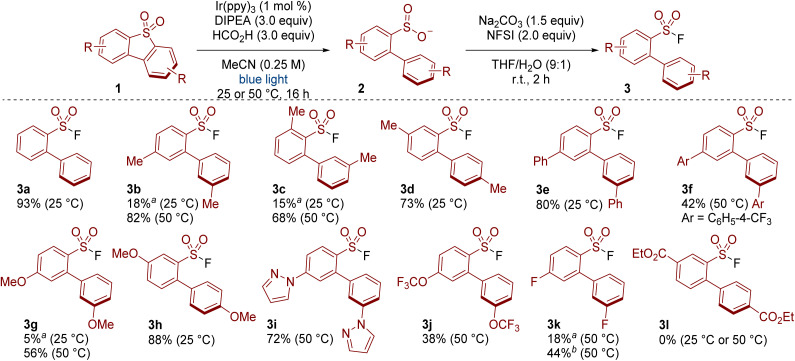
Scope of the photoredox catalysed cleavage of symmetrical dibenzothiophene dioxides. ^*a* 1^H NMR yields using CHCl_2_CHCl_2_ as an internal standard. ^*b*^ [Ir(dtbbpy)(ppy)_2_]PF_6_ was used instead of Ir(ppy)_3_.

**Scheme 3:**
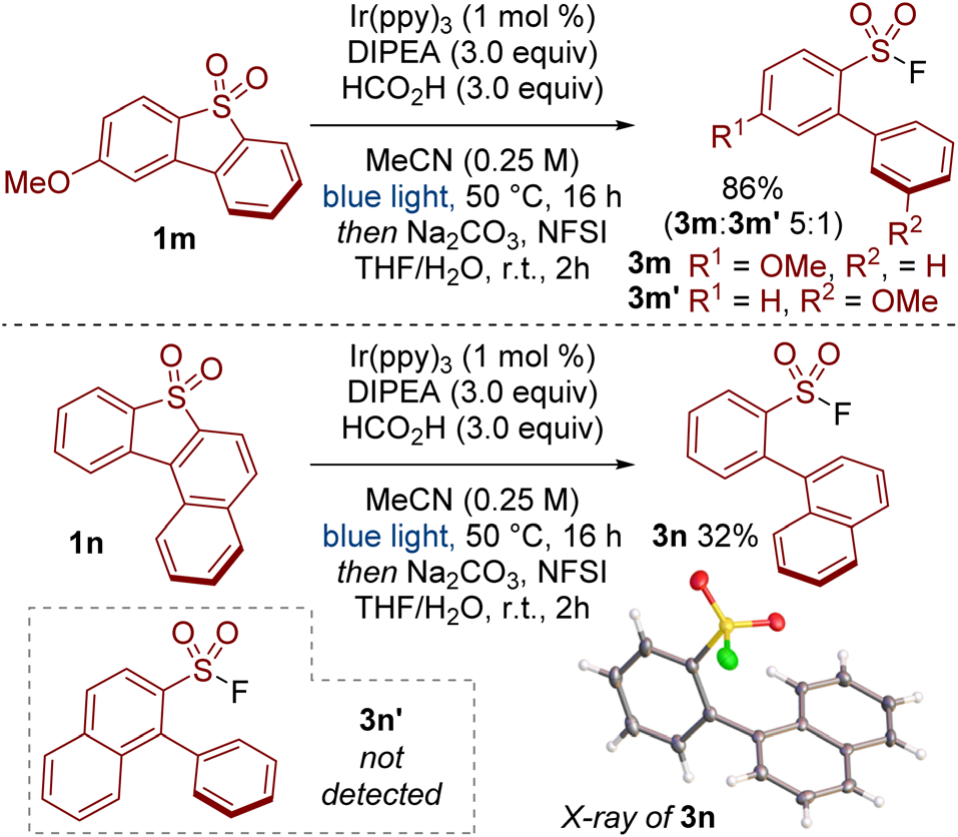
Scope of the photoredox catalysed cleavage of unsymmetrical dibenzothiophene dioxides.

A simplified mechanism for this reaction is provided in Scheme 4A. We propose a reductive quenching cycle in which a highly reducing iridium species Ir(ii) is generated from exposure of the excited state catalyst Ir(iii)* to DIPEA (step i, and ii).^19^ This causes the reduction of the dibenzothiophene dioxide 1 to give the radical anion Int1 (step iii), which subsequently undergoes spontaneous C–S bond cleavage to the radical species Int2 (step iv).^20^ We then tentatively propose that hydrogen atom transfer between Int2 and the radical cation DIPEA˙^+^ provides the sulfinate salt 2 (step v).^21^ Under the standard conditions, the sulfinate salt 2 is transformed into the sulfonyl fluoride 3 through subsequent treatment with NFSI.

**Scheme 4:**
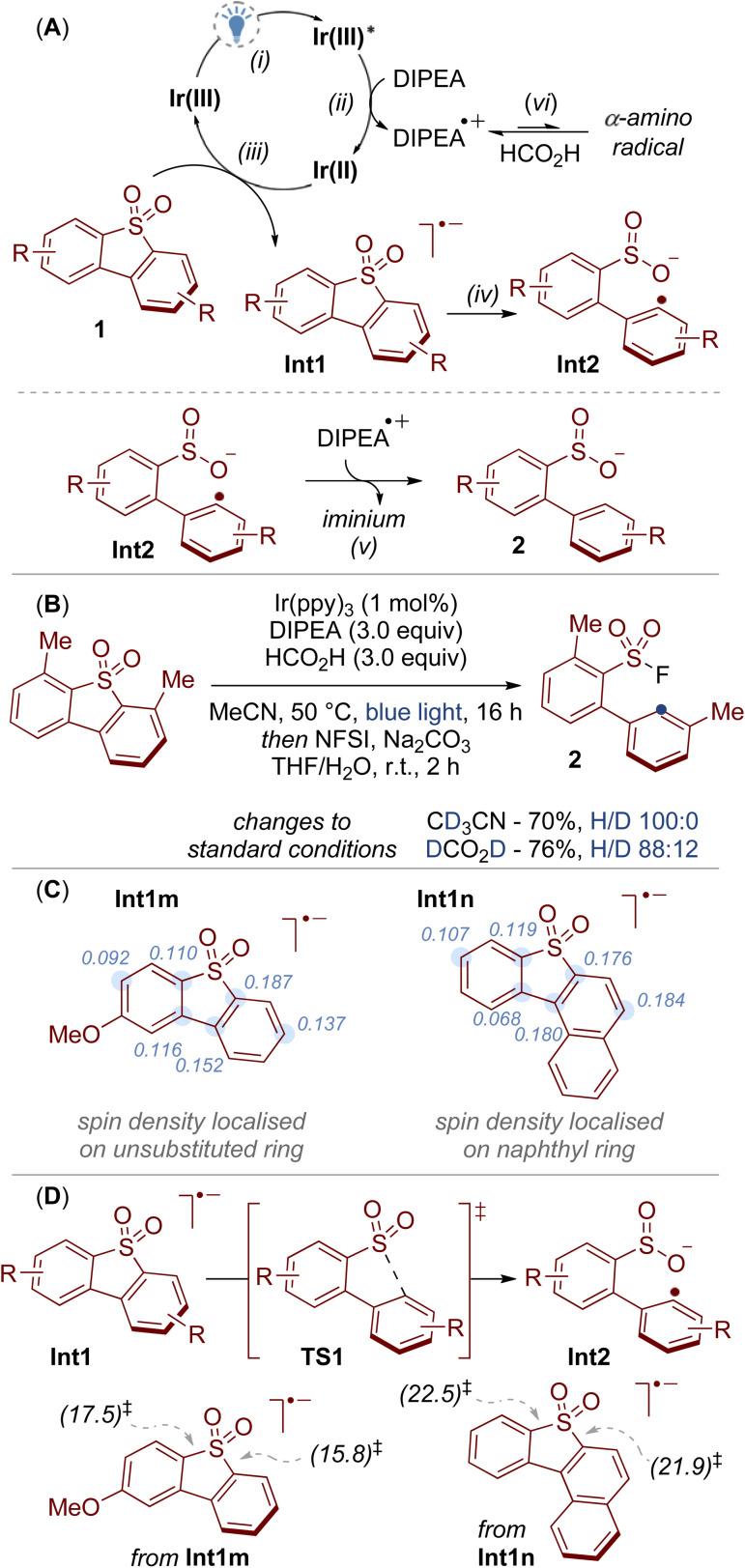
Mechanistic studies. (A) Proposed mechanism for the photoredox catalysed reductive C–S bond cleavage. (B) Deuterium labelling experiments. (C) NBO spin density analysis of radical anion intermediates. Major spin density values are shown. (D) Potential energy diagram for the C–S bond cleavage of unsymmetrical dibenzothiophene dioxides computed at the SMD(MeCN)/M06-2X/6-311++G(d,p)//SMD(MeCN)/B3LYP-D3/6-31+G(d) level of theory. Activation energy barriers (Δ*G*^‡^ [kcal mol^−1^]) are provided in the insert.

To provide support for this mechanism we first conducted a series of deuterium labelling experiments (Scheme 4B). Under otherwise standard conditions, deuterium incorporation was not observed when deuterated solvent (CD_3_CN) was used, and only a small amount (12%) was incorporated when using deuterated formic acid (DCO_2_D). We therefore suggest that DIPEA is the main hydrogen atom source for this reaction, which we tentatively propose is incorporated through hydrogen atom transfer between Int2 and the radical cation of the amine (Scheme 4A, step v).^21^ One possibility for the role of formic acid is that it impedes deprotonation of the radical cation DIPEA˙^+^, thereby lessening the formation of α-amino radicals that may otherwise lead to unproductive side reactions (Scheme 4A, step vi).^22^

Computational studies provided insight into the interesting selectivity we observed with unsymmetrical dibenzothiophene dioxides (*c.f.*Scheme 3). When modelling the radical anion intermediates Int1m and Int1n it was found that the non-bonding orbital (NBO) spin density was localised on the aromatic ring that underwent C–S bond cleavage (Scheme 4C). In addition, DFT calculations suggested that the energy barriers *via* C–S bond cleavage transition state TS1 were lower in energy for the pathways that led to products 3m and 3n in comparison to the pathways towards isomers 3m′ and 3n′ (Scheme 4D). For example, cleavage of the C–S bond that led to the formation of 3m was calculated to have an energy barrier of 15.8 kcal mol^−1^, whereas the pathway to the minor isomer 3m′ required a higher energy input of 17.5 kcal mol^−1^. Thus, calculation of the spin density of the radical anion intermediates Int1 provides a tool for predicting the site selectivity for C–S bond cleavage.

An advantage of our photoredox catalysed methodology over related reductive processes (*c.f.*Scheme 1B) is that the sulfur moiety remains present as a sulfinate at the end of the reaction. In addition to fluorination (Scheme 2), we have shown the utility of the sulfinate products by accessing sulfones 4a and performing a palladium catalysed desulfinylative cross-coupling to give *ortho*-terphenyl 5a (Scheme 5A).^23^

**Scheme 5:**
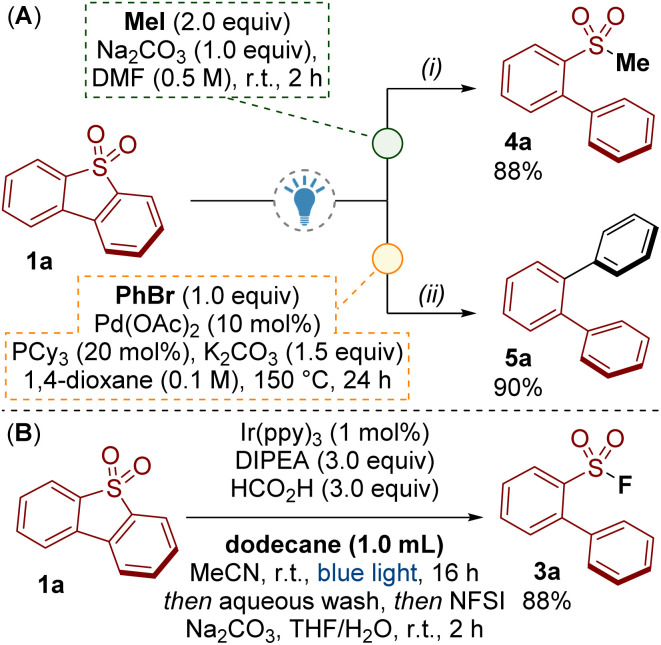
(A) Derivatisations of the sulfinate products. (B) Tolerance of the reaction towards dodecane.

Finally, oxidative desulfurization is a process for removing dibenzothiophene impurities from petroleum *via* oxidation to the dibenzothiophene dioxide and subsequent extraction. Significant achievements have been made in this area though several drawbacks remain, including efficient separation of the dibenzothiophene dioxide from the crude oil.^7^Scheme 5B shows that our method was tolerant of an excess of dodecane oil. In this process, dibenzothiophene 1a was converted to the sulfinate salt 2a, which was easily removed from dodecane by extraction with water. The fluorination step to give 3a was performed to demonstrate the recovery of the sulfinate. Though primitive, transforming dibenzothiophene dioxides 1 into water soluble/oil-immiscible sulfinate salts 2 presents an alternative concept for oxidative desulfurization.

## Conclusions

We have developed a photoredox catalysed reductive cleavage of C–S bonds in dibenzothiophene dioxides 1. The sulfinates 2 that were accessed through this route were transformed into a variety of products, such as sulfonyl fluorides 3, sulfones 4 and biaryls 5. Experimental and computational studies provided insight into the reaction mechanism and the impressive selectivity observed with unsymmetrical substrates. In addition, we designed and developed a 3D printed photoreactor that allowed the temperature of the process to be accurately controlled and offers a practical, accessible and low-cost set-up for performing photochemical reactions at reduced or elevated temperatures.

## Author contributions

GJPP and HY conceptualised the chemical reaction. SW, YS, IYK and GJPP performed the chemical reactions. GJPP and AMN conceptualised the 3D printed photoreactor. TL and AMN developed the 3D printed photoreactor. KY performed DFT calculations. SW and GJPP prepared the SI for the chemical reactions. TL, AMN and GJPP prepared the SI for the 3D printed photoreactor. GJPP wrote the original draft of the manuscript. GJPP, AMN and HY reviewed and edited the manuscript and SI. GJPP and HY obtained funding for the project.

## Conflicts of interest

There are no conflicts of interest to declare.

## Supplementary Material

SC-OLF-D5SC05889A-s001

SC-OLF-D5SC05889A-s002

SC-OLF-D5SC05889A-s003

SC-OLF-D5SC05889A-s004

## Data Availability

CCDC 2427585 (3a) and 2408586 (3n) contain the supplementary crystallographic data for this paper.^18*a*,*b*^ The data supporting this article have been included as part of the supplementary information (SI). Supplementary information is available. See DOI: https://doi.org/10.1039/d5sc05889a.
