# Supplementary material for: Photoredox catalysed reductive cleavage of dibenzothiophene dioxides enabled by a temperature-controlled photoreactor
Source: Chem Sci. 2025 Oct 23;16(48):23246–52. doi: 10.1039/d5sc05889a (PMC12588190; doi:10.1039/d5sc05889a)
Supplement: SC-016-D5SC05889A-s001 [file SC-016-D5SC05889A-s001.pdf]

Supplementary Information (SI) for Chemical Science.
This journal is © The Royal Society of Chemistry 2025

SUPPORTING INFORMATION

Photoredox catalysed reductive cleavage of dibenzothiophene dioxides enabled by a temperature-controlled photoreactor

Siyuan Wang, Quang Truong Le, Yoshiteru Shishido, Ismail Y. Kokculer, Ken Yamazaki, Gregory J. P. Perry,* Adrian M. Nightingale* and Hideki Yorimitsu*

School of Chemistry and Chemical Engineering, University of Southampton, Southampton, SO17 1BJ (UK)

Mechanical Engineering, Faculty of Engineering and Physical Sciences, University of Southampton, Southampton SO17 1BJ (UK)

Department of Chemistry, Graduate School of Science, Kyoto University, Sakyo-ku, Kyoto, 606-8502 (Japan)

Division of Applied Chemistry, Okayama University, Tsushimanaka, Okayama 700-8530, (Japan)

*Corresponding Authors

Gregory Perry: gregory.perry@soton.ac.uk

Adrian Nightingale: a.nightingale@soton.ac.uk

Hideki Yorimitsu: yori@kuchem.kyoto-u.ac.jp

Contents

1) Preparation of substituted dibenzothiophene dioxides (Procedure 1) 4
2) Preparation of 3,7-dibromodibenzo[*b,d*]thiophene 5,5-dioxide (Procedure 2) 11
3) Preparation of 3,7-dibromodibenzo[*b,d*]thiophene (Procedure 3) 12
4) Preparation of methyl substituted dibenzo[*b,d*]thiophene 5,5-dioxides (Procedure 4) 13
5) Preparation of aryl substituted dibenzo[*b,d*]thiophene 5,5-dioxides (Procedure 5) . 15
6) Preparation of 3,7-dihydroxydibenzo[*b,d*]thiophene 5,5-dioxide (Procedure 6) 17
7) Preparation of 3,7-dimethoxydibenzo[*b,d*]thiophene 5,5-dioxide (Procedure 7) 18
8) Preparation of methoxy substituted dibenzo[*b,d*]thiophene 5,5-dioxides (Procedure 8) 19
9) Preparation of 2,8-di(1*H*-pyrazol-1-yl)dibenzo[*b,d*]thiophene (Procedure 9) 21
10) Preparation of 2,8-difluorodibenzo[*b,d*]thiophene (Procedure 10) 22
11) Preparation of dibenzo[*b,d*]thiophene-3,7-dicarboxylic acid 5,5-dioxide (Procedure 11) 23
12) Preparation of diethyl dibenzo[*b,d*]thiophene-3,7-dicarboxylate 5,5-dioxide (Procedure 12) 24
13) Optimization of the photoredoxcatalysed reductive cleavage (Procedure 13) 25
14) Preparation of sulfonyl fluorides and associated characterization data (Procedure 14) 31
15) Deuterium labeling studies with CD_3CN (Procedure 15) 43
16) Deuterium labeling studies using DCO_2D (Procedure 16) 44
17) Preparation of methyl sulfone (Procedure 17) 45
18) Preparation of biaryl (Procedure 18) 46
19) Tolerance of the reaction to oil (Procedure 19) 47
20) Solubility tests 48
21) Cyclic voltammograms 49
22) 3D-Printed photoreactor 51
23) Reproducibility tests 54
24) Computational studies 55
25) NMR Spectra 71
25) References 171

General Experimental

^{1}H (400 MHz), ^{13}C (100 MHz) and $^{19}F\{^{1}H\}$ (376 MHz) NMR measurements were carried out using either a Bruker AVII400 FT-NMR or AVIII HD 400 FT-NMR spectrometer. For ^{13}C NMR spectra, all spectra were recorded with decoupling to 1H unless otherwise noted. Chemical shifts in ^{1}H NMR spectra are reported in delta (δ) units, parts per million (ppm) relative to residual solvent peaks found at δ = 7.26 ppm ($CDCl_3$), δ = 2.50 ppm (DMSO-d_6) or δ = 4.79 ppm (D_2O). Chemical shifts in ^{13}C NMR spectra are reported in delta (δ) units, parts per million (ppm) relative to the residual solvent peaks found at δ = 77.16 ppm ($CDCl_3$) or δ = 39.52 ppm (DMSO-d_6). All J coupling constants were measured in Hertz. The following abbreviations are used for spin multiplicity: s = singlet, d = doublet, t = triplet, q = quartet, m = multiplet, br = broad.

High resolution electrospray ionisation (ESI) samples were analysed using a MaXis (Bruker Daltonics, Bremen, Germany) time of flight (TOF) mass spectrometer. Samples were introduced to the mass spectrometer via a Dionex Ultimate 3000 autosampler and uHPLC pump. High resolution electron ionisation (EI) mass spectrometry was carried out using a LECO HRT+ (LECO Corporation, St Joseph, Mi, USA) time-of-flight (TOF) mass spectrometer.

Analytical thin layer chromatography (TLC) was performed on Merck precoated analytical plates, 0.25-mm thick, silica gel 60 F254.

Flash chromatography was performed using Silica Gel high purity grade, pore size 60 Å, 230-400 mesh particle size, 40-60 μm particle size, purchased from Merck.

The reaction setup for the photoredox reactions is described in section 22. When required, the reactions for substrate/reagent preparation were heated using DrySyn® blocks. All reagents were bought from commercial suppliers and used without further purification unless otherwise noted. In some cases, we found that the dibenzothiophene dioxide **1a** (1016-05-3) purchased from commercial suppliers gave reduced yields. However, good reactivity was restored by purifying the reagent through column chromatography. We are unsure of the reason for the low yield when using dibenzothiophene dioxide from commercial suppliers and we did not observe any impurities by ^{1}H NMR. One possibility is that the oxidant used during the preparation of the dibenzothiophene dioxide is contaminating the commercially supplied reagent, which is removed upon purification. Anhydrous AcroSeal™ acetonitrile purchased from Thermo Fisher Scientific Inc. was used for the photoredox reactions. The concentration of *n*-BuLi in hexane was determined by titration prior to use.[1]

1) Preparation of substituted dibenzothiophene dioxides (Procedure 1)

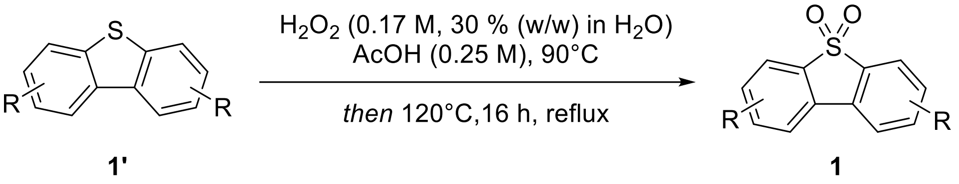

Following a previously reported procedure,[2] a 2-neck round bottom flask equipped with a reflux condenser and stirrer bar was charged with dibenzothiophene (1.0 equiv) and AcOH (25 mL, 0.25 M). The mixture was warmed to 90 °C then hydrogen peroxide (0.17 M, 30 % (w/w) in H_2O) was added and the resulting mixture heated to 120 °C for 16 h. After cooling to room temperature, distilled water was added, and the mixture was filtered. The filter cake was washed with distilled water then dried under vacuum to give the dibenzothiophene dioxide **1**. In some cases, small amounts (<10 %) of the dibenzothiophene sulfoxide was present, in which case the product was purified by column chromatography.

2,8-Dimethyldibenzo[*b*,*d*]thiophene 5,5-dioxide (1b):

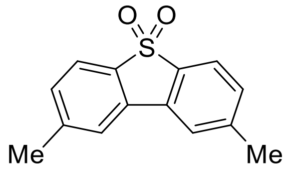

White solid. Obtained in 97% yield (708 mg, 2.9 mmol) from 2,8-dimethyldibenzo[*b*,*d*]thiophene (637 mg, 3.0 mmol, 1.0 equiv), hydrogen peroxide (18 mL, 0.17 M, 30 % (w/w) in H_2O) and AcOH (12 mL, 0.25 M) following **Procedure 1**.

See procedure 4 for the preparation of 2,8-dimethyldibenzo[*b,d*]thiophene.

^{1}H NMR (400 MHz, $CDCl_3$): δ 7.69 (d, J = 7.8 Hz, 2H, Ar*H*), 7.57 (s, 2H, Ar*H*), 7.31 (d, J = 7.8 Hz, 2H, Ar*H*), 2.48 (s, 6H, ArC*H₃*).

^{13}C NMR (101 MHz, $CDCl_3$): δ 144.8 (C_{Ar}), 135.7 (C_{Ar}), 132.1 (C_{Ar}), 131.0 (CH_{Ar}), 122.1 (CH_{Ar}), 122.1 (CH_{Ar}), 22.1 (ArCH_3).

All the resonances in the ^{1}H and ^{13}C NMR spectra were consistent with the reported values.[3]

4,6-Dimethyldibenzo[*b*,*d*]thiophene 5,5-dioxide (1c):

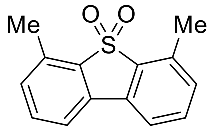

White solid. Obtained in 90% yield (1.32 g, 5.4 mmol) from 4,6-dimethyldibenzo[*b*,*d*]thiophene (1.27 g, 6.0 mmol, 1.0 equiv), hydrogen peroxide (35 mL, 0.17 M, 30 % (w/w) in H_2O) and AcOH (24 mL, 0.25 M) following **Procedure 1**.

4,6-Dimethyldibenzo[*b,d*]thiophene was purchased from a commercial supplier and used without further purification.

^{1}H NMR (400 MHz, $CDCl_3$): δ 7.56 (d, J = 7.7 Hz, 2H, Ar*H*), 7.47 (app t, J = 7.6 Hz, 2H, Ar*H*), 7.24 (d, J = 7.6 Hz, 2H, Ar*H*), 2.71 (s, 6H, ArCH_3).

^{13}C NMR (101 MHz, $CDCl_3$): δ 135.9 (C_{Ar}), 135.8 (C_{Ar}), 133.5 (CH_{Ar}), 132.2 (C_{Ar}), 132.1 (CH_{Ar}), 118.9 (CH_{Ar}), 17.0 (ArCH_3).

All the resonances in the ^{1}H and ^{13}C NMR spectra were consistent with the reported values.[4]

3,7-Dimethyldibenzo[*b*,*d*]thiophene 5,5-dioxide (1d):

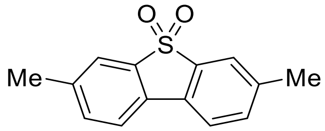

White solid. Obtained in 85% yield (415 mg, 1.7 mmol) from 3,7-dimethyldibenzo[*b*,*d*]thiophene (425 mg, 2.0 mmol, 1.0 equiv) and hydrogen peroxide (12 mL, 0.17 M, 30% (w/w) in H_2O) and AcOH (8 mL, 0.25 M) following **Procedure 1**.

See procedure 4 for the preparation of 3,7-dimethyldibenzo[*b,d*]thiophene.

^{1}H NMR (400 MHz, DMSO-d_6): δ 8.01 (d, J = 7.9 Hz, 2H, Ar*H*), 7.74 – 7.76 (m, 2H, Ar*H*), 7.59 (ddd, J = 8.0, 1.5, 0.7 Hz, 2H, Ar*H*), 2.43 (s, 6H, ArCH_3).

^{13}C NMR (101 MHz, $CDCl_3$): δ 140.7 (C_{Ar}), 137.8 (C_{Ar}), 134.6 (CH_{Ar}), 129.3 (C_{Ar}), 122.4 (CH_{Ar}), 121.2 (CH_{Ar}), 21.5 (ArCH_3).

All the resonances in the ^{1}H and ^{13}C NMR spectra were consistent with the reported values.[5]

2,8-Diphenyldibenzo[*b*,*d*]thiophene 5,5-dioxide (1e):

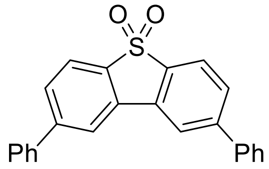

White solid. Obtained in 95% yield (2.10 g, 5.7 mmol) from 2,8-diphenyldibenzo[*b*,*d*]thiophene (2.02 g, 6.0 mmol, 1.0 equiv) and hydrogen peroxide (35 mL, 0.17 M, 30% (w/w) in H_2O) and AcOH (24 mL, 0.25 M) following **Procedure 1**.

See procedure 5 for the preparation of 2,8-diphenyldibenzo[*b,d*]thiophene.

^{1}H NMR (400 MHz, $CDCl_3$): δ 8.03 (d, J = 1.5 Hz, 2H, Ar*H*), 7.91 (dd, J = 8.1, 0.6 Hz, 2H, Ar*H*), 7.74 (dd, J = 8.0, 1.5 Hz, 2H, Ar*H*), 7.67 – 7.64 (m, 4H, Ar*H*), 7.54 – 7.44 (m, 6H, Ar*H*).

^{13}C NMR (101 MHz, $CDCl_3$): δ 147.6 (C_{Ar}), 139.5 (C_{Ar}), 137.0 (C_{Ar}), 132.4 (C_{Ar}), 129.5 (CH_{Ar}), 129.3 (CH_{Ar}), 129.0 (CH_{Ar}), 127.5 (CH_{Ar}), 122.7 (CH_{Ar}), 120.4 (CH_{Ar}).

All the resonances in the ^{1}H and ^{13}C NMR spectra were consistent with the reported values.[6]

2,8-Bis(4-(trifluoromethyl)phenyl)dibenzo[*b*,*d*]thiophene 5,5-dioxide (1f):

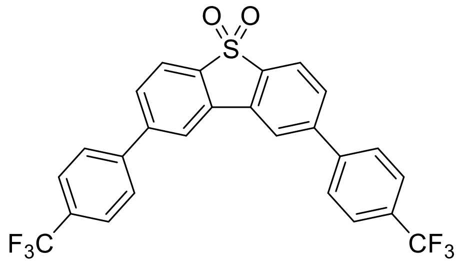

White solid. Obtained in 86% yield (1.26 g, 2.5 mmol) from 2,8-bis(4-(trifluoromethyl)phenyl)dibenzo[*b*,*d*]thiophene (1.37 g, 2.9 mmol, 1.0 equiv) and hydrogen peroxide (17 mL, 0.17 M, 30% (w/w) in H_2O) and AcOH (12 mL, 0.25 M) following **Procedure 1**.

See procedure 5 for the preparation of 2,8-bis(4-(trifluoromethyl) phenyl) dibenzo[*b*,*d*]thiophene.

^{1}H NMR (400 MHz, $CDCl_3$): δ 8.05 (d, J = 1.2 Hz, 2H, Ar*H*), 7.97 (d, J = 8.0 Hz, 2H, Ar*H*), 7.81 – 7.75 (m, 10H, Ar*H*).

^{13}C NMR (101 MHz, $CDCl_3$): δ 1×C_{Ar} missing and 1×CF_3 missing, 146.1 (C_{Ar}), 142.8 (C_{Ar}), 137.8 (C_{Ar}), 132.3 (C_{Ar}), 130.0 (CH_{Ar}), 128.0 (CH_{Ar}), 126.3 (CH_{Ar}), 123.1 (CH_{Ar}), 120.6 (CH_{Ar}).

^{19}F NMR (376 MHz, $CDCl_3$): δ –62.63 (s, 6F, ArCF_3).

2,8-Dimethoxydibenzo[*b*,*d*]thiophene 5,5-dioxide (1g):

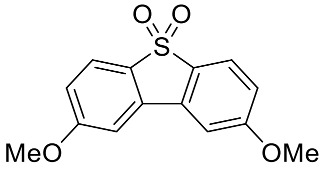

White solid. Obtained in 80% yield (1.33 g, 4.8 mmol) from 2,8-dimethoxydibenzo[*b*,*d*]thiophene(1.47 g, 6.0 mmol, 1.0 equiv) and hydrogen peroxide (35 mL, 0.17 M, 30 % (w/w) in H_2O) and AcOH (24 mL, 0.25 M) following **Procedure 1**.

See procedure 8 for the preparation of 2,8-dimethoxydibenzo[*b,d*]thiophene.

^{1}H NMR (400 MHz, $CDCl_3$): δ 7.73 (d, J = 8.5 Hz, 2H, Ar*H*), 7.20 (d, J = 2.3 Hz, 2H, Ar*H*), 6.99 (dd, J = 8.5, 2.3 Hz, 2H, Ar*H*), 3.93 (s, 6H, ArO*CH₃*).

^{13}C NMR (101 MHz, $CDCl_3$): δ 164.3 (C_{Ar}), 133.9 (C_{Ar}), 131.0 (C_{Ar}), 123.8 (CH_{Ar}), 115.5 (CH_{Ar}), 107.3 (CH_{Ar}), 56.1 (ArOCH_3).

HRMS (EI, m/z): Calculated for $[C_{14}H_{12}O_4S]^+$: 276.0451; Found: 276.0453.

2,8-Di(1*H*-pyrazol-1-yl)dibenzo[*b*,*d*]thiophene 5,5-dioxide (1i):

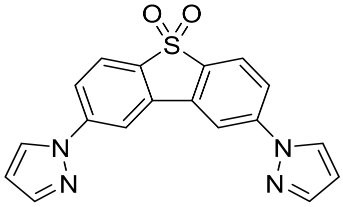

Yellow solid. Obtained in 58% yield (321 mg, 0.92 mmol) from 2,8-di(1*H*-pyrazol-1-yl)dibenzo[*b*,*d*]thiophene (506 mg, 1.6 mmol, 1.0 equiv) and hydrogen peroxide (9 mL, 0.17 M, 30% (w/w) in H_2O) and AcOH (6 mL, 0.25 M) following **Procedure 1**.

See procedure 9 for the preparation of 2,8-di(1*H*-pyrazol-1-yl)dibenzo[*b*,*d*]thiophene.

^{1}H NMR (400 MHz, $CDCl_3$): δ 8.34 (d, J = 2.0 Hz, 2H, Ar*H*), 8.08 (d, J = 2.6 Hz, 2H, Ar*H*), 7.91 (d, J = 8.4 Hz, 2H, Ar*H*), 7.85 – 7.81 (m, 4H, Ar*H*), 6.60 – 6.58 (m, 2H, Ar*H*).

^{13}C NMR (101 MHz, $CDCl_3$): δ 144.6 (C_{Ar}), 142.7 (C_{Ar}), 135.4 (C_{Ar}), 133.0 (C_{Ar}), 127.2 (CH_{Ar}), 123.7 (CH_{Ar}), 120.1 (CH_{Ar}), 112.2 (CH_{Ar}), 109.4 (CH_{Ar}).

All the resonances in the ^{1}H and ^{13}C NMR spectra were consistent with the reported values.[7]

2,8-Bis(trifluoromethoxy)dibenzo[*b*,*d*]thiophene 5,5-dioxide (1j):

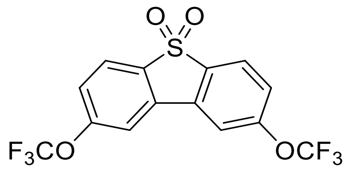

White solid. Obtained in 74% yield (1.11 g, 2.9 mmol) from 2,8-bis(trifluoromethoxy)dibenzo[*b*,*d*]thiophene (1.37 g, 3.9 mmol, 1.0 equiv) and hydrogen peroxide (23 mL, 0.17 M, 30 % (w/w) in H_2O) and AcOH (16 mL, 0.25 M) following **Procedure 1**.

2,8-Bis(trifluoromethoxy)dibenzo[*b*,*d*]thiophene was purchased from a commercial supplier and used without further purification.

^{1}H NMR (400 MHz, $CDCl_3$): δ 7.90 (d, J = 8.4 Hz, 2H, Ar*H*), 7.60 (s, 2H, Ar*H*), 7.43 (dq, J = 8.4, 1.0 Hz, 2H, Ar*H*).

^{13}C NMR (101 MHz, $CDCl_3$): δ 153.6 (q, J = 1.6 Hz, C_{Ar}), 136.5 (C_{Ar}), 132.9 (C_{Ar}), 124.4 (CH_{Ar}), 123.3 (CH_{Ar}), 120.3 (q, J = 260.1 Hz, OCF_3), 114.6 (CH_{Ar}).

^{19}F NMR (376 MHz, $CDCl_3$): δ –57.73 (s, 6F, ArOCF_3).

HRMS (EI, m/z): Calculated for $[C_{14}H_6F_6O_4S]^+$: 383.9886; Found: 383.9904.

2,8-Difluorodibenzo[*b*,*d*]thiophene 5,5-dioxide (1k):

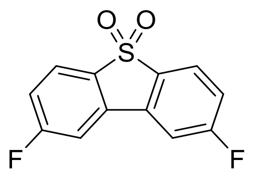

White solid. Obtained in 68% yield (681 mg, 2.7 mmol) from 2,8-difluorodibenzo[*b*,*d*]thiophene (881 mg, 4.0 mmol, 1.0 equiv) and hydrogen peroxide (24 mL, 0.17 M, 30 % (w/w) in H_2O) and AcOH (16 mL, 0.25 M) following **Procedure 1**.

See procedure 10 for the preparation of 2,8-difluorodibenzo[*b*,*d*]thiophene.

^{1}H NMR (400 MHz, DMSO-d_6): δ 8.20 (dd, J = 9.1, 2.4 Hz, 2H, Ar*H*), 8.12 (dd, J = 8.6, 4.9 Hz, 2H, Ar*H*), 7.55 (td, J = 8.7, 2.4 Hz, 2H, Ar*H*).

^{13}C NMR (101 MHz, $CDCl_3$): δ 166.4 (d, J = 256.0 Hz, C_{Ar}), 134.6 (d, J = 3.1 Hz, C_{Ar}), 133.6 (dd, J = 9.9, 2.2 Hz, C_{Ar}), 124.8 (d, J = 9.9 Hz, CH_{Ar}), 118.4 (d, J = 23.8 Hz, CH_{Ar}), 109.7 (d, J = 24.6 Hz, CH_{Ar}).

^{19}F NMR (376 MHz, $CDCl_3$): δ –102.64 (s, 2F, Ar*F*).

All the resonances in the ^{1}H and ^{13}C NMR spectra were consistent with the reported values.[3,8]

2-Methoxydibenzo[*b*,*d*]thiophene 5,5-dioxide (1m):

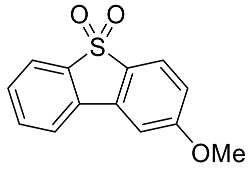

White solid. Obtained in 55% yield (813 mg, 3.3 mmol) from 2-methoxydibenzo[*b*,*d*]thiophene (1.29 g, 6.0 mmol, 1.0 equiv) and hydrogen peroxide (35 mL, 0.17 M, 30 % (w/w) in H_2O) and AcOH (24 mL, 0.25 M) following **Procedure 1**.

See procedure 8 for the preparation of 2-dimethoxydibenzo[*b,d*]thiophene.

^{1}H NMR (400 MHz, $CDCl_3$) δ 7.81 (dd, J = 7.6, 0.6 Hz, 1H, Ar*H*), 7.77 – 7.73 (m, 2H, Ar*H*), 7.63 (app t, J = 7.6 Hz, 1H, Ar*H*), 7.53 (app t, J = 7.6 Hz, 1H, Ar*H*), 7.25 (d, J = 2.3 Hz, 1H, Ar*H*), 7.00 (dd, J = 8.6, 2.3 Hz, 1H, Ar*H*), 3.94 (s, 3H, ArOCH_3).

^{13}C NMR (101 MHz, $CDCl_3$): δ 164.4 (C_{Ar}), 139.0 (C_{Ar}), 134.2 (C_{Ar}), 133.8 (CH_{Ar}), 131.5 (C_{Ar}), 130.7 (CH_{Ar}), 129.8 (C_{Ar}), 124.0 (CH_{Ar}), 122.2 (CH_{Ar}), 121.6 (CH_{Ar}), 115.5 (CH_{Ar}), 107.3 (CH_{Ar}), 56.1 (ArOCH_3).

All the resonances in the ^{1}H and ^{13}C NMR spectra were consistent with the reported values.[9]

Benzo[*b*]naphtho[1,2-*d*] thiophene 7,7-dioxide (1n):

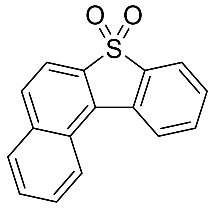

White solid. Obtained in 91% yield (1.94 g, 7.3 mmol) from benzo[*b*]naphtho[1,2-*d*]thiophene (1.87 g, 8.0 mmol, 1.0 equiv) and hydrogen peroxide (47 mL, 0.17 M, 30 % (w/w) in H_2O) and AcOH (32 mL, 0.25 M) following **Procedure 1**.

Benzo[*b*]naphtho[1,2-*d*]thiophene was purchased from a commercial supplier and used without further purification.

^{1}H NMR (400 MHz, $CDCl_3$): δ 8.73 (d, J = 8.6 Hz, 1H, Ar*H*), 8.46 (d, J = 8.1 Hz, 1H, Ar*H*), 8.04 (d, J = 8.3 Hz, 1H, Ar*H*), 8.01−7.98 (m, 1H, Ar*H*), 7.93 (ddd, J = 7.6, 1.3, 0.6 Hz, 1H, Ar*H*), 7.88 (d, J = 8.4 Hz, 1H, Ar*H*), 7.78−7.66 (m, 3H, Ar*H*), 7.57 (app td, J = 7.6, 0.9 Hz, 1H, Ar*H*).

^{13}C NMR (101 MHz, $CDCl_3$): δ 1×C_{Ar} missing, 138.5 (C_{Ar}), 136.9 (C_{Ar}), 136.2 (C_{Ar}), 134.1 (CH_{Ar}), 132.9 (C_{Ar}), 132.2 (CH_{Ar}), 130.2 (CH_{Ar}), 129.8 (CH_{Ar}), 129.1 (CH_{Ar}), 128.5 (CH_{Ar}), 128.2 (C_{Ar}), 125.5 (CH_{Ar}), 124.9 (CH_{Ar}), 122.6 (CH_{Ar}), 117.2 (CH_{Ar}).

All the resonances in the ^{1}H and ^{13}C NMR spectra were consistent with the reported values.[6]

2,8-Dibromodibenzo[*b*,*d*]thiophene 5,5-dioxide (1o):

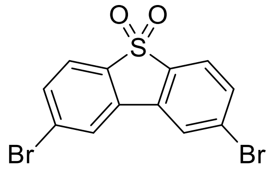

White solid. Obtained in 94% yield (3.52 g, 9.4 mmol) from 2,8-dibromodibenzo[*b*,*d*]thiophene (3.42 g, 10.0 mmol, 1.0 equiv) and hydrogen peroxide (59 mL, 0.17 M, 30 % (w/w) in H_2O) and AcOH (40 mL, 0.25 M) following **Procedure 1**.

2,8-Dibromodibenzo[*b*,*d*]thiophene was purchased from a commercial supplier and used without further purification.

^{1}H NMR (400 MHz, $CDCl_3$): δ 7.93 (dd, J = 1.3, 0.8 Hz, 2H, Ar*H*), 7.71 – 7.70 (m, 4H, Ar*H*).

^{13}C NMR (101 MHz, $CDCl_3$): δ 137.0 (C_{Ar}), 134.2 (CH_{Ar}), 132.4 (C_{Ar}), 129.0 (C_{Ar}), 125.3 (CH_{Ar}), 123.8 (CH_{Ar}).

All the resonances in the ^{1}H and ^{13}C NMR spectra were consistent with the reported values.[10]

2) Preparation of 3,7-dibromodibenzo[*b,d*]thiophene 5,5-dioxide (Procedure 2)

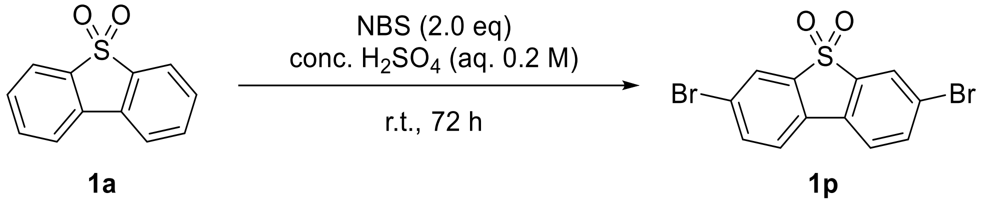

Following a previously reported procedure,[11] dibenzo[*b*,*d*]thiophene 5,5-dioxide (649 mg, 3.0 mmol, 1.0 equiv) was dissolved in concentrated sulfuric acid (aq., 15 mL, 0.2 M) in a round bottom flask. *N*-Bromosuccinimide (1.07 g, 6.0 mmol, 2.0 equiv) was added in small portions, and the reaction was stirred at room temperature for 72 h. Distilled water was then added, and the mixture was filtered. The filter cake was washed with distilled water then dried under vacuum. The product was purified by column chromatography on silica gel (hexane/EtOAc gradient = 98:2 to 95:5) to give 3,7-dibromodibenzo[*b,d*]thiophene 5,5-dioxide as a white powder (1.08 g, 2.9 mmol, 97%).

^{1}H NMR (400 MHz, $CDCl_3$): δ 7.93 (d, J = 1.6 Hz, 2H, Ar*H*), 7.77 (dd, J = 8.2, 1.7 Hz, 2H, Ar*H*), 7.64 (d, J = 8.2 Hz, 2H, Ar*H*).

^{13}C NMR (101 MHz, $CDCl_3$): δ 139.1 (C_{Ar}), 137.3 (CH_{Ar}), 129.7 (C_{Ar}), 125.7 (CH_{Ar}), 124.8 (C_{Ar}), 123.1 (CH_{Ar}).

All the resonances in the ^{1}H and ^{13}C NMR spectra were consistent with the reported values.[11,12]

3) Preparation of 3,7-dibromodibenzo[*b,d*]thiophene (Procedure 3)

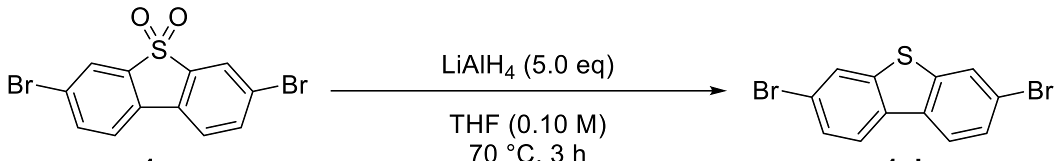

1p **1p'**

Following a previously reported procedure,[13] a round bottom flask equipped with a reflux condenser was charged with 3,7-dibromodibenzo[*b*,*d*]thiophene 5,5-dioxide (1.12 g, 3.0 mmol, 1.0 equiv, see procedure 2 for the preparation of 3,7-dibromodibenzo[*b,d*]thiophene 5,5-dioxide) and anhydrous THF (0.10 M, 30 mL). $LiAlH_4$ (569 mg, 15.0 mmol, 5.0 equiv) was added portion-wise at room temperature and then the mixture was warmed to reflux (70 °C) and stirred for 3 h. The reaction mixture was cooled to 0 °C and cold water was slowly added to quench excess $LiAlH_4$. The mixture was acidified with concentrated HCl to pH 3 and extracted with diethyl ether (×3). The organic layers were combined and dried over anhydrous Na_2SO_4, filtered, and then the solvent was removed by evaporation under a reduced pressure. The crude product was purified by column chromatography on silica gel (hexane/EtOAc gradient = 9.8:0.2 to 9.5:0.5), and compound **1p'** was obtained as a pale-yellow powder (992 mg, 2.9 mmol, 97%).

^{1}H NMR (400 MHz, $CDCl_3$): δ 7.96 (d, J = 1.8 Hz, 2H, ArH), 7.93 (d, J = 8.4 Hz, 2H, ArH), 7.56 (dd, J = 8.4, 1.8 Hz, 2H, ArH).

^{13}C NMR ($CDCl_3$): δ 141.0 (C_{Ar}), 133.8 (C_{Ar}), 128.2 (CH_{Ar}), 125.6 (CH_{Ar}), 122.7 (CH_{Ar}), 120.9 (C_{Ar}).

All the resonances in the ^{1}H and ^{13}C NMR spectra were consistent with the reported values.[13]

4) Preparation of methyl substituted dibenzo[*b,d*]thiophene 5,5-dioxides (Procedure 4)

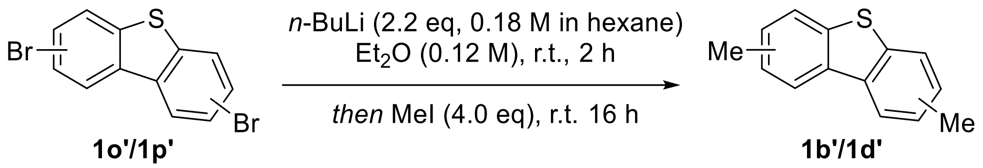

Following a previously reported procedure,[14] a heat-gun dried round bottom flask was charged with dibromodibenzothiophene (1.0 equiv), and anhydrous Et_2O (0.12 M) under a nitrogen atmosphere. Then *n*-butyllithium (2.2 equiv, 1.8 M in hexane) was added dropwise at room temperature. The mixture was stirred at room temperature for 2 h. Subsequently, methyl iodide (4.0 equiv) was added in one portion and reacted overnight (Note: for reasons unknown, the reaction failed if methyl iodide was added dropwise. Take care when adding the methyl iodide in one portion as the reaction mixture becomes warm). After removing the solvent under vacuum, H_2O was added and the product extracted with CH_2Cl_2 (×3) The organic portions were combined and washed with brine before drying with anhydrous Na_2SO_4. Then the solvent was removed under vacuum and the residue purified by column chromatography.

2,8-Dimethyldibenzo[*b,d*]thiophene (1b'):

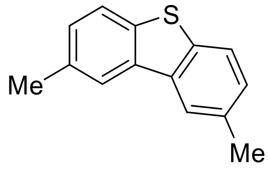

White solid. Obtained in 83% yield (531 mg, 2.5 mmol) from 2,8-dibromodibenzo[*b*,*d*]thiophene (1.03 g, 3.0 mmol, 1.0 equiv), anhydrous Et_2O (25 mL, 0.12 M), *n*-butyllithium (3.0 mL, 6.6 mmol, 2.2 equiv, 1.8 M in hexane) and methyl iodide (750 µL, 12 mmol, 4.0 equiv) following **Procedure 4.**

R_F: 0.69 (hexane/EtOAc, 98:2).

^{1}H NMR (400 MHz, $CDCl_3$): δ 7.94 (dt, J = 1.6, 0.8 Hz, 2H, Ar*H*), 7.71 (d, J = 8.2 Hz, 2H, Ar*H*), 7.28 – 7.25 (m, 2H, Ar*H*), 2.53 (s, 6H, ArCH_3).

^{13}C NMR (101 MHz, $CDCl_3$): δ 137.0 (C_{Ar}), 135.8 (C_{Ar}), 134.1 (C_{Ar}), 128.2 (CH_{Ar}), 122.6 (CH_{Ar}), 121.9 (CH_{Ar}), 21.6 (ArCH_3).

All the resonances in the ^{1}H and ^{13}C NMR spectra were consistent with the reported values.[15]

3,7-Dimethyldibenzo[*b*,*d*]thiophene (1d'):

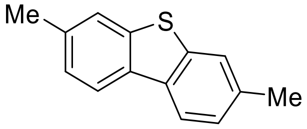

White solid. Obtained in 58% yield (488 mg, 2.3 mmol) from 3,7-dibromodibenzo[*b*,*d*]thiophene (1.37 g, 4.0 mmol, 1.0 equiv, See procedure 3 for the preparation of 3,7-dibromodibenzo[*b,d*]thiophene), anhydrous Et_2O (33 ml, 0.12 M), *n*-butyllithium (4.9 ml, 8.8 mmol, 2.2 equiv, 1.8 M in hexane) and methyl iodide (1.0 ml, 16 mmol, 4.0 equiv) following **Procedure 4**.

R_F: 0.69 (hexane/EtOAc, 98:2).

^1H NMR (400 MHz, $CDCl_3$): δ 7.99 (d, J = 8.1 Hz, 2H, Ar*H*), 7.64 – 7.63 (m, 2H, Ar*H*), 7.27 – 7.24 (m, 2H, Ar*H*), 2.51 (s, 6H, ArCH_3).

^{13}C NMR (101 MHz, $CDCl_3$): δ 139.5 (C_{Ar}), 136.5 (C_{Ar}), 133.4 (C_{Ar}), 125.9 (CH_{Ar}), 122.9 (CH_{Ar}), 121.0 (CH_{Ar}), 21.8 (ArCH_3).

All the resonances in the ^1H and ^{13}C NMR spectra were consistent with the reported values.[15]

5) Preparation of aryl substituted dibenzo[*b,d*]thiophene 5,5-dioxides (Procedure 5)

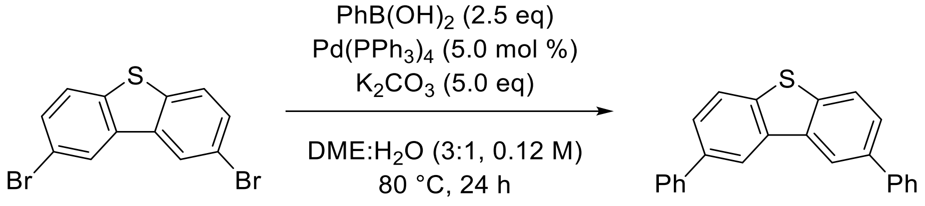

1l' **1e'**

Following a previously reported procedure,[6] a 250 mL 2-neck round bottom flask was charged with 2,8-dibromodibenzo[*b,d*]thiophene (1.0 equiv), phenylboronic acid (2.5 equiv), K_2CO_3 (5.0 equiv) and $Pd(PPh_3)_4$ (5.0 mol %). The mixture was placed under a nitrogen atmosphere by flushing with nitrogen gas. 1,2-Dimethoxyethane and H_2O (DME/H_2O = 3:1, Total = 0.12 M) were added to this mixture. The resulting solution was stirred at 80 °C for 24 h. The reaction mixture was diluted with water and extracted with ethyl acetate (×3). The organic layers were combined, dried over Na_2SO_4, and concentrated under vacuum. The product was isolated using column chromatography on silica gel (Hexane/EtOAc = 9/1).

2,8-Diphenyldibenzo[*b,d*]thiophene (1e):

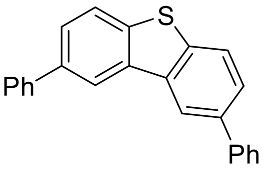

White solid. Obtained in 87% yield (4.27 g, 12.7 mmol) from 2,8-dibromodibenzo[*b,d*]thiophene (4.99 g, 14.6 mmol, 1.0 equiv), phenylboronic acid (4.45 g, 36.5 mmol, 2.5 equiv), K_2CO_3 (10.1 g, 73.0 mmol, 5.0 equiv), $Pd(PPh_3)_4$ (844 mg, 0.73 mmol, 5.0 mol %) and 1,2-dimethoxyethane (90 mL) and H_2O (30 mL) (DME/H_2O = 3:1, Total = 120 mL, 0.12 M) following **Procedure 5**.

^{1}H NMR (400 MHz, $CDCl_3$): δ 8.41 (d, J = 1.5 Hz, 2H, Ar*H*), 7.93 (d, J = 8.4 Hz, 2H, Ar*H*), 7.76 – 7.71 (m, 6H, Ar*H*), 7.54 – 7.49 (m, 4H, Ar*H*), 7.43 – 7.39 (m, 2H, Ar*H*).

^{13}C NMR (101 MHz, $CDCl_3$): δ 141.2 (C_{Ar}), 139.1 (C_{Ar}), 138.1 (C_{Ar}), 136.2 (C_{Ar}), 129.0 (CH_{Ar}), 127.5 (CH_{Ar}), 127.4 (CH_{Ar}), 126.4 (CH_{Ar}), 123.2 (CH_{Ar}), 120.2 (CH_{Ar}).

All the resonances in the ^{1}H and ^{13}C NMR spectra were consistent with the reported values.[16]

2,8-Bis(4-(trifluoromethyl)phenyl)dibenzo[b,d]thiophene (1f):

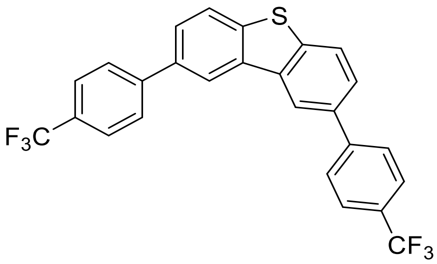

White solid. Obtained in 60% yield (1.42 g, 3.0 mmol) from 2,8-dibromodibenzo[*b*,*d*]thiophene (1.71 g, 5.0 mmol), (4-(trifluoromethyl)phenylboronic acid (2.37 g, 12.5 mmol, 2.5 equiv), K_2CO_3 (3.46 g, 25.0 mmol, 5.0 equiv), $Pd(PPh_3)_4$ (289 mg, 0.25 mmol, 5.0 mol %) and 1,2-dimethoxyethane (32 mL) and H_2O (10 mL) (DME/H_2O = 3:1, Total = 42 mL, 0.12 M) following **Procedure 5**.

^{1}H NMR (400 MHz, $CDCl_3$): δ 8.38 (s, 2H, Ar*H*), 7.94 (d, *J* = 8.3 Hz, 2H, Ar*H*), 7.83−7.69 (m, 10H, Ar*H*).

^{13}C NMR (101 MHz, $CDCl_3$): δ 144.6 (C_{Ar}), 140.1 (C_{Ar}), 136.7 (C_{Ar}), 136.1 (C_{Ar}), 129.6 (q, *J* = 32.8 Hz, C_{Ar}), 127.8 (CH_{Ar}), 126.5 (CH_{Ar}), 126.0 (d, *J* = 3.7 Hz, CH_{Ar}), 124.4 (q, *J* = 271.8 Hz, CF_3), 123.6 (CH_{Ar}), 120.4 (CH_{Ar}).

^{19}F NMR (376 MHz, $CDCl_3$): δ –62.34 (s, 6F, ArCF_3).

6) Preparation of 3,7-dihydroxydibenzo[*b,d*]thiophene 5,5-dioxide (Procedure 6)

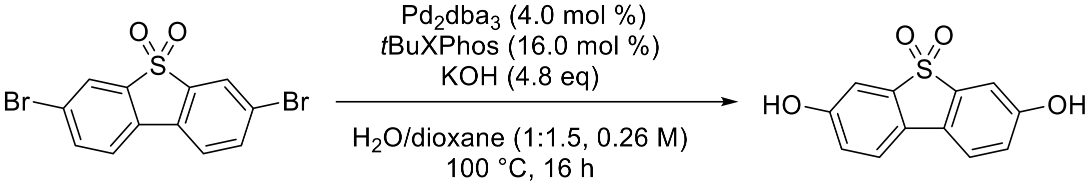

Following a previously reported procedure,[6] a 50 mL round bottom flask equipped with a reflux condenser was charged with 3,7-dibromodibenzo[*b*,*d*]thiophene 5,5-dioxide (**1p**) (965 mg, 2.58 mmol, 1.0 equiv, see procedure 2 for the preparation of 3,7-dibromodibenzo[*b,d*]thiophene 5,5-dioxide), KOH (696 mg, 12.4 mmol, 4.8 equiv), $Pd_2(dba)_3$ (92 mg, 0.10 mmol, 4.0 mol %) and *t*BuXPhos (174 mg, 0.41 mmol, 16.0 mol %). The mixture was placed under a nitrogen atmosphere through vacuum/nitrogen cycles. 1,4-Dioxane (6.0 mL) and H_2O (4.0 mL) (1,4-dioxane/H_2O = 3:2, Total = 10 mL, 0.26 M) were added and the resulting solution was stirred at 100 °C for 16 h. The reaction mixture was cooled to room temperature and acidified with dilute HCl (aq., 0.1 M). The resulting mixture was extracted with ethyl acetate. The organic layers were combined, dried over Na_2SO_4, and concentrated under vacuum. The crude residue was purified using column chromatography on silica gel (Hexane/EtOAc = 4/1) to provide 3,7-dihydroxydibenzo[*b*,*d*]thiophene 5,5-dioxide as a pale-yellow solid (531 mg, 2.14 mmol, 83% yield).

^{1}H NMR (400 MHz, DMSO-d_6): δ 7.80 (d, J = 8.4 Hz, 2H, Ar*H*), 7.15 (d, J = 2.3 Hz, 2H, Ar*H*), 7.07 (dd, J = 8.4, 2.3 Hz, 2H, Ar*H*).

^{13}C NMR (101 MHz, DMSO-d_6): δ 158.5 (C_{Ar}), 138.2 (C_{Ar}), 122.9 (CH_{Ar}), 122.3 (C_{Ar}), 121.2 (CH_{Ar}), 108.1 (CH_{Ar}).

HRMS (ESI, m/z): Calculated for $[C_{12}H_9O_4S]^+$: 249.0216; Found: 249.0214.

7) Preparation of 3,7-dimethoxydibenzo[*b,d*]thiophene 5,5-dioxide (Procedure 7)

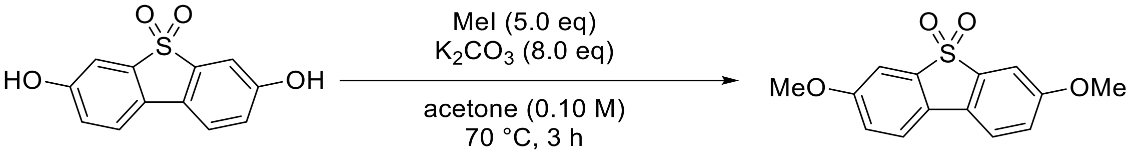

1q **1h**

Following a previously reported procedure,[6] a 100 mL round bottom flask equipped with a reflux condenser was charged with 3,7-dihydroxydibenzo[*b*,*d*]thiophene 5,5-dioxide (290 mg, 1.17 mmol, 1.0 equiv, see procedure 6 for the preparation of 3,7-dihydroxydibenzo[*b,d*]thiophene 5,5-dioxide), MeI (364 µL, 5.85 mmol, 5.0 equiv) and K_2CO_3 (1.29 g, 9.36 mmol, 8.0 equiv). Acetone (12 mL, 0.10 M) was added and the resulting mixture refluxed for 3 h at 70 °C. After cooling to room temperature, the reaction mixture was diluted with water and the product extracted with ethyl acetate (× 3). The organic layers were combined, dried over Na_2SO_4, and concentrated under vacuum. The crude residue was purified using column chromatography on silica gel (Hexane/EtOAc = 4/1) to provide 3,7-dimethoxydibenzo[*b*,*d*]thiophene 5,5-dioxide (**1g**) as a colorless solid (321 mg, 1.16 mmol, 99% yield).

^{1}H NMR (400 MHz, $CDCl_3$): δ 7.57 (d, J = 8.6 Hz, 2H, Ar*H*), 7.28 (d, J = 2.4 Hz, 2H, Ar*H*), 7.10 (dd, J = 8.6, 2.7 Hz, 2H, Ar*H*), 3.88 (s, 6H, ArOC*H₃*).

^{13}C NMR (101 MHz, $CDCl_3$): δ 160.7 (C_{Ar}), 139.1 (C_{Ar}), 124.4 (C_{Ar}), 122.1 (CH_{Ar}), 120.8 (CH_{Ar}), 106.7 (CH_{Ar}), 56.1 (ArOCH_3).

All the resonances in the ^{1}H and ^{13}C NMR spectra were consistent with the reported values.[6]

8) Preparation of methoxy substituted dibenzo[*b,d*]thiophene 5,5-dioxides (Procedure 8)

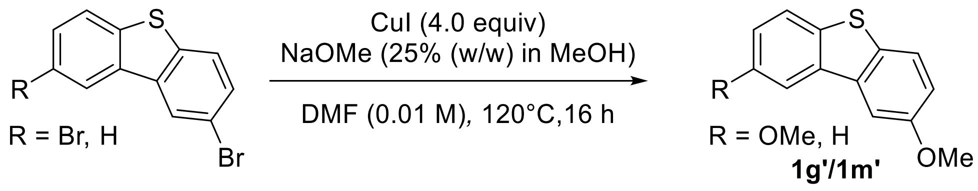

Following a previously reported procedure,[17] a round bottom flask was charged with (di)bromodibenzothiophene (1.0 equiv), CuI (4.0 equiv), DMF (1.0 M) and NaOMe (0.19 M, 25% (w/w) in MeOH). The mixture was warmed to 120 °C and stirred for 16 h. After cooling to room temperature, the reaction was quenched with saturated NH_4Cl (aq.), and the product was extracted with CH_2Cl_2 (× 3). The organic layers were combined, dried over Na_2SO_4, and concentrated under vacuum. The crude residue was purified using column chromatography on silica gel to provide the pure product.

2,8-Dimethoxydibenzo[*b*,*d*]thiophene (1g'):

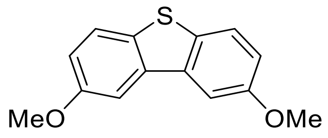

White solid. Obtained in 84% yield (3.01 g, 12.3 mmol) from 2,8-dibromodibenzo[*b*,*d*]thiophene (5.0 g, 14.6 mmol, 1.0 equiv), CuI (11.1 g, 58.4 mmol, 4.0 equiv), DMF (15 mL, 1.0 M) and NaOMe (77 mL, 0.19 M, 25% (w/w) in MeOH,) following **Procedure 8**.

R_F: 0.50 (hexane/EtOAc, 4:1).

^{1}H NMR (400 MHz, $CDCl_3$): δ 7.70 (d, J = 8.7 Hz, 2H, Ar*H*), 7.56 (d, J = 2.6 Hz, 2H, Ar*H*), 7.09 (dd, J = 8.8, 2.6 Hz, 2H, Ar*H*), 3.95 (s, 6H, ArOC*H*$_3$).

^{13}C NMR (101 MHz, $CDCl_3$): δ 157.7 (C_{Ar}), 136.6 (C_{Ar}), 132.6 (C_{Ar}), 123.8 (CH_{Ar}), 116.0 (CH_{Ar}), 105.0 (CH_{Ar}), 55.9 (ArO*C*H$_3$).

All the resonances in the ^{1}H and ^{13}C NMR spectra were consistent with the reported values.[17]

2-Methoxydibenzo[*b*,*d*]thiophene (1m'):

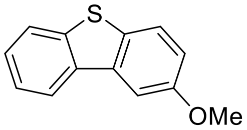

White solid. Obtained in 58% yield (1.82 g, 8.5 mmol) from 2-bromodibenzo[*b*,*d*]thiophene (3.84 g, 14.6 mmol, 1.0 equiv), CuI (11.1 g, 58.4 mmol, 4.0 equiv), DMF (15 mL, 1.0 M) and NaOMe (77 mL, 0.19 M, 25% (w/w) in MeOH) following **Procedure 8**.

R_F: 0.50 (hexane/EtOAc, 4:1).

^{1}H NMR (400 MHz, $CDCl_3$): δ 8.14−8.09 (m, 1H, Ar*H*), 7.86−7.81 (m, 1H, Ar*H*), 7.73 (dd, *J* = 8.7, 0.5 Hz, 1H, Ar*H*), 7.63 (d, *J* = 2.4 Hz, 1H, Ar*H*), 7.47−7.42 (m, 2H, Ar*H*), 7.10 (dd, *J* = 8.7, 2.5 Hz, 1H, Ar*H*), 3.95 (s, 3H, ArOCH_3).

^{13}C NMR (101 MHz, $CDCl_3$): δ 157.8 (C_{Ar}), 140.7 (C_{Ar}), 136.8 (C_{Ar}), 135.6 (C_{Ar}), 131.5 (C_{Ar}), 126.8 (CH_{Ar}), 124.3 (CH_{Ar}), 123.6 (CH_{Ar}), 123.1 (CH_{Ar}), 121.7 (CH_{Ar}), 116.0 (CH_{Ar}), 105.1 (CH_{Ar}), 55.9 (ArOCH_3).

All the resonances in the ^{1}H and ^{13}C NMR spectra were consistent with the reported values.[18]

9) Preparation of 2,8-di(1*H*-pyrazol-1-yl)dibenzo[*b*,*d*]thiophene (Procedure 9)

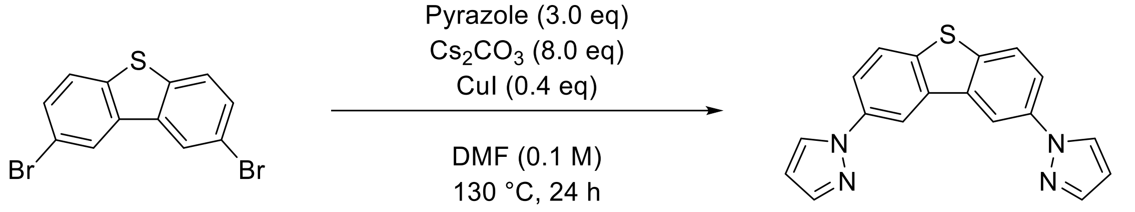

1o' **1i'**

Following a previously reported procedure,[19] a 100 mL round bottom flask was charged with 2,8-dibromodibenzothiophene (1.71 g, 5.0 mmol, 1.0 equiv), pyrazole (1.02 g, 15.0 mmol), Cs_2CO_3 (13.0 g, 40.0 mmol, 8.0 equiv), and CuI (381 mg, 2.0 mmol, 0.4 equiv) were taken in an oven-dried Schlenk flask under an nitrogen atmosphere and DMF (50 mL, 0.1 M) was added. The resulting solution was stirred at 130 °C for 24 h. After this time, the reaction mixture was diluted with cold water and extracted with ethyl acetate. The organic layer was separated, dried over Na_2SO_4, and concentrated under vacuum. The crude residue was purified using column chromatography on silica gel (hexane/ethyl acetate = 9:1) to provide 2,8-bis(1*H*-pyrazol-1-yl)dibenzo[*b*,*d*]thiophene as a colorless solid (633 mg, 2.0 mmol, 40% yield).

^{1}H NMR (400 MHz, $CDCl_3$): δ 8.53 (d, J = 2.1 Hz, 2H, Ar*H*), 8.06 (d, J = 2.5 Hz, 2H, Ar*H*), 7.91 (d, J = 8.6 Hz, 2H, Ar*H*), 7.84 (dd, J = 8.6, 2.1 Hz, 2H, Ar*H*), 7.79 (d, J = 1.8 Hz, 2H, Ar*H*), 6.54 (app t, J = 2.1 Hz, 2H, Ar*H*).

^{13}C NMR (101 MHz, $CDCl_3$): δ 141.4 (CH$_{Ar}$), 138.3 (C_{Ar}), 137.9 (C_{Ar}), 136.3 (C_{Ar}), 127.2 (CH$_{Ar}$), 123.9 (CH$_{Ar}$), 119.1 (CH$_{Ar}$), 112.6 (CH$_{Ar}$), 108.1 (CH$_{Ar}$).

All the resonances in the ^{1}H and ^{13}C NMR spectra were consistent with the reported values.[19]

10) Preparation of 2,8-difluorodibenzo[*b,d*]thiophene (Procedure 10)

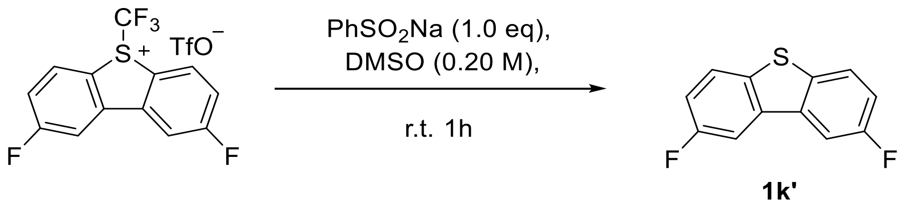

Following a previously reported procedure,[20] a round bottom flask was charged with 2,8-difluoro-5-(trifluoromethyl)-5*H*-dibenzo[*b*,*d*]thiophen-5-ium-trifluoromethanesulfonate (2.63 g, 6.0 mmol, 1.0 equiv) and anhydrous DMSO (30 mL, 0.20 M). $PhSO_2Na$ (985 mg, 6.0 mmol, 1.0 equiv) was added at room temperature and then the mixture was stirred for 1 h. The reaction mixture was quenched with H_2O and extracted with Et_2O (×3). The organic layers were combined, dried over anhydrous Na_2SO_4, filtered, and then the solvent was removed by evaporation under reduced pressure. The crude product was purified by column chromatography on silica gel (Hexane/EtOAc gradient = 9.0:1.0 to 8.0:2.0) to provide compound **1h'** as a white powder (881 mg, 4.0 mmol, 67%).

R_F = 0.69 (hexane/EtOAc, 98:2).

^{1}H NMR (400 MHz, $CDCl_3$): δ 7.76 (dd, *J* = 8.9, 4.6 Hz, 2H, Ar*H*), 7.71 (dd, *J* = 9.1, 2.5 Hz, 2H, Ar*H*), 7.22 (app td, *J* = 8.7, 2.5 Hz, 2H, Ar*H*).

^{13}C NMR (101 MHz, $CDCl_3$): δ 161.0 (d, *J* = 243.1 Hz, C_{Ar}), 136.4 (dd, *J* = 8.8, 3.8 Hz, C_{Ar}), 135.9 (d, *J* = 1.9 Hz, C_{Ar}), 124.2 (d, *J* = 8.9 Hz, CH_{Ar}), 115.7 (d, *J* = 24.6 Hz, CH_{Ar}), 108.2 (d, *J* = 23.5 Hz, CH_{Ar}).

^{19}F NMR (376 MHz, $CDCl_3$): δ –117.89 (s, Ar*F*).

All the resonances in the ^{1}H and ^{13}C NMR spectra were consistent with the reported values.[20]

11) Preparation of dibenzo[*b,d*]thiophene-3,7-dicarboxylic acid 5,5-dioxide (Procedure 11)

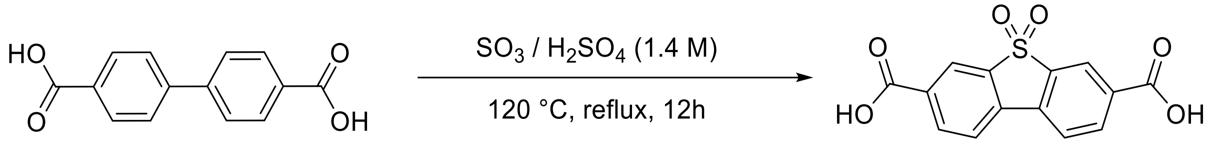

1l'

Following a previously reported procedure,[21] a 10 mL round bottom flask equipped with a reflux condenser was charged with (1,1'-biphenyl)-4,4'-dicarboxylic acid (993 mg, 4.1 mmol, 1.0 equiv) and fuming sulfuric acid (oleum, 20% SO_3, 3.0 mL, 1.4 M). The resulting mixture was refluxed at 120 °C for 12 h. After cooling to room temperature, the solution was poured into a beaker containing 20 ml of distilled water. A precipitate immediately formed which was isolated via suction filtration, washed with copious amounts of H_2O and dried under vacuum to obtain dibenzo[*b*,*d*]thiophene-3,7-dicarboxylic acid 5,5-dioxide (971 mg, 3.2 mmol, 78% yield) as a brown solid.

^{1}H NMR (400 MHz, DMSO-d$_6$): δ 8.43 (d, J = 8.1 Hz, 2H, ArH), 8.37 (dd, J = 6.3, 1.3 Hz, 2H, ArH), 8.35 (d, J = 1.5 Hz, 2H, ArH).

^{13}C NMR (101 MHz, DMSO-d$_6$): δ 165.5 (CO$_2$H), 138.0 (C_{Ar}), 135.6 (CH$_{Ar}$), 133.9 (C_{Ar}), 133.6 (CH$_{Ar}$), 124.1 (C_{Ar}), 122.5 (CH$_{Ar}$).

All the resonances in the ^{1}H and ^{13}C NMR spectrum were consistent with the reported values.[21,22]

12) Preparation of diethyl dibenzo[*b,d*]thiophene-3,7-dicarboxylate 5,5-dioxide (Procedure 12)

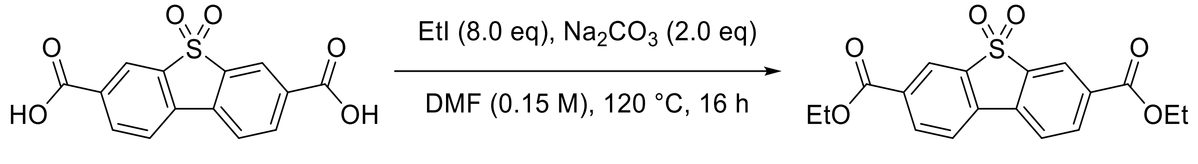

1l' **1l**

A 25 mL round bottom flask was charged with dibenzo[*b*,*d*]thiophene-3,7-dicarboxylic acid 5,5-dioxide (456 mg, 1.5 mmol, 1.0 equiv, see procedure 11 for the preparation of dibenzo[*b*,*d*]thiophene-3,7-dicarboxylic acid 5,5-dioxide), Na_2CO_3 (318 mg, 3.0 mmol, 2.0 equiv), EtI (964 µL, 12 mmol, 8.0 equiv) and DMF (10 ml, 0.15 M). The resulting mixture was refluxed at 120 °C for 16 h. After cooling to room temperature, the resulting solution was quenched with 20 mL distilled water and extracted with CH_2Cl_2 (3 × 20 mL). the organic layers were combined, dried over Na_2SO_4 and concentrated under reduced pressure. The residue was then purified by column chromatography on silica gel (hexane/EtOAc gradient = 9.0:1.0 to 8.0:2.0) to provide compound **1l** as a pale yellow powder (79 mg, 0.22 mmol, 15%).

^{1}H NMR (400 MHz, $CDCl_3$) δ 8.52 (s, 2H, Ar*H*), 8.37 (ddd, *J* = 8.0, 1.5, 0.8 Hz, 2H, Ar*H*), 7.94 (d, *J* = 8.2 Hz, 2H, Ar*H*), 4.45 (q, *J* = 7.1 Hz, 4H, CH_2CH_3), 1.44 (t, *J* = 7.1 Hz, 6H, CH_2CH_3).

^{13}C NMR (101 MHz, $CDCl_3$): *δ* 164.4 ($ArCO_2Et$), 139.0 (C_{Ar}), 135.5 (CH_{Ar}), 134.3 (C_{Ar}), 133.6 (C_{Ar}), 123.9 (CH_{Ar}), 122.4 (CH_{Ar}), 62.2 (OCH_2), 14.4 (CH_3).

HRMS (ESI, m/z): Calculated for $[C_{18}H_{16}O_6SNa]^+$: 383.0560; Found: 383.0560.

13) Optimization of the photoredoxcatalysed reductive cleavage (Procedure 13)

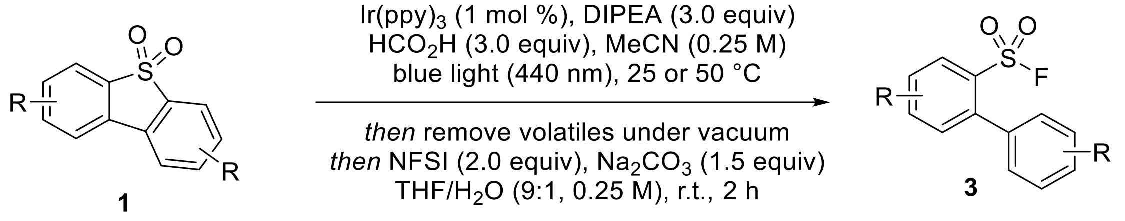

Any changes to the standard reaction conditions are highlighted in the table below. A 10-mL vial was charged with Ir(ppy)$_3$ (3 mg, 0.005 mmol, 1.0 mol %) and dibenzothiophene dioxide **1** (0.50 mmol, 1.0 equiv). The vial was sealed using a septum and the mixture flushed with nitrogen for 15 minutes using a nitrogen balloon. Then MeCN (2.0 mL, 0.25 M), DIPEA (260 µL, 1.5 mmol, 3.0 equiv) and HCO_2H (57 µL, 1.5 mmol, 3.0 equiv) were added (Note: white fumes are produced upon addition of HCO_2H to the mixture containing DIPEA). The mixture was then irradiated with blue light (Kessil, PR160L, 440nm, placed ~10 cm from the vial, see section 22 for further details on the photoreactor set up) at the given temperature (25 or 50 °C) for 16 h. The irradiation was then stopped. The volatile components were removed under vacuum using a rotary evaporator followed by high vacuum for 1 h. The resulting mixture was reconstituted in THF (1.8 mL) and H_2O (0.2 mL) (THF/H_2O = 9:1, Total = 2.0 mL, 0.25 M) then Na_2CO_3 (79 mg, 0.75 mmol, 1.5 equiv) and NFSI (315 mg, 1.0 mmol, 2.0 equiv) were added (Note: the reaction becomes warm upon addition of NFSI, so we recommend this reagent is added portion wise when performing on larger scales). The resulting mixture was stirred at room temperature for 2 h then quenched by addition of H_2O. The mixture was extracted with CH_2Cl_2 (×3), dried over Na_2SO_4, filtered and concentrated under vacuum. The crude mixture was analysed by quantitative ^{1}H NMR using $CHCl_2CHCl_2$ as an internal standard.

Optimization table (Table S1)

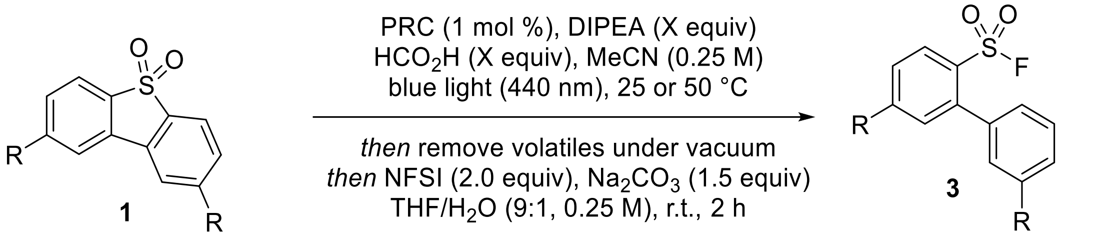

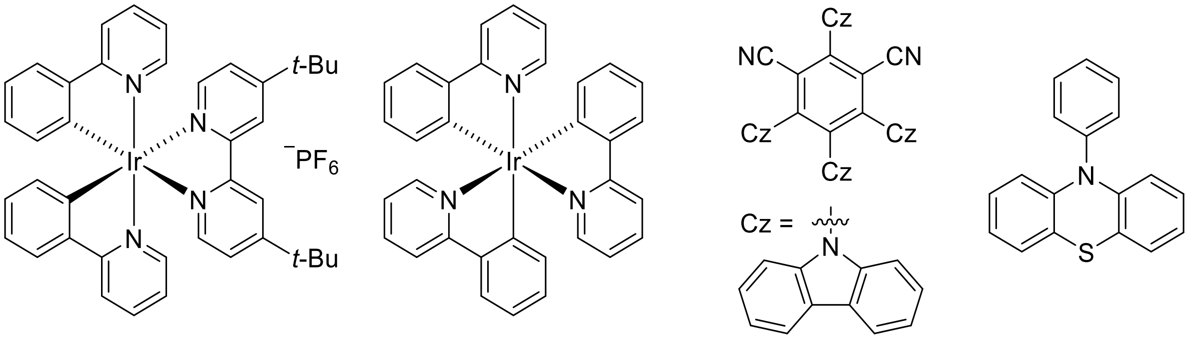

Entry	R	PRC	DIPEA (equiv)	HCO_2H (equiv)	T (°C)	3a/3b (%)[a]
1	**H (1a)**	**Ir1**	4.0	-[b]	**25**	38
2	**H (1a)**	**Ir1**	4.0	4.0	**25**	44
3	**H (1a)**	**Ir2**	4.0	4.0	**25**	96
4	**H (1a)**	**Ir2**	3.0	3.0	**25**	95 (93)[c]
5[d]	**H (1a)**	**Ir2**	3.0	3.0	**25**	95
6[e]	**H (1a)**	**4-CzIPN**	3.0	3.0	**25**	12
7	**H (1a)**	**Ir2**	2.0	2.0	**25**	85[f]
8	**H (1a)**	**Ir2**	1.0	1.0	**25**	34
9	**H (1a)**	**Ir2**	3.0	1.0	**25**	44
10[g]	**H (1a)**	-	3.0	3.0	**25**	14[h]
11[i]	**H (1a)**	**Ir2**	3.0	3.0	**25**	0[j]
12	**Me (1b)**	**Ir2**	3.0	3.0	**25**	18
13	**Me (1b)**	**PTH**[k]	3.0	3.0	**25**	36
14[l]	**Me (1b)**	**Ir2**	3.0	3.0	**25**	32
15	**Me (1b)**	**Ir2**	3.0	3.0	**50**	85 (82)[c]

[a] ^{1}H NMR yields using $CHCl_2CHCl_2$ as an internal standard. Reactions irradiated using a Kessil PR160L 440nm. [b] HCO_2H = 0 equiv, solvent = MeCN/H_2O (6:1, 0.25 M). [c] Yield in parenthesis is isolated material. [d] Selectfluor (1-(chloromethyl)-4-fluoro-1,4-diazabicyclo[2.2.2]octane-

1,4-diium ditetrafluoroborate) was used instead of NFSI (*N*-fluorobenzenesulfonimide). [e] 4-CzIPN (5 mol %). [f] 14% recovered starting material (**1a**). [g] Control experiment – no photoredox catalyst added. [h] 73% recovered starting material (**1a**). [i] Control experiment – no irradiation. The reaction vial was wrapped in foil when performing the reaction. [j] Quantitative recovery of starting material (**1a**). [k] 10-Phenylphenothiazine (PTH, 5 mol %), light = Kessil PR160L 390 nm. [l] MeCN (0.125M).

Solvent screening (Table S2)

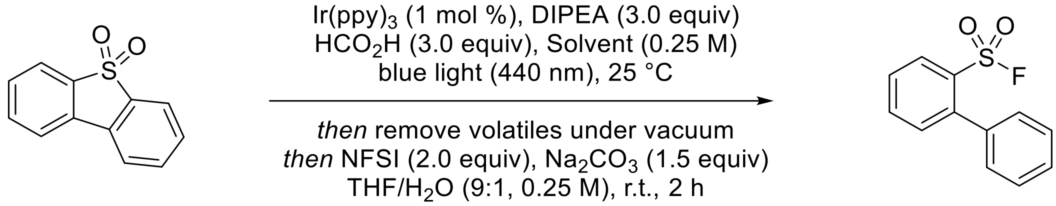

Entry	Solvent	4a (%)[a]
1	**MeCN**	95
2	**DCE**	97
3	**DCM**	0
4	**DMF**	61
5	**DMSO**	71
6	**MeOH**	0
7	***t*-BuOH**	76
8	**MeOH/Acetone (1:1)**	42
9	**DMF/H_2O (9:1)**	60

[a] ^{1}H NMR yields using $CHCl_2CHCl_2$ as an internal standard. Reactions irradiated using a Kessil PR160L 440nm. DCE = 1,2-Dichloroethane. DMF = *N*,*N*-Dimethylformamide. DMSO = Dimethyl sulfoxide.

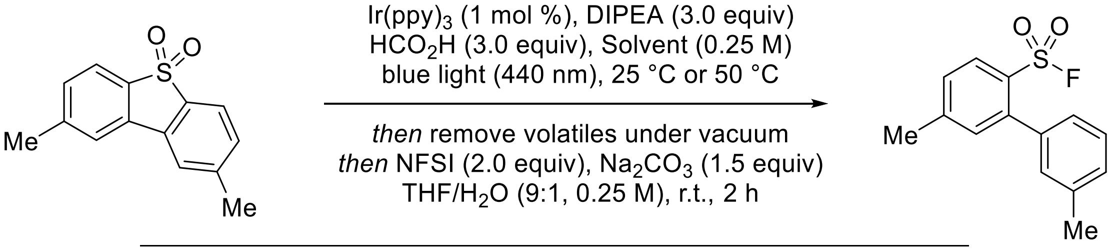

Entry	Solvent	Temp (°C)	4a (%)[a]
1	**MeCN**	25	18
2	**MeCN**	50	82
3	**DCE**	25	36
4	**DCE**	50	72

[a] ^{1}H NMR yields using $CHCl_2CHCl_2$ as an internal standard. Reactions irradiated using a Kessil PR160L 440nm. DCE = 1,2-Dichloroethane.

Acid and amine screening (Table S3)

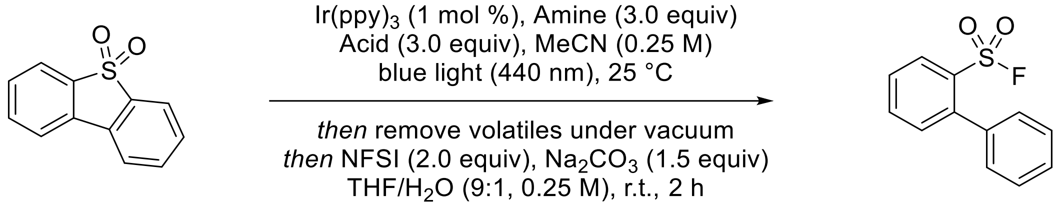

Entry	Amine	Acid	4a (%)[a]
1	**DIPEA**	HCO_2H	95
2	**DIPEA**	CF_3CO_2H	0
3	**DIPEA**	$PhCO_2H$	37
4	**DIPEA**	CH_3CO_2H	58
5	**TMP**	HCO_2H	17

[a] ^{1}H NMR yields using $CHCl_2CHCl_2$ as an internal standard. Reactions irradiated using a Kessil PR160L 440nm. DIPEA = *N*,*N*-Diisopropylethylamine. TMP = 2,2,6,6-Tetramethylpiperidine.

Unsuccessful substrates (Table S4)

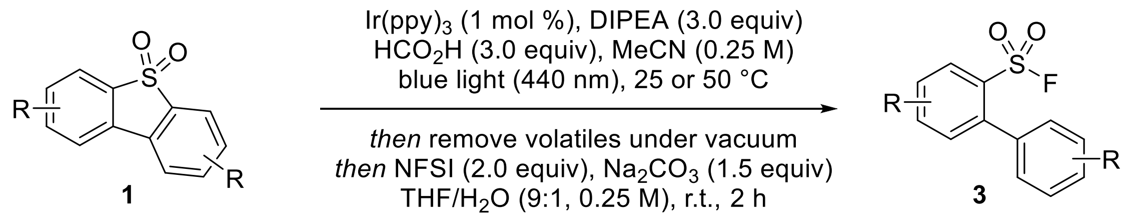

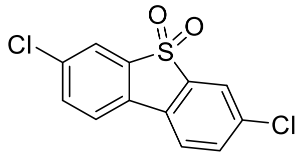

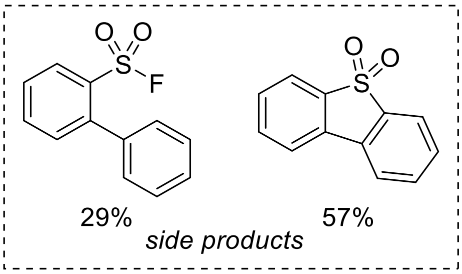

none of the desired product observed, only side products from C–Cl bond cleavage

trying less reducing PRC ([Ir(dtbbpy)(ppy)$_2$]PF$_6$ or (Ir[dF(CF$_3$)ppy]$_2$(dtbpy))PF$_6$) did not improve the result and C–Cl bond cleavage was still observed

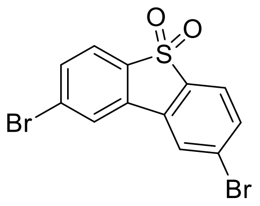

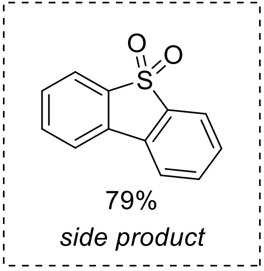

none of the desired product observed, only side product from C–Br bond cleavage

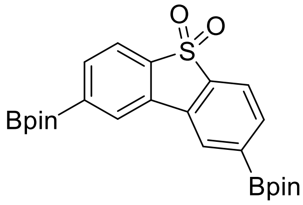

With Ir(ppy)$_3$, a trace of product was observed by GCMS, but we were unable to isolate by column chromatography.

With [Ir(dtbbpy)(ppy)$_2$]PF$_6$, only starting material was recovered (84% rsm)

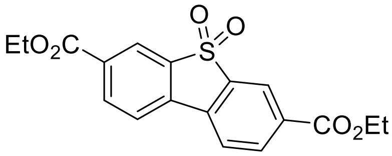

None of the desired product was observed. We were unable to quantify the mass recovery of starting material as the starting material was very insoluble.

Using 1,2-dichloroethane instead of MeCN did not improve the yield.

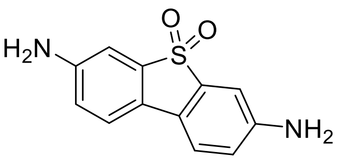

None of the desired product was observed. We were unable to quantify the mass recovery of starting material as the starting material was very insoluble.

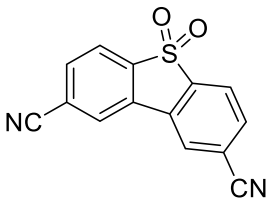

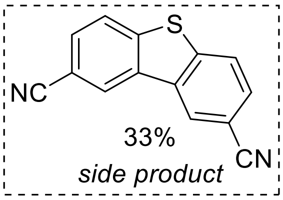

none of the desired product observed, only side product from S=O bond cleavage

14) Preparation of sulfonyl fluorides and associated characterization data (Procedure 14)

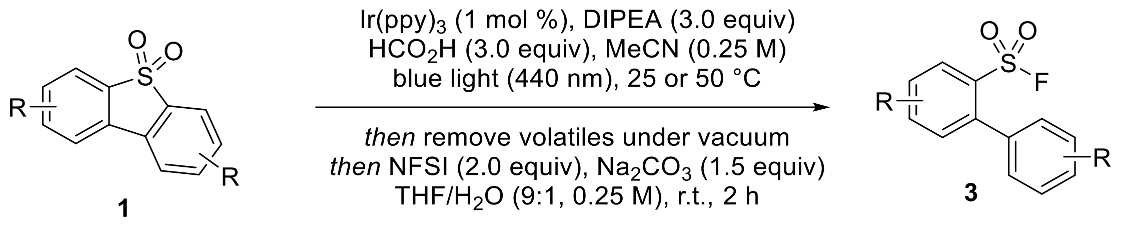

A 10-mL vial was charged with Ir(ppy)$_3$ (3 mg, 0.005 mmol, 1.0 mol %) and dibenzothiophene dioxide **1** (0.50 mmol, 1.0 equiv). The vial was sealed using a septum and the mixture flushed with nitrogen for 15 minutes using a nitrogen balloon. Then MeCN (2.0 mL, 0.25 M), DIPEA (260 µL, 1.5 mmol, 3.0 equiv) and HCO_2H (57 µL, 1.5 mmol, 3.0 equiv) were added (Note: white fumes are produced upon addition of HCO_2H to the mixture containing DIPEA). The mixture was then irradiated with blue light (Kessil, PR160L, 440nm, placed ~10 cm from the vial, see section 22 for further details on the photoreactor set up) at the given temperature (25 or 50 °C) for 16 h. The irradiation was then stopped. The volatile components were removed under vacuum using a rotary evaporator followed by high vacuum for 1 h. The resulting mixture was reconstituted in THF (1.8 mL) and H_2O (0.2 mL) (THF/H_2O = 9:1, Total = 2.0 mL, 0.25 M) then Na_2CO_3 (79 mg, 0.75 mmol, 1.5 equiv) and NFSI (315 mg, 1.0 mmol, 2.0 equiv) were added (Note: the reaction becomes warm upon addition of NFSI, so we recommend this reagent is added portion wise when performing on larger scales). The resulting mixture was stirred at room temperature for 2 h then quenched by addition of H_2O. The mixture was extracted with CH_2Cl_2 (×3), dried over Na_2SO_4, filtered and concentrated under vacuum. The crude mixture was purified by silica gel column chromatography to yield the desired sulfonyl fluoride product **3**.

[1,1'-Biphenyl]-2-sulfonyl fluoride (3a):

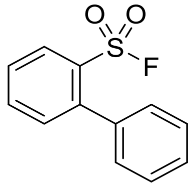

White solid. Obtained in 93% yield (109 mg, 0.46 mmol) from **1a** (108 mg, 0.5 mmol, 1.0 equiv) following **Procedure 14**.

Reaction temperature: 25 °C.

Reaction vial: 10 mL CEM® microwave vial.

R_F: 0.69 (hexane/EtOAc, 19:1).

^{1}H NMR (400 MHz, $CDCl_3$): δ 8.18 (d, J = 8.1 Hz, 1H, ArH), 7.77 (app td, J = 7.6, 1.4 Hz, 1H, ArH), 7.61 (tt, J = 8.0, 1.4 Hz, 1H, ArH), 7.51−7.44 (m, 4H, ArH), 7.43−7.37 (m, 2H, ArH).

^{13}C NMR (101 MHz, $CDCl_3$): δ 143.2 (C_{Ar}), 138.0 (C_{Ar}), 134.9 (CH_{Ar}), 133.2 (CH_{Ar}), 132.5 (d, J = 22.0 Hz, C_{Ar}), 130.1 (CH_{Ar}), 129.0 (CH_{Ar}), 128.7 (CH_{Ar}), 128.2 (CH_{Ar}), 128.1 (CH_{Ar}).

^{19}F NMR (376 MHz, $CDCl_3$): δ 67.40 (s, $ArSO_2F$).

All the resonances in the ^{1}H and ^{13}C NMR spectra were consistent with the reported values.[23]

X-Ray Crystallography:

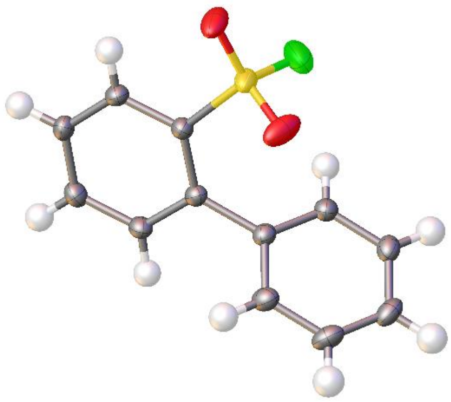

Experimental. Single clear colourless plate-shaped crystals of **2a** recrystallised from a mixture of hexane and DCM by slow evaporation. A suitable crystal with dimensions 0.25 × 0.07 × 0.04 mm3 was selected and mounted on a MITIGEN holder with silicon oil on a ROD, Synergy Custom system, HyPix-CAr 100 diffractometer. The crystal was kept at a steady T = 100(2) K during data collection. The structure was solved with the ShelXT 2014/5 (Sheldrick, 2014) solution program using dual methods and by using Olex2 1.5-alpha (Dolomanov et al., 2009) as the graphical interface. The model was refined with ShelXL 2016/6 (Sheldrick, 2015) using full matrix least squares minimisation on F2.

Crystal Data. $C_{12}H_9FO_2S$, M_r = 236.25, monoclinic, $P2_1/n$ (No. 14), a = 14.5997(2) Å, b = 7.95050(10) Å, c = 18.3482(2) Å, b = 95.9900(10)°, a = g = 90°, V = 2118.14(5) Å^3, T = 100(2) K, Z = 8, Z' = 2, m(Cu K$_a$) = 2.705, 44180 reflections measured, 4052 unique (R_{int} = 0.0381) which were used in all calculations. The final wR_2 was 0.1145 (all data) and R_1 was 0.0384 (I≥2 s(I)).

3',5-Dimethyl-[1,1'-biphenyl]-2-sulfonyl fluoride (3b):

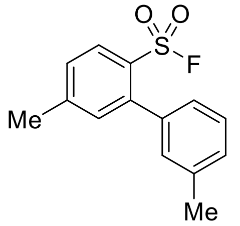

White solid. Obtained in 82% yield (108 mg, 0.41 mmol) from **1b** (122 mg, 0.5 mmol, 1.0 equiv) following **Procedure 14**.

See procedure 1 for the preparation of the starting material **1b**.

Reaction temperature: 50 °C.

Reaction vial: 10 mL CEM® microwave vial.

R_F: 0.69 (hexane/EtOAc, 99:1).

^{1}H NMR (400 MHz, DMSO-d$_6$): δ 8.09 (d, J = 8.3 Hz, 1H, Ar*H*), 7.56 (d, J = 8.3 Hz, 1H, Ar*H*), 7.40 (s, 1H, Ar*H*), 7.34 (t, J = 7.5 Hz, 1H, Ar*H*), 7.26 (d, J = 7.7 Hz, 1H, Ar*H*), 7.18 – 7.13 (m, 2H, Ar*H*), 2.47 (s, 3H, ArCH_3), 2.35 (s, 3H, ArCH_3).

^{13}C NMR (101 MHz, CDCl$_3$): δ 146.2 (C_{Ar}), 143.3 (C_{Ar}), 138.2 (C_{Ar}), 137.7 (C_{Ar}), 133.8 (d, J = 1.1 Hz, CH$_{Ar}$), 130.2 (d, J = 1.2 Hz, CH$_{Ar}$), 129.7 (d, J = 1.5 Hz, CH$_{Ar}$), 129.4 (d, J = 20.5 Hz, C_{Ar}), 129.3 (CH$_{Ar}$), 128.6 (CH$_{Ar}$), 127.9 (CH$_{Ar}$), 126.1 (d, J = 1.8 Hz, CH$_{Ar}$), 21.7 (ArCH$_3$), 21.5 (ArCH$_3$).

^{19}F NMR (376 MHz, CDCl$_3$): δ 68.08 (s, ArSO$_2$*F*).

HRMS (EI, m/z): Calculated for $[C_{14}H_{13}O_2FS]^+$: 264.0615; Found: 264.0612.

3,3'-Dimethyl-[1,1'-biphenyl]-2-sulfonyl fluoride (3c):

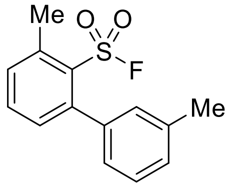

White solid. Obtained in 68% yield (90 mg, 0.34 mmol) from **1c** (122 mg, 0.5 mmol, 1.0 equiv) following **Procedure 14**.

See procedure 1 for the preparation of the starting material **1c**.

Reaction temperature: 50 °C.

Reaction vial: 10 mL CEM® microwave vial.

R_F: 0.69 (hexane/EtOAc, 98:2).

^{1}H NMR (400 MHz, $CDCl_3$) δ 7.54 (app t, J = 7.6 Hz, 1H, ArH), 7.41 (d, J = 7.6 Hz, 1H, ArH), 7.31 (app t, J = 7.4 Hz, 1H, ArH), 7.24 (app t, J = 7.4 Hz, 2H, ArH), 7.14 – 7.12 (m, 2H, ArH), 2.78 (d, J = 2.3 Hz, 3H, ArCH_3), 2.41 (s, 3H, ArCH_3).
^{13}C NMR (101 MHz, $CDCl_3$): δ 144.4 (C_{Ar}), 140.0 (C_{Ar}), 139.8 (C_{Ar}), 137.7 (C_{Ar}), 133.3 (CH_{Ar}), 132.3 (d, J = 21.3 Hz, C_{Ar}), 132.2 (CH_{Ar}), 131.0 (d, J = 1.5 Hz CH_{Ar}), 129.3 (CH_{Ar}), 128.9 (CH_{Ar}), 127.9 (CH_{Ar}), 125.9 (CH_{Ar}), 22.4 (d, J = 3.6 Hz, ArCH$_3$), 21.6 (ArCH$_3$).
^{19}F NMR (376 MHz, $CDCl_3$): δ 67.98 (s, ArSO$_2$$F$).
HRMS (EI, m/z): Calculated for $[C_{14}H_{13}O_2FS]^+$: 264.0615; Found: 264.0616.

4,4'-Dimethyl-[1,1'-biphenyl]-2-sulfonyl fluoride (3d):

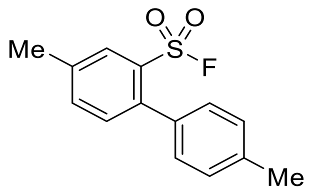

White solid. Obtained in 73% yield (96 mg, 0.37 mmol) from **1d** (122 mg, 0.5 mmol, 1.0 equiv) following **Procedure 14**.
See procedure 1 for the preparation of the starting material **1d**.
Reaction temperature: 25 °C.
Reaction vial: 10 mL CEM® microwave vial.
R_F: 0.69 (hexane/EtOAc, 98:2).
^{1}H NMR (400 MHz, $CDCl_3$): δ 7.99 (s, 1H, ArH), 7.55 (d, J = 7.8 Hz, 1H, ArH), 7.35 (d, J = 7.8 Hz, 1H, ArH), 7.28 – 7.24 (m, 4H, ArH), 2.52 (s, 3H, ArCH_3), 2.43 (s, 3H, ArCH_3).
^{13}C NMR (101 MHz, $CDCl_3$): δ 140.4 (C_{Ar}), 138.4 (C_{Ar}), 138.3 (C_{Ar}), 135.6 (CH_{Ar}), 135.2 (C_{Ar}), 133.2 (d, J = 1.4 Hz, CH_{Ar}), 132.1 (d, J = 21.3 Hz, C_{Ar}), 130.3 (d, J = 1.5 Hz, CH_{Ar}), 129.1 (d, J = 1.7 Hz, CH_{Ar}), 128.8 (CH_{Ar}), 21.5 (ArCH$_3$), 21.1 (ArCH$_3$).
^{19}F NMR (376 MHz, $CDCl_3$): δ 67.40 (s, ArSO$_2$$F$).
HRMS (EI, m/z): Calculated for $[C_{14}H_{13}O_2FS]^+$: 264.0615; Found: 264.0613.

[1,1':3',1'':3'',1'''-Quaterphenyl]-4'-sulfonyl fluoride (3e):

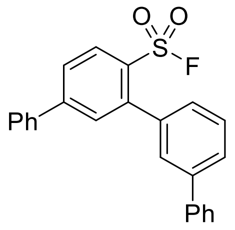

White oil. Obtained in 80% yield (155 mg, 0.40 mmol) from **1e** (184 mg, 0.5 mmol, 1.0 equiv) following **Procedure 14**.

See procedure 1 for the preparation of the starting material **1e**.

Reaction temperature: 25 °C.

Reaction vial: 10 mL CEM® microwave vial.

R_F: 0.69 (hexane/EtOAc, 49:1).

^{1}H NMR (400 MHz, $CDCl_3$) δ 8.26 (d, J = 8.4 Hz, 1H, ArH), 7.81 (app dt, J = 8.4, 1.6 Hz, 1H, ArH), 7.76 – 7.64 (m, 7H, ArH), 7.58 – 7.42 (m, 7H, ArH), 7.38 – 7.34 (m, 1H, ArH).

^{13}C NMR (101 MHz, $CDCl_3$): δ 1×CH$_{Ar}$ missing, 147.9 (C_{Ar}), 143.6 (C_{Ar}), 141.2 (C_{Ar}), 140.7 (C_{Ar}), 138.6 (C_{Ar}), 138.4 (C_{Ar}), 131.7 (CH$_{Ar}$), 131.0 (CH_{Ar}), 130.9 (d, J = 22.3 Hz, C_{Ar}), 129.4 (CH$_{Ar}$), 129.0 (CH$_{Ar}$), 128.7 (CH$_{Ar}$), 128.1 (d, J = 1.5 Hz, CH$_{Ar}$), 128.0 (d, J = 1.5 Hz, CH$_{Ar}$), 127.7 (CH$_{Ar}$), 127.6 (CH$_{Ar}$), 127.5 (CH$_{Ar}$), 127.4 (CH$_{Ar}$), 126.6 (CH$_{Ar}$).

^{19}F NMR (376 MHz, $CDCl_3$): δ 67.95 (s, ArSO_2F).

HRMS (EI, m/z): Calculated for $[C_{24}H_{17}O_2FS]^+$: 388.0928; Found: 388.0926.

4,4'''-Bis(trifluoromethyl)-[1,1':3',1'':3'',1'''-quaterphenyl]-4'-sulfonyl fluoride (3f):

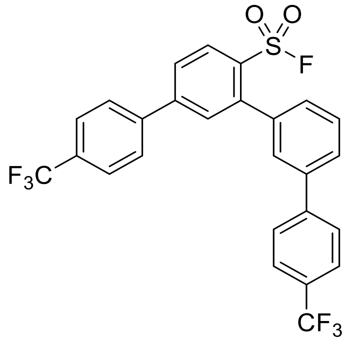

White solid. Obtained in 43% yield (58 mg, 0.11 mmol) from **1f** (126 mg, 0.25 mmol, 1.0 equiv) following **Procedure 14**.

See procedure 1 for the preparation of the starting material **1f**.

Reaction temperature: 50 °C.

Reaction vial: 10 mL CEM® microwave vial.

R_F: 0.69 (hexane/EtOAc, 99:1).

^{1}H NMR (400 MHz, $CDCl_3$): δ 8.31 (d, J = 8.4 Hz, 1H, ArH), 7.84 (d, J = 8.4 Hz, 1H, ArH), 7.77 – 7.67 (m, 11H, ArH), 7.59 (app t, J = 7.6 Hz, 1H, ArH), 7.47 (d, J = 7.7 Hz, 1H, ArH).

^{13}C NMR (101 MHz, $CDCl_3$): δ 146.4 (C_{Ar}), 144.1 (q, J = 1.2 Hz, C_{Ar}), 143.4 (C_{Ar}), 141.8 (q, J = 1.2 Hz, C_{Ar}), 139.8 (C_{Ar}), 138.5 (C_{Ar}), 131.9 (CH_{Ar}), 131.9 (d, J = 22.7 Hz, C_{Ar}), 131.4 (q, J = 32.8 Hz, C_{Ar}), 131.1 (CH_{Ar}), 129.9 (q, J = 32.5 Hz, C_{Ar}), 129.0 (CH_{Ar}), 128.8 (CH_{Ar}), 128.2 (CH_{Ar}), 128.0 (CH_{Ar}), 127.8 (CH_{Ar}), 127.7 (CH_{Ar}), 127.0 (CH_{Ar}), 126.3 (q, J = 3.8 Hz, CH_{Ar}), 126.0 (q, J = 3.7 Hz, CH_{Ar}), 124.4 (q, J = 272.2 Hz, CF_3), 124.0 (q, J = 272.3 Hz, CF_3).

^{19}F NMR (376 MHz, $CDCl_3$): δ 67.85 (s, $ArSO_2F$), –62.43 (s, $ArCF_3$), –62.68 (s, $ArCF_3$).

3',5-Dimethoxy-[1,1'-biphenyl]-2-sulfonyl fluoride (3g):

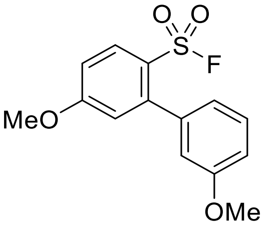

White oil. Obtained in 56% yield (83 mg, 0.28 mmol) from **1g** (138 mg, 0.5 mmol, 1.0 equiv) following **Procedure 14**.

See procedure 1 for the preparation of the starting material **1g**.

Reaction temperature: 50 °C.

Reaction vial: 10 mL CEM® microwave vial.

R_F: 0.69 (hexane/EtOAc, 19:1).

^{1}H NMR (400 MHz, $CDCl_3$) δ 8.10 (d, J = 9.0 Hz, 1H, ArH), 7.37 – 7.33 (m, 1H, ArH), 7.05 – 6.91 (m, 5H, ArH), 3.91 (s, 3H, $ArOCH_3$), 3.84 (s, 3H, $ArOCH_3$).

^{13}C NMR (101 MHz, $CDCl_3$): δ 164.3 (C_{Ar}), 159.1 (C_{Ar}), 145.5 (C_{Ar}), 139.4 (C_{Ar}), 132.8 (CH_{Ar}), 129.2 (CH_{Ar}), 123.7 (d, J = 23.1 Hz, C_{Ar}), 121.3 (d, J = 1.7 Hz, CH_{Ar}), 118.4 (CH_{Ar}), 114.7 (d, J = 1.8 Hz, CH_{Ar}), 114.3 (CH_{Ar}), 113.3 (CH_{Ar}), 56.1 ($ArOCH_3$), 55.4 ($ArOCH_3$).

^{19}F NMR (376 MHz, $CDCl_3$): δ 69.36 (s, $ArSO_2F$).

HRMS (EI, m/z): Calculated for $[C_{14}H_{13}O_4FS]^+$: 296.0513; Found: 296.0515.

4,4'-Dimethoxy-[1,1'-biphenyl]-2-sulfonyl fluoride (3h):

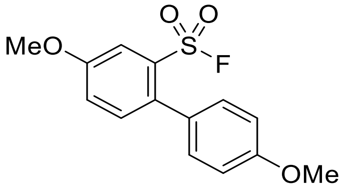

White solid. Obtained in 88% yield (130 mg, 0.44 mmol) from **1h** (138 mg, 0.5 mmol, 1.0 equiv) following **Procedure 14**.

See procedure 7 for the preparation of the starting material **1h**.

Reaction temperature: 25 °C.

Reaction vial: 10 mL CEM® microwave vial.

R_F: 0.69 (hexane/EtOAc, 98:2).

^{1}H NMR (400 MHz, DMSO-d$_6$): δ 7.61 (d, J = 2.1 Hz, 1H, ArH), 7.52 – 7.46 (m, 2H, ArH), 7.26 (d, J = 8.3 Hz, 2H, ArH), 7.02 – 6.98 (m, 2H, ArH), 3.92 (s, 3H, ArOCH_3), 3.81 (s, 3H, ArOCH_3).

^{13}C NMR (101 MHz, CDCl$_3$): δ 159.7 (C_{Ar}), 158.8 (C_{Ar}), 135.0 (C_{Ar}), 134.5 (d, J = 1.7 Hz, CH$_{Ar}$), 133.0 (d, J = 21.6 Hz, C_{Ar}), 130.5 (d, J = 1.7 Hz, CH$_{Ar}$), 130.0 (C_{Ar}), 121.0 (CH$_{Ar}$), 114.6 (CH$_{Ar}$), 113.5 (CH$_{Ar}$), 56.0 (ArOCH$_3$), 55.3 (ArOCH$_3$).

^{19}F NMR (376 MHz, CDCl$_3$): δ 66.67 (s, ArSO$_2$$F$).

HRMS (EI, m/z): Calculated for $[C_{14}H_{13}O_4FS]^+$: 296.0513; Found: 296.0513.

3,5-Di(1*H*-pyrazol-1-yl)-[1,1'-biphenyl]-2-sulfonyl fluoride (3i):

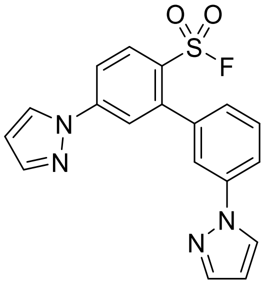

White solid. Obtained in 72% yield (66 mg, 0.18 mmol) from **1i** (87 mg, 0.25 mmol, 1.0 equiv) following **Procedure 14**.

See procedure 1 for the preparation of the starting material **1i**.

Reaction temperature: 50 °C.

Reaction vial: 10 mL CEM® microwave vial.

R_F: 0.69 (hexane/EtOAc, 99:1).

^{1}H NMR (400 MHz, $CDCl_3$): δ 8.26 (d, J = 8.8 Hz, 1H, ArH), 8.05 (d, J = 2.7 Hz, 1H, ArH), 7.98 – 7.94 (m, 2H, ArH), 7.89 (d, J = 2.3 Hz, 1H, ArH), 7.83 – 7.79 (m, 3H, ArH) 7.72 (d, J = 2.0 Hz, 1H, ArH), 7.57 – 7.52 (m, 1H, ArH), 7.34 (d, J = 7.6 Hz, 1H, ArH), 6.55 (app t, J = 2.3 Hz, 1H, ArH), 6.48 (app t, J = 2.1 Hz, 1H, ArH).

^{13}C NMR (101 MHz, $CDCl_3$): δ 144.4 (C_{Ar}), 144.2 (C_{Ar}), 143.2 (CH_{Ar}), 141.6 (CH_{Ar}), 140.1 (C_{Ar}), 138.7 (C_{Ar}), 132.4 (CH_{Ar}), 129.4 (CH_{Ar}), 129.0 (d, J = 23.5 Hz, C_{Ar}), 127.3 (CH_{Ar}), 127.0 (CH_{Ar}), 127.0 (CH_{Ar}), 122.3 (CH_{Ar}), 119.9 (CH_{Ar}), 119.6 (CH_{Ar}), 117.5 (CH_{Ar}), 109.7 (CH_{Ar}), 108.1 (CH_{Ar}).

^{19}F NMR (376 MHz, $CDCl_3$): δ 68.69 (s, $ArSO_2F$).

HRMS (EI, m/z): Calculated for $[C_{18}H_{14}O_2N_4FS]^+$: 369.0816; Found: 269.0815.

3,5-bis(trifluoromethoxy)-[1,1'-biphenyl]-2-sulfonyl fluoride (3j):

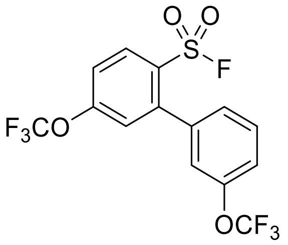

White solid. Obtained in 38% yield (38 mg, 0.10 mmol) from **1j** (96 mg, 0.25 mmol, 1.0 equiv) following **Procedure 14**.

See procedure 1 for the preparation of the starting material **1j**.

Reaction temperature: 50 °C.

Reaction vial: 10 mL CEM® microwave vial.

R_F: 0.69 (hexane/EtOAc, 99:1).

^{1}H NMR (400 MHz, $CDCl_3$) δ 8.26 (d, J = 8.9 Hz, 1H, ArH), 7.53 – 7.44 (m, 2H, ArH), 7.37 – 7.25 (m, 4H, ArH).

^{13}C NMR (101 MHz, $CDCl_3$): δ 153.6 (C_{Ar}), 149.0 (C_{Ar}), 144.1 (C_{Ar}), 138.3 (C_{Ar}), 132.9 (CH_{Ar}), 130.4 (d, J = 24.2 Hz, C_{Ar}), 130.0 (CH_{Ar}), 127.4 (CH_{Ar}), 124.2 (CH_{Ar}), 121.8 (CH_{Ar}), 121.7 (CH_{Ar}), 120.6 (q, J = 257.9 Hz, OCF_3), 120.3 (q, J = 261.2 Hz, OCF_3), 119.8 (CH_{Ar}).

^{19}F NMR (376 MHz, $CDCl_3$): δ 68.35 (s, $ArSO_2F$), –57.59 (s, $ArOCF_3$), –57.88 (s, $ArOCF_3$).

3',5-Difluoro-[1,1'-biphenyl]-2-sulfonyl fluoride (3k):

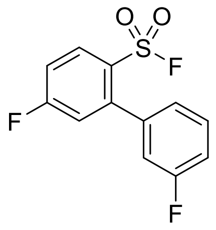

White solid. Obtained in 18% yield (25 mg, 0.09 mmol) from **1k** (126 mg, 0.5 mmol, 1.0 equiv) following **Procedure 14**.

An increase in yield was observed when substituting Ir(ppy) with [Ir-(dtbbpy)$(ppy)_2$]PF_6: White solid. Obtained in 44% yield (60 mg, 0.22 mmol) from **1k** (126 mg, 0.5 mmol, 1.0 equiv) and [Ir-(dtbbpy)$(ppy)_2$]PF_6 (5 mg, 1 mol %) following **Procedure 14**.

See procedure 1 for the preparation of the starting material **1k**.

Reaction temperature: 50 °C.

Reaction vial: 10 mL CEM® microwave vial.

R_F: 0.69 (hexane/EtOAc, 99:1).

^{1}H NMR (400 MHz, $CDCl_3$): δ 8.22 (dd, J = 8.9, 5.4 Hz, 1H, ArH), 7.43 (app td, J = 7.9, 5.8 Hz, 1H, ArH), 7.31 (dddd, J = 8.9, 7.6, 2.7, 1.1 Hz, 1H, ArH), 7.21 – 7.14 (m, 3H, ArH), 7.10 (d, J = 9.2 Hz, 1H, ArH).

^{13}C NMR (101 MHz, $CDCl_3$): δ 165.9 (d, J = 261.2 Hz, C_{Ar}), 162.3 (d, J = 248.0 Hz, C_{Ar}), 145.1 (dd, J = 9.7, 2.2 Hz, C_{Ar}), 138.7 (dd, J = 7.8, 1.5 Hz, C_{Ar}), 133.5 (dd, J = 10.1, 1.3 Hz, CH_{Ar}), 130.0 (d, J = 8.4 Hz, CH_{Ar}), 128.4 (dd, J = 24.0, 3.3 Hz, C_{Ar}), 124.7 (dd, J = 3.1, 1.6 Hz, CH_{Ar}), 120.4 (dd, J = 23.0, 1.4 Hz, CH_{Ar}), 116.3 (d, J = 20.9 Hz, CH_{Ar}), 116.2 (dd, J = 22.9, 1.7 Hz, CH_{Ar}),115.9 (d, J = 22.3 Hz, CH_{Ar}).

^{19}F {^{1}H} NMR (376 MHz, $CDCl_3$): δ 68.64 (s, ArSO_2F), –100.33 (s, ArF), –112.43 (s, ArF).

HRMS (EI, m/z): Calculated for $[C_{12}H_7O_2F_3S]^+$: 272.0013; Found: 272.0014.

5-Methoxy-[1,1'-biphenyl]-2-sulfonyl fluoride and 3'-methoxy-[1,1'-biphenyl]-2-sulfonyl fluoride (3j):

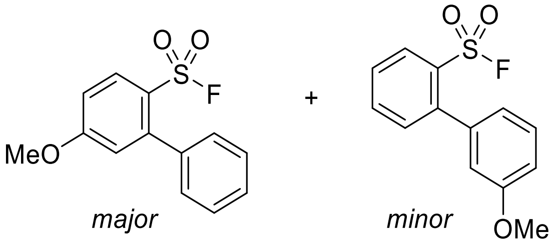

White solid. Obtained as a 5:1 mixture of isomers in 86% yield (115 mg, 0.43 mmol) from **1j** (123 mg, 0.5 mmol, 1.0 equiv) following **Procedure 14**.

See procedure 1 for the preparation of the starting material **1j**.

Reaction temperature: 50 °C.

Reaction vial: 10 mL CEM® microwave vial.

R_F: 0.69 (hexane/EtOAc, 99:1).

^{1}H NMR (400 MHz, $CDCl_3$) δ 8.18 (d, J = 8.0 Hz, 1H, ArH, *minor*), 8.11 (d, J = 9.0 Hz, 1H, ArH, *major*), 7.76 (td, J = 7.6, 1.4 Hz, 1H, ArH, *minor*), 7.60 (tt, J = 7.7, 1.4 Hz, 1H, ArH, *minor*), 7.50 – 7.34 (m, 5H, ArH, *major* & m, 2H, ArH, *minor*), 7.06 – 6.92 (m, 2H, ArH, *major* & m, 3H, ArH, *minor*), 3.91 (s, 3H, ArOCH_3, *major*), 3.84 (s, 3H, ArOCH_3, *minor*).

^{13}C NMR (101 MHz, $CDCl_3$) δ 164.3 (C_{Ar}, *major*), 159.2 (C_{Ar}, *minor*), 145.7 (C_{Ar}, *major*), 143.0 (C_{Ar}, *minor*), 139.3 (C_{Ar}, *minor*), 138.1 (C_{Ar}, *major*), 134.9 (CH_{Ar}, *minor*), 133.0 (CH_{Ar}, *minor*), 132.9 (CH_{Ar}, *major*), 132.4 (d, J = 22.0 Hz, C_{Ar}, *minor*), 130.1 (CH_{Ar}, *minor*), 129.20 (CH_{Ar}, *minor*), 128.9 (d, J = 1.8 Hz, CH_{Ar}, *major*), 128.7 (CH_{Ar}, *major*), 128.2 (CH_{Ar}, *minor*), 128.1 (CH_{Ar}, *major*), 123.7 (d, J = 23.0 Hz, C_{Ar}, *major*), 121.5 (d, J = 1.5 Hz, CH_{Ar}, *minor*), 118.5 (d, J = 1.3 Hz, CH_{Ar}, *major*), 114.9 (d, J = 1.8 Hz, CH_{Ar}, *minor*), 114.3 (CH_{Ar}, *minor*), 113.2 (CH_{Ar}, *major*), 56.1 (CH_3, *major*), 55.4 (CH_3, *minor*).

^{19}F NMR (376 MHz, $CDCl_3$) δ 69.41 (s, $ArSO_2F$), 67.25 (s, $ArSO_2F$).

HRMS (EI, m/z): Calculated for $[C_{13}H_{11}O_3FS]^+$: 266.0398; Found: 266.0407.

2-(Naphthalen-1-yl)benzenesulfonyl fluoride (3k):

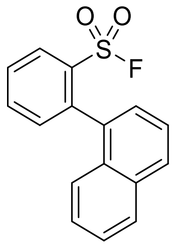

White solid. Obtained in 32% yield (46 mg, 0.16 mmol) from **1k** (133 mg, 0.5 mmol, 1.0 equiv) following **Procedure 14**.

See procedure 1 for the preparation of the starting material **1k**.

Reaction temperature: 50 °C.

Reaction vial: 10 mL CEM® microwave vial.

R_F: 0.69 (hexane/EtOAc, 99:1).

^{1}H NMR (400 MHz, $CDCl_3$) δ 8.28 (dd, J = 8.0, 1.3 Hz, 1H, ArH), 7.95 (d, J = 8.3 Hz, 1H, ArH), 7.92 (d, J = 8.2 Hz, 1H, ArH), 7.82 (app t, J = 7.6 Hz, 1H, ArH), 7.70 (app t, J = 7.8 Hz, 1H, ArH), 7.57 – 7.48 (m, 3H, ArH), 7.44 – 7.38 (m, 2H, ArH), 7.29 (d, J = 8.4 Hz, 1H, ArH).

^{13}C NMR (101 MHz, $CDCl_3$): δ 141.5 (C_{Ar}), 134.9 (C_{Ar}), 134.7 (C_{Ar}), 133.9 (C_{Ar}), 133.6 (d, J = 16.8 Hz, C_{Ar}), 133.4 (CH_{Ar}), 132.2 (CH_{Ar}), 130.3 (CH_{Ar}), 129.3 (CH_{Ar}), 128.6 (CH_{Ar}), 128.5 (CH_{Ar}), 127.5 (CH_{Ar}), 126.6 (CH_{Ar}), 126.2 (CH_{Ar}), 125.7 (CH_{Ar}), 124.8 (CH_{Ar}).

^{19}F NMR (376 MHz, $CDCl_3$): δ 65.80 (s, $ArSO_2F$).

HRMS (EI, m/z): Calculated for $[C_{16}H_{11}O_2FS]^+$: 286.0458; Found: 286.0461.

X-Ray Crystallography:

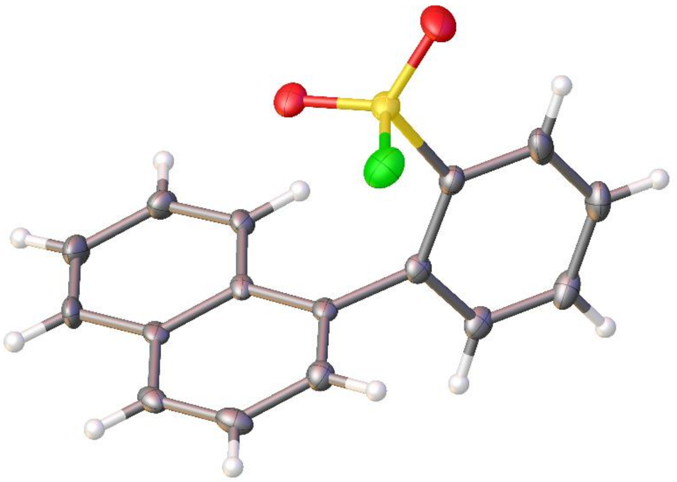

Experimental. Single clear colourless prism-shaped crystals of **3k** recrystallised from a mixture of DCM and hexane by slow evaporation. A suitable crystal with dimensions

0.37 × 0.11 × 0.06 mm^3 was selected and mounted on a MITIGEN holder with silicon oil on a ROD, Synergy Custom system, HyPix diffractometer. The crystal was kept at a steady T = 100(2) K during data collection. The structure was solved with the ShelXT 2014/5 (Sheldrick, 2014) solution program using dual methods and by using Olex2 1.5-alpha (Dolomanov et al., 2009) as the graphical interface. The model was refined with ShelXL 2016/6 (Sheldrick, 2015) using full matrix least squares minimisation on $\boldsymbol{F^2}$.

Crystal Data. $C_{16}H_{11}FO_2S$, M_r = 286.31, orthorhombic, *Pbcn* (No. 60), a = 20.5177(10) Å, b = 15.8556(8) Å, c = 8.0579(3) Å, $a = b = g = 90^{\circ}$, V = 2621.4(2) Å^3, T = 100(2) K, Z = 8, Z' = 1, m(Mo K_a) = 0.256, 28432 reflections measured, 3322 unique (R_{int} = 0.0620) which were used in all calculations. The final wR_2 was 0.3682 (all data) and R_1 was 0.1184 (I≥2 s(I)).

15) Deuterium labeling studies with CD_3CN (Procedure 15)

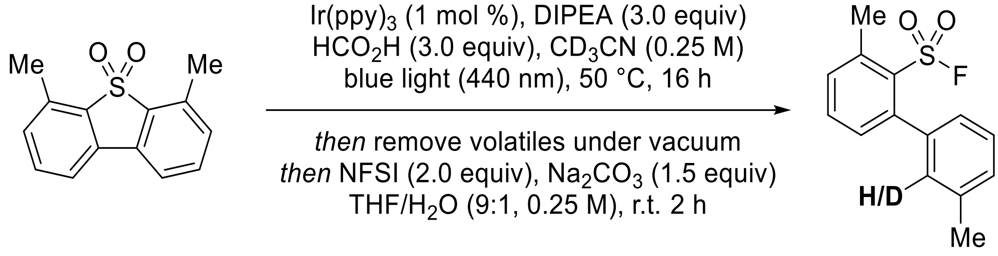

1c

3c 70%
H/D = 100:0

A 10-mL vial was charged with $Ir(ppy)_3$ (3 mg, 0.005 mmol, 1.0 mol %) and 4,6-dimethyldibenzo[*b,d*]thiophene 5,5-dioxide (122 mg, 0.50 mmol, 1.0 equiv). The vial was sealed using a septum and the mixture flushed with nitrogen for 15 minutes. Then CD_3CN (2.0 mL, 0.25 M), DIPEA (260 µL, 1.5 mmol, 3.0 equiv) and HCO_2H (57 µL, 1.5 mmol, 3.0 equiv) were added (Note: white fumes are produced upon addition of HCO_2H to the mixture containing DIPEA). The mixture was then irradiated with blue light at 50 °C for 16 h. The irradiation was stopped, and the volatile components were removed under vacuum using a rotary evaporator followed by high vacuum for 1 h. The resulting mixture was reconstituted in THF (1.8 mL) and H_2O (0.2 mL) (THF/H_2O 9:1, Total = 2.0 mL, 0.25 M) and Na_2CO_3 (80 mg, 0.75 mmol, 1.5 equiv) and NFSI (315 mg, 1.0 mmol, 2.0 equiv) were added (Note: the reaction becomes hot upon addition of NFSI, so we recommend this reagent is added portion wise when performing on larger scales). The mixture was stirred at room temperature for 2 h then quenched by addition of H_2O. The mixture was extracted with CH_2Cl_2, dried over Na_2SO_4, filtered and concentrated under vacuum. The crude mixture was purified by silica gel column chromatography to yield the desired sulfonyl fluoride product **3c** as a white solid in 70% yield (93 mg, 0.35 mmol). The deuterium incorporation was determined by quantitative ^1H NMR analysis. No significant deuterium incorporation was observed.

16) Deuterium labeling studies using DCO_2D (Procedure 16)

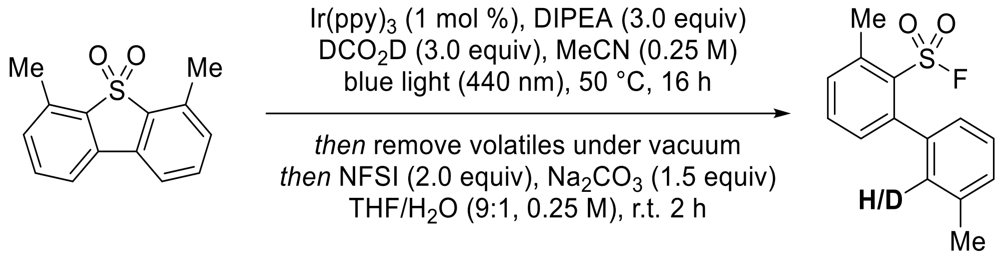

1c

3c 76%
H/D = 88:12

A 10-mL vial was charged with Ir(ppy)3 (3 mg, 0.005 mmol, 1.0 mol %) and 4,6-dimethyldibenzo[*b,d*]thiophene 5,5-dioxide (122 mg, 0.50 mmol, 1.0 equiv). The vial was sealed using a septum and the mixture flushed with nitrogen for 15 minutes. Then MeCN (2.0 mL, 0.25 M), DIPEA (260 µL, 1.5 mmol, 3.0 equiv), DCO_2D (60 µL, 1.5 mmol, 3.0 equiv, 95 wt. % in H_2O) were added (Note: white fumes are produced upon addition of DCO_2D to the mixture containing DIPEA). The mixture was then irradiated with blue light at room temperature for the given time (16 h). The irradiation was stopped, and the volatile components were removed under vacuum using a rotary evaporator followed by high vacuum for 1 h. The resulting mixture was reconstituted in THF (1.8 mL) and H_2O (0.2 mL) (THF/H_2O 9:1, Total = 2.0 mL, 0.25 M) and Na_2CO_3 (80 mg, 0.75 mmol, 1.5 equiv) and NFSI (315 mg, 1.0 mmol, 2.0 equiv) were added (Note: the reaction becomes hot upon addition of NFSI, so we recommend this reagent is added portion wise when performing on larger scales). The mixture was stirred at room temperature for 2 h then quenched by addition of H_2O. The mixture was extracted with CH_2Cl_2, dried over Na_2SO_4, filtered and concentrated under vacuum. The crude mixture was purified by silica gel column chromatography to yield the desired sulfonyl fluoride product **3c** as a white solid in 70% yield (93 mg, 0.35 mmol). The deuterium incorporation was determined by quantitative ^1H NMR analysis. A small difference in the integrals suggested a 12% incorporation of deuterium for this compound.

17) Preparation of methyl sulfone (Procedure 17)

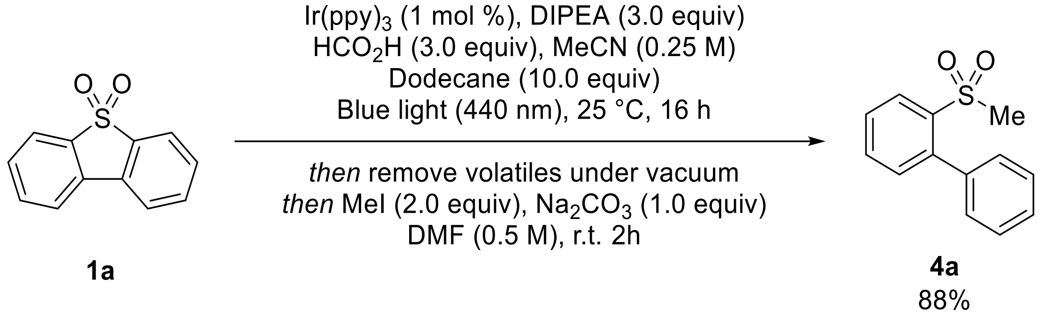

A 10-mL vial was charged with Ir(ppy)$_3$ (3 mg, 0.005 mmol, 1.0 mol %) and dibenzo[*b,d*]thiophene 5,5-dioxide (108 mg, 0.50 mmol, 1.0 equiv). The vial was sealed using a septum and the mixture flushed with nitrogen for 15 minutes. Then MeCN (2.0 mL, 0.25 M), DIPEA (260 µL, 1.5 mmol, 3.0 equiv), HCO_2H (57 µL, 1.5 mmol, 3.0 equiv) were added (Note: white fumes are produced upon addition of HCO_2H to the mixture containing DIPEA). The mixture was then irradiated with blue light at 25 °C for 16 h. The irradiation was stopped, and the volatile components were removed under vacuum using a rotary evaporator followed by high vacuum for 1 h. The resulting mixture was reconstituted in DMF (1.0 mL) and Na_2CO_3 (53 mg, 0.5 mmol, 1.0 equiv) and MeI (142 mg, 1.0 mmol, 2.0 equiv) were added. The mixture was stirred at room temperature for 2 h then quenched by addition of H_2O. The mixture was extracted with CH_2Cl_2, dried over Na_2SO_4, filtered and concentrated under vacuum. The product was purified by column chromatography on silica gel (Hexane/EtOAc gradient = 9.5:0.5 to 8.5:1.5), and compound **4a** was obtained as a white powder (102 mg, 0.44 mmol, 88%).

^{1}H NMR (400 MHz, $CDCl_3$): δ 8.24 (dd, J = 8.0, 1.4 Hz, 1H, Ar*H*), 7.65 (td, J = 7.5, 1.5 Hz, 1H, Ar*H*), 7.56 (td, J = 7.7, 1.4 Hz, 1H, Ar*H*), 7.51 – 7.40 (m, 5H, Ar*H*), 7.37 (dd, J = 7.5, 1.4 Hz, 1H, Ar*H*), 2.62 (s, 3H, $ArSO_2CH_3$).

^{13}C NMR (101 MHz, $CDCl_3$): δ 141.5 (C_{Ar}), 139.4 (C_{Ar}), 138.6 (C_{Ar}), 133.2 (CH_{Ar}), 132.8 (CH_{Ar}), 130.2 (CH_{Ar}), 128.6 (CH_{Ar}), 128.5 (CH_{Ar}), 128.1 (CH_{Ar}), 128.1 (CH_{Ar}) 43.5 (SO_2CH_3). All the resonances in the ^{1}H and ^{13}C NMR spectra were consistent with the reported values.[24]

18) Preparation of biaryl (Procedure 18)

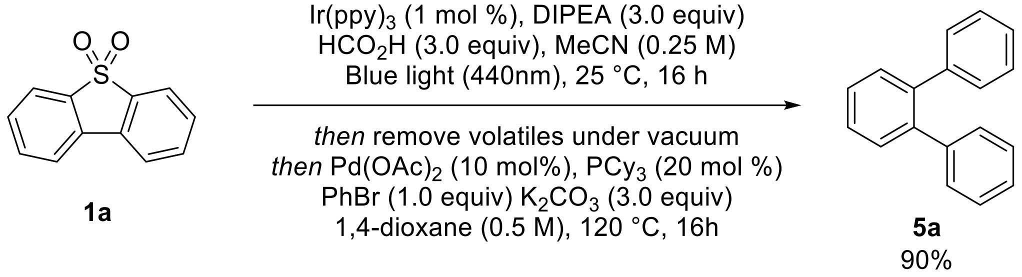

A 10-mL vial was charged with $Ir(ppy)_3$ (3 mg, 0.005 mmol, 1.0 mol %) and dibenzo[*b,d*]thiophene 5,5-dioxide (108 mg, 0.50 mmol, 1.0 equiv). The vial was sealed using a septum and the mixture flushed with nitrogen for 15 minutes. Then MeCN (2.0 mL, 0.25 M), DIPEA (260 µL, 1.5 mmol, 3.0 equiv) and HCO_2H (57 µL, 1.5 mmol, 3.0 equiv) were added (Note: white fumes are produced upon addition of HCO_2H to the mixture containing DIPEA). The mixture was then irradiated with blue light at 25 °C for 16 h. The irradiation was stopped, and the volatile components were removed under vacuum using a rotary evaporator followed by high vacuum for 1 h. The resulting mixture was reconstituted in 1,4-dioxane (1.0 mL) and K_2CO_3 (207 mg, 1.5 mmol, 3.0 equiv), PhBr (79 mg, 0.5 mmol, 1.0 equiv), $Pd(OAc)_2$ (11 mg, 0.05 mmol, 10.0 mol%) and PCy_3 (28 mg, 0.1 mmol, 20 mol %) were added. The mixture was stirred at 120 °C for 16 h then quenched by addition of H_2O. The mixture was extracted with CH_2Cl_2, dried over Na_2SO_4, filtered and concentrated under vacuum. Then the crude product was purified by column chromatography on silica gel (Hexane/EtOAc gradient = 9.5:0.5 to 8.5:1.5) to provide compound **5a** as a yellow oil (104 mg, 0.45 mmol, 90%).

^{1}H NMR (400 MHz, $CDCl_3$): δ 7.46 – 7.40 (m, 4H, Ar*H*), 7.24 – 7.18 (m, 6H, Ar*H*), 7.17 – 7.12 (m, 4H, Ar*H*).

^{13}C NMR (101 MHz, $CDCl_3$): δ 141.7 (C_{Ar}), 140.7 (C_{Ar}), 130.7 (CH_{Ar}), 130.0 (CH_{Ar}), 128.0 (CH_{Ar}), 127.6 (CH_{Ar}), 126.6 (CH_{Ar}).

All the resonances in the ^{1}H and ^{13}C NMR spectra were consistent with the reported values.[25]

19) Tolerance of the reaction to oil (Procedure 19)

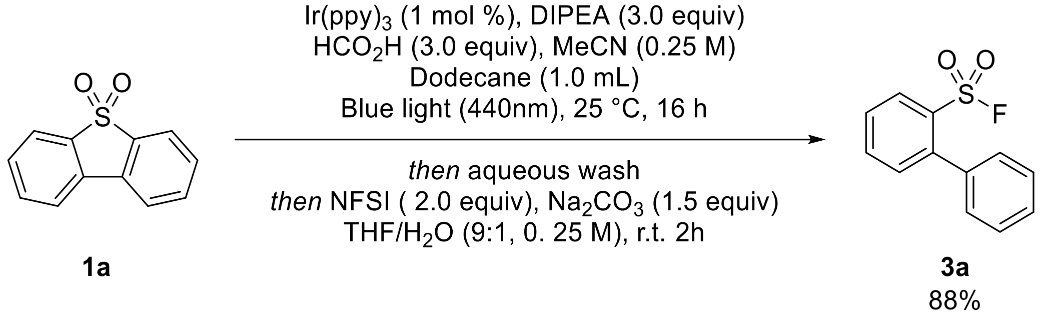

A 10-mL vial was charged with Ir(ppy)$_3$ (3 mg, 0.005 mmol, 1.0 mol %) and dibenzo[*b,d*]thiophene 5,5-dioxide (108 mg, 0.50 mmol, 1.0 equiv). The vial was sealed using a septum and the mixture flushed with nitrogen for 15 minutes. Then MeCN (2.0 mL, 0.25 M), DIPEA (260 µL, 1.5 mmol, 3.0 equiv), dodecane (1.0 mL, 4.4 mmol, 8.8 equiv) and HCO_2H (57 µL, 1.5 mmol, 3.0 equiv) were added (Note: white fumes are produced upon addition of HCO_2H to the mixture containing DIPEA). The mixture was then irradiated with blue light at 25 °C for 16 h. The irradiation was stopped, and the mixture transferred to a separating vial using water (~10 mL) and CH_2Cl_2 (~20 mL). The aqueous layer was washed with CH_2Cl_2 (10 mL × 3) and the organic phases discarded. The aqueous phase was concentrated under vacuum. The resulting mixture was reconstituted in THF (1.8 mL) and H_2O (0.2 mL) (THF/H_2O 9:1, Total = 2.0 mL, 0.25 M) and Na_2CO_3 (80 mg, 0.75 mmol, 1.5 equiv) and NFSI (315 mg, 1.0 mmol, 2.0 equiv) were added (Note: the reaction becomes hot upon addition of NFSI, so we recommend this reagent is added portionwise when performing on larger scales). The mixture was stirred at room temperature for 2 h then quenched by addition of H_2O. The mixture was extracted with CH_2Cl_2, dried over Na_2SO_4, filtered and concentrated under vacuum. The product **3a** was observed in 88% ^{1}H NMR yield using 1,1,2,2-tetrachloroethane as an internal standard.

20) Solubility tests

General procedure: For dibenzothiophene dioxide **1a**, 0.5 mmol of material was weighed into a 20 mL vial. MeCN was then added in 1 mL portions until the compound was fully dissolved. The solution was stirred for 15 minutes in between portions. 12 mL of MeCN was required to dissolve 0.5 mmol of **1a**, corresponding to a solubility of 18 mg/mL in MeCN.

All other substrates were much less soluble. For other substrates (**1b**, **1e**, **1h**, **1i**), 0.5 mmol of material was weighed into a 20 mL vial and 9.0 mL of MeCN was added. The reaction was allowed to stir at room temperature for 30 minutes. The mixture was then filtered into a pre-weighed 20 mL vial by passing through a Pasteur pipette plugged with a small piece of cotton. The MeCN was removed under vacuum and the vial reweighed. The mass recorded provided an estimate for the solubility of these compounds in MeCN.

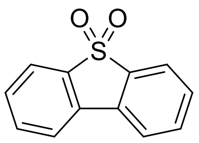

1a

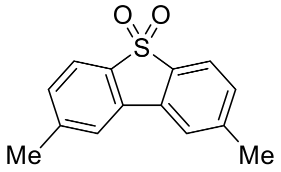

1b

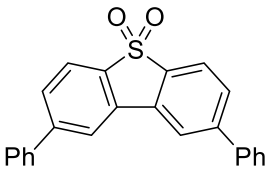

1e

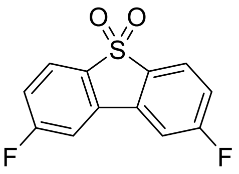

1h

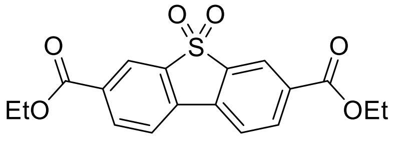

1i

Substrates	MW (g / mol)	Solvent	Solubility
**1a**	216.25	MeCN	18 mg / mL
**1b**	244.31	MeCN	2 mg / mL
**1e**	368.45	MeCN	5 mg / mL
**1h**	252.23	MeCN	5 mg / mL
**1i**	360.38	MeCN	<1 mg / mL

21) Cyclic voltammograms

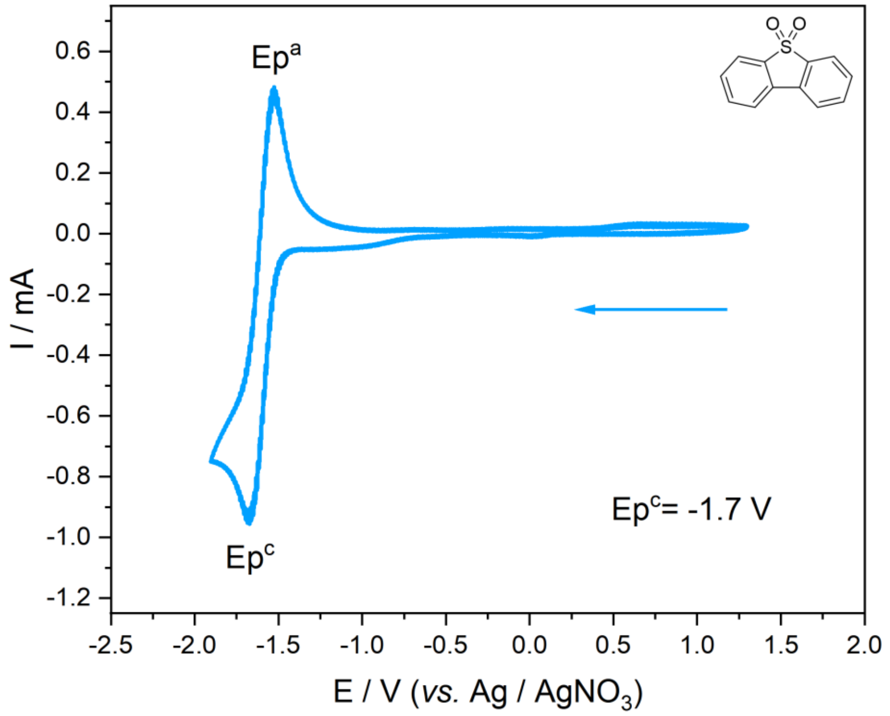

Cyclic voltammogram for dibenzothiophene dioxide **1a** [0.005 M] in [0.1 M] *n*-Bu_4NPF_6 in CH_3CN. Sweep rate: 0.1 V/s. Glassy carbon working electrode, Ag/$AgNO_3$ reference electrode, Pt wire auxiliary electrode. Reversible reduction, Ep^C = E(**1a**/**1a**$^{\bullet-}$) = –1.7 V *vs.* Ag/$AgNO_3$ in MeCN, where Ep^C refers to the cathodic peak potential, while the E value describes the electrochemical properties of **1a**.

The Ep^C values were converted from the Ag/$AgNO_3$ reference electrode to the SCE reference electrode using the reported conversion value of +0.298 V.[26] Using this conversion value, Ep^C = E(**1a**/**1a**$^{\bullet-}$) = –1.4 V *vs.* SCE in MeCN.

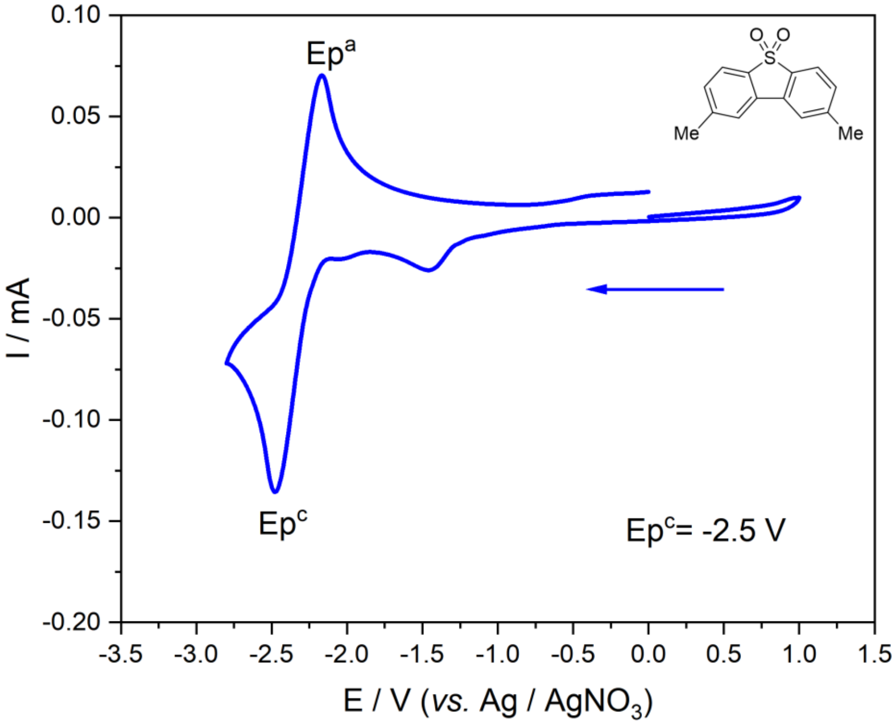

Cyclic voltammogram for dibenzothiophene dioxide **1b** [0.005 M] in [0.1 M] *n*-Bu_4NPF_6 in CH_3CN. Sweep rate: 0.1 V/s. Glassy carbon working electrode, Ag/$AgNO_3$ reference electrode, Pt wire auxiliary electrode. Reversible reduction, Ep^C = E(**1b**/**1b**$^{•−}$) = –2.5 V *vs.* Ag/$AgNO_3$ in MeCN, where Ep^C refers to the cathodic peak potential, while the E value describes the electrochemical properties of **1b**.

The Ep^C values were converted from the Ag/$AgNO_3$ reference electrode to the SCE reference electrode using the reported conversion value of +0.298 V.[26] Using this conversion value, Ep^C = E(**1b**/**1b**$^{•−}$) = –2.2 V *vs.* SCE in MeCN.

22) 3D-Printed photoreactor

The reactor was designed in Solidworks (Dassault software). For ease of printing, it was printed in three parts: the main reactor itself (Fig. S1.A) and two alignment supports (Fig. S1.B) that ensured the reactor sat centrally on the stirrer plate. The parts were glued together after printing, with pins on the supports and corresponding recesses in the reactor assisting positioning. Printing these as separate files maximised contact with the buildplate and minimised printing errors due to warping. 3D files for all parts are available as part of the supplementary information.

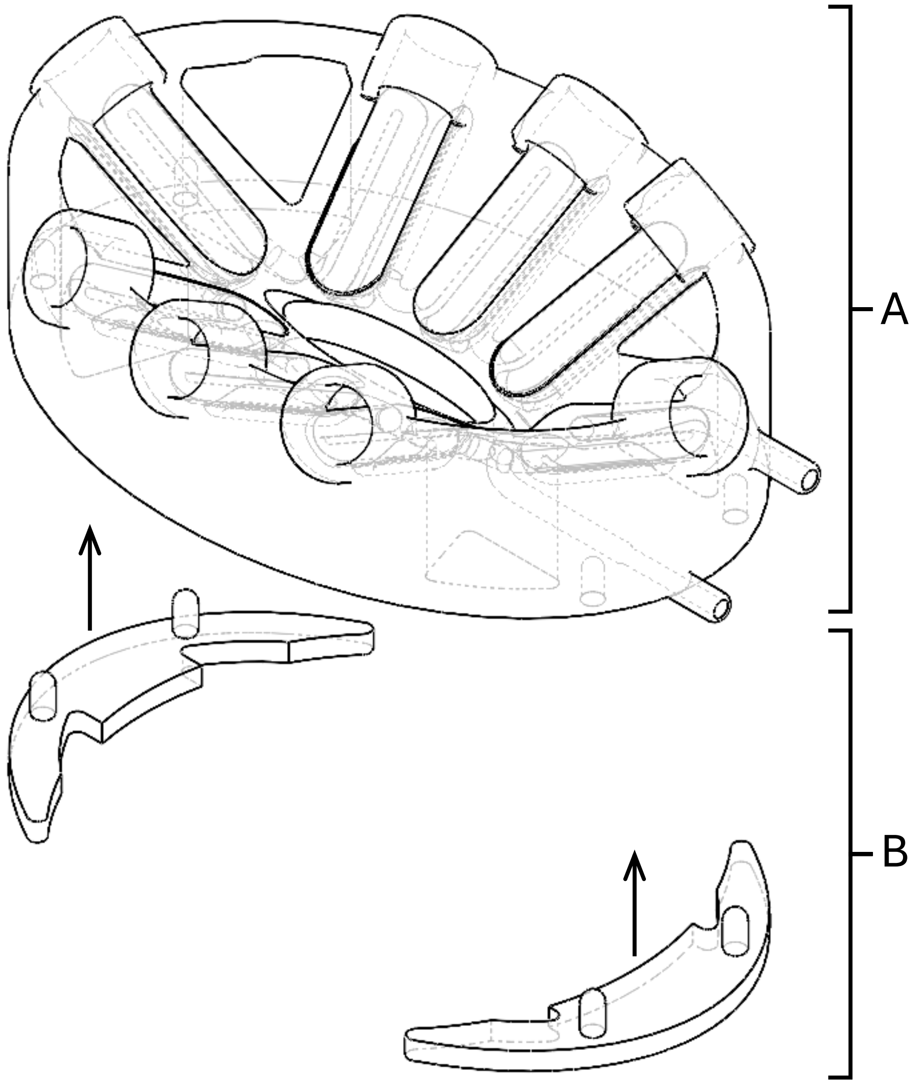

Figure S1: Computer aided drawing of the printed parts showing how they fitted together. A) The main reactor part. B) Supports to ensure alignment on stirrer plate.

The design files were exported as .stl files and sliced using Bambu Studio software ready for printing on a BambuLab X1-Carbon printer with a 1.75 mm nozzle, a BambuLab "Engineering" buildplate, and using BambuLab transparent polybarbonate filament. Parts were printed using default settings, with the following exceptions:

Support was enabled on the build plate only, at a threshold angle of 25°, and without small overhangs. The layer height was set to 0.06 mm, and a flow ratio of 1.1 (i.e. 110 % material flow) for the reactor - ensuring there was no leakage from the heating/cooling channels.[27] The supports (Fig. S1, part B) were printed with a flow ratio of 1.0. For buildplate adhesion, a 10 mm brim was used with an initial layer height of 0.28 mm. Infill used a lighting sparse pattern with 15 % infill density and three wall loops. Auxiliary and part fans on the printer were switched off to slow cooling, which could cause the print to warp.

With these settings, the final reactor (part A in Fig. S1 below) took 12 h to print and used 120 g of PC filament (equivalent to £4.44 of raw material cost).

The photoreactor fits on standard sized stirrer plates (e.g. IKA or Radleys stirrer plates) and holds standard vials (e.g. CEM 10 mL pressure vessels and Fisherbrand™ Test Tubes ⌀ = 16 mm). Using standard tubing, the input and output ports of the 3D-printed photoreactor were connected to a Julabo F12 circulator. The Kessil PR160L lamp was placed ~10 cm above the photoreactor.

To assess the temperatures that would be experienced within the vials, a Fisherbrand™ Test Tube (height = 100 mm, ⌀ = 16 mm) was filled with 4 mL of silicone oil and a thermometer was placed inside, from which the temperature was determined. To obtain a temperature of 25 °C within the vials, the Julabo F12 circulator was set to 15 °C (Note: the Kessil Lamps give off heat, therefore, the temperature of the circulator is set below the desired temperature in this case). To obtain a temperature of 50 °C within the vials, the Julabo F12 circulator was set to 50 °C.

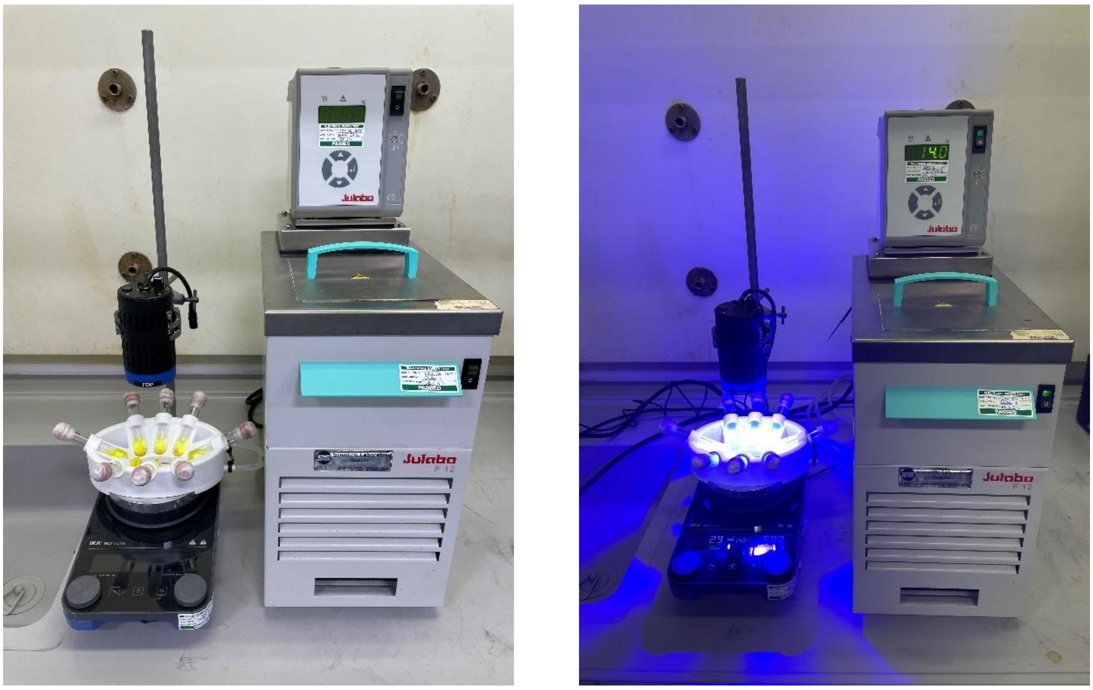

Figure S2: Photoredox reaction setup before (A) and during (B) irradiation.

23) Reproducibility tests

To ensure that significant differences in yield were not observed depending on the position of the vial in the photoreactor, 8 identical reactions were performed in tandem.

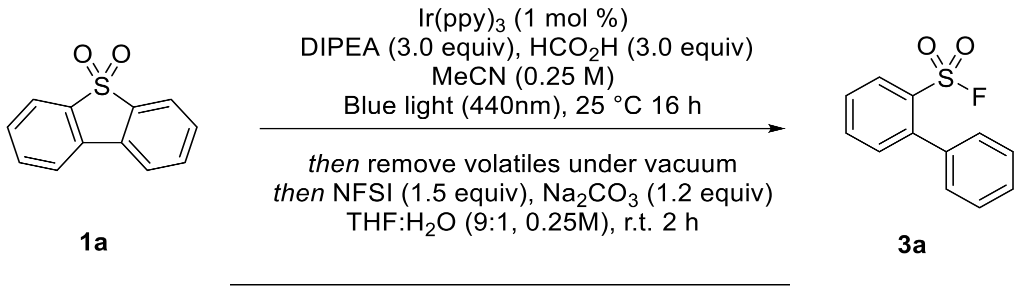

1a **3a**

Entry	NMR Yield (%[a])
A	95
B	89
C	90
D	88
E	92
F	88
G	89
H	85

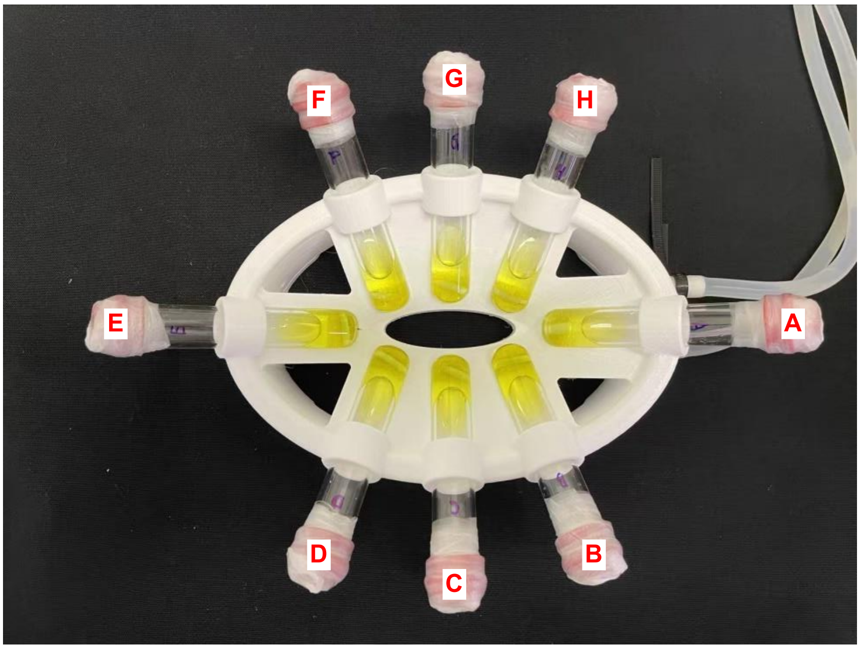

Figure S3: Parallel reaction in photoreactor.

24) Computational studies

All geometry optimizations and frequency calculations for reported structures were performed using the B3LYP functional[28] with the 6-31G+(d) basis set using the Gaussian 16 (G16) program.[29] Dispersion interactions were included using Grimme's DFT-D3 correction.[30] The SMD solvent effects were incorporated into all calculations with acetonitrile as the solvent.[31] This level is referred to as SMD(MeCN)/B3LYP-D3/6-31+G(d). All stationary points have been verified, through vibrational analysis, to be minima (zero imaginary frequencies) or transition structures (one imaginary frequency). The character of the normal mode associated with the imaginary frequency has been analyzed to ensure it resembles the reaction under consideration. Energy changes were shown by the use of Gibbs free energies (T = 298.15 K and P = 1 atm). Potential energies were refined by means of single point calculations using the M06-2X functional with the 6-311++G(d,p) basis set.[32] This level is denoted SMD(MeCN)/M06-2X/6-311++G(d,p)//SMD(MeCN)/B3LYP-D3/6-31+G(d).

Computational Details

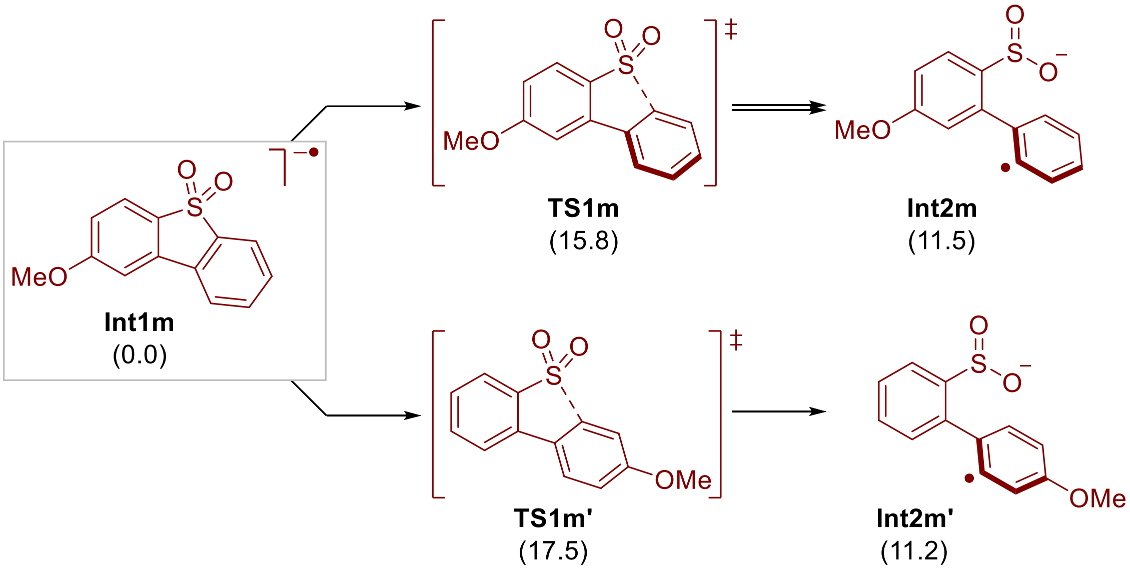

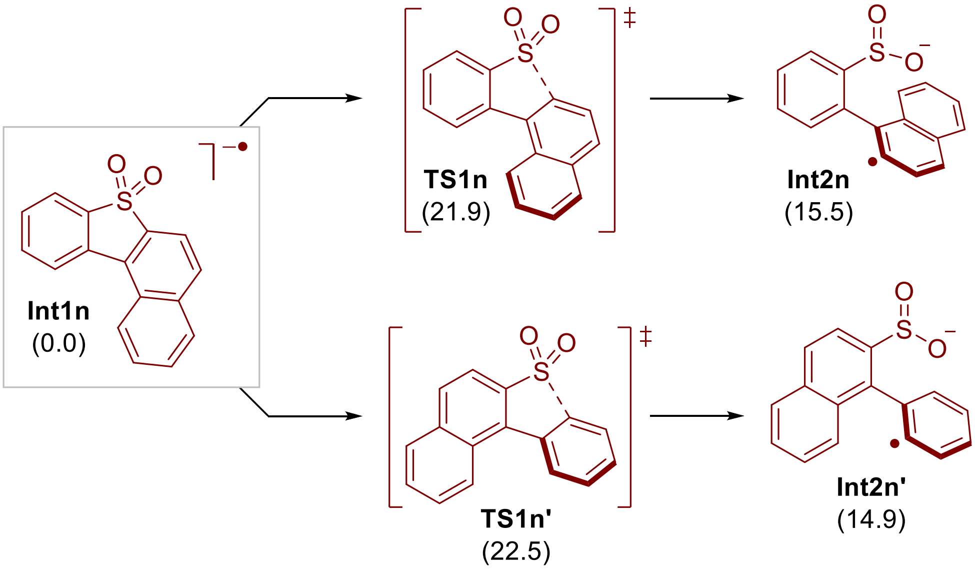

Scheme S1: Potential energy diagram for the C–S bond cleavage of unsymmetrical dibenzothiophene dioxides computed at the SMD(MeCN)/M06-2X/6-311++G(d,p)//SMD(MeCN)/B3LYP-D3/6-31+G(d) level of theory. Energies ($\Delta G^{\ddagger}$ [kcal mol^{-1}]) are provided in the insert.

Table S1: Energies (electronic energies (E), enthalpies (H) and Gibbs free energies (G) in Hartrees), spin contamination ($<S^2>$) and imaginary frequencies (in cm^{-1}) of all stationary points computed at SMD(MeCN)/B3LYP-D3/6-31+G(d) level of theory are provided. Electronic energies (E^{high} in Hartrees) at SMD(MeCN)/M06-2X/6-311++G(d,p)//SMD(MeCN)/B3LYP-D3/6-31+G(d) level of theory are also provided.

structure	*E*	*H*	*G*	E^{high}	$<S^2>$	*Imag. Freq.*
**Int1j**	-1125.356067	-1125.142720	-1125.200115	-1125.195438	0.759	-
**TS1j**	-1125.336717	-1125.124260	-1125.180807	-1125.170139	0.7587	187.9*i*
**TS1j'**	-1125.334494	-1125.122020	-1125.178311	-1125.167806	0.7585	201.4*i*
**Int2j**	-1125.341200	-1125.127377	-1125.187760	-1125.17509	0.767	-
**Int2j'**	-1125.341625	-1125.127845	-1125.187790	-1125.175068	0.7666	-
**Int1k**	-1164.488207	-1164.259950	-1164.317531	-1164.308887	0.762	-
**TS1k**	-1164.458977	-1164.231869	-1164.288514	-1164.273747	0.7611	83.2*i*
**TS1k'**	-1164.458070	-1164.231014	-1164.287344	-1164.273064	0.7599	126.7*i*
**Int2k**	-1164.462395	-1164.234508	-1164.294932	-1164.280957	0.7636	-
**Int2k'**	-1164.46138	-1164.233627	-1164.296046	-1164.279723	0.7621	-

Cartesian Coordinates

Int1m

S	-1.24874700	-1.56921900	-0.00017600
C	0.35574200	0.53972900	0.00033800
C	0.38404900	-0.89706600	0.00057700
O	-1.55091700	-2.35715200	-1.24581100
C	-3.76386100	1.64632400	0.00005700
C	-2.78723900	2.69054400	-0.00009400
C	-1.42902500	2.40567500	-0.00001600
C	-0.97506900	1.06374700	0.00023200
C	-1.98391100	0.02882800	0.00037500
C	-3.35396600	0.31567100	0.00022900
C	1.57712700	-1.61516700	0.00068300
H	-4.82281200	1.89096200	0.00003800
H	-3.11667300	3.72725100	-0.00029800
H	-0.70233500	3.21609300	-0.00015400
C	1.59961200	1.21389200	0.00015300
H	-4.08595600	-0.48988100	0.00029300
C	2.79013300	0.49238500	0.00019400
C	2.79437700	-0.93275700	0.00056400
H	3.72153300	-1.49257700	0.00086600
H	1.56973600	-2.70323600	0.00090000
H	1.64208600	2.30019200	-0.00010700

O	3.93936000	1.24323200	-0.00015100
O	-1.55184000	-2.35860200	1.24426400
C	5.20335200	0.57490500	-0.00050800
H	5.32969700	-0.04424600	0.89671300
H	5.95496300	1.36757400	-0.00103800
H	5.32895400	-0.04472900	-0.89750600

TS1m

S	1.24750200	1.72381500	-0.10260800
C	-0.30650600	-0.51932900	0.04330400
C	-0.35272200	0.89324200	0.02844900
O	1.01560000	2.77325400	-1.22320500
C	3.76553200	-1.78315200	-0.00621200
C	2.77220700	-2.76832200	-0.18764400
C	1.41982200	-2.41451600	-0.17988700
C	1.05066800	-1.07596500	0.03759500
C	2.05349800	-0.10031500	0.23623400
C	3.40944400	-0.44361800	0.17561500
C	-1.56969400	1.56955200	0.00745700
H	4.81561200	-2.07046500	-0.00274800
H	3.05962700	-3.80508600	-0.34503400
H	0.65868300	-3.17584800	-0.34137800
C	-1.50895500	-1.23563400	0.02887700
H	4.18057000	0.31762100	0.29419300

C	-2.73405000	-0.55534800	0.03173800
C	-2.77003500	0.85392500	0.02711900
H	-3.71102400	1.39165200	0.03027500
H	-1.59027200	2.65592700	-0.03140100
H	-1.51364500	-2.32218300	0.03075200
O	-3.84803700	-1.34749100	0.03946500
O	1.58246900	2.44881600	1.19782800
C	-5.13606900	-0.72263900	0.04797700
H	-5.85938500	-1.54062700	0.05569300
H	-5.28667100	-0.11033100	-0.84995100
H	-5.27263500	-0.10562100	0.94488600

TS1m'

S	-1.42425400	-1.60644000	0.13416000
C	-0.87420800	1.05939000	-0.08541900
C	-1.93053900	0.12302300	-0.02132100
O	-2.33330700	-2.14148000	1.27830400
C	2.84401700	-1.02962000	-0.11508000
C	2.87800500	0.37470500	0.02178700
C	1.68827400	1.11333300	0.04076000
C	0.45984200	0.45436800	-0.10833800
C	0.42767100	-0.94970400	-0.26049300
C	1.61156600	-1.68661400	-0.22352000
C	-3.26674900	0.52869800	0.01749700

H	3.76108900	-1.60880300	-0.13964700
O	4.03344400	1.10645200	0.15365700
H	1.74220600	2.19172300	0.17152900
C	-1.19083700	2.42917200	-0.09086100
H	1.59358600	-2.77349200	-0.30153200
C	-2.52464500	2.83919300	-0.07398300
C	-3.56495000	1.89174400	-0.02841500
H	-4.60100100	2.22142400	-0.02024800
H	-4.05932200	-0.21210500	0.08980800
H	-0.39476600	3.16916800	-0.12463000
H	-2.76146100	3.90018800	-0.09724800
O	-1.74648500	-2.37915400	-1.14313400
C	5.28236500	0.41264000	0.16779600
H	5.33842500	-0.29315200	1.00699600
H	6.04734200	1.18270000	0.29024500
H	5.45388300	-0.12313700	-0.77490800

Int2m

S	1.04247200	2.14661600	-0.04804000
C	-0.17970000	-0.42522000	-0.05372900
C	-0.38761900	0.97245300	-0.07198300
O	0.43521000	3.41770800	-0.66809600
C	3.86602800	-1.94908200	-0.13537700
C	2.81128400	-2.80107800	0.21603100

C	1.49429100	-2.33171000	0.25219800
C	1.20002200	-0.98801100	-0.07252000
C	2.27952900	-0.17564400	-0.41277900
C	3.59398900	-0.60495900	-0.44770200
C	-1.67862100	1.49109900	-0.07304400
H	4.88846500	-2.32132800	-0.15942900
H	3.01458500	-3.83792400	0.47314600
H	0.70250700	-3.01375400	0.55149100
C	-1.30675200	-1.25848300	-0.03808100
H	4.40712900	0.07028600	-0.71111200
C	-2.60606500	-0.73119900	-0.02420500
C	-2.80129900	0.65726700	-0.04186900
H	-3.79491300	1.09112200	-0.04312900
H	-1.80928100	2.56999200	-0.10757900
H	-1.20432700	-2.33891400	-0.05346500
O	-3.61343400	-1.65326700	-0.00788800
O	1.32299400	2.32137600	1.45319700
C	-4.96691700	-1.18601500	0.00127400
H	-5.58798000	-2.08388900	0.01681100
H	-5.19068900	-0.60132400	-0.89979700
H	-5.17223300	-0.58317300	0.89471000

Int2m’

S	1.53910100	1.79124300	0.06795300

C	1.01438300	-1.00868400	0.02885400
C	2.00583100	-0.00476000	0.06717300
O	2.72676000	2.43670700	0.80363600
C	-3.15247000	0.16823100	0.08631900
C	-2.81816300	-1.18321600	-0.10002700
C	-1.47548200	-1.57848300	-0.12240000
C	-0.42822800	-0.64898300	0.04089100
C	-0.81718100	0.67983500	0.22028600
C	-2.12089300	1.11930700	0.24440900
C	3.36383500	-0.32581500	0.07551000
O	-4.42754500	0.65170500	0.12449900
H	-3.58890500	-1.93408800	-0.23233000
H	-1.26278200	-2.63289000	-0.27962800
C	1.45271600	-2.34905100	-0.00433000
H	-2.38516900	2.16688800	0.38280500
C	2.81043300	-2.67135600	-0.01082200
C	3.77688800	-1.65926900	0.02871800
H	4.83590700	-1.90631400	0.03087100
H	4.09208700	0.47986200	0.12958600
H	0.72748100	-3.15711600	-0.01736400
H	3.11240500	-3.71571900	-0.03688800
O	1.53581500	2.14458800	-1.42722400
C	-5.51530200	-0.26730000	-0.03186000
H	-6.42261600	0.33640200	0.03520000

H	-5.51562900	-1.01998400	0.76647500
H	-5.47684700	-0.76365700	-1.00957400

Int1n

S	1.83402300	-1.42643500	0.00012000
C	0.80708100	1.01332400	0.00006800
C	2.06737600	0.32437100	0.00007900
O	2.36121500	-2.07054100	-1.24817600
C	-2.15063200	-2.11466500	-0.00028200
C	-2.65687300	-0.76769200	-0.00013600
C	-1.75322600	0.36504100	0.00004500
C	-0.32683200	0.11206200	0.00000600
C	0.08731700	-1.26286900	-0.00003500
C	-0.79122700	-2.35307100	-0.00020900
C	3.30840900	0.95411200	-0.00010000
H	-2.85797300	-2.94028200	-0.00047300
C	-4.05347200	-0.54147900	-0.00015500
C	-2.34189700	1.65744200	0.00022500
C	0.90122000	2.43140600	-0.00000500
H	-0.40524900	-3.37043000	-0.00025700
C	2.13832400	3.07022000	-0.00015200
C	3.35303800	2.34907300	-0.00023500
H	4.30607600	2.87051400	-0.00039400
H	4.22361000	0.36594800	-0.00012600

H	0.01382700	3.04997600	0.00004300
H	2.16358900	4.15776000	-0.00022600
O	2.36090200	-2.07055400	1.24855800
C	-3.72225500	1.84493800	0.00021200
C	-4.59062200	0.74182800	0.00001700
H	-4.71410800	-1.40676800	-0.00031300
H	-1.71490000	2.53742200	0.00036700
H	-4.12220800	2.85650200	0.00036800
H	-5.66835000	0.88684000	-0.00000800

TS1n

S	-2.07956800	-1.44261500	0.30124800
C	-0.72351100	0.92779200	-0.20390100
C	-1.99383400	0.36509600	0.04856300
O	-2.90457900	-1.56488800	1.60013500
C	2.27704500	-2.15464200	-0.42302700
C	2.74825800	-0.83159400	-0.16838100
C	1.81058100	0.25527200	-0.01231000
C	0.41132800	-0.02846700	-0.22525000
C	0.01933200	-1.34481200	-0.42491800
C	0.92359500	-2.41986100	-0.48300800
C	-3.15362700	1.13560500	0.09384900
H	3.00725100	-2.95409500	-0.54055500
C	4.14033800	-0.58304900	-0.01705200

C	2.33588600	1.51878300	0.38744400
C	-0.67516400	2.31102600	-0.46947800
H	0.56525300	-3.44141700	-0.60678100
C	-1.83725700	3.08683900	-0.44885400
C	-3.07944700	2.50844900	-0.15689100
H	-3.97988500	3.11750300	-0.13996300
H	-4.10766200	0.66281400	0.31505900
H	0.26241200	2.78442000	-0.73671300
H	-1.77166400	4.14913100	-0.67181800
O	-2.84614300	-2.00094400	-0.89571400
C	4.61357500	0.66916300	0.32141700
C	3.69477400	1.72153200	0.54626800
H	4.83138200	-1.41209000	-0.15713700
H	1.66185400	2.33832000	0.60245500
H	5.68064100	0.84223800	0.43742600
H	4.05804200	2.69887500	0.85587500

TS1n’

S	-1.94755900	-1.55217000	-0.01856000
C	0.26987300	0.08446500	0.00432300
C	-0.15502600	-1.24332000	-0.03577600
O	-2.12913300	-2.57440900	-1.16349400
C	-3.16591200	2.66588600	-0.03485100
C	-1.93443100	3.20223800	-0.44704000

C	-0.77930700	2.41368300	-0.44797400
C	-0.82820500	1.07778500	0.00400500
C	-2.08259000	0.55072700	0.37891600
C	-3.24654600	1.31848200	0.33735000
C	0.73448900	-2.33325100	-0.14654400
H	-4.05695900	3.29131100	-0.02528900
H	-1.87787000	4.23293100	-0.78902900
H	0.14022000	2.83957900	-0.83618600
C	1.68942400	0.34182200	0.03771800
H	-4.20999000	0.87934000	0.59687100
C	2.59638500	-0.77096600	-0.08537300
C	2.08859400	-2.09922200	-0.18745800
H	2.79156600	-2.92439500	-0.27820800
H	0.33859200	-3.34327400	-0.21268600
C	2.25647100	1.63012700	0.25728000
C	3.99893900	-0.53657400	-0.07139300
O	-2.28473600	-2.18748800	1.32726700
C	4.50966500	0.73417800	0.09860000
C	3.62513100	1.82229900	0.28639000
H	1.60979100	2.47732100	0.44483400
H	4.66686500	-1.38823200	-0.18250700
H	5.58440700	0.89749000	0.11255300
H	4.02355600	2.81767500	0.46738400

Int2n

S	1.26442300	1.63719900	0.90042400
C	0.99205000	-0.99369700	-0.19713600
C	1.83374100	0.12236700	-0.04719900
O	1.61290400	1.22245200	2.34752200
C	-3.08440700	-1.12338300	1.31209500
C	-2.80002000	-0.29168500	0.19083900
C	-1.46472200	-0.22417600	-0.33955100
C	-0.42281300	-1.00069700	0.29180500
C	-0.81233500	-1.77686700	1.34233200
C	-2.08767700	-1.88337400	1.90108900
C	3.14024800	0.08960000	-0.53830000
H	-4.10110800	-1.15867300	1.69870700
C	-3.82606300	0.48004500	-0.42589500
C	-1.22238500	0.59838700	-1.47481300
C	1.49868700	-2.14357000	-0.82736000
H	-2.29500400	-2.52499600	2.75369100
C	2.81130200	-2.17995300	-1.30636800
C	3.63501600	-1.05732100	-1.16609700
H	4.65508800	-1.07590500	-1.54356700
H	3.76043100	0.97587300	-0.42331000
H	0.85052300	-3.00858900	-0.94809500
H	3.18433400	-3.07772700	-1.79331700
O	2.23744400	2.71180500	0.37595100

C	-3.55643700	1.27443000	-1.52056500
C	-2.24237400	1.32876000	-2.05069100
H	-4.83252300	0.42809800	-0.01626300
H	-0.21983300	0.64994100	-1.88803100
H	-4.34945800	1.85758700	-1.98187000
H	-2.03686300	1.95196400	-2.91762600

Int2n'

S	-1.97468000	-1.89622700	-0.02159400
C	0.16100700	-0.02463000	-0.02531400
C	-0.19142900	-1.36119400	-0.02412500
O	-2.00241700	-2.84139600	-1.24198800
C	-2.85220700	3.09336600	-0.05722900
C	-2.35908500	2.59797000	1.15614600
C	-1.38683500	1.59200400	1.17679500
C	-0.88374500	1.05006200	-0.02482900
C	-1.40756100	1.59102000	-1.18347400
C	-2.36557400	2.57783900	-1.27360900
C	0.79589400	-2.38001700	-0.01454300
H	-3.60930500	3.87446700	-0.06712800
H	-2.73622700	2.99606200	2.09478600
H	-1.01144400	1.21032200	2.12419100
C	1.55186500	0.34245100	-0.01808100
H	-2.73597200	2.94753000	-2.22690500

C	2.54603300	-0.69293800	-0.00678000
C	2.13120600	-2.05647600	-0.00543700
H	2.89117200	-2.83562900	0.00187900
H	0.47595900	-3.41898100	-0.01733500
C	1.98646400	1.69837800	-0.02033200
C	3.92209000	-0.33435300	0.00180200
O	-2.03997700	-2.69577300	1.29839300
C	4.31056000	0.99039200	-0.00055900
C	3.33123600	2.01454700	-0.01194600
H	1.24645400	2.49295400	-0.02854600
H	4.66626000	-1.12815300	0.01034000
H	5.36598500	1.25126500	0.00613900
H	3.64162800	3.05661000	-0.01407400

25) NMR Spectra

1b ^{1}H NMR (400 MHz, $CDCl_3$)

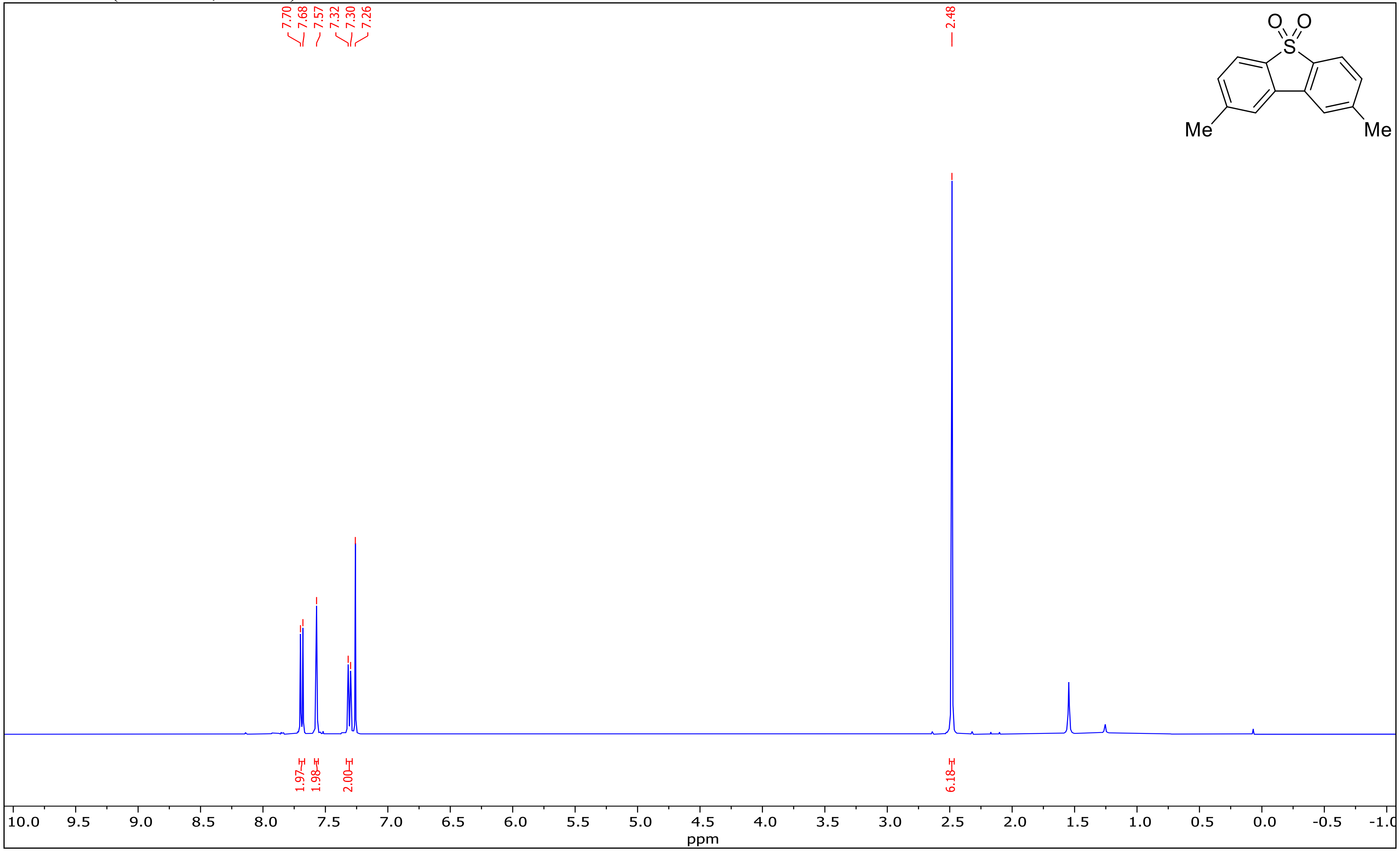

1b ^{13}C NMR (101 MHz, $CDCl_3$)

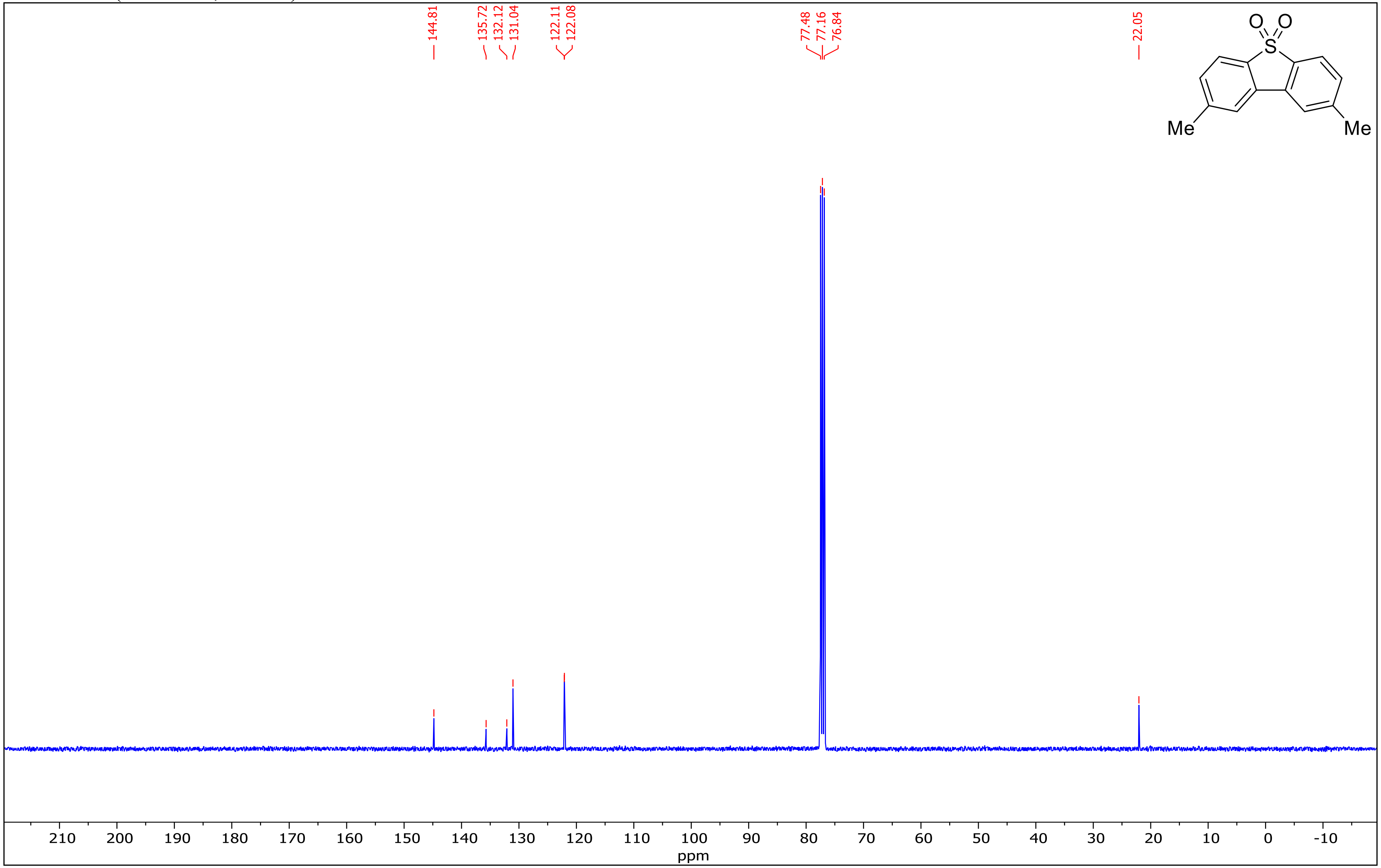

1c ^{1}H NMR (400 MHz, $CDCl_3$)

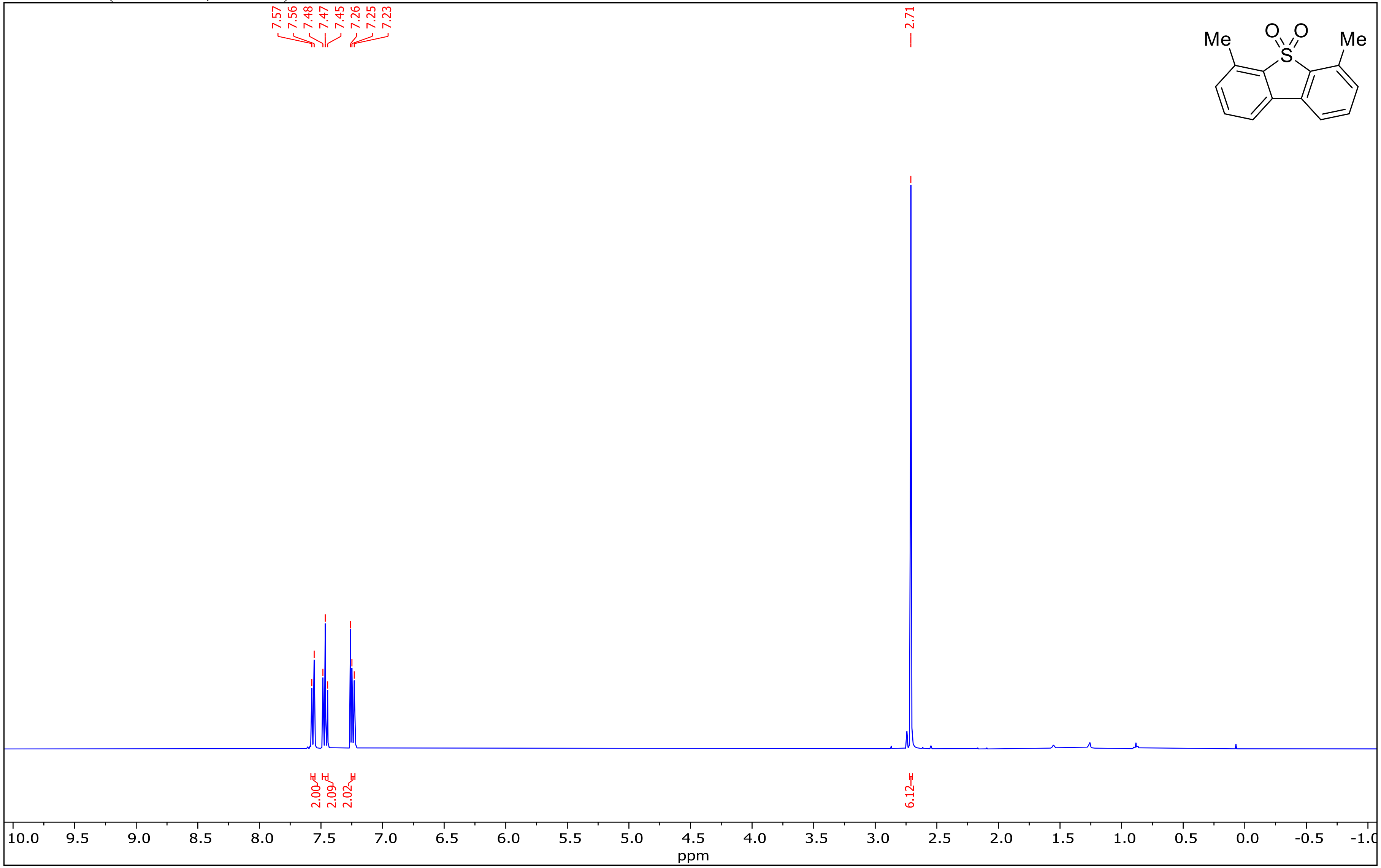

1c ^{13}C NMR (101 MHz, CDCl$_3$)

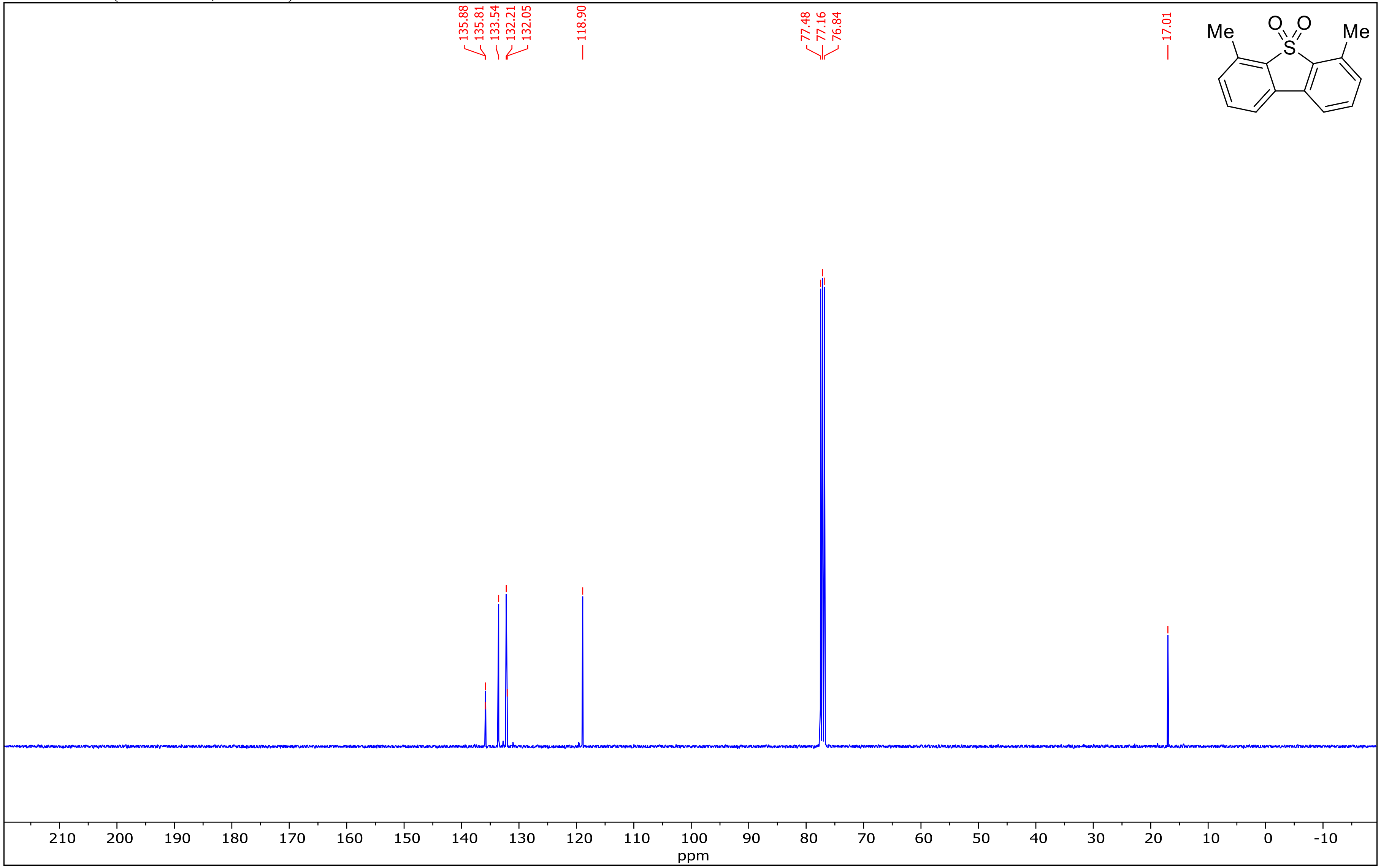

1d ¹H NMR (400 MHz, DMSO-d$_6$)

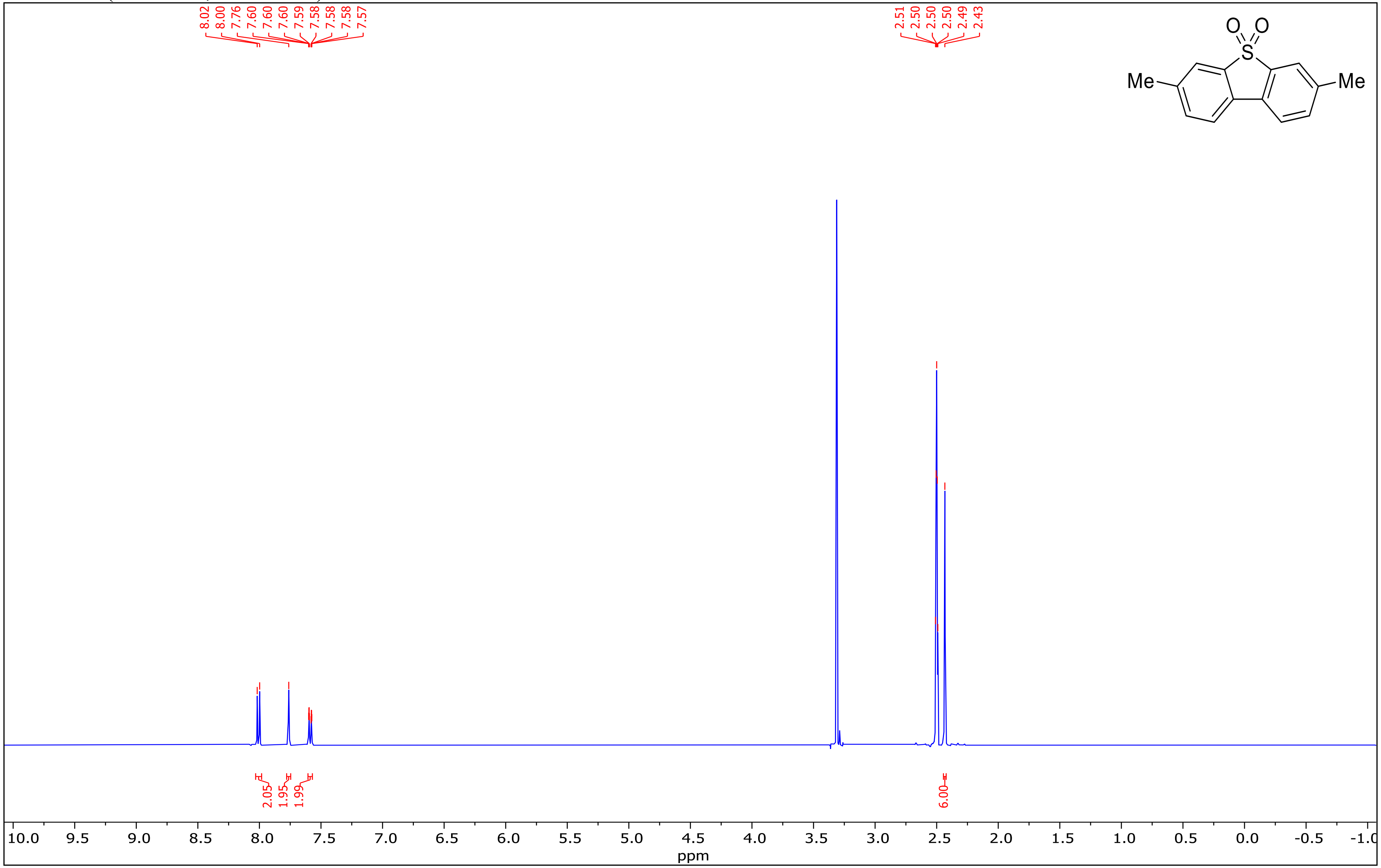

1d ^{13}C NMR (101 MHz, $CDCl_3$)

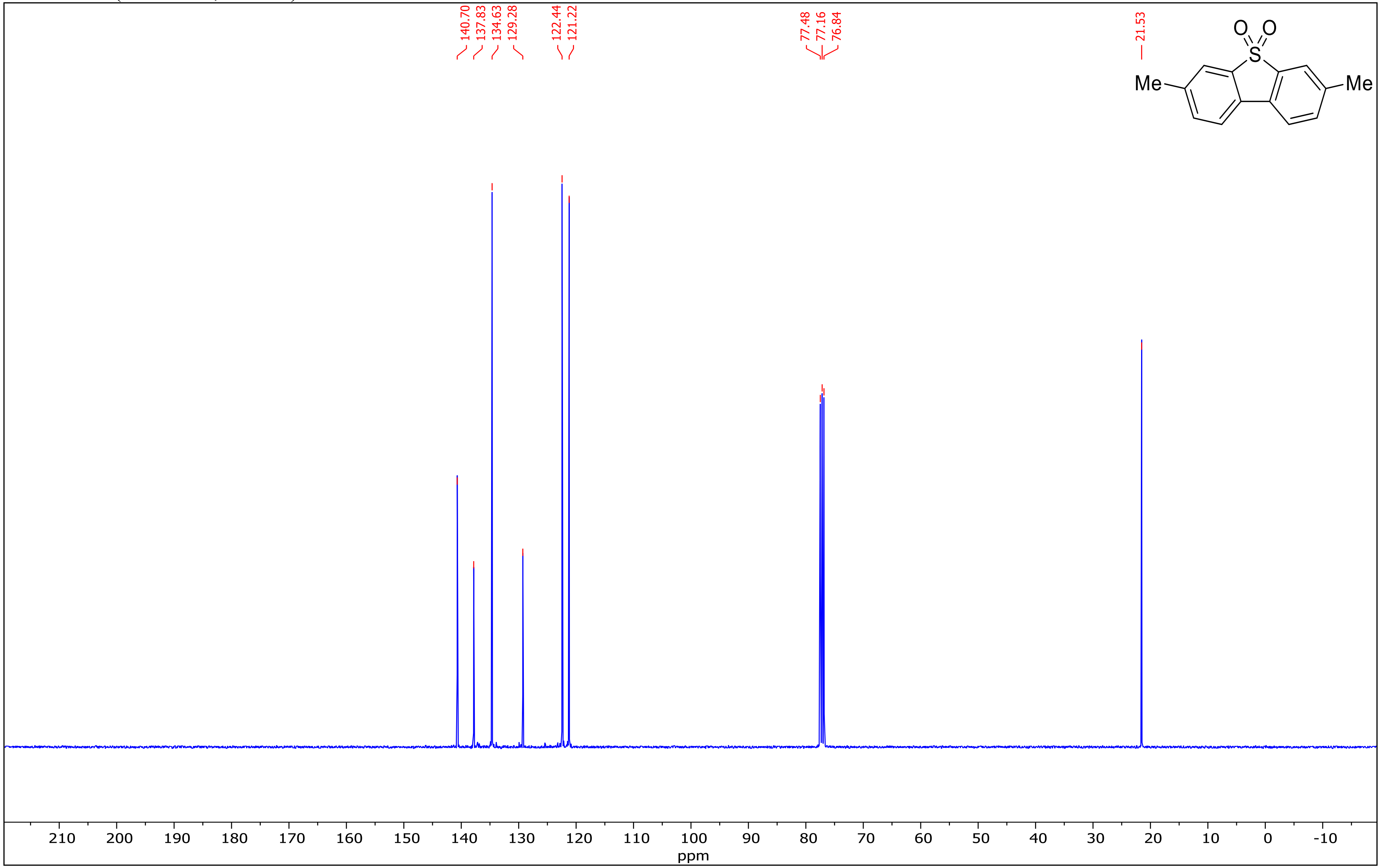

1e ^{1}H NMR (400 MHz, $CDCl_3$)

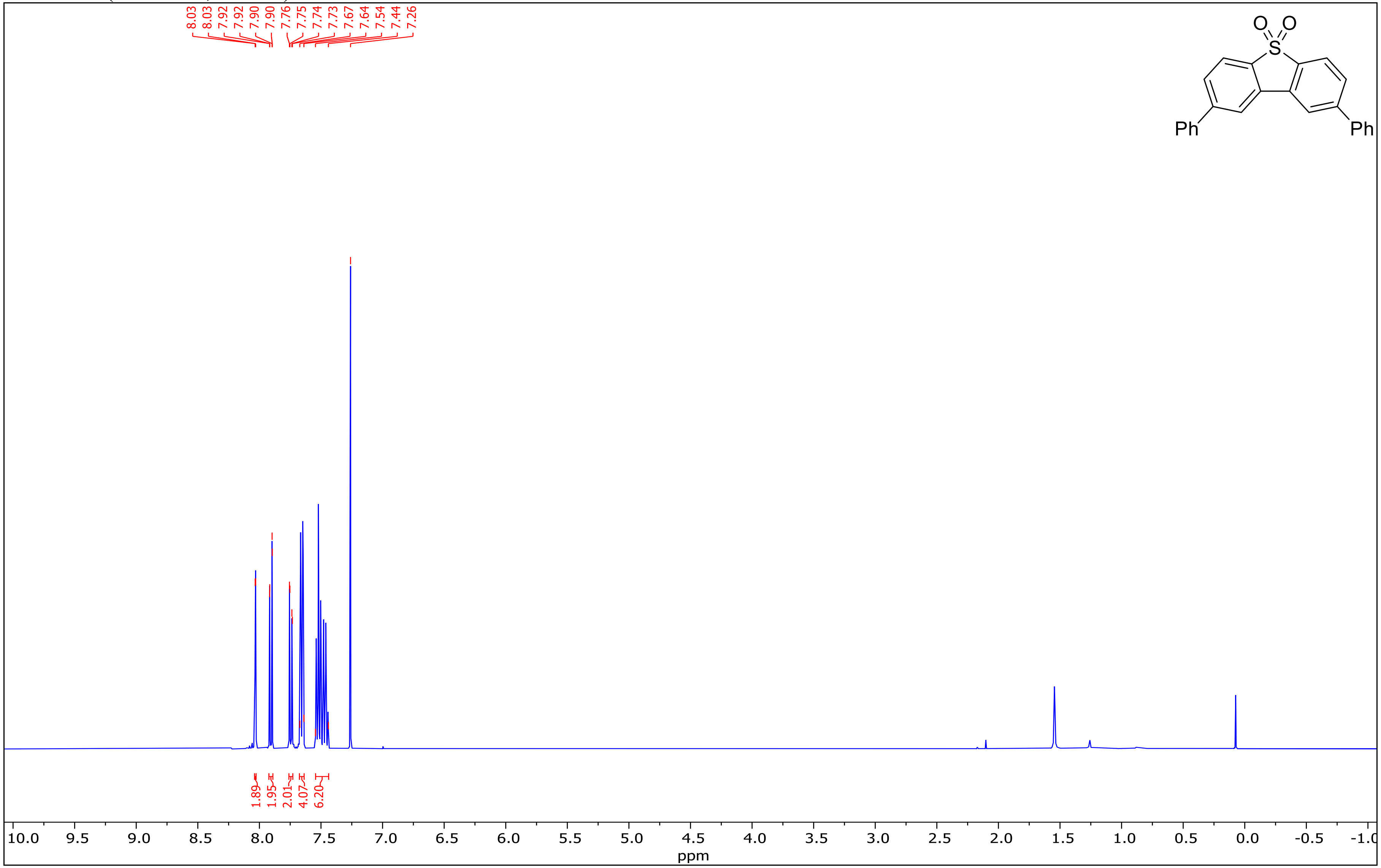

1e ^{13}C NMR (101 MHz, $CDCl_3$)

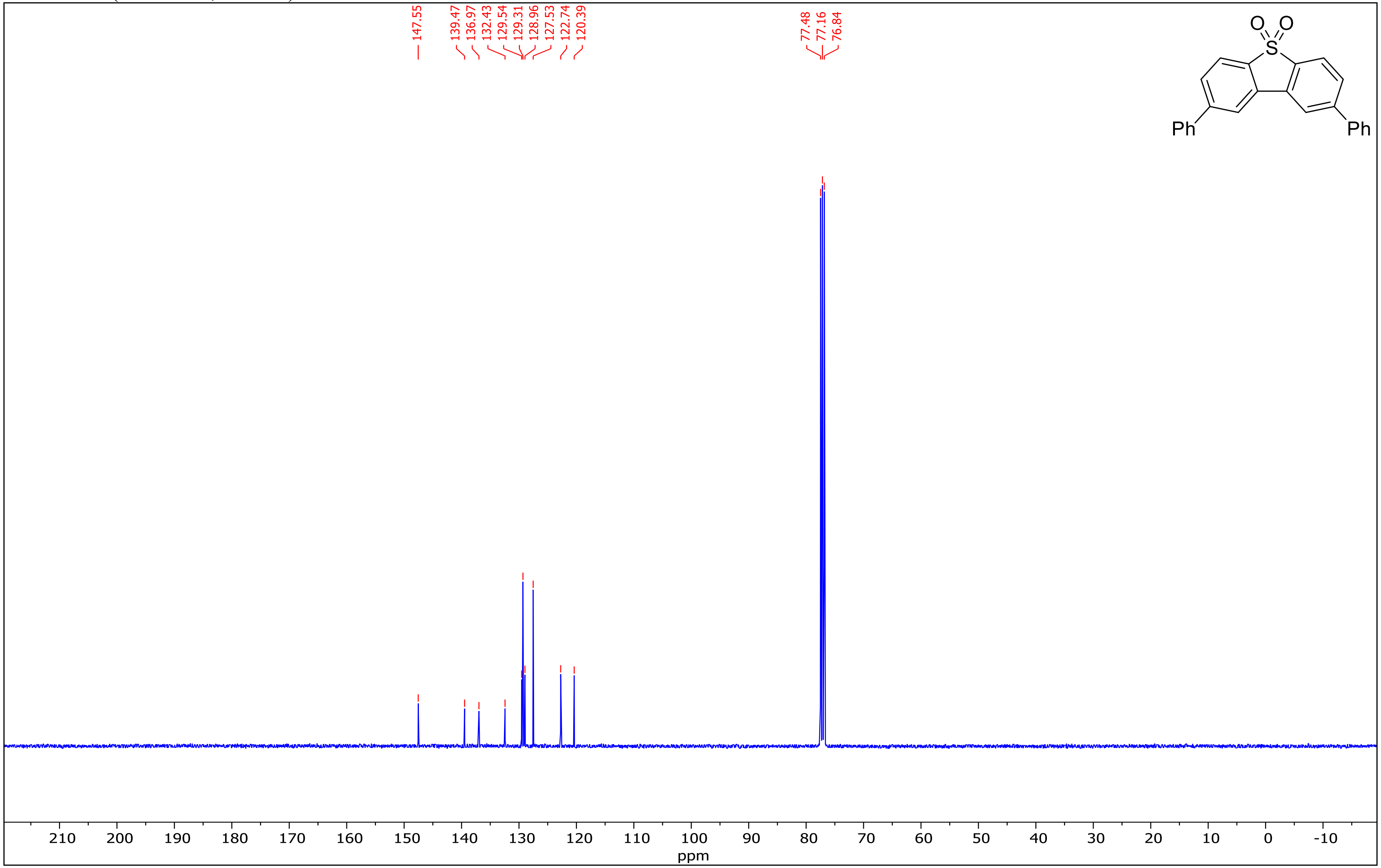

1f ^{1}H NMR (400 MHz, $CDCl_3$)

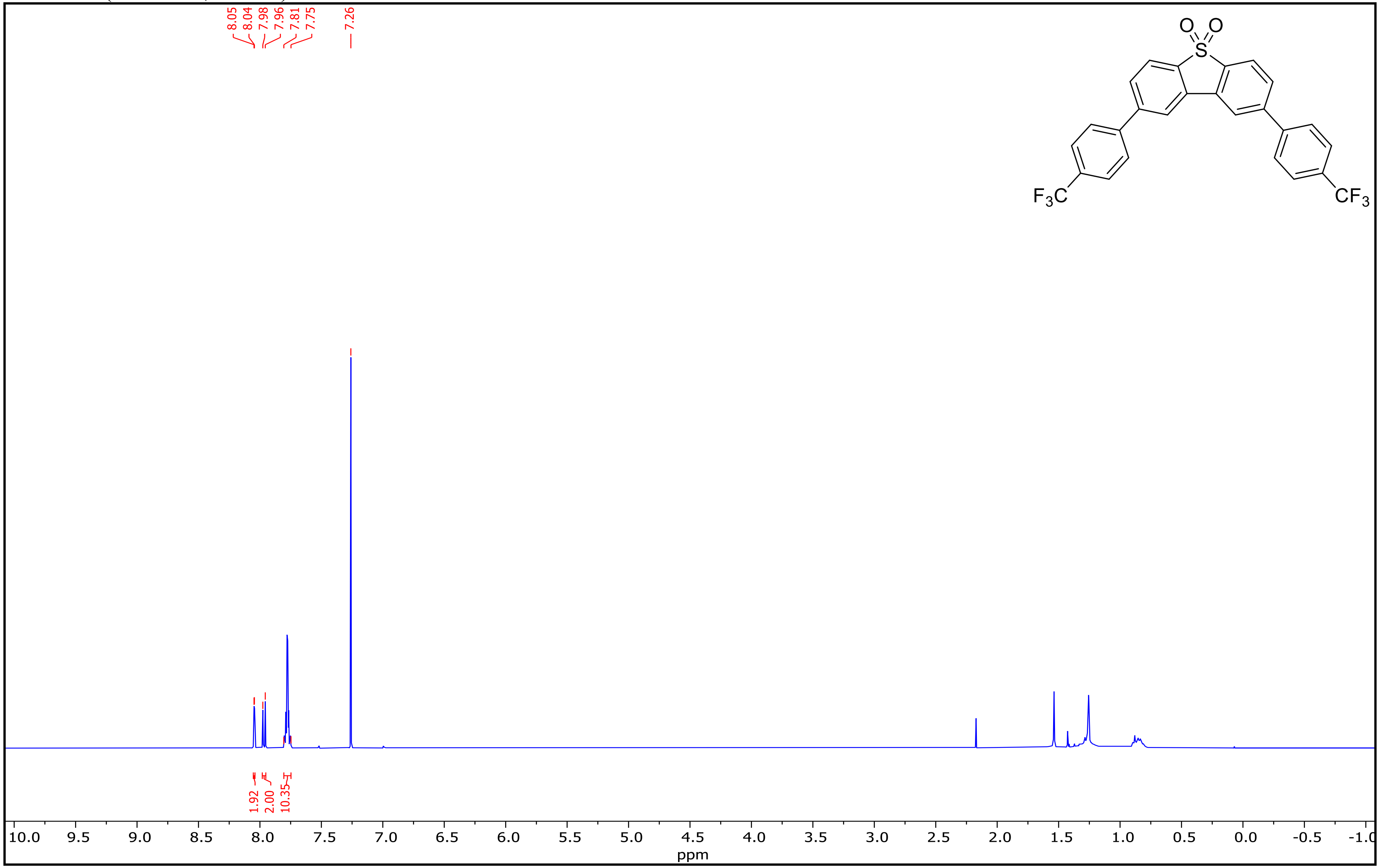

1f ^{13}C NMR (101 MHz, $CDCl_3$)

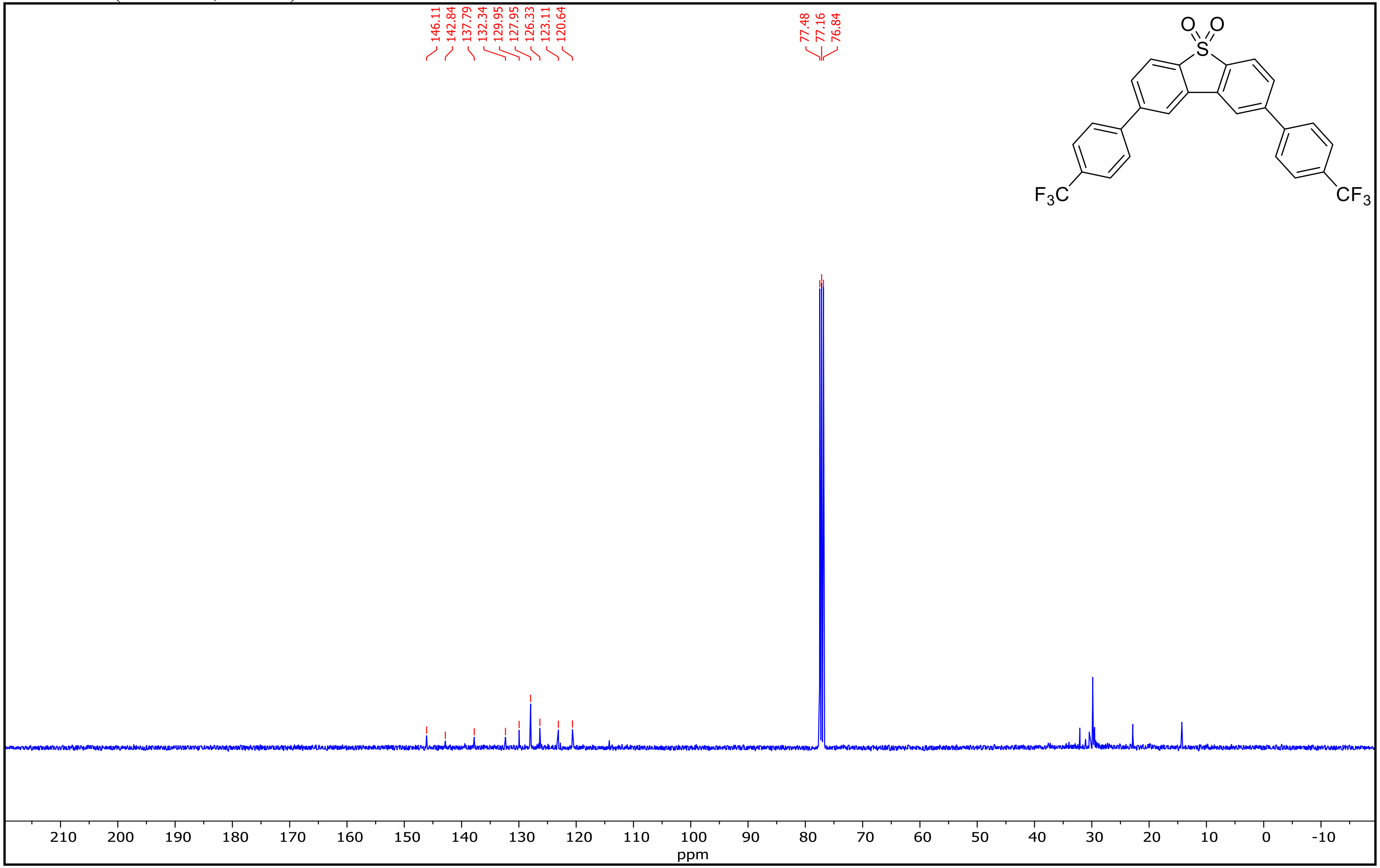

1f ^{19}F NMR (376 MHz, $CDCl_3$)

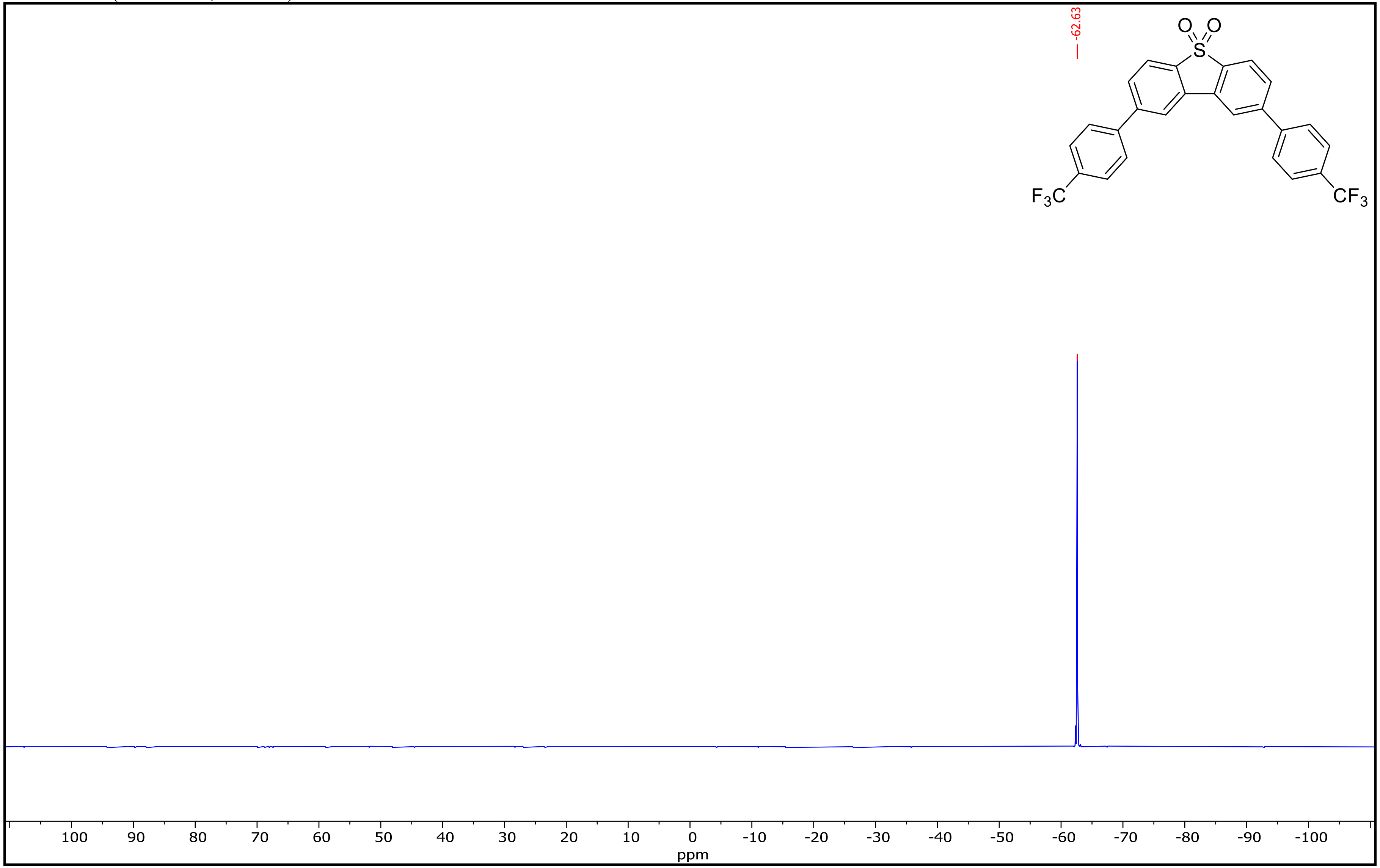

1g ^{1}H NMR (400 MHz, $CDCl_3$)

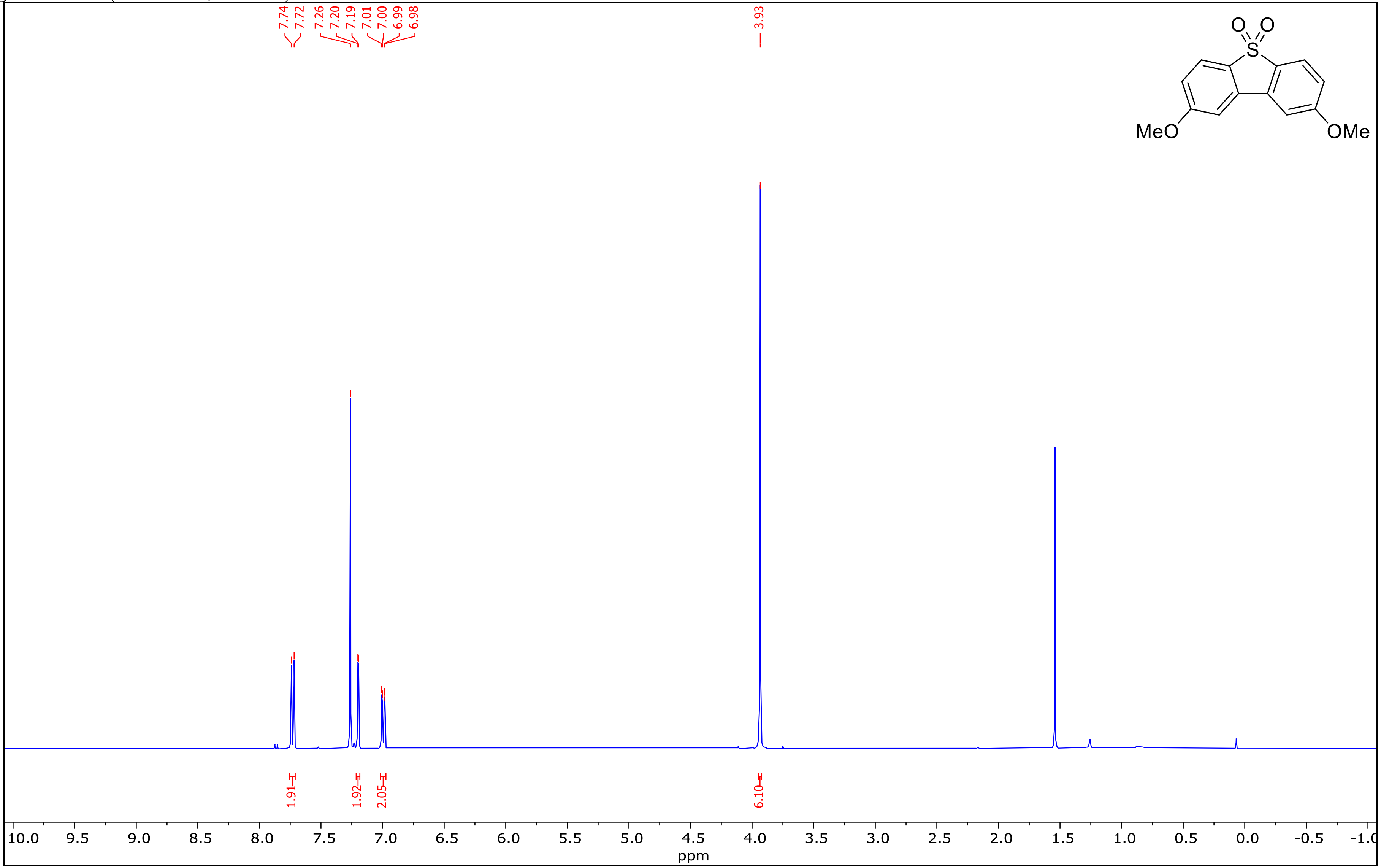

1g ^{13}C NMR (101 MHz, $CDCl_3$)

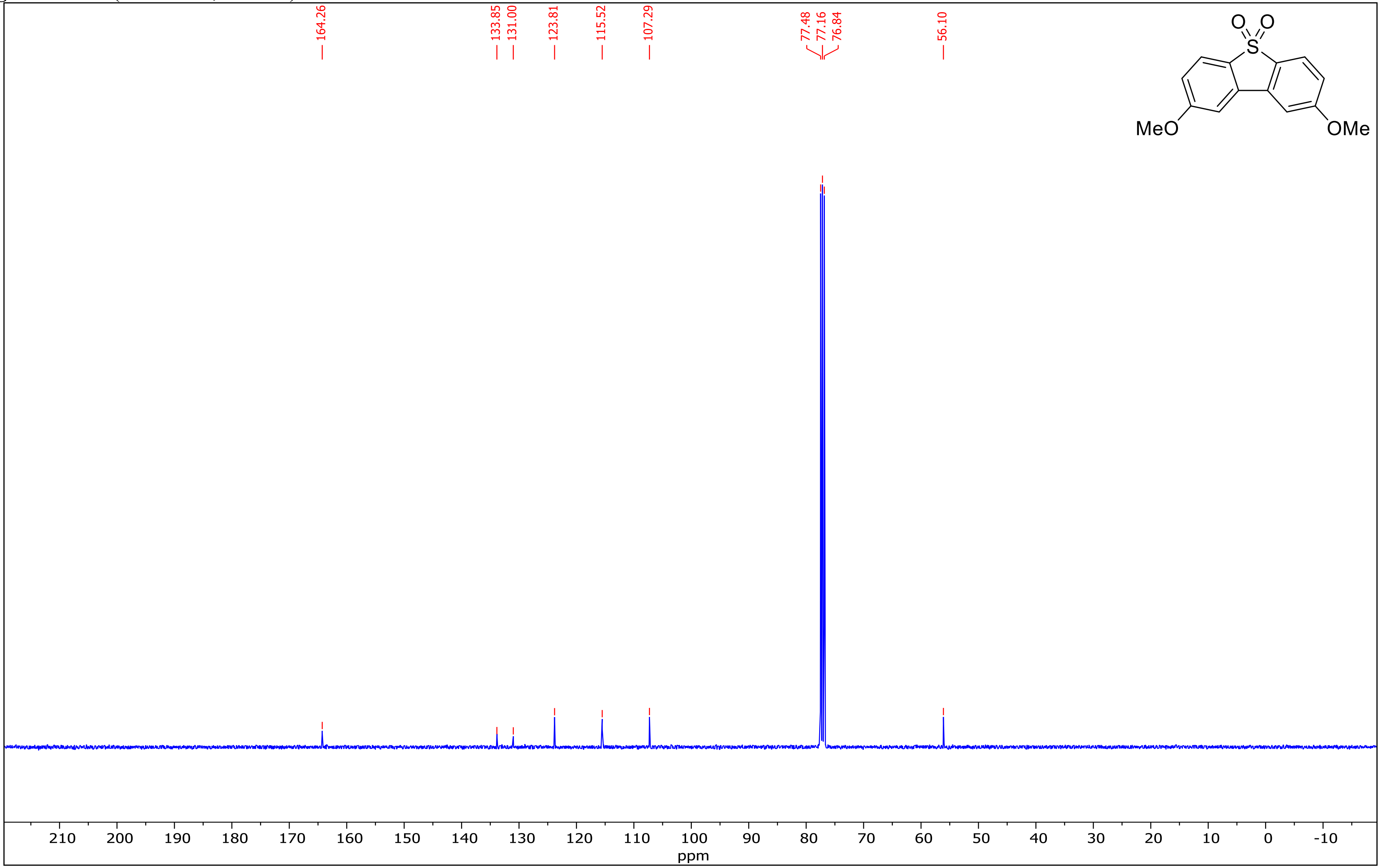

1h ^{1}H NMR (400 MHz, $CDCl_3$)

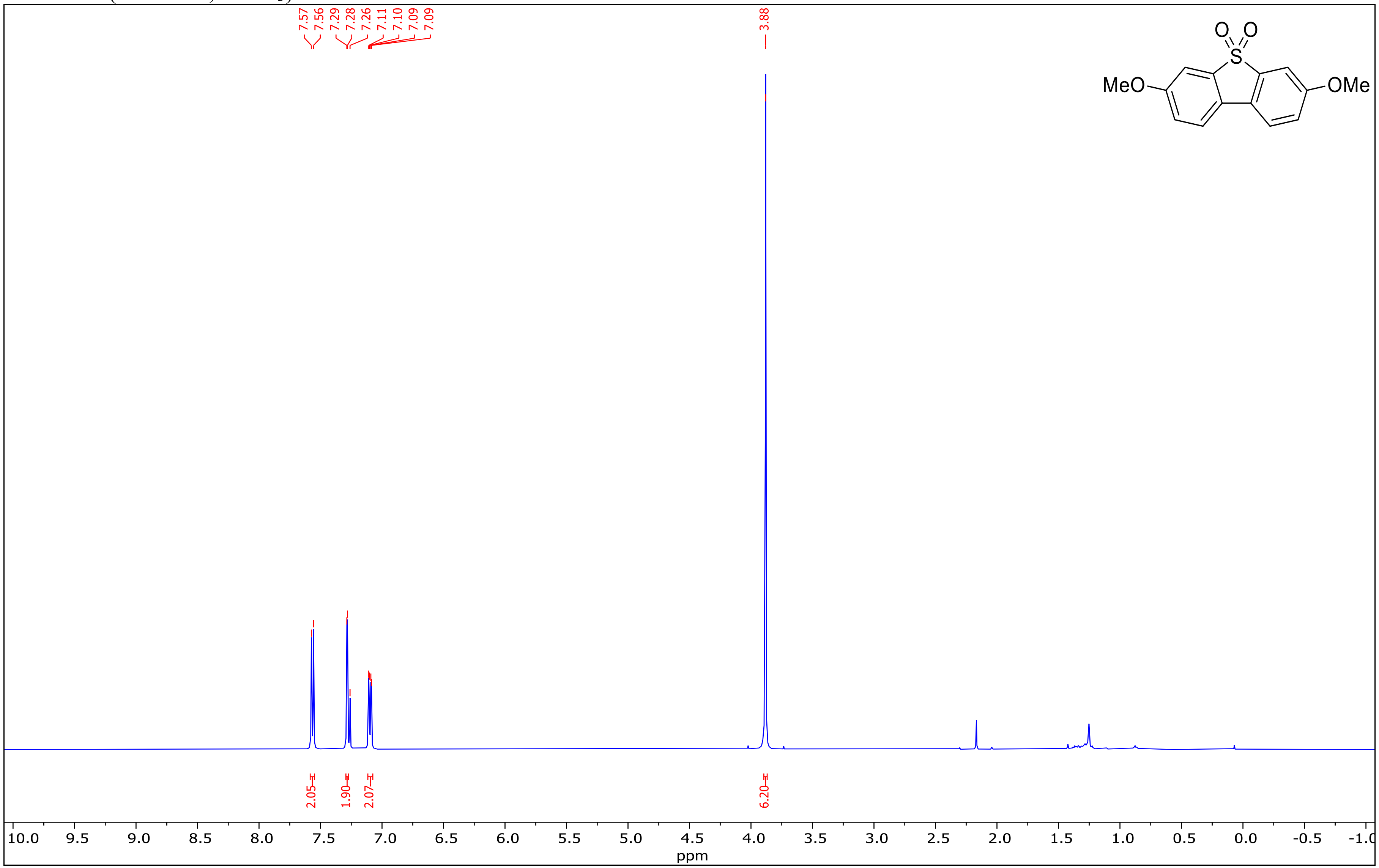

1h ^{13}C NMR (101 MHz, $CDCl_3$)

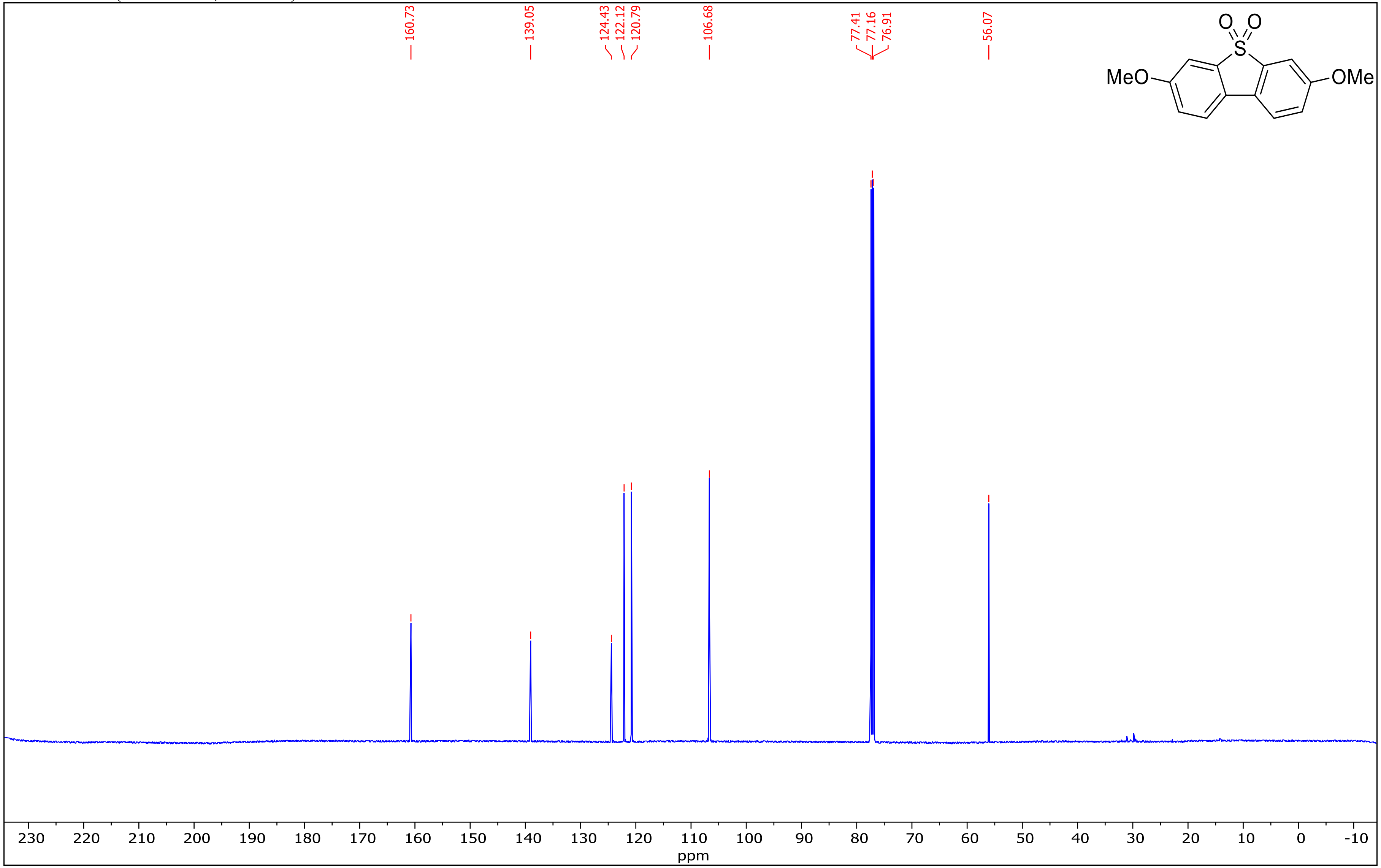

1i ^{1}H NMR (400 MHz, $CDCl_3$)

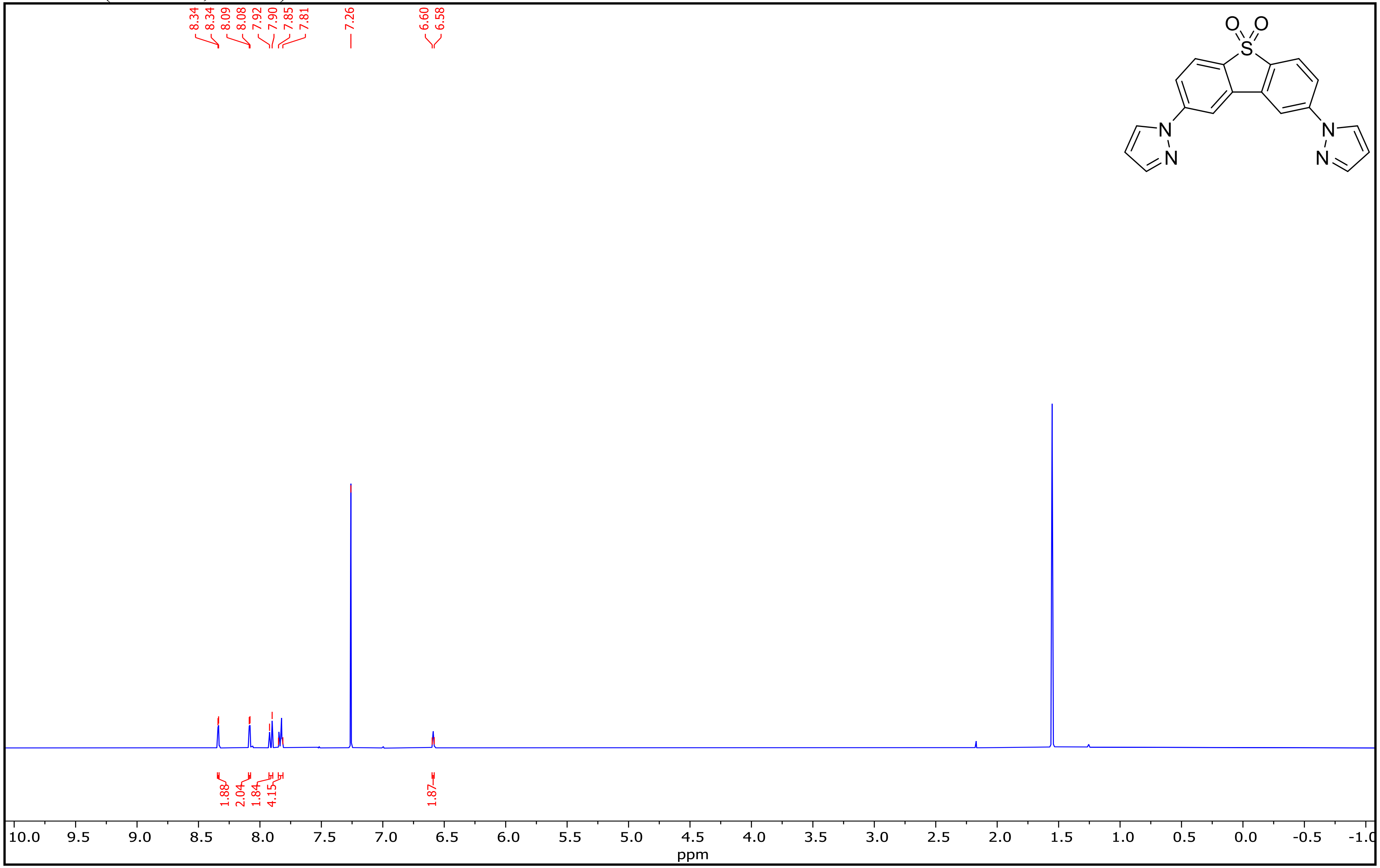

1i ^{13}C NMR (101 MHz, $CDCl_3$)

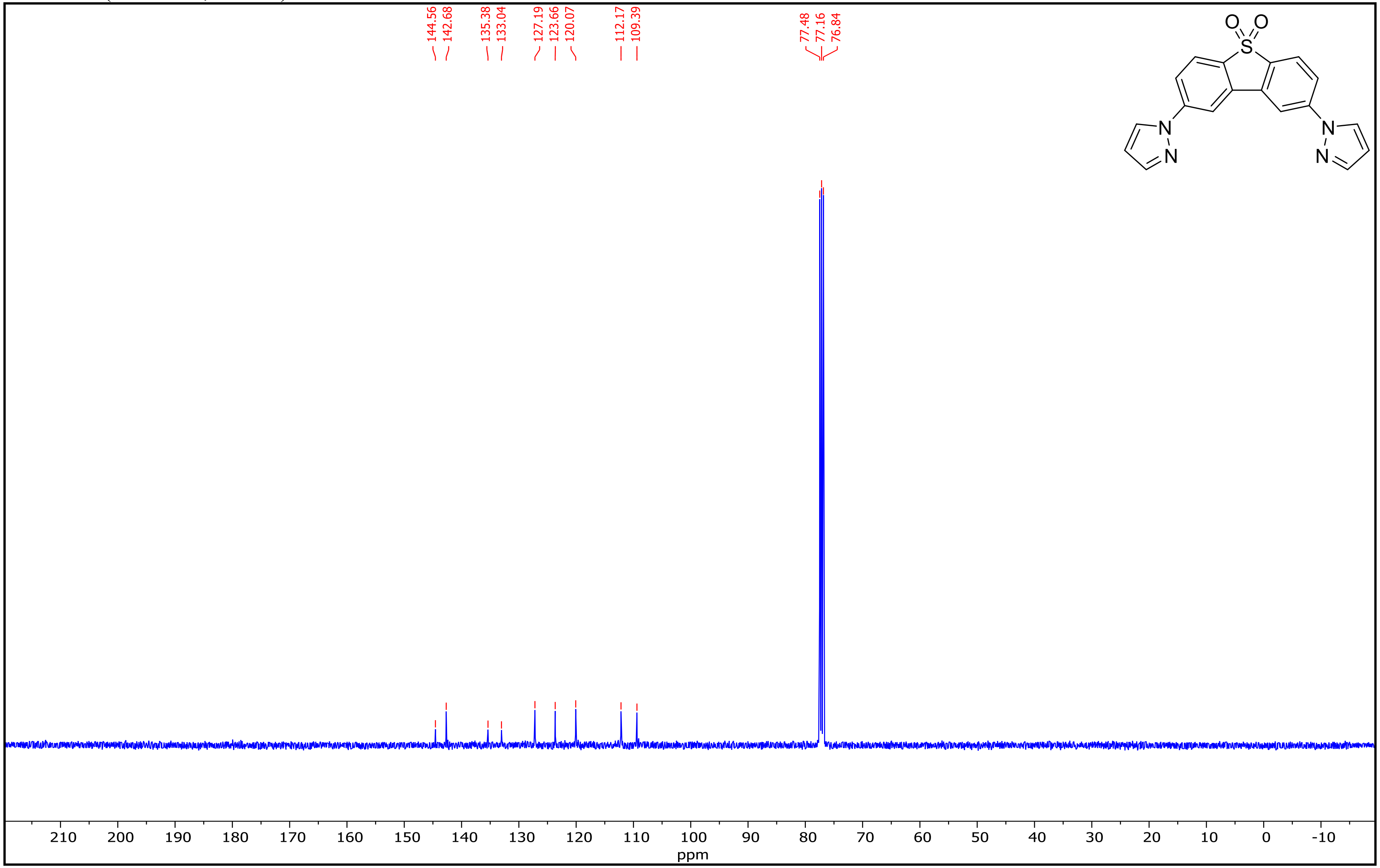

1j ^{1}H NMR (400 MHz, $CDCl_3$)

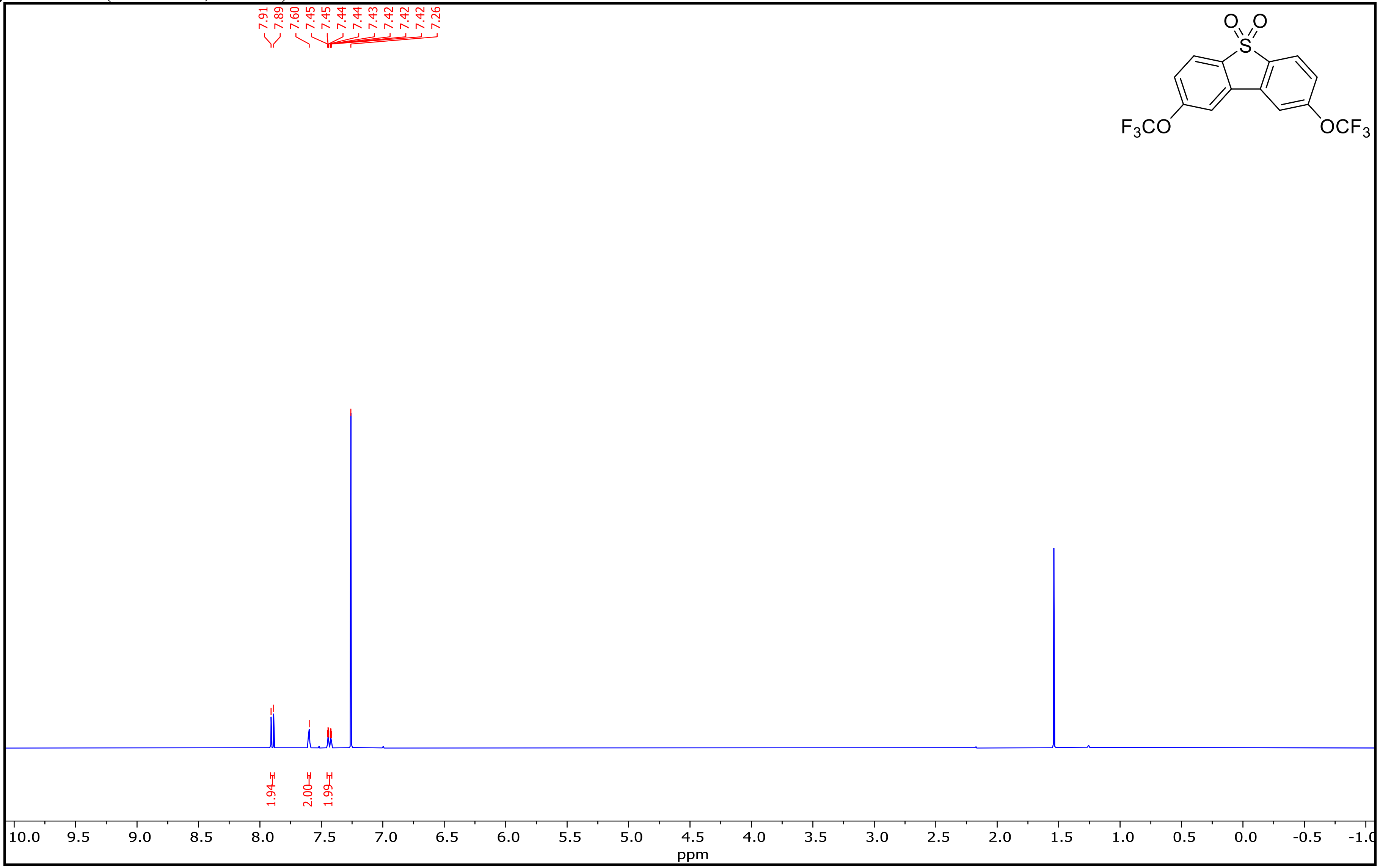

1j ^{13}C NMR (101 MHz, CDCl$_3$)

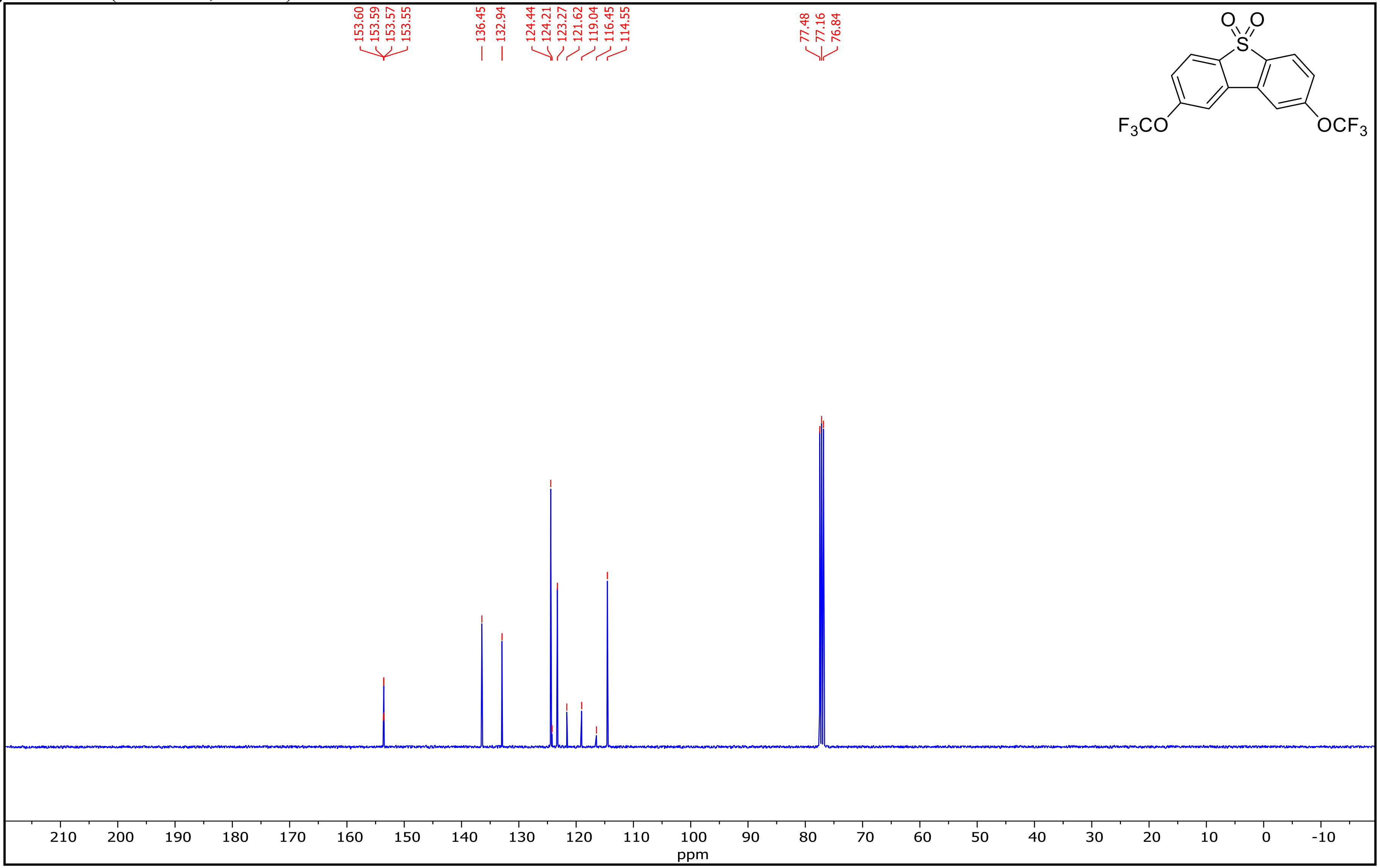

1j ^{19}F NMR (376 MHz, $CDCl_3$)

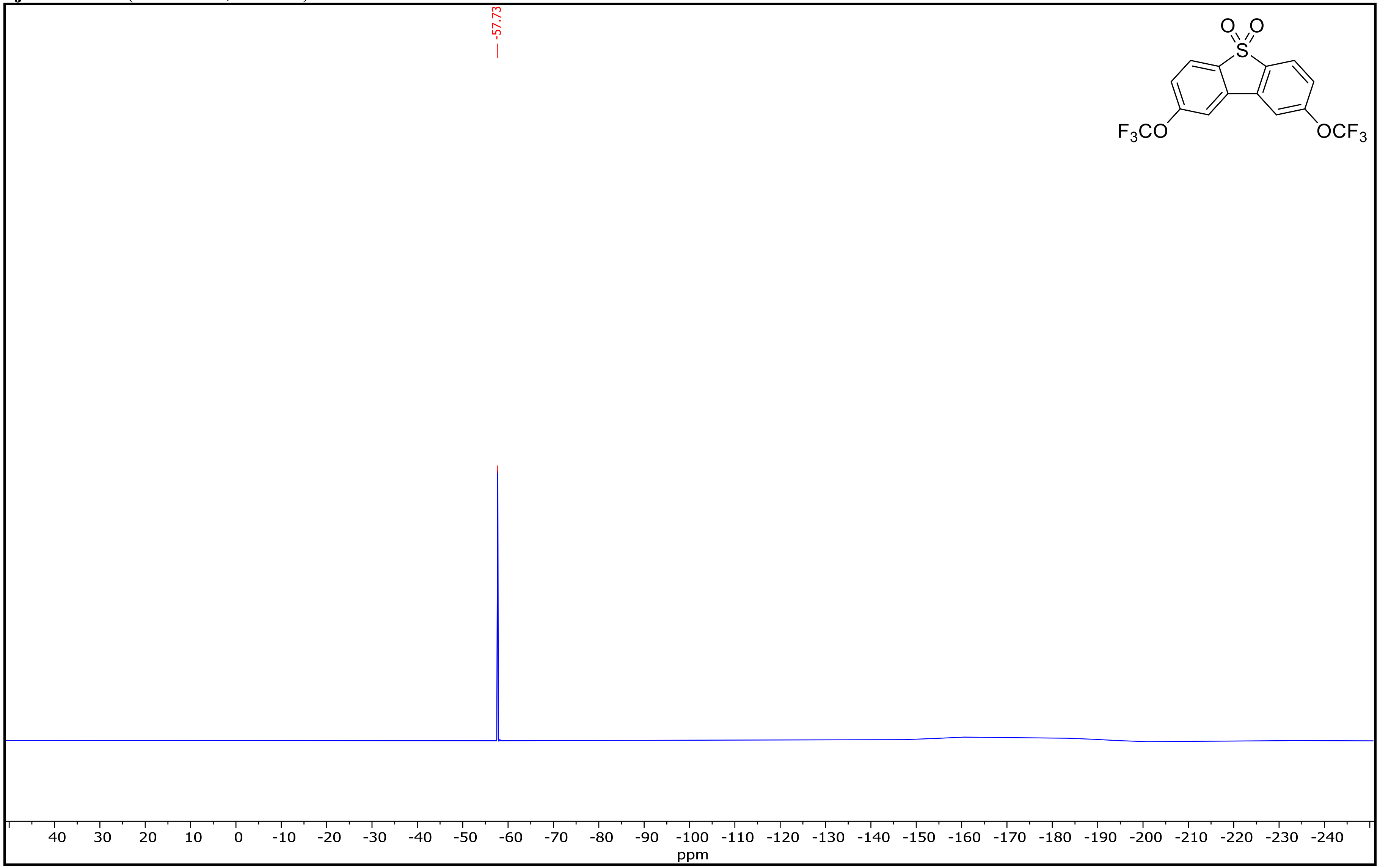

1k ^{1}H NMR (400 MHz, DMSO-d$_6$)

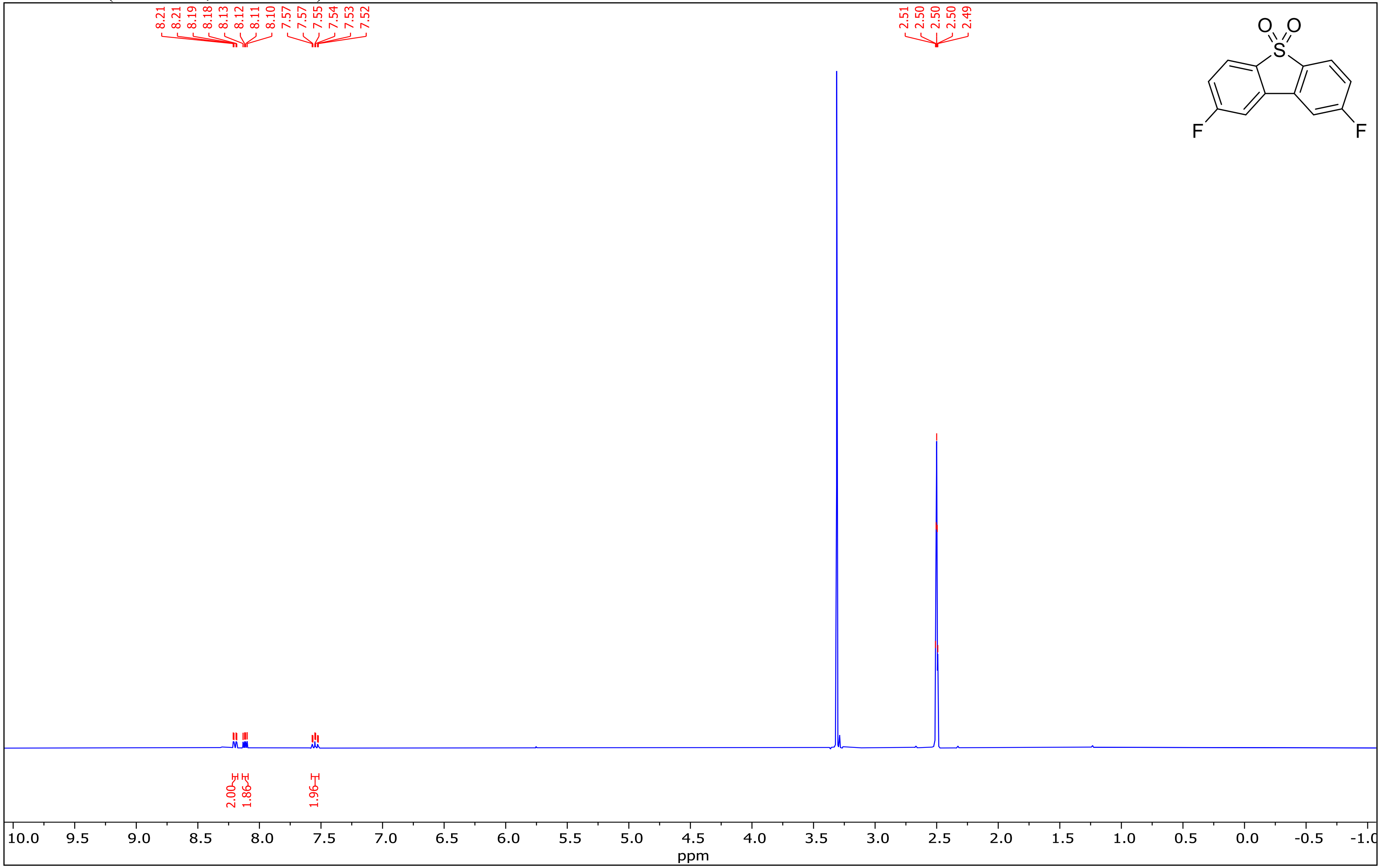

1k ^{13}C NMR (101 MHz, $CDCl_3$)

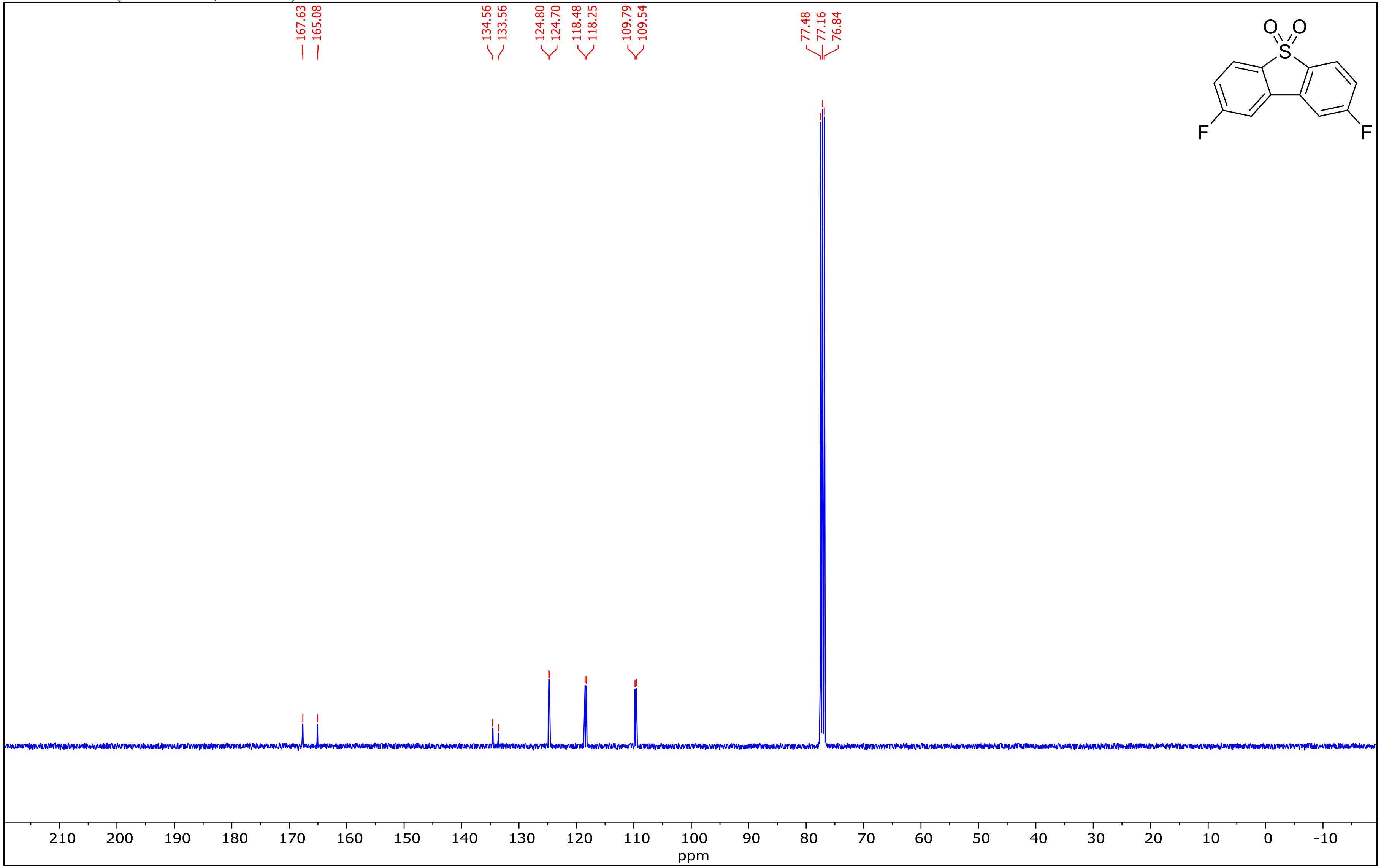

1k ^{19}F NMR (376 MHz, $CDCl_3$)

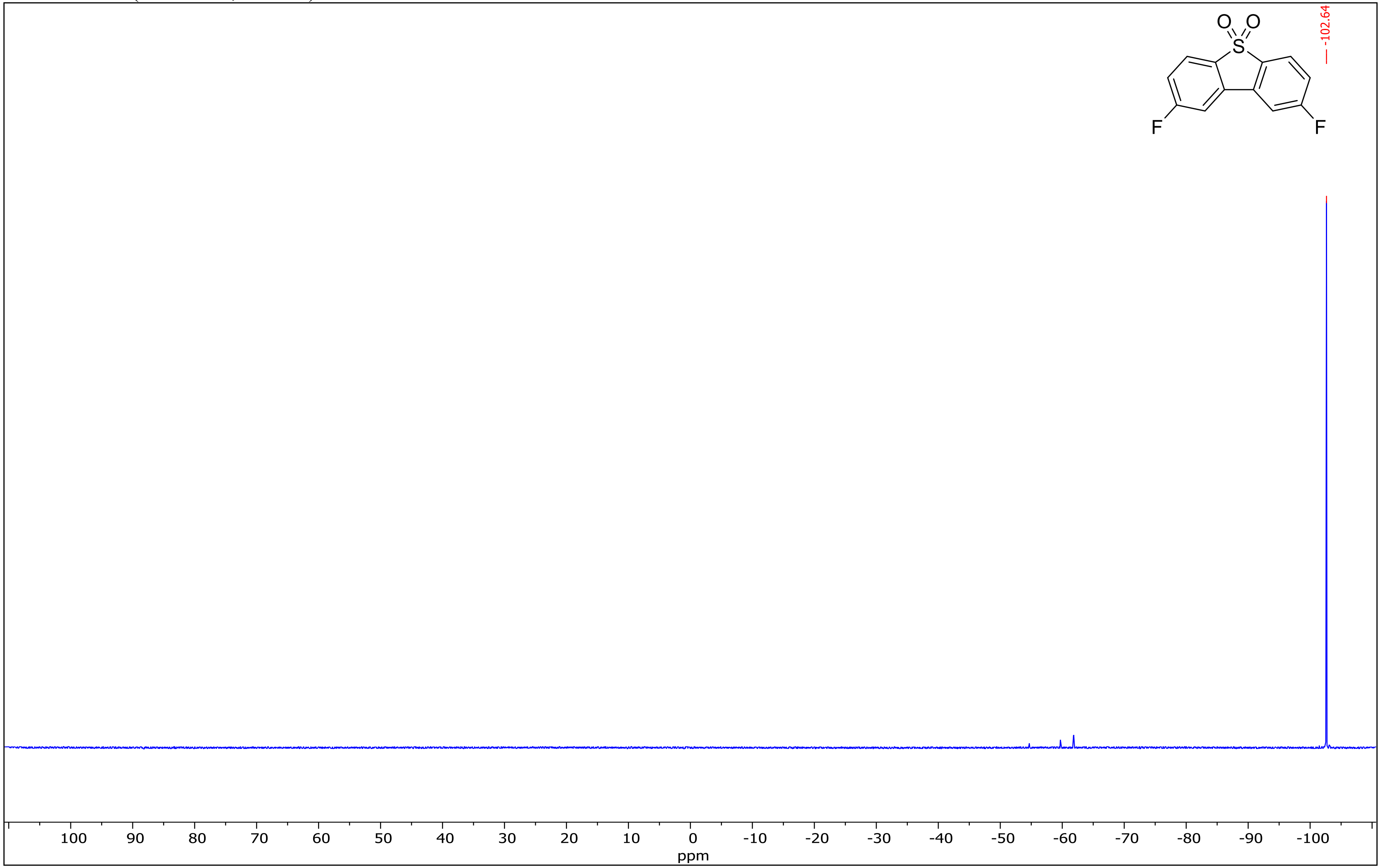

1l ^{1}H NMR (400 MHz, $CDCl_3$)

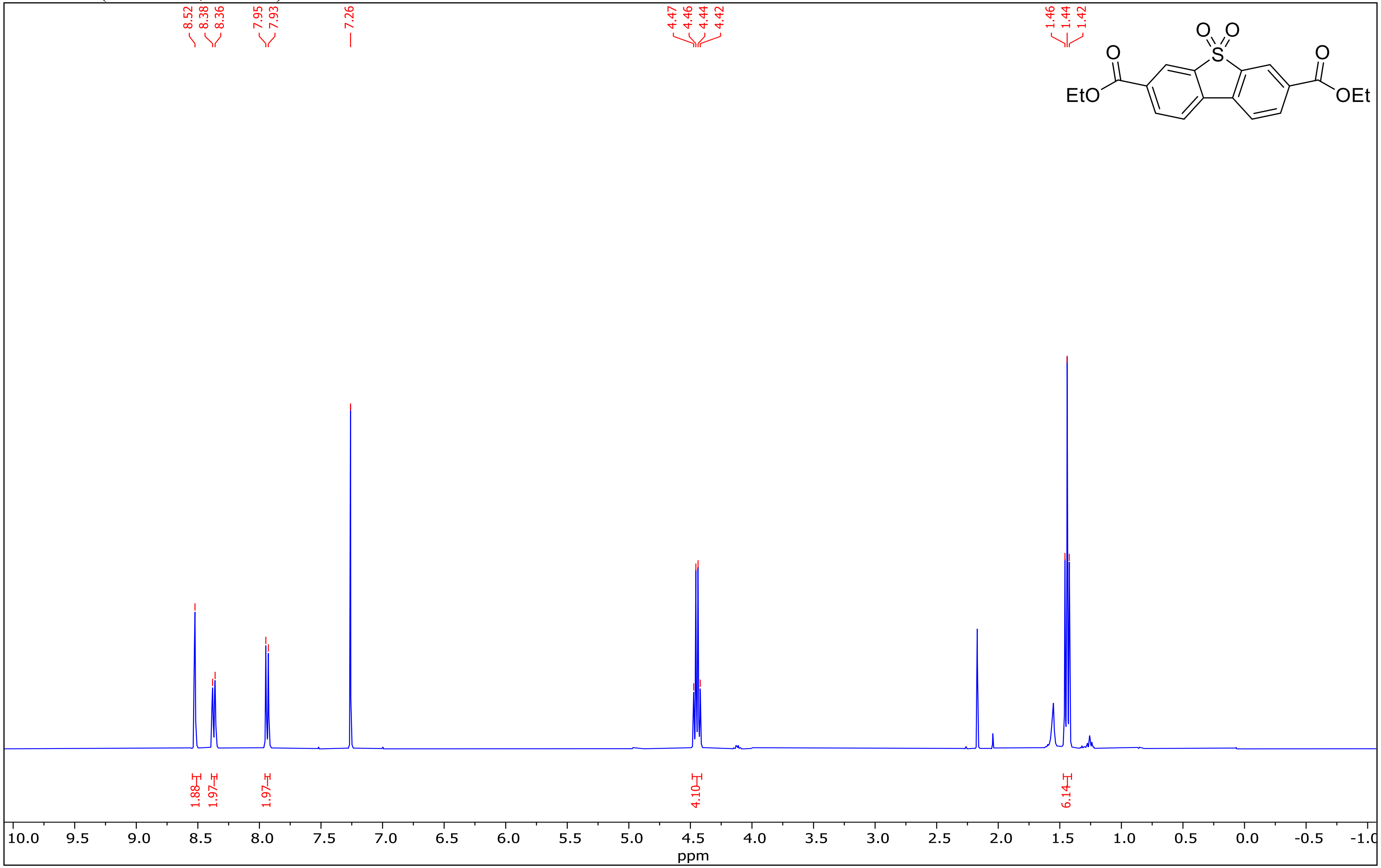

1l ^{13}C NMR (101 MHz, $CDCl_3$)

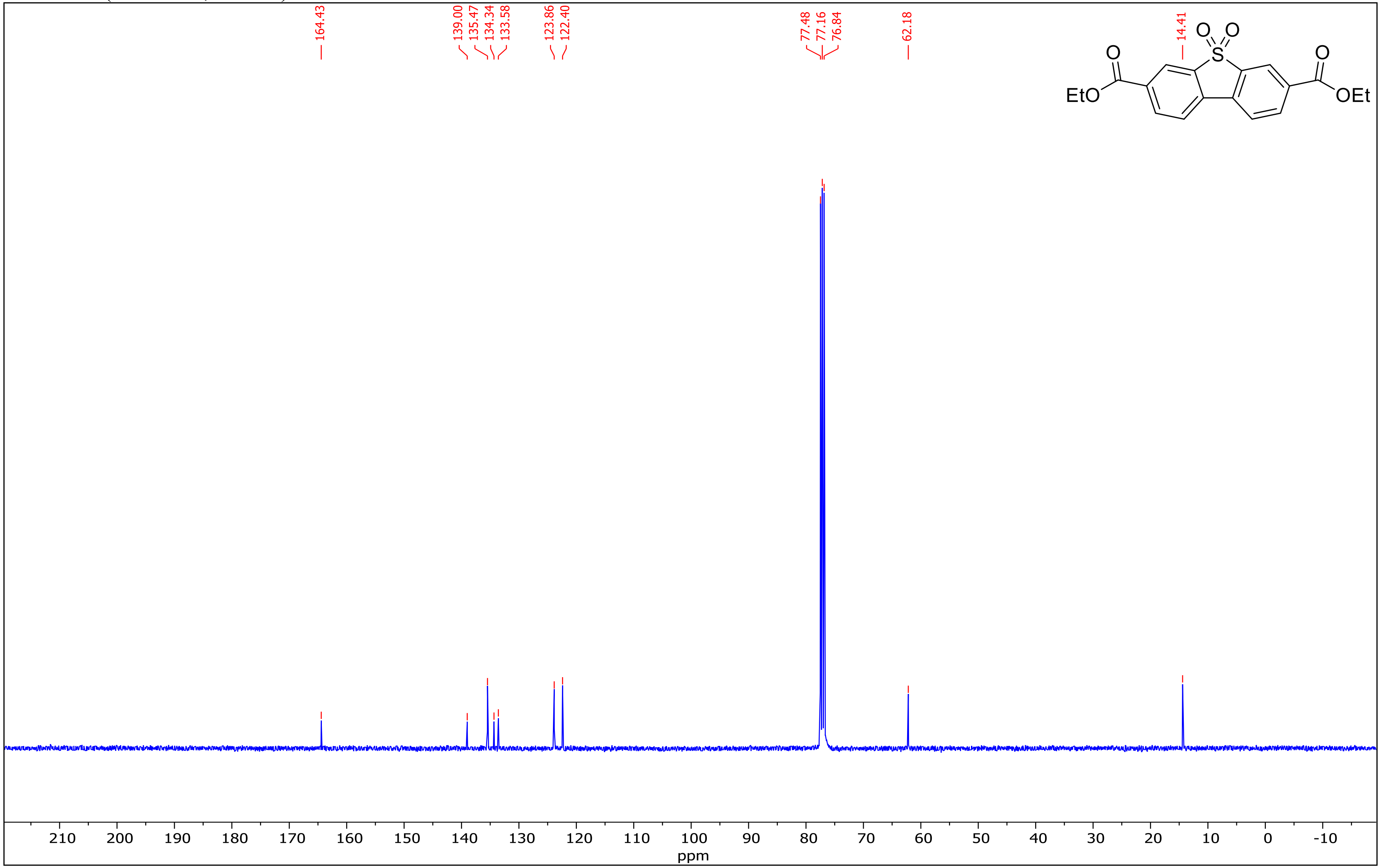

1m ^{1}H NMR (400 MHz, $CDCl_3$)

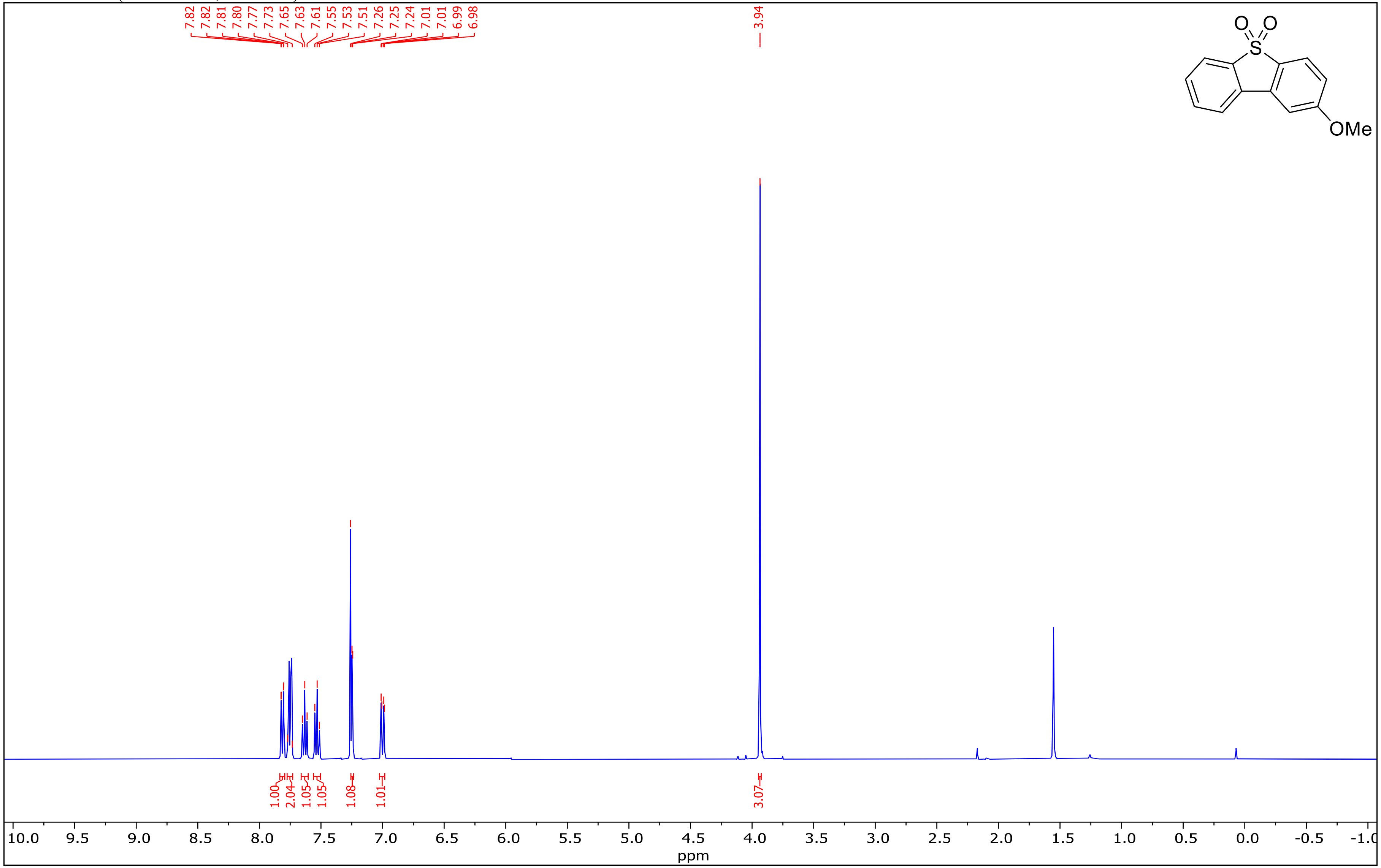

1m ^{13}C NMR (101 MHz, $CDCl_3$)

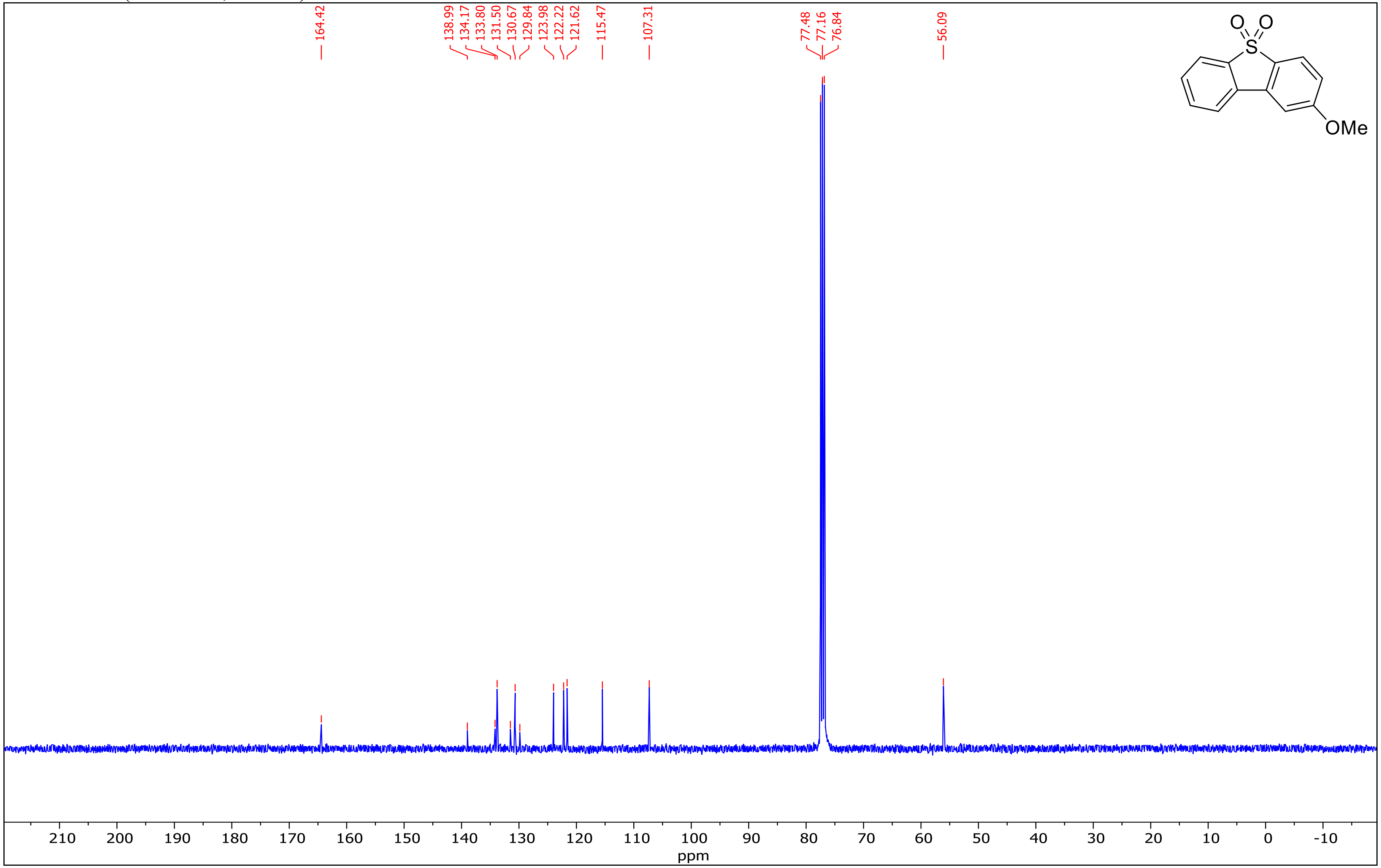

1n ^{1}H NMR (400 MHz, $CDCl_3$)

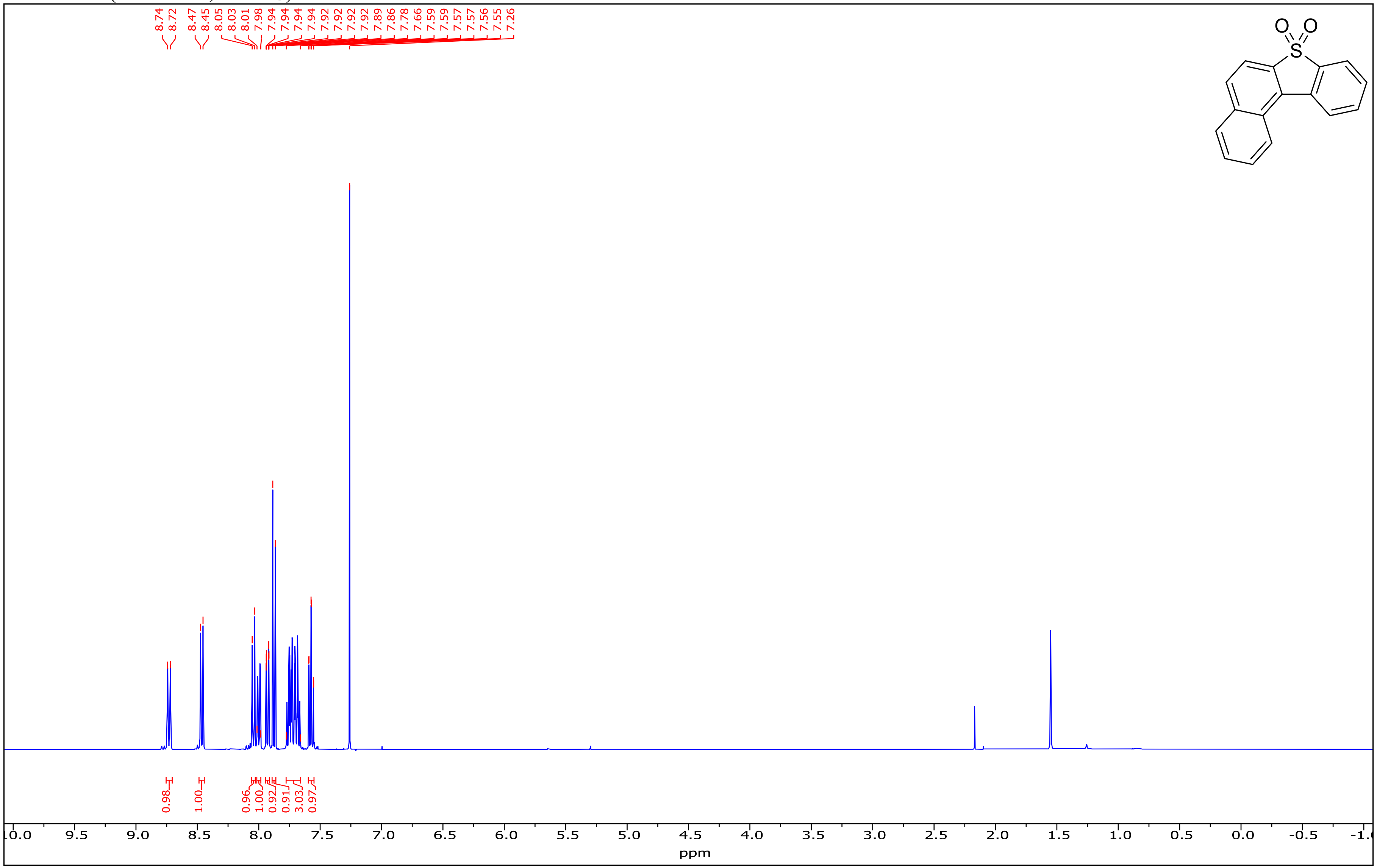

1n ^{13}C NMR (101 MHz, $CDCl_3$)

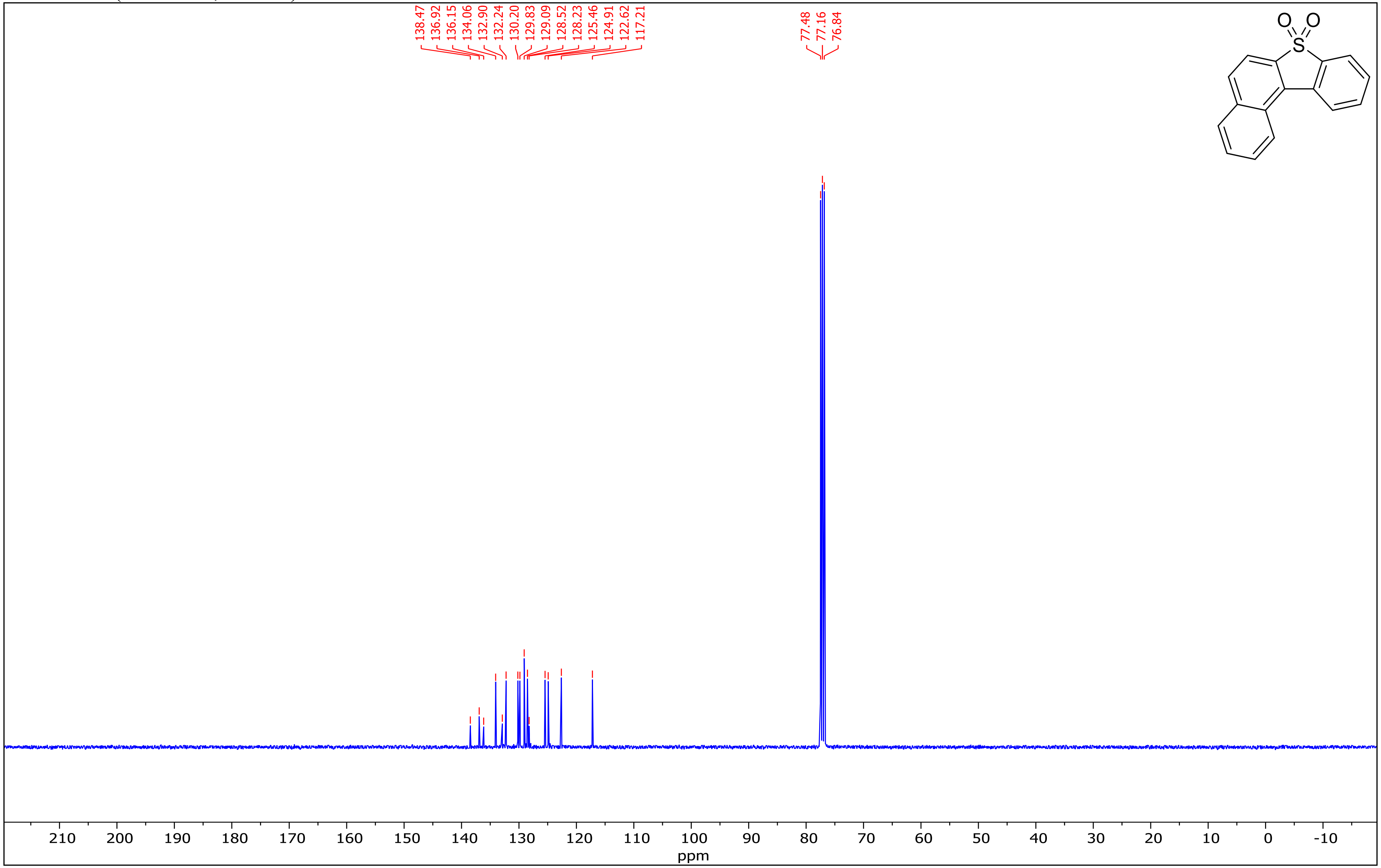

1o ^{1}H NMR (400 MHz, $CDCl_3$)

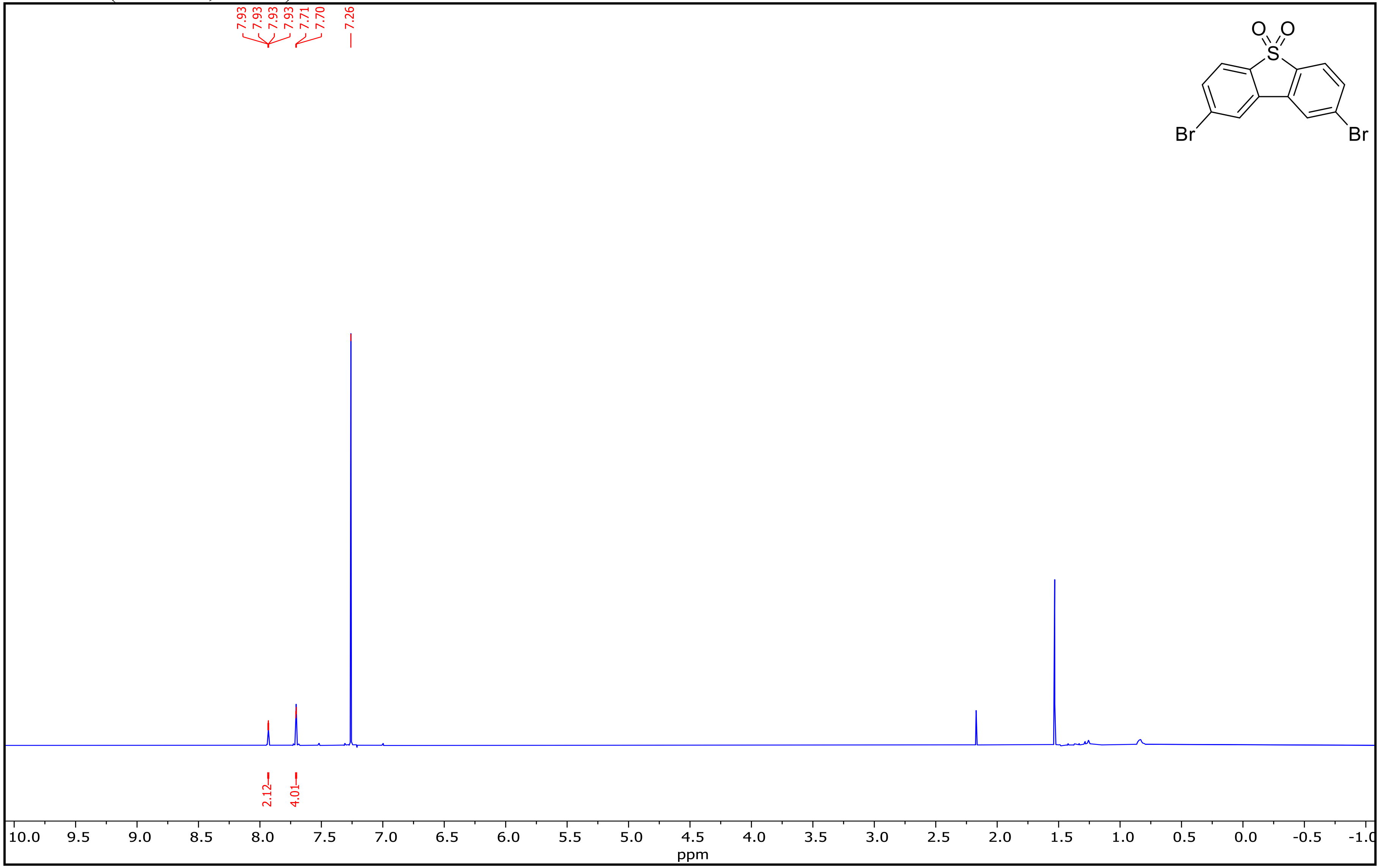

1o ^{13}C NMR (101 MHz, $CDCl_3$)

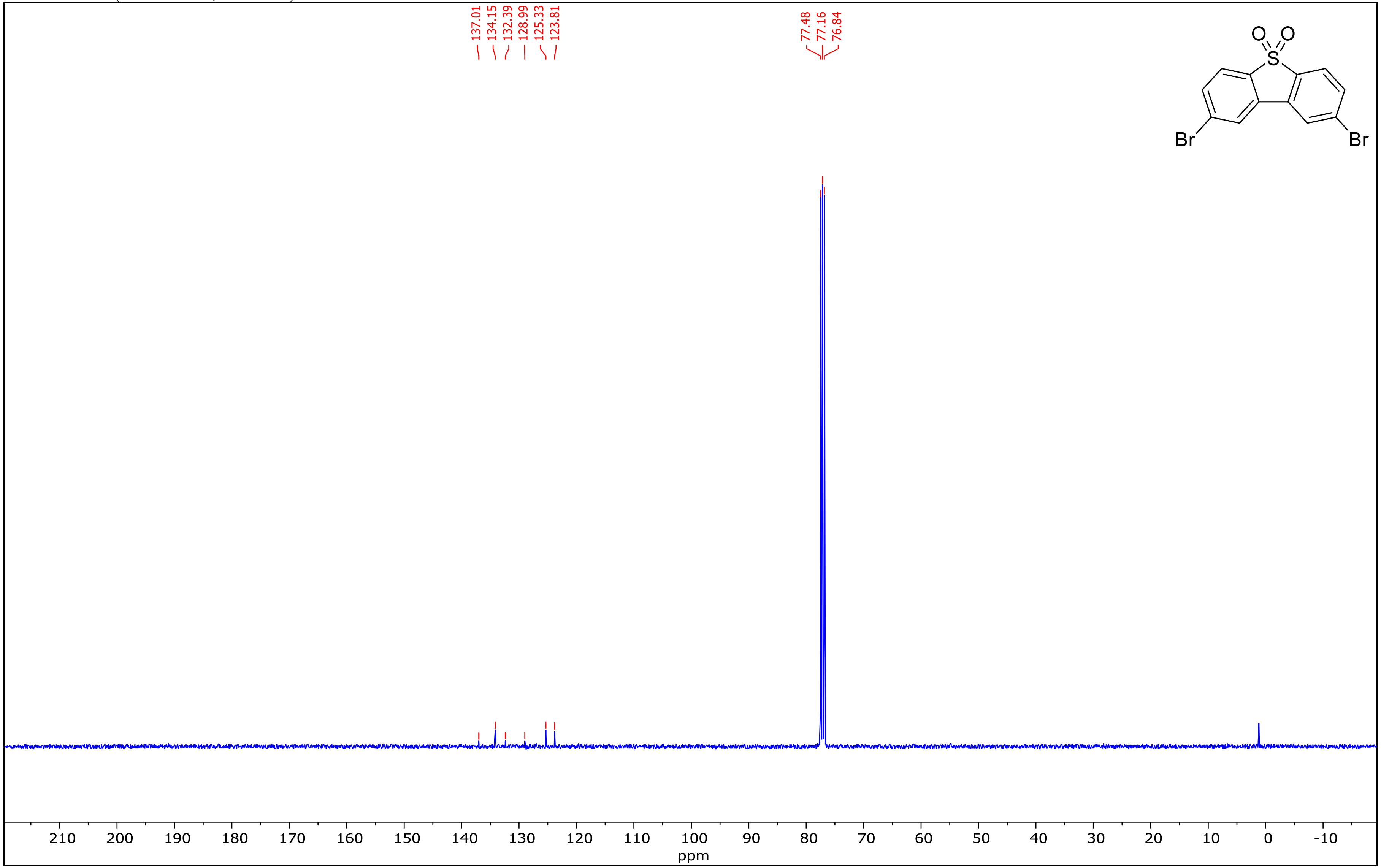

1p ^{1}H NMR (400 MHz, $CDCl_3$)

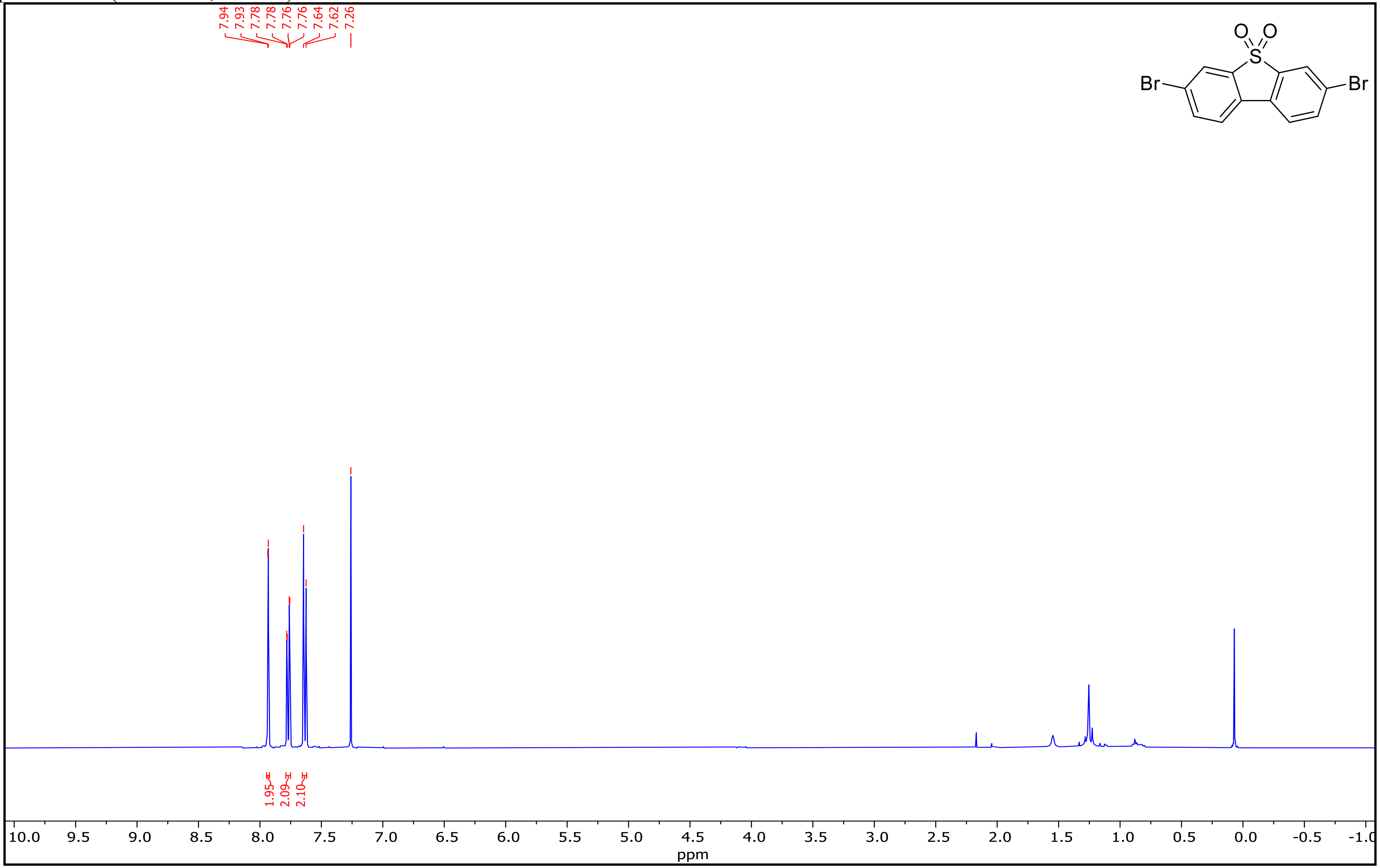

1p ^{13}C NMR (101 MHz, CDCl$_3$)

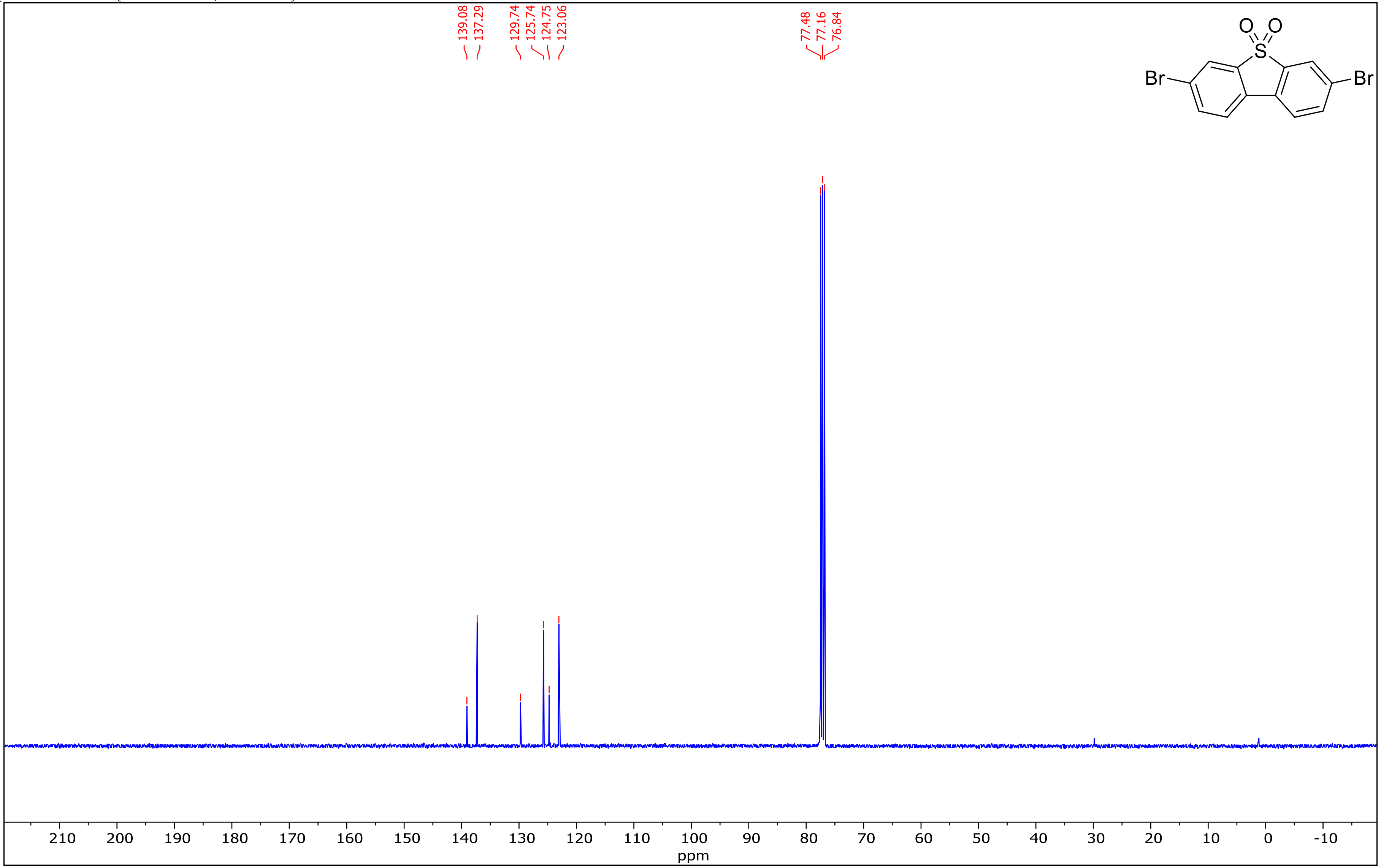

1q ^{1}H NMR (400 MHz, DMSO-d$_6$)

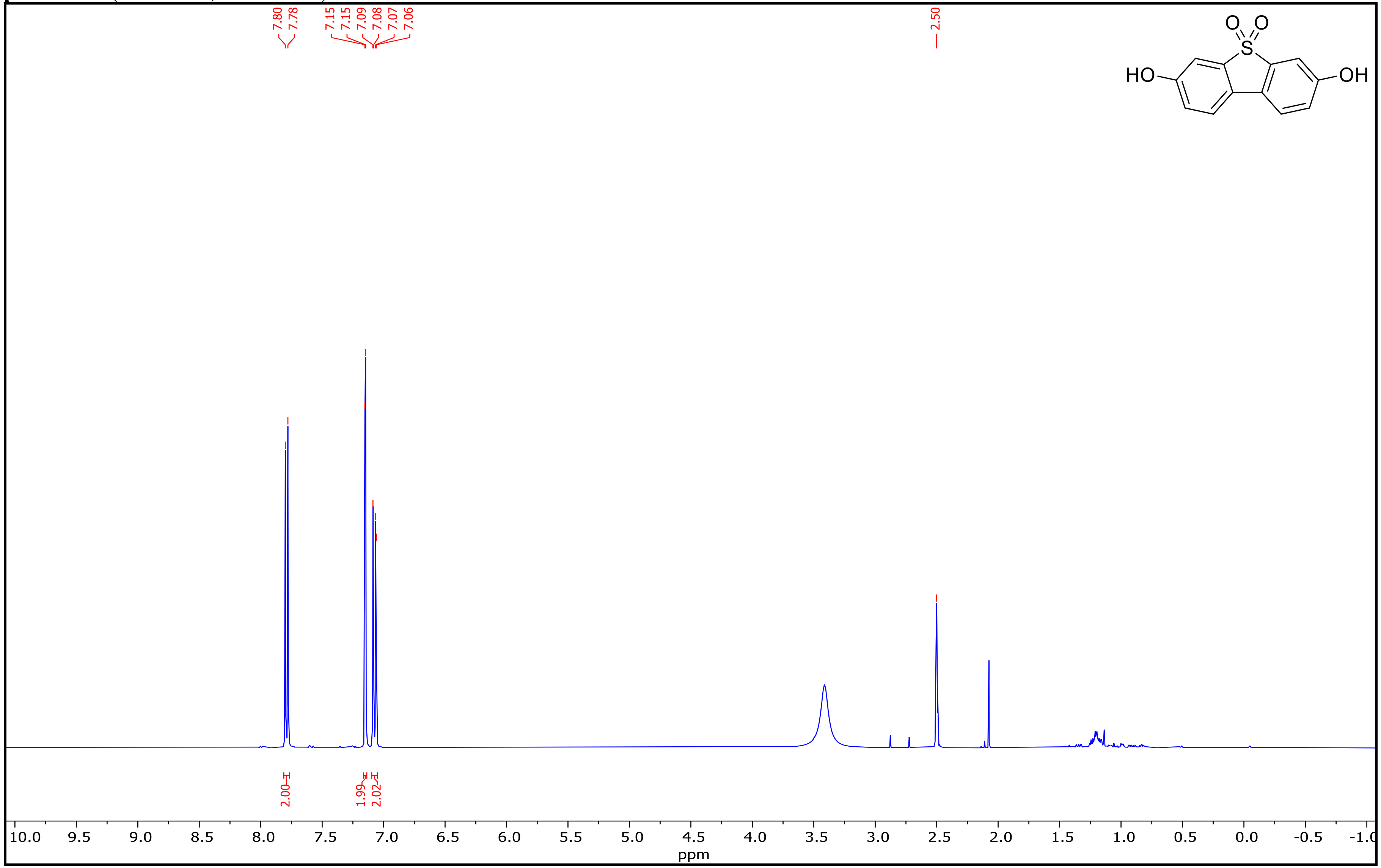

1q ^{13}C NMR (101 MHz, DMSO-d_6)

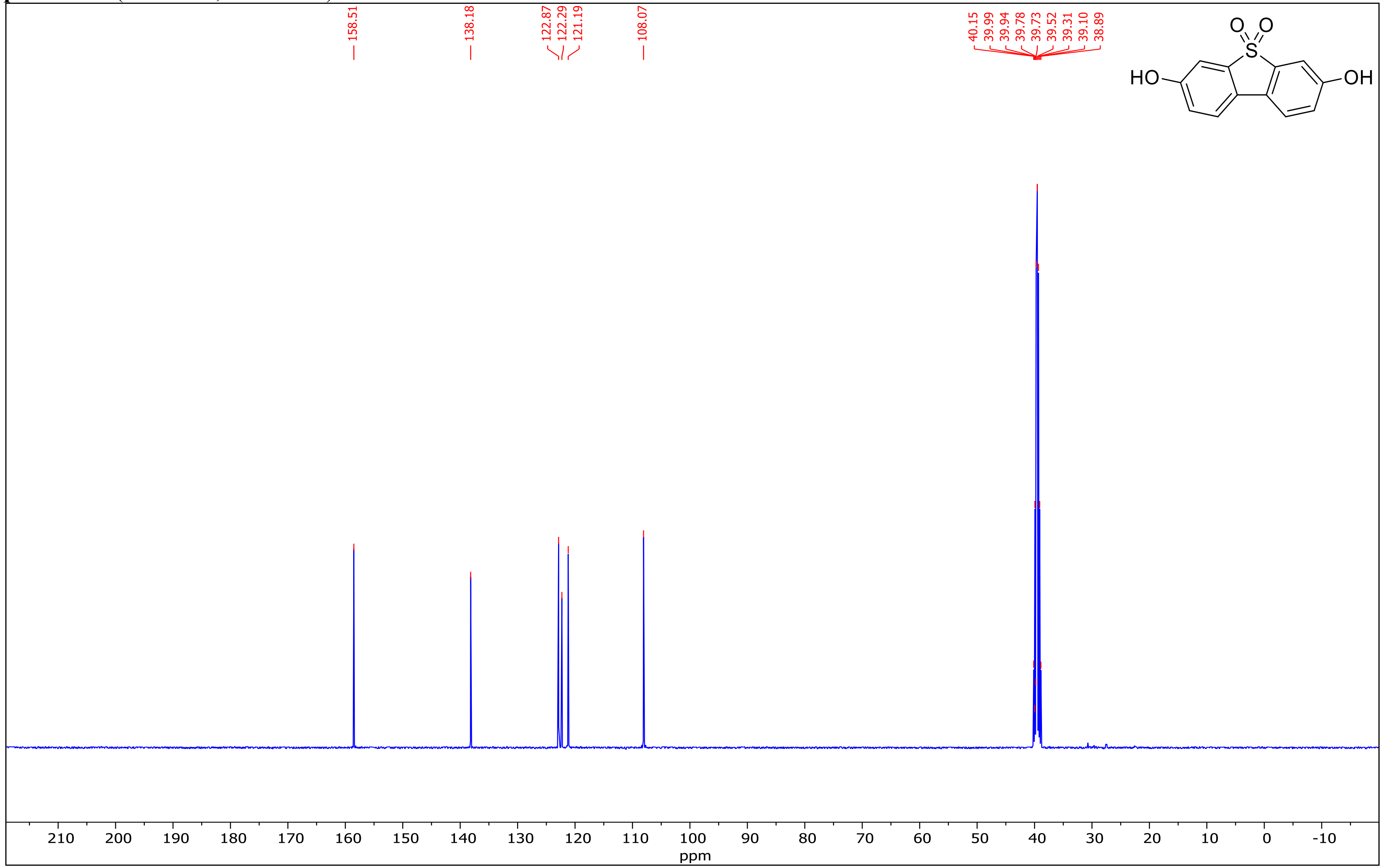

1b’ ^{1}H NMR (400 MHz, $CDCl_3$)

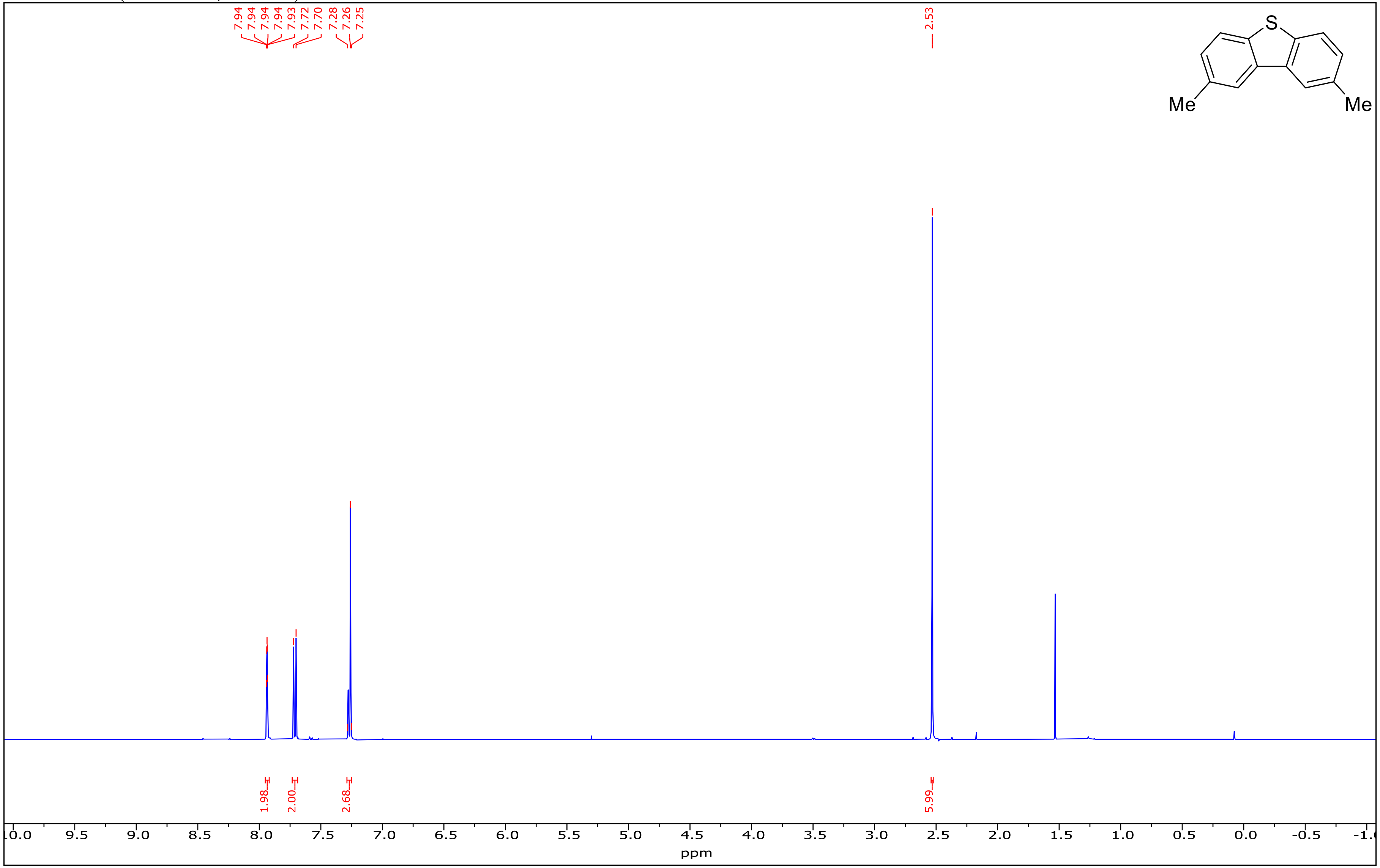

1b' ^{13}C NMR (101 MHz, $CDCl_3$)

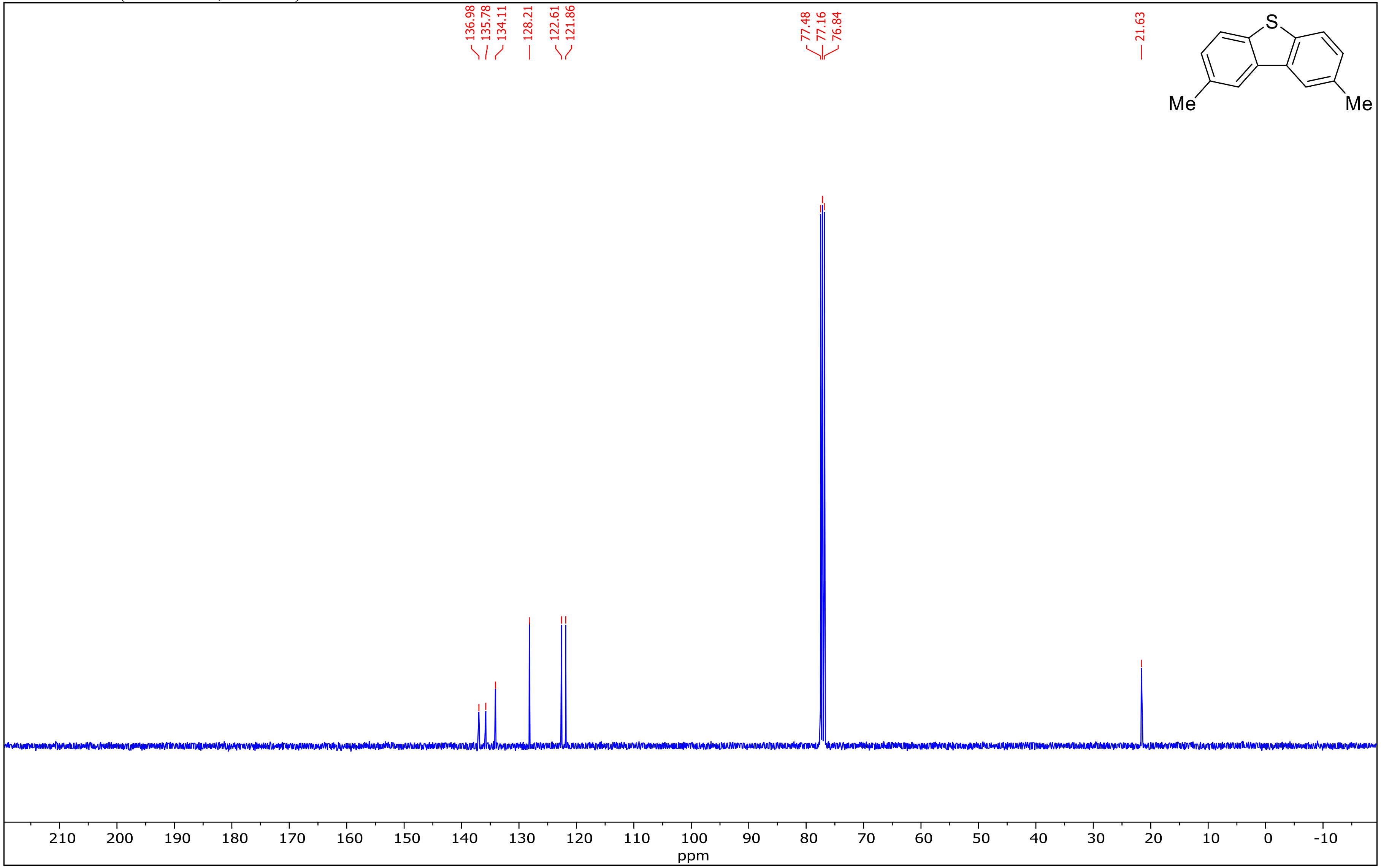

1d' ^{1}H NMR (400 MHz, $CDCl_3$)

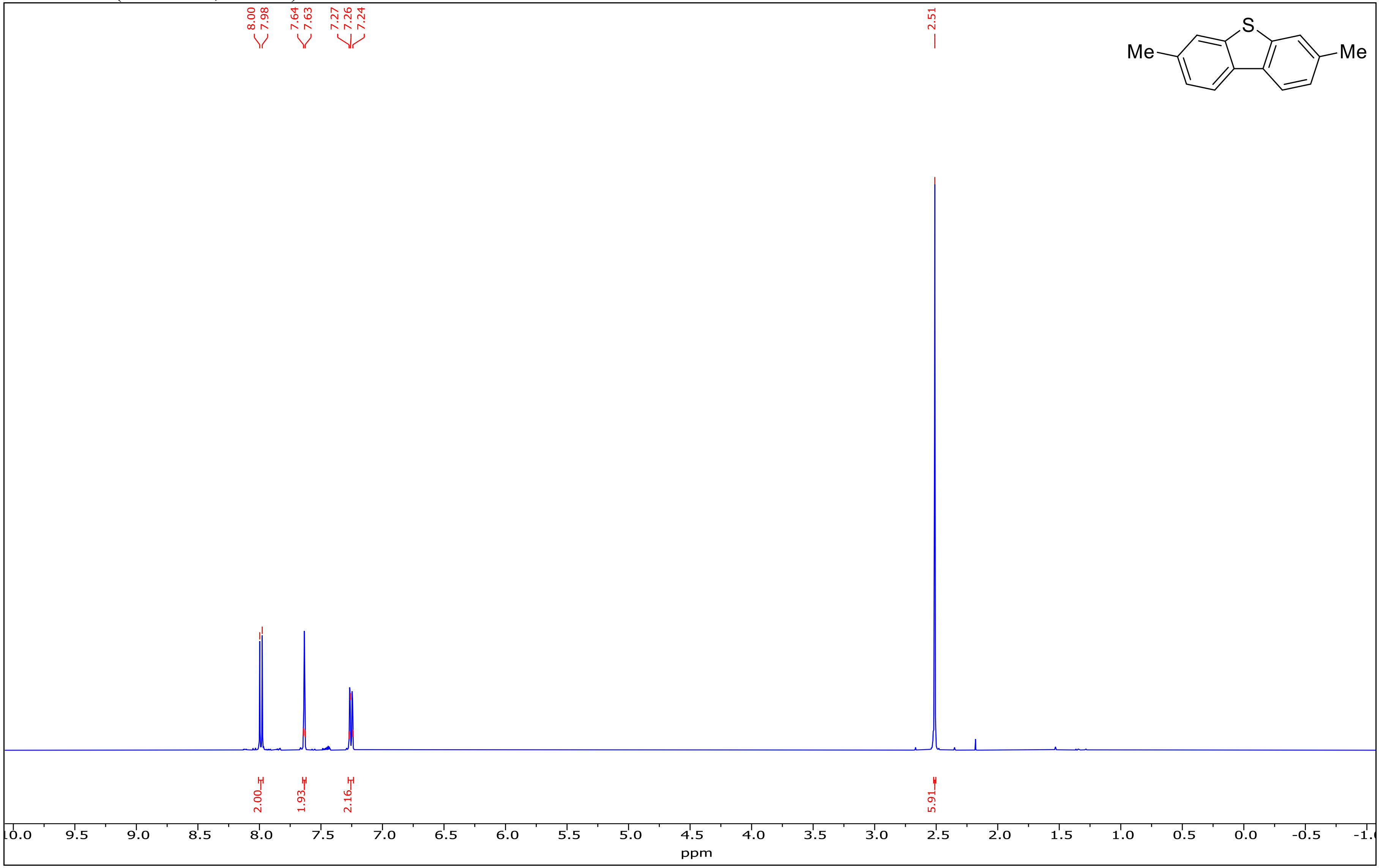

1d' ^{13}C NMR (101 MHz, $CDCl_3$)

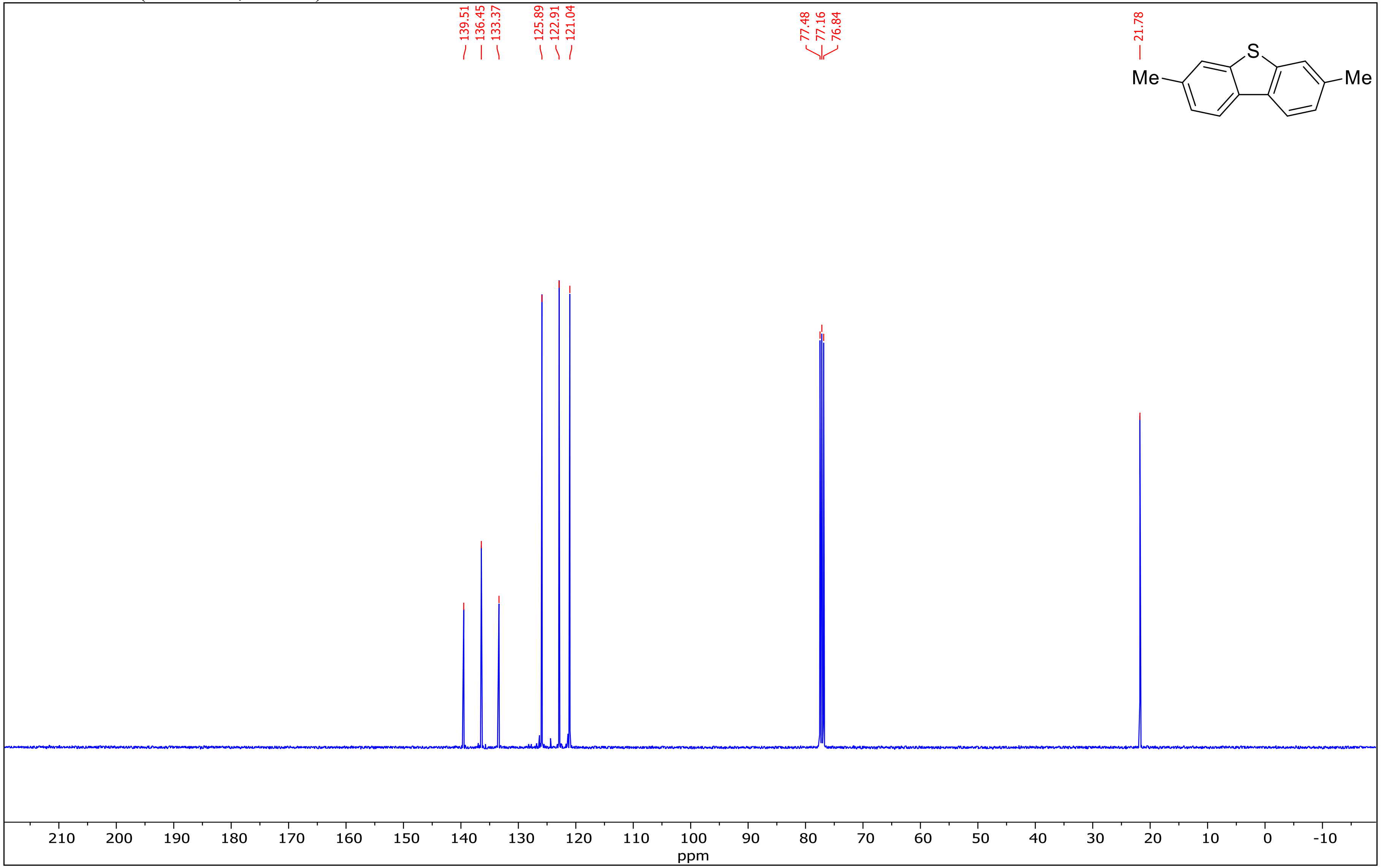

1e' ^{1}H NMR (400 MHz, $CDCl_3$)

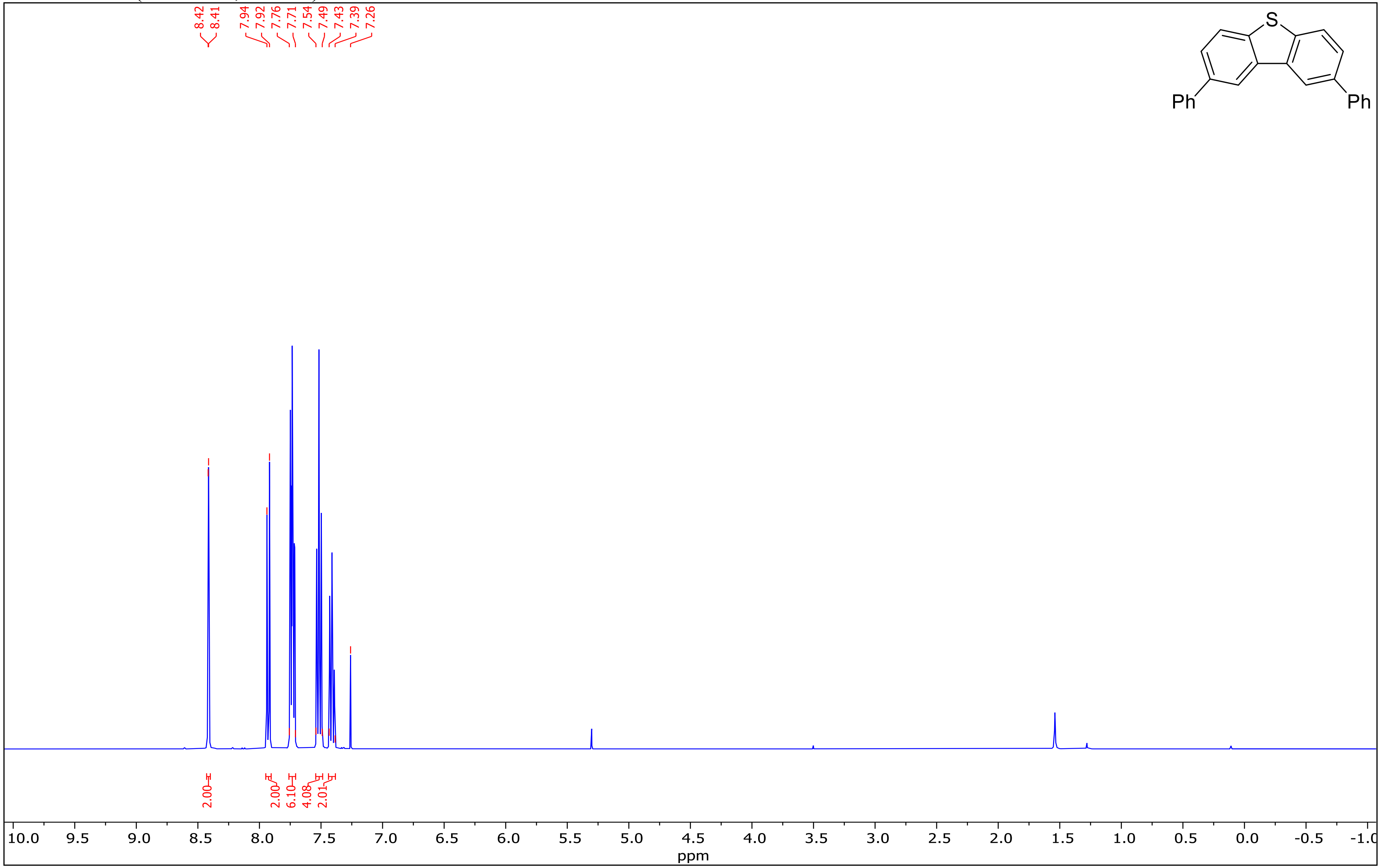

1e' ^{13}C NMR (101 MHz, $CDCl_3$)

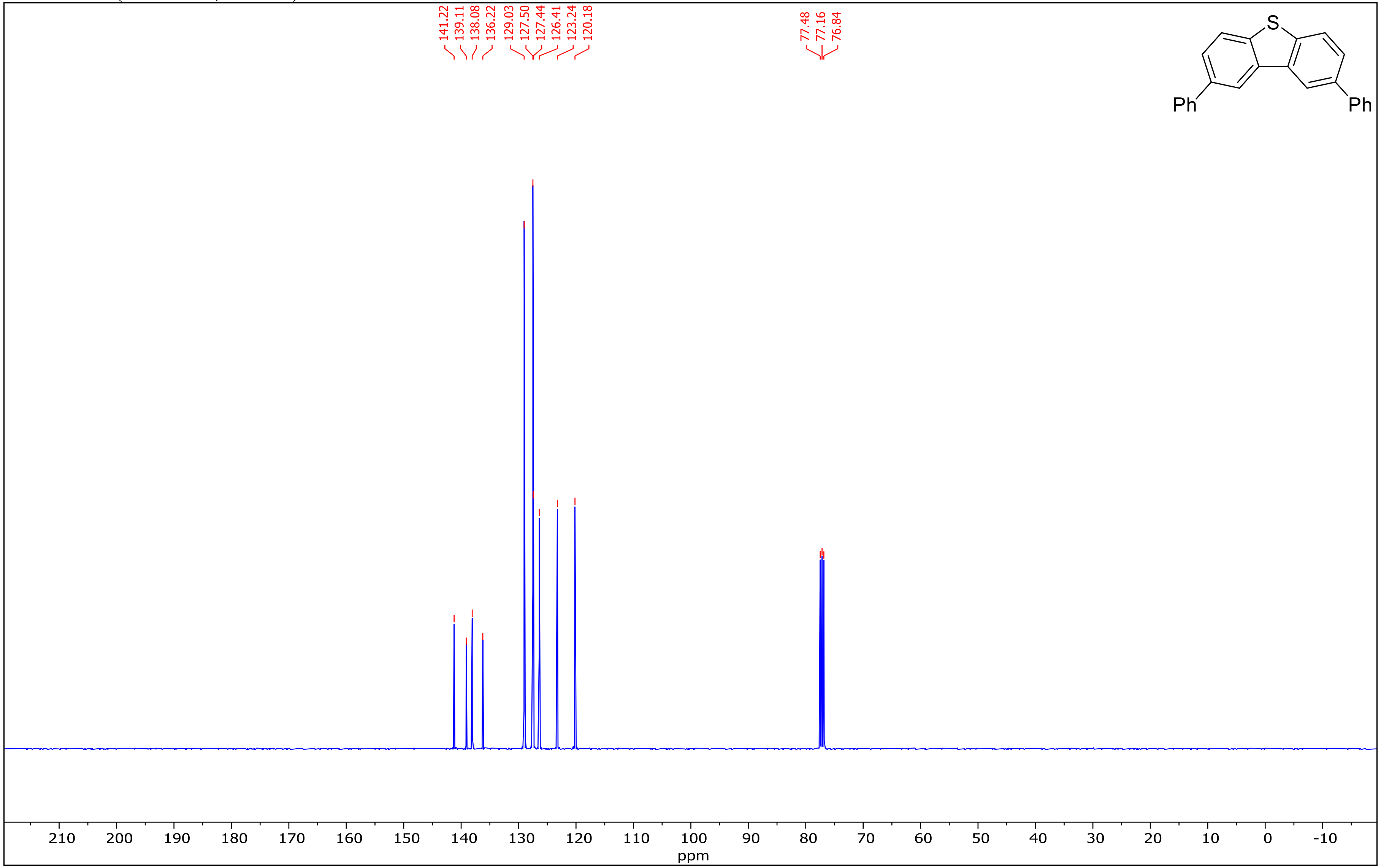

1f' ^{1}H NMR (400 MHz, $CDCl_3$)

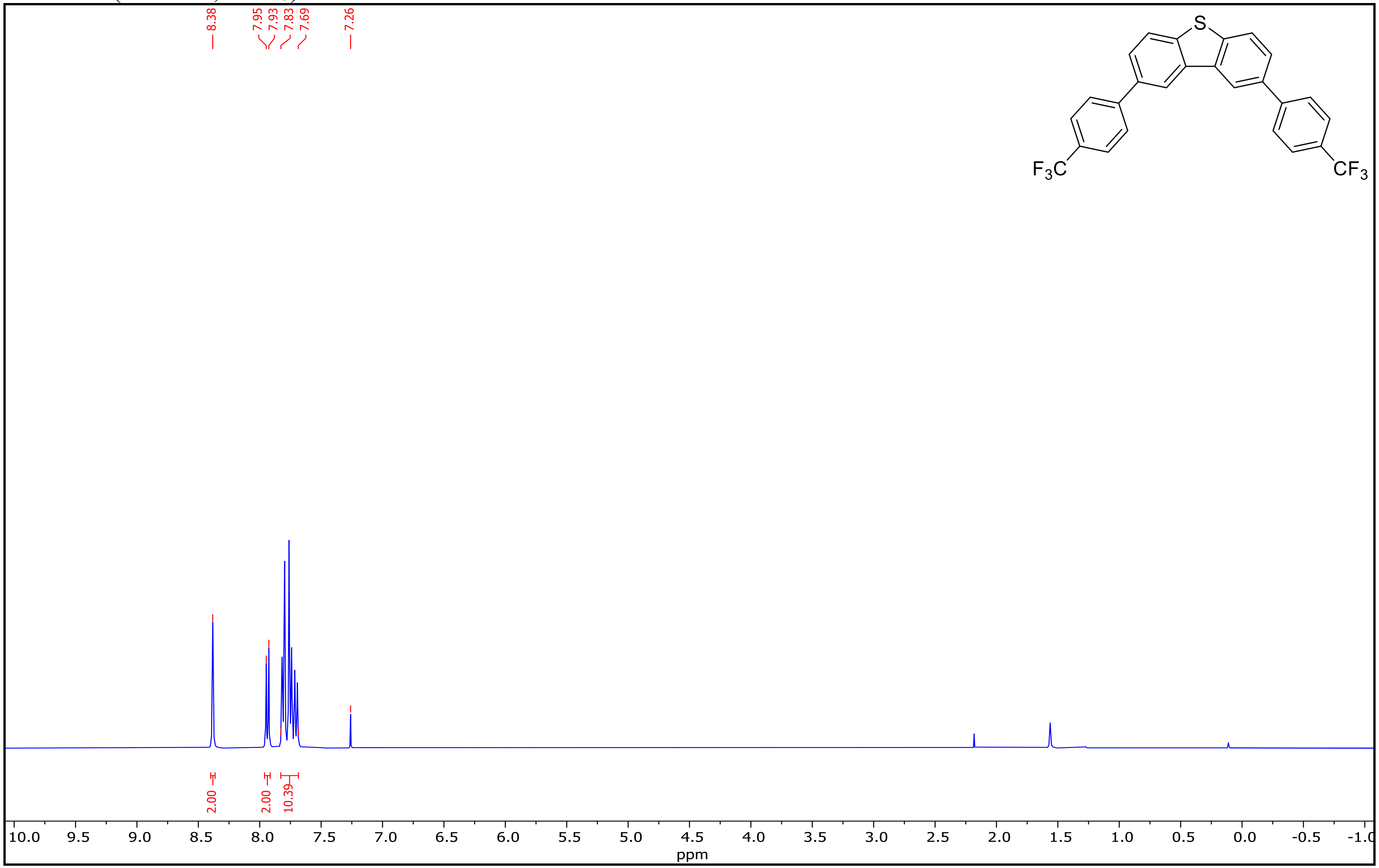

1f’ ^{13}C NMR (101 MHz, $CDCl_3$)

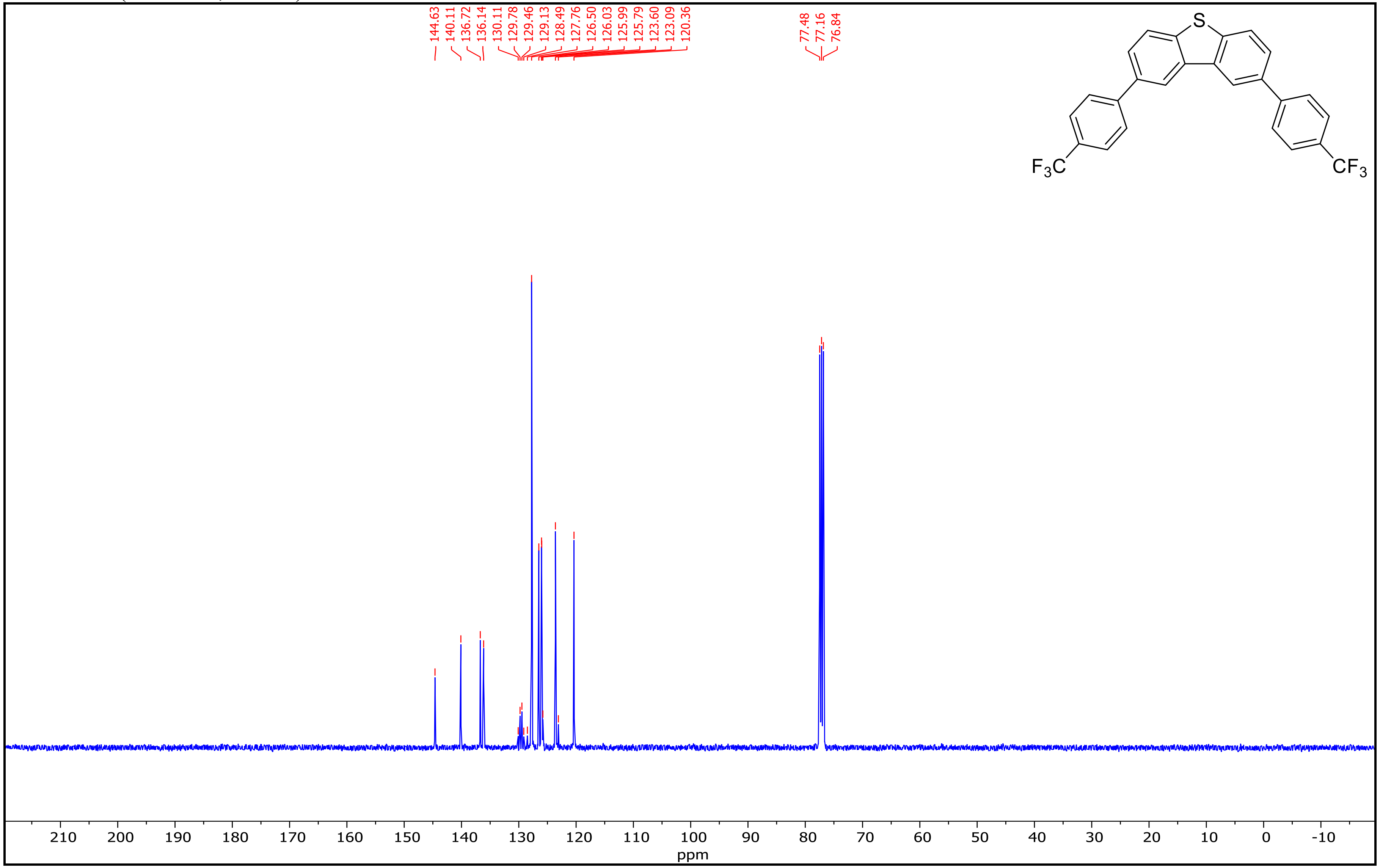

1f' ^{19}F NMR (376 MHz, $CDCl_3$)

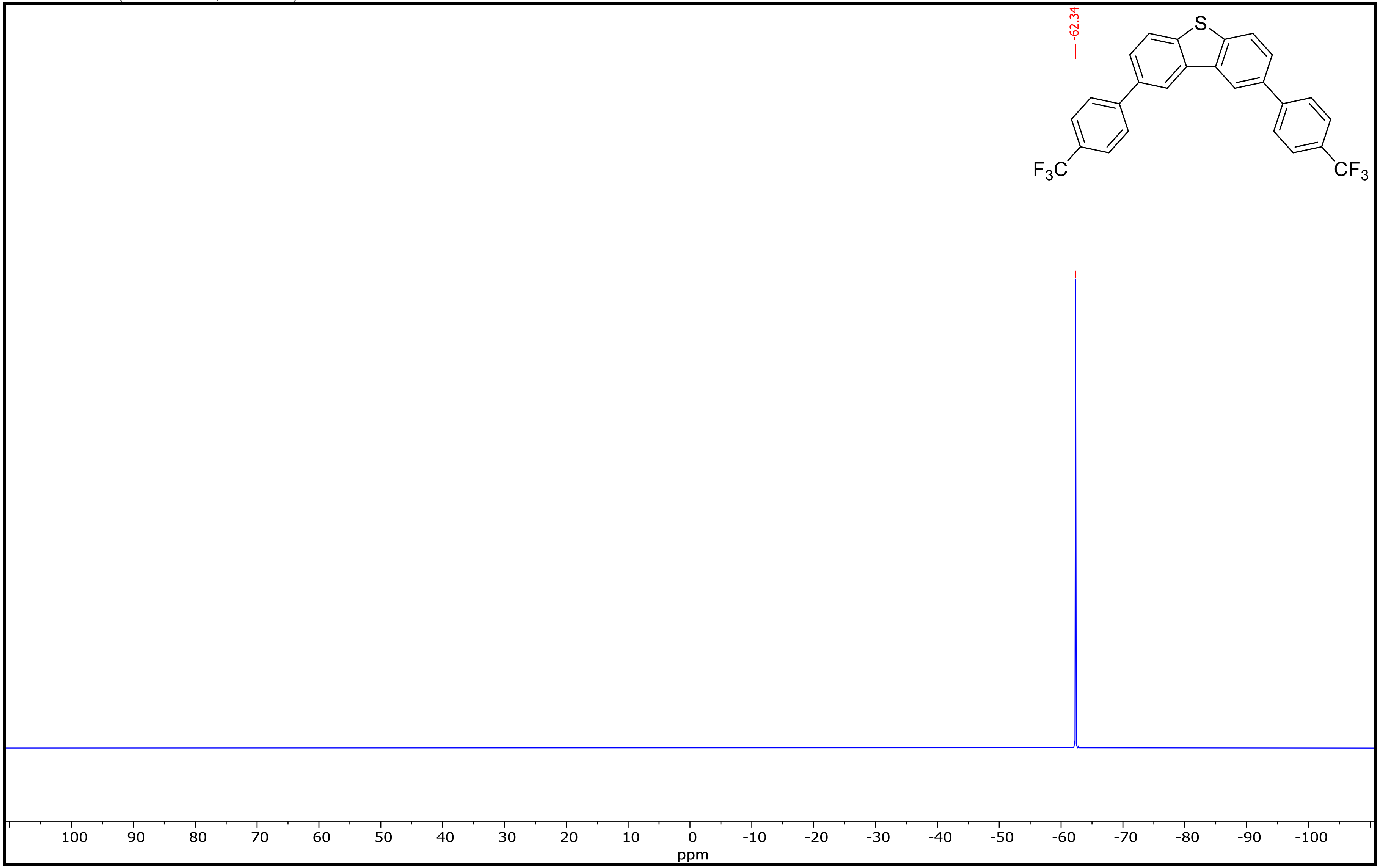

1g’ ^{1}H NMR (400 MHz, $CDCl_3$)

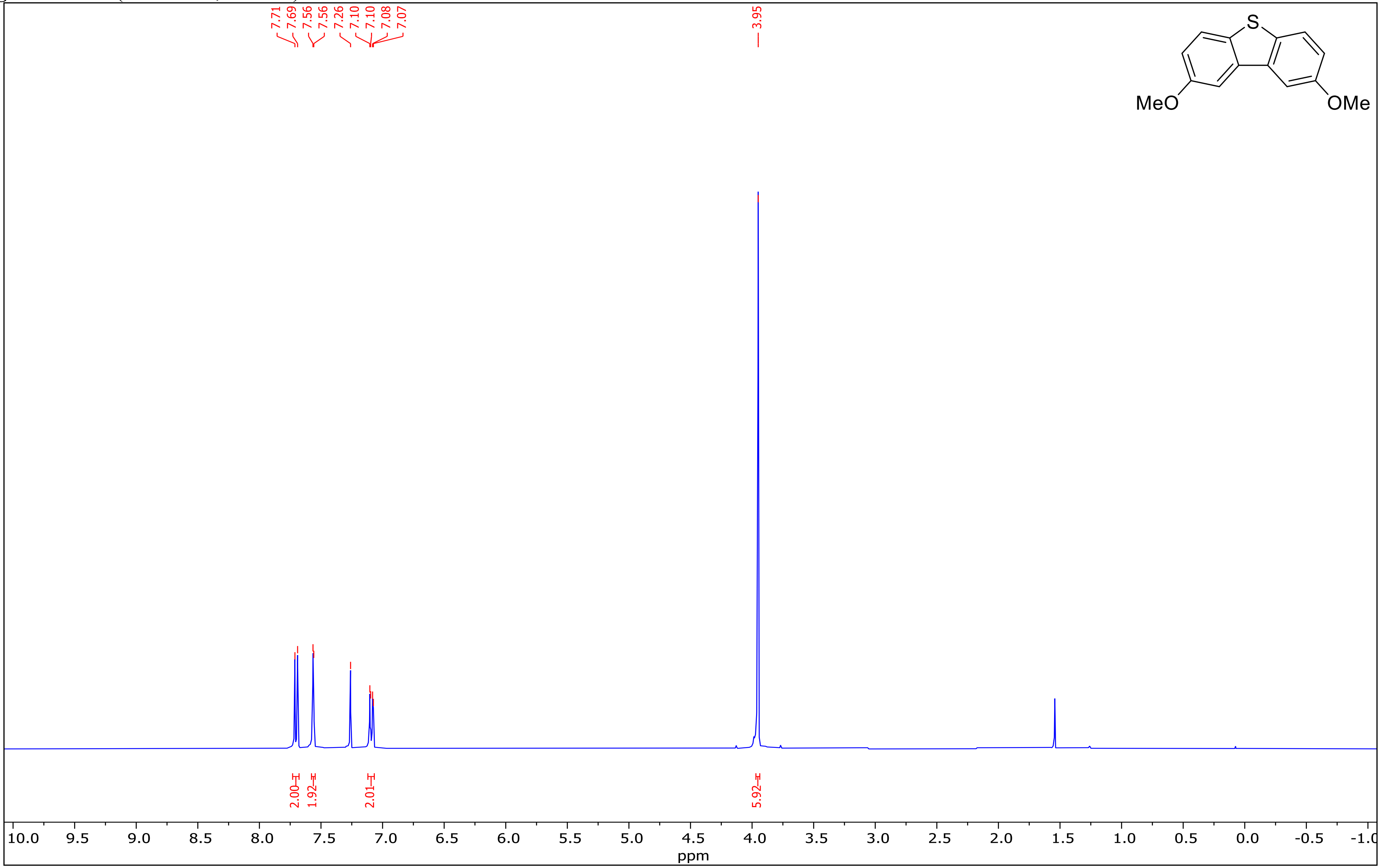

1g' ^{13}C NMR (101 MHz, $CDCl_3$)

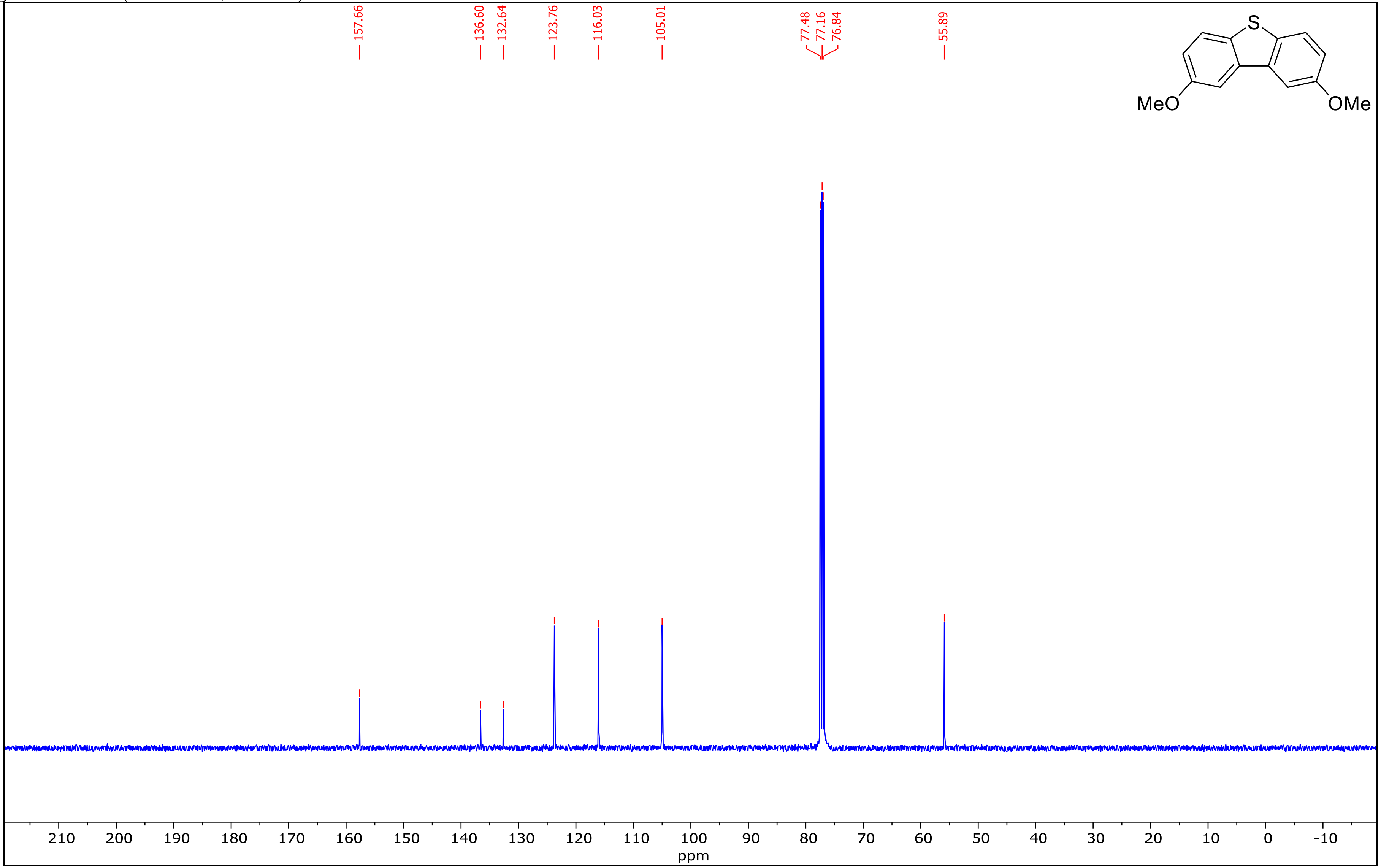

1i' ^{1}H NMR (400 MHz, $CDCl_3$)

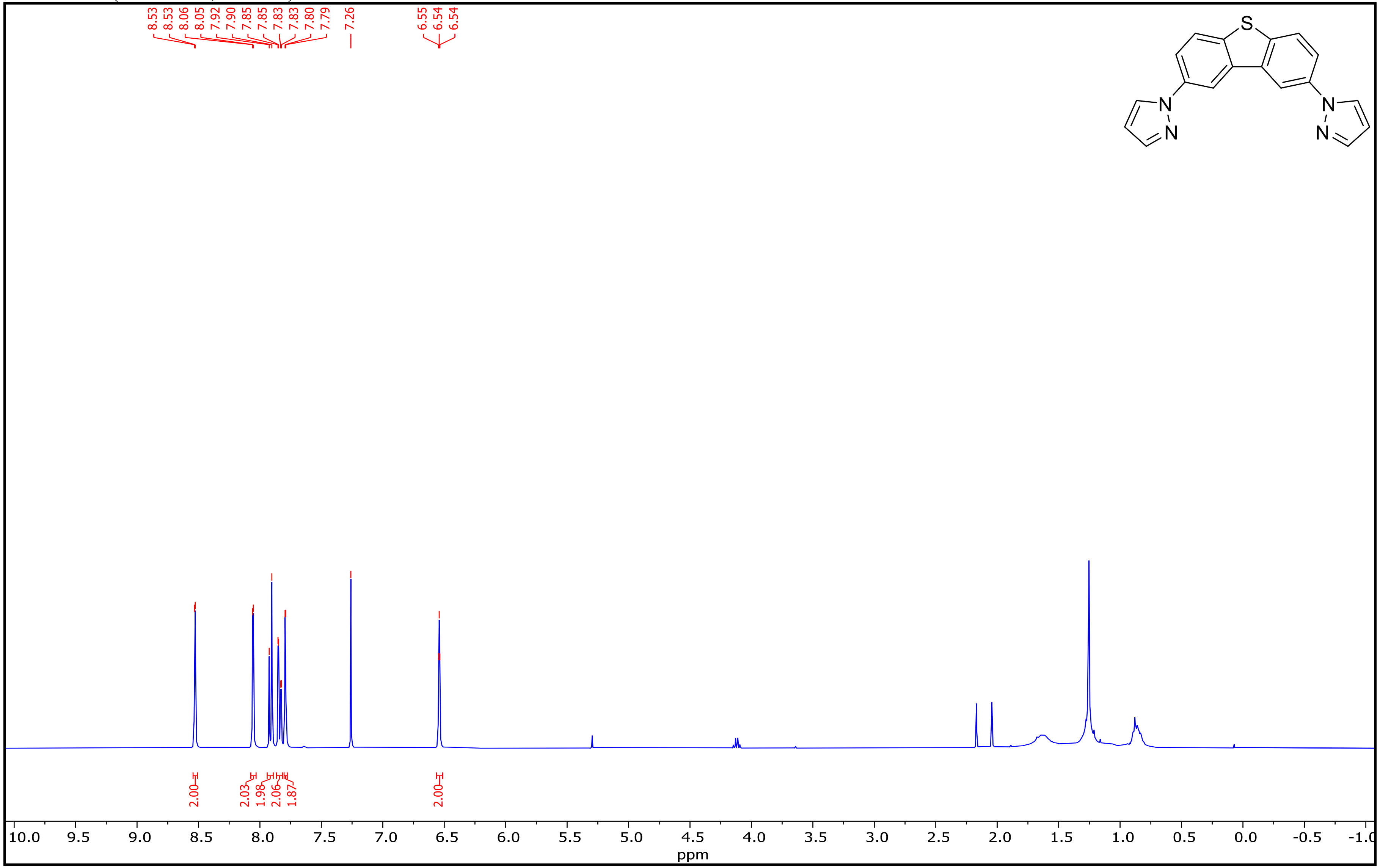

1i' ^{13}C NMR (101 MHz, $CDCl_3$)

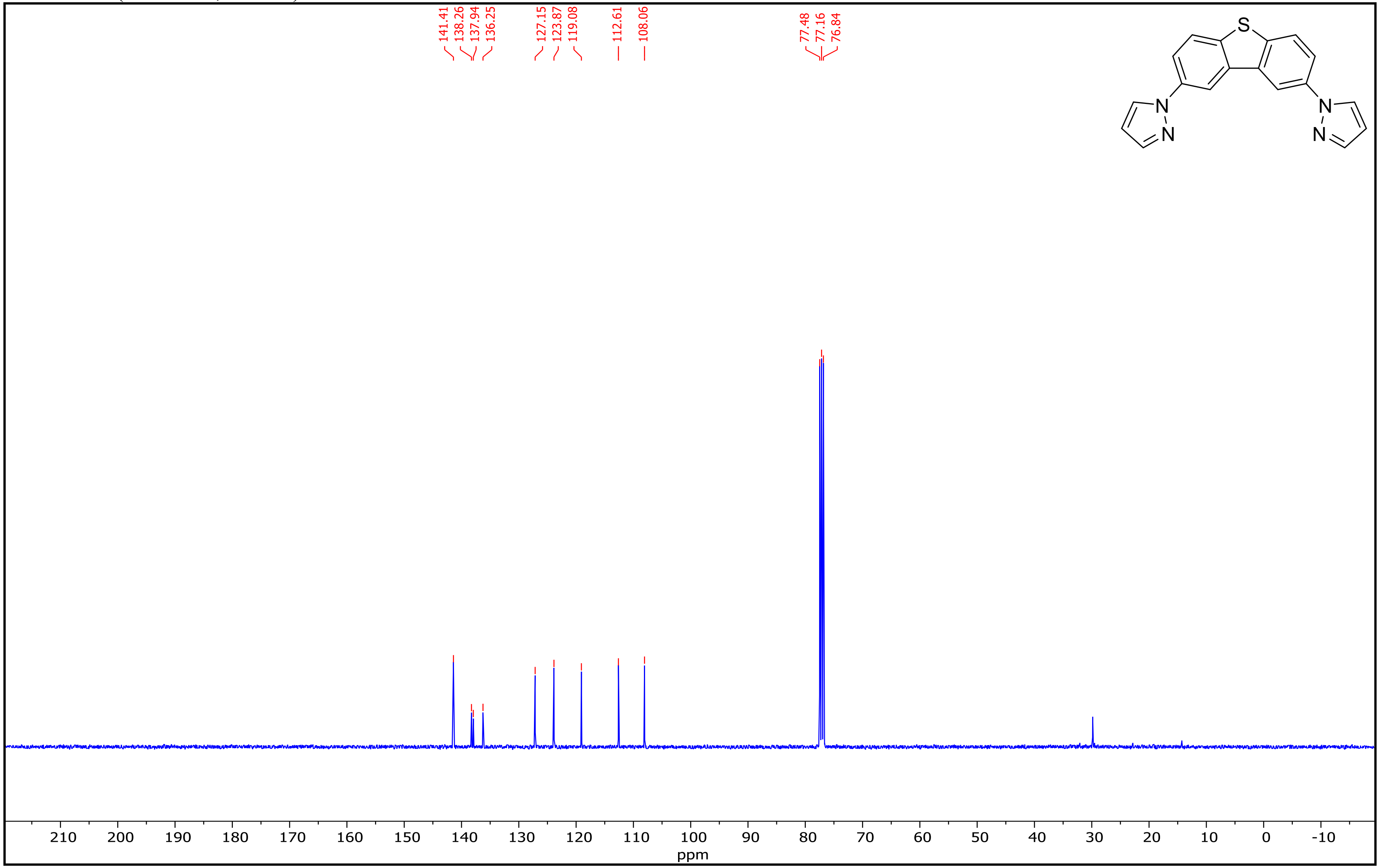

1k' ^{1}H NMR (400 MHz, $CDCl_3$)

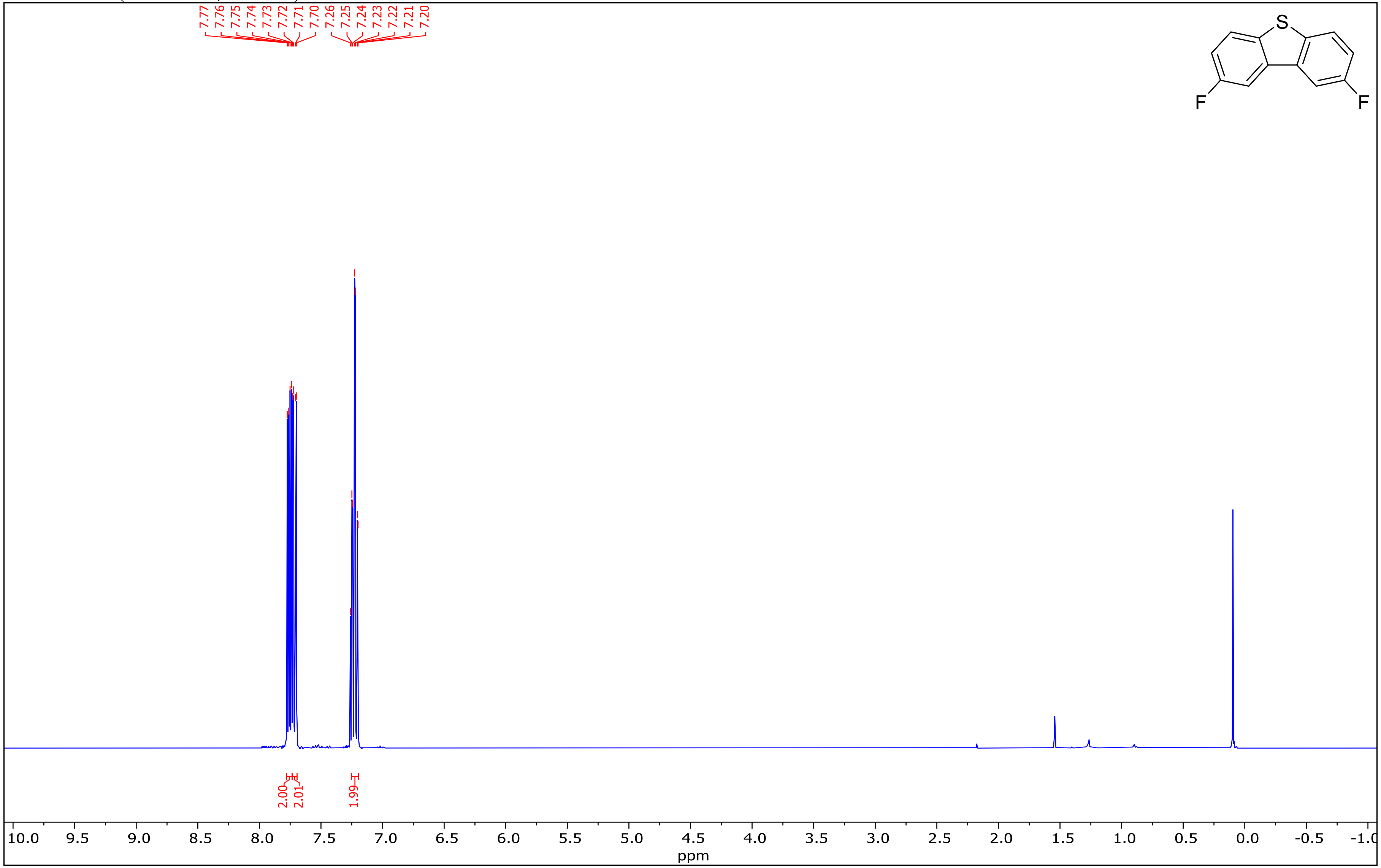

1k' ^{13}C NMR (101 MHz, $CDCl_3$)

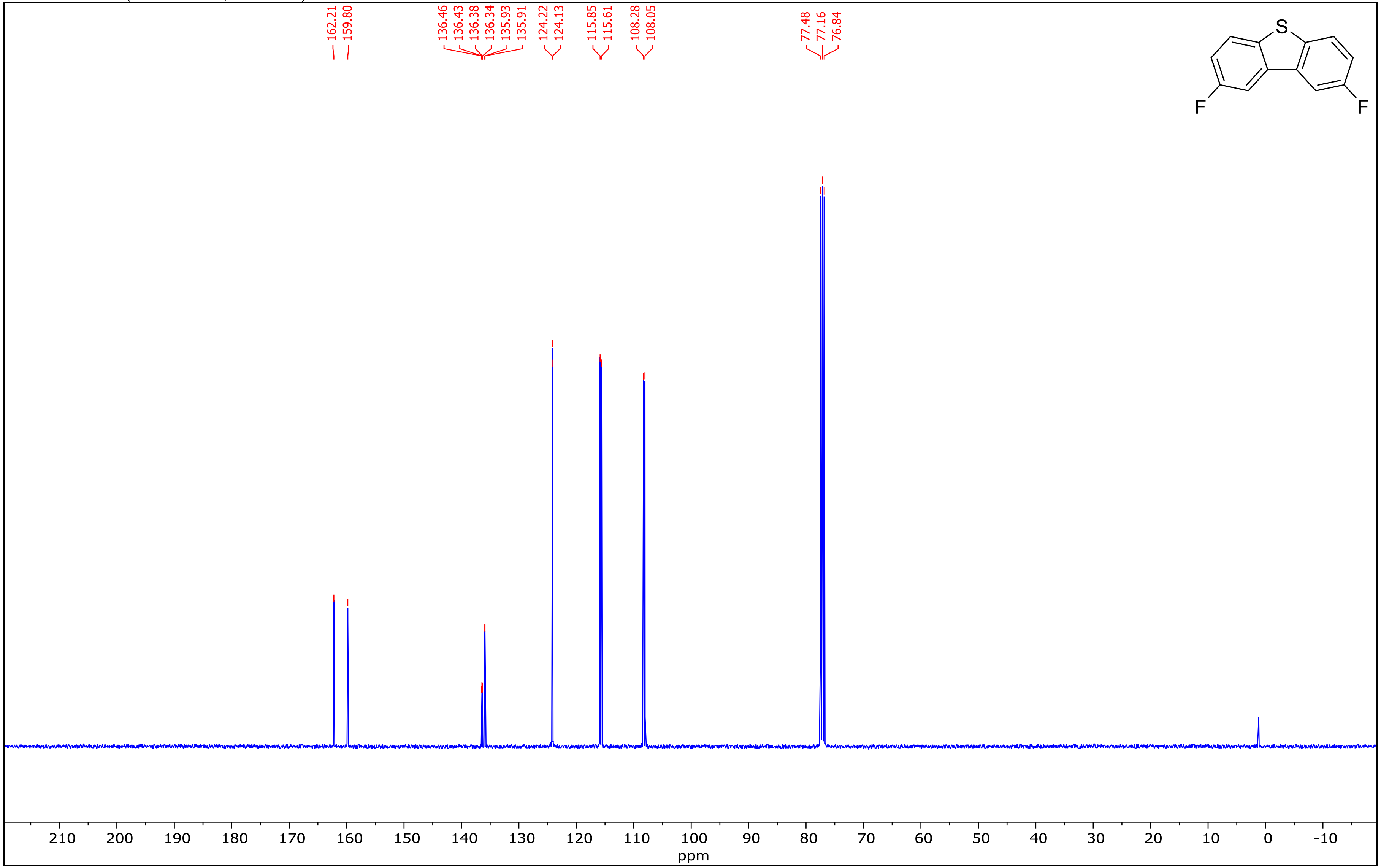

1k’ ^{19}F NMR (376 MHz, $CDCl_3$)

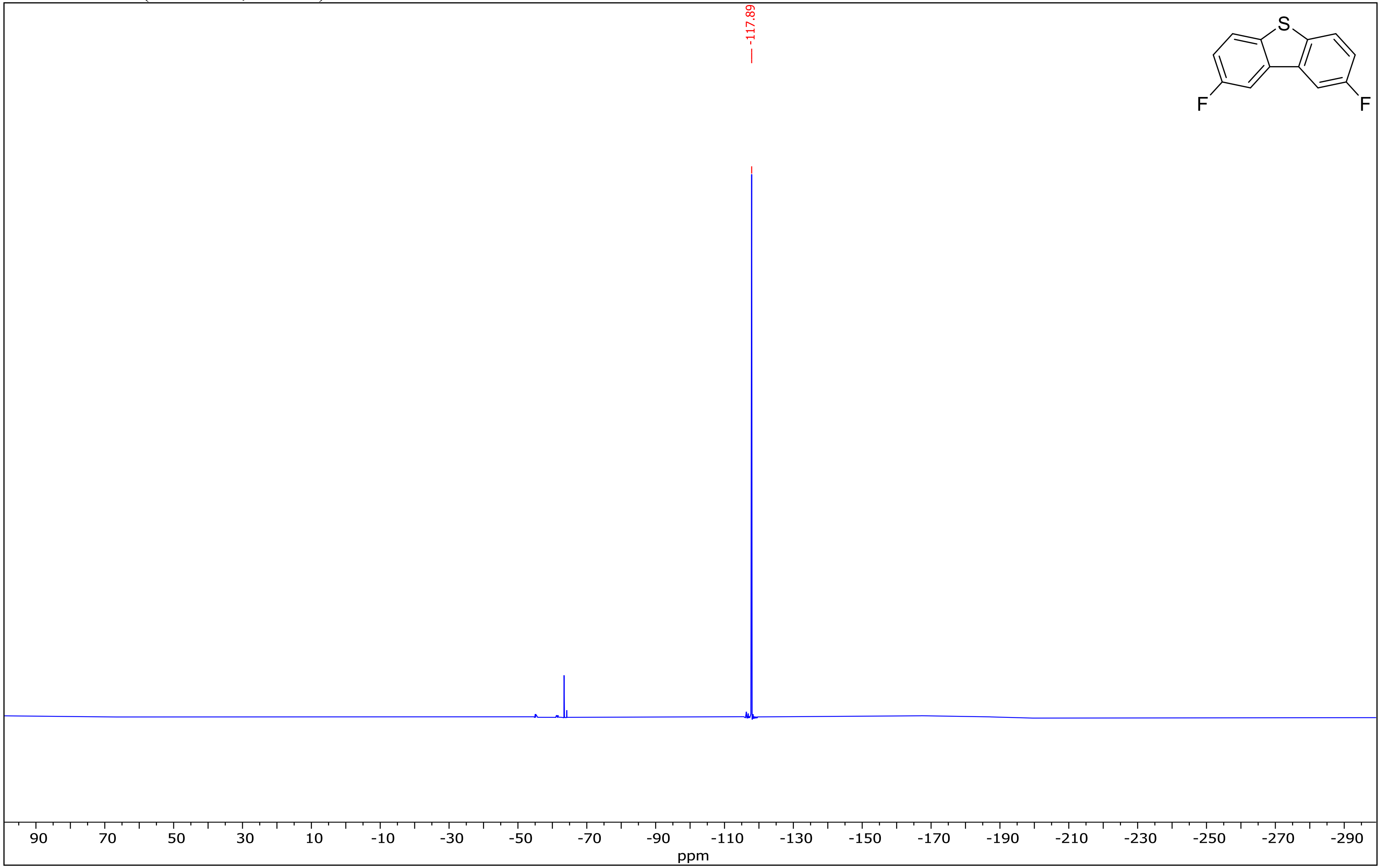

1l' ^{1}H NMR (400 MHz, DMSO-d$_6$)

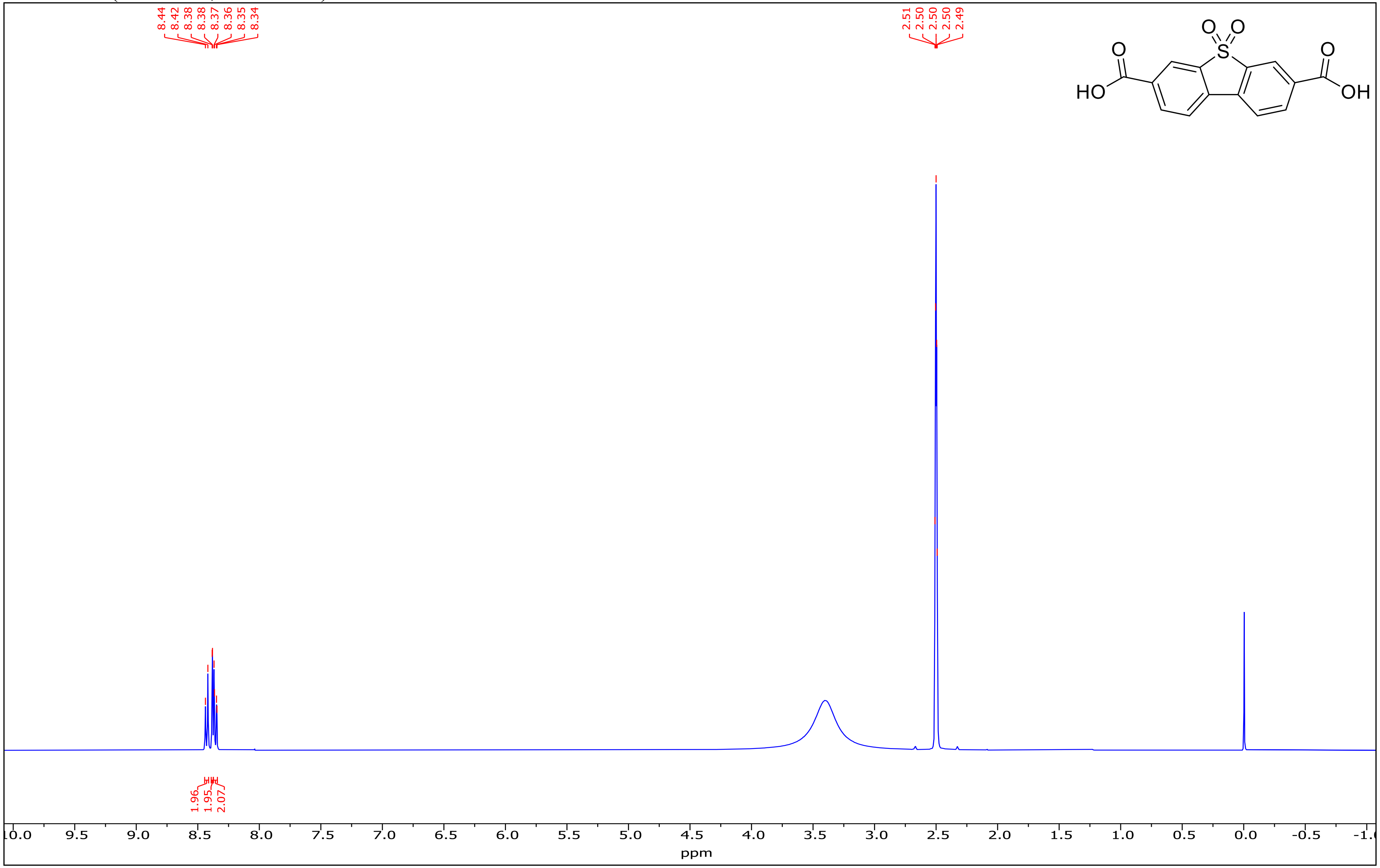

1l' ^{13}C NMR (101 MHz, DMSO-d$_6$)

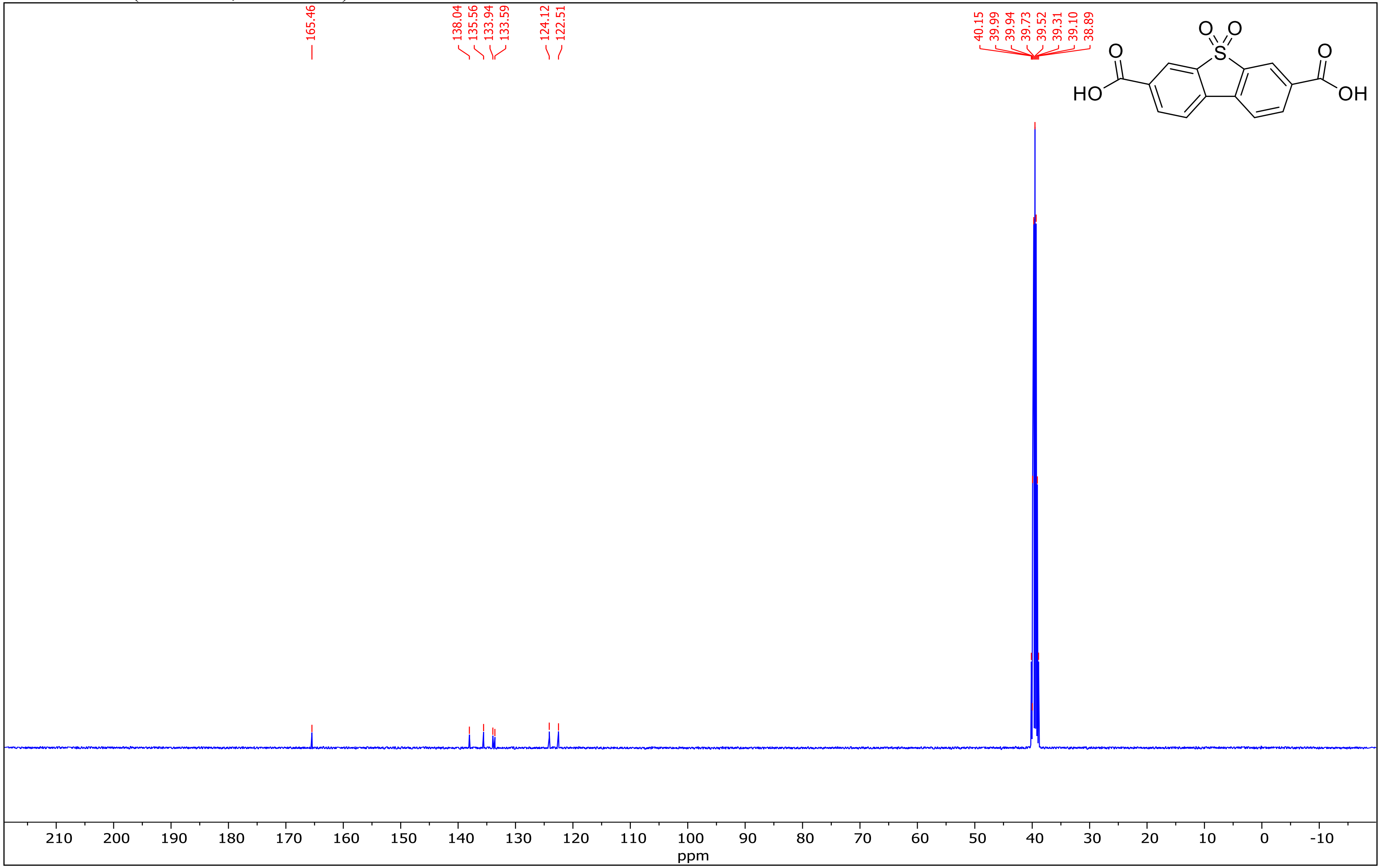

1m' ^{1}H NMR (400 MHz, $CDCl_3$)

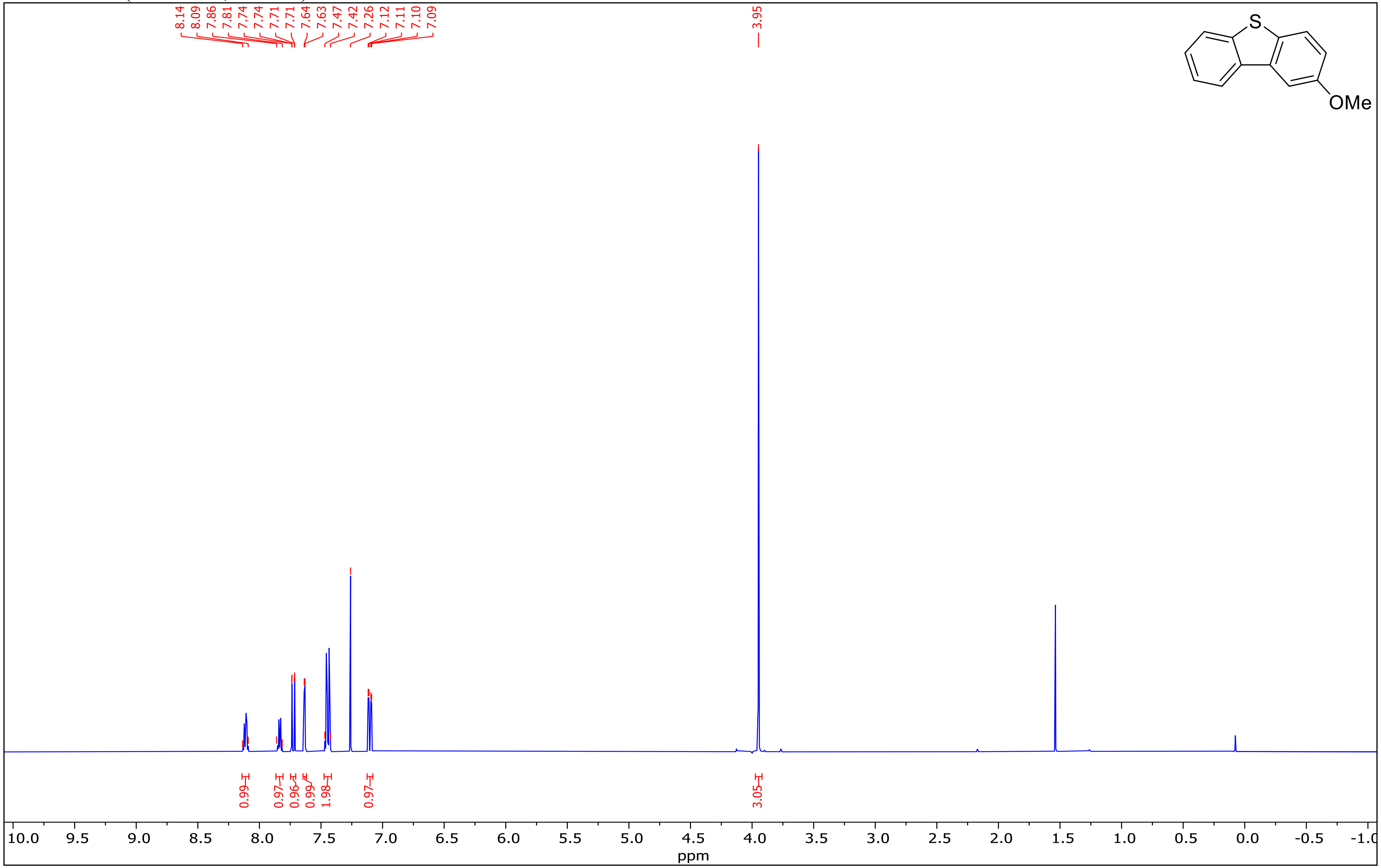

1m' ^{13}C NMR (101 MHz, $CDCl_3$)

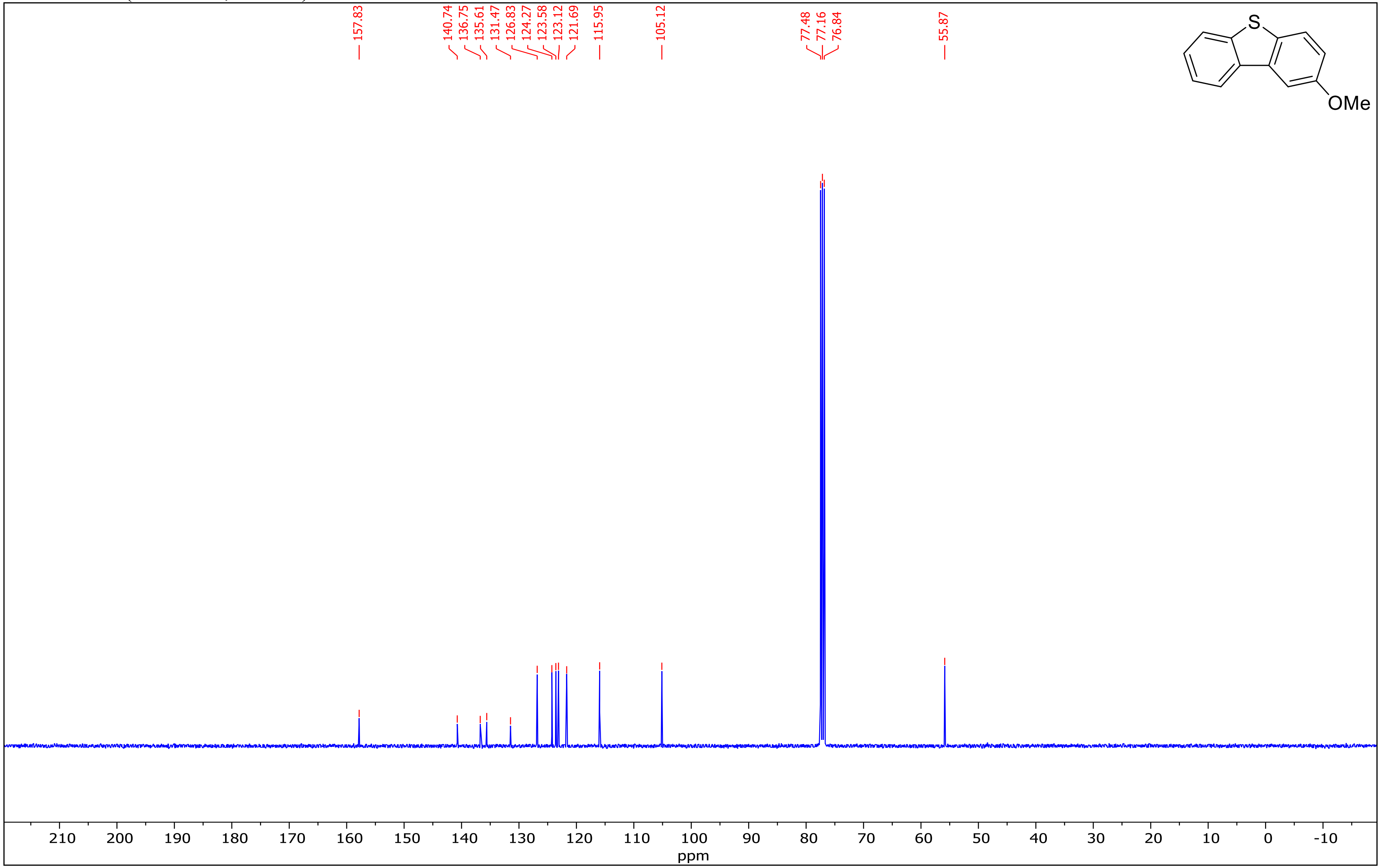

1p' ^{1}H NMR (400 MHz, $CDCl_3$)

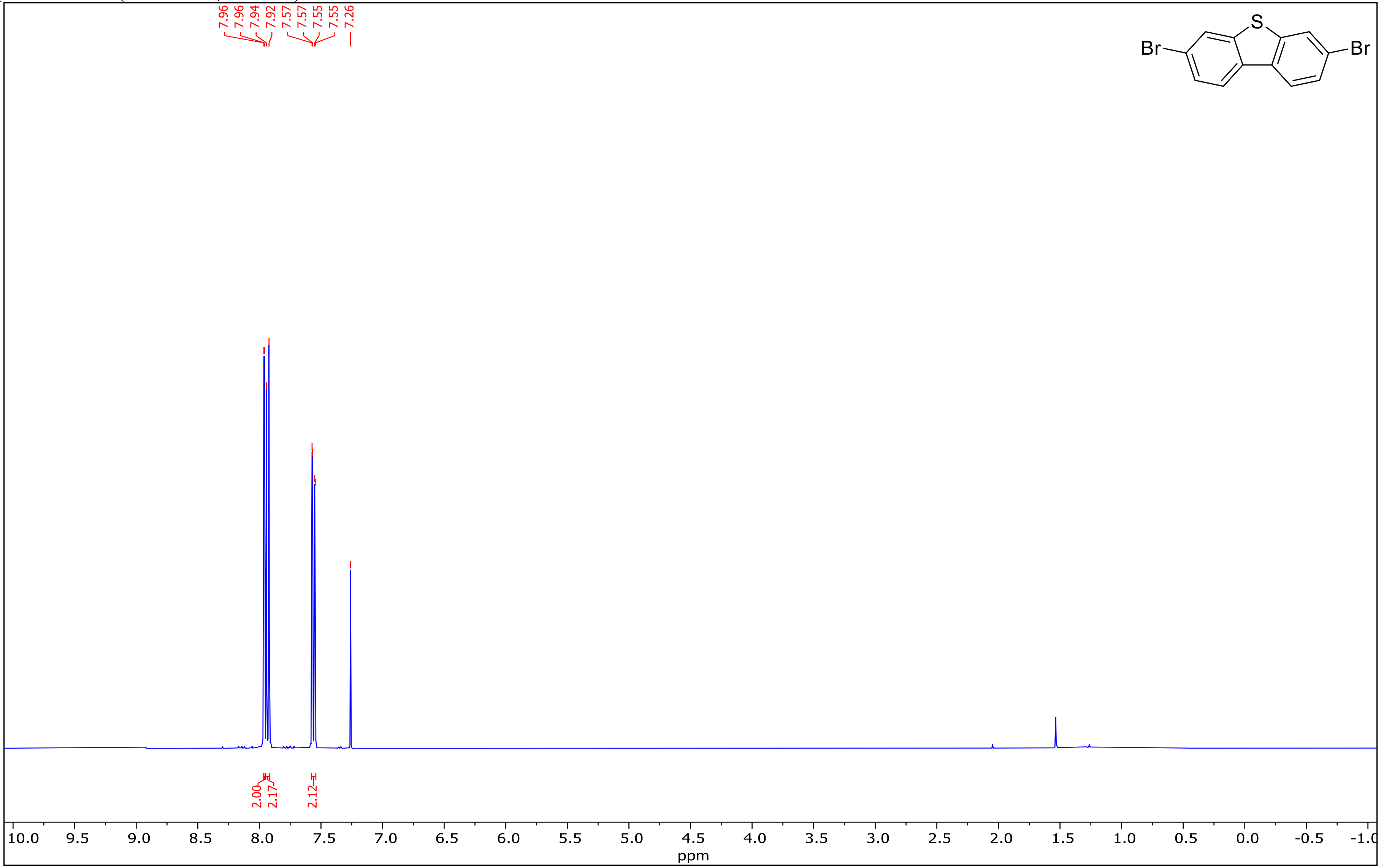

1p' ^{13}C NMR (101 MHz, $CDCl_3$)

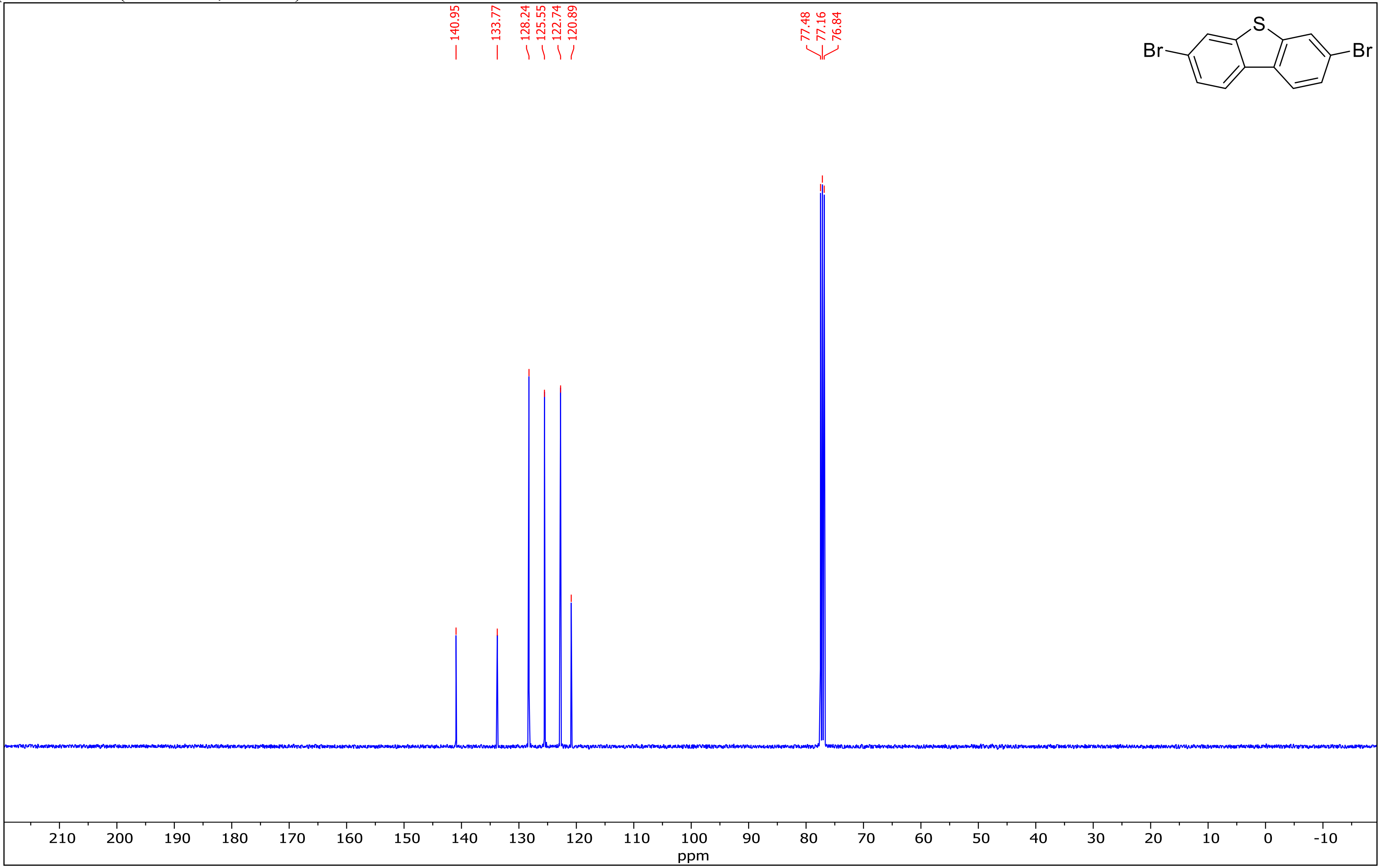

3a ^{1}H NMR (400 MHz, $CDCl_3$)

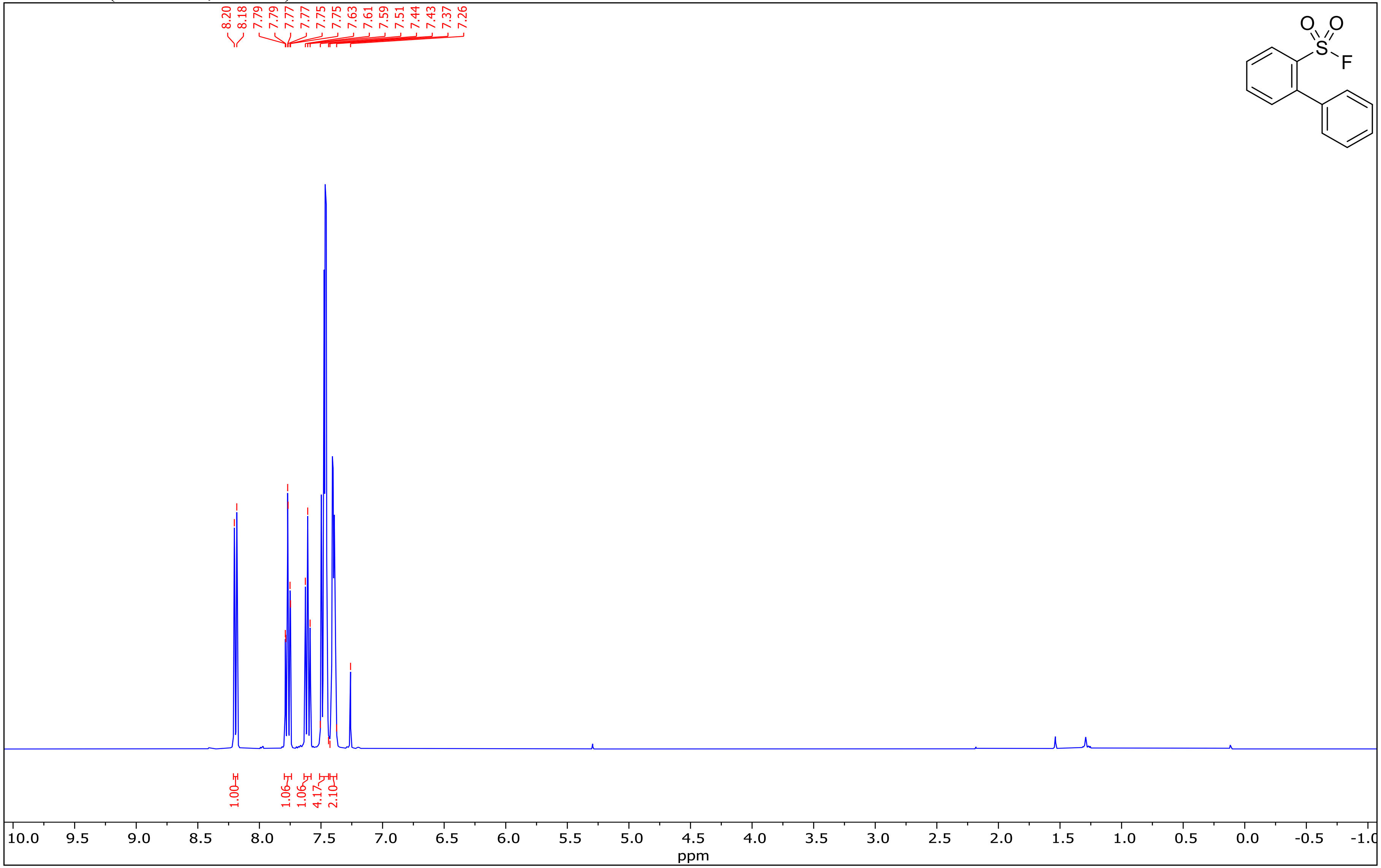

3a ^{13}C NMR (101 MHz, $CDCl_3$)

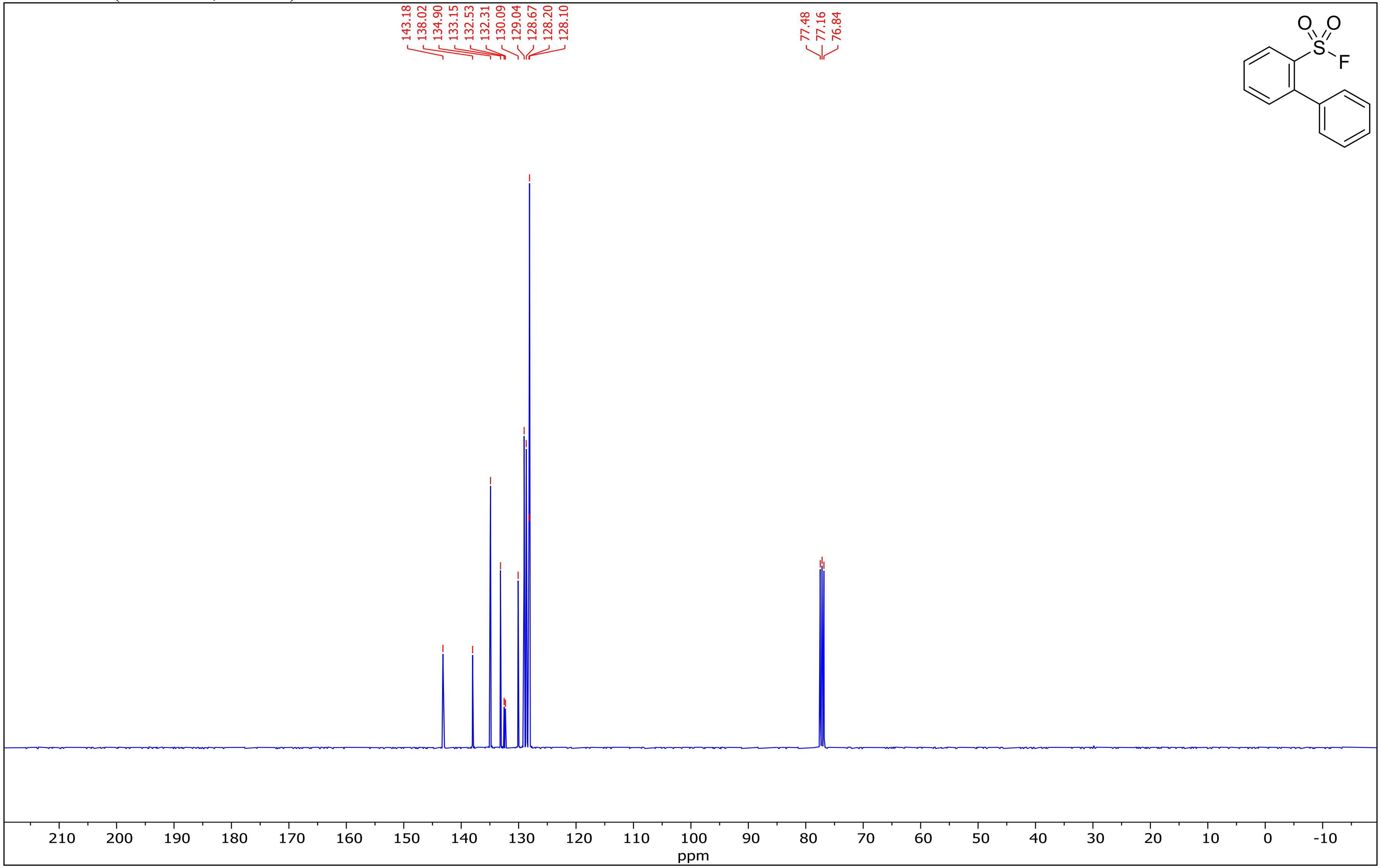

3a ^{19}F NMR (376 MHz, $CDCl_3$)

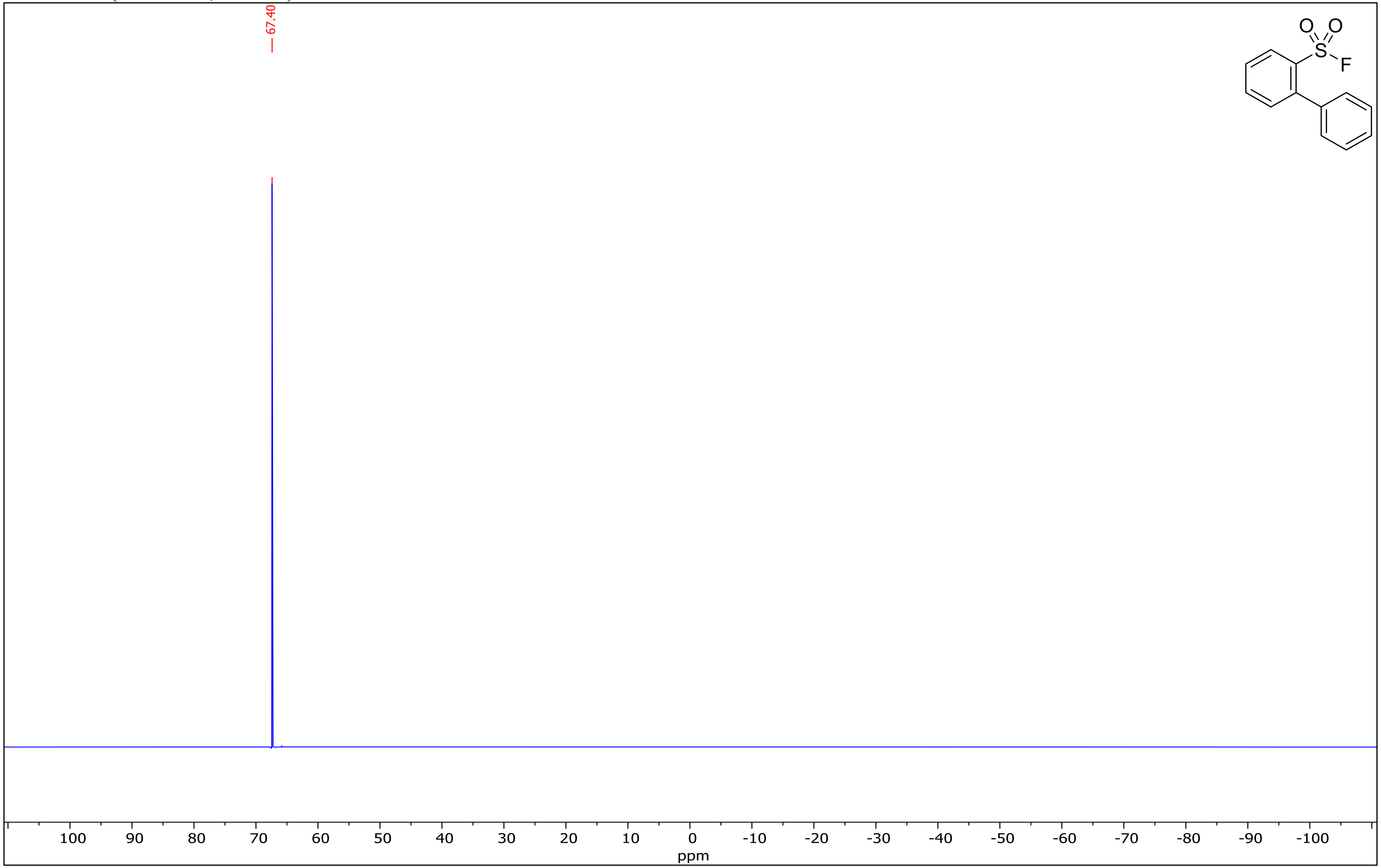

3b ^{1}H NMR (400 MHz, DMSO-d_6)

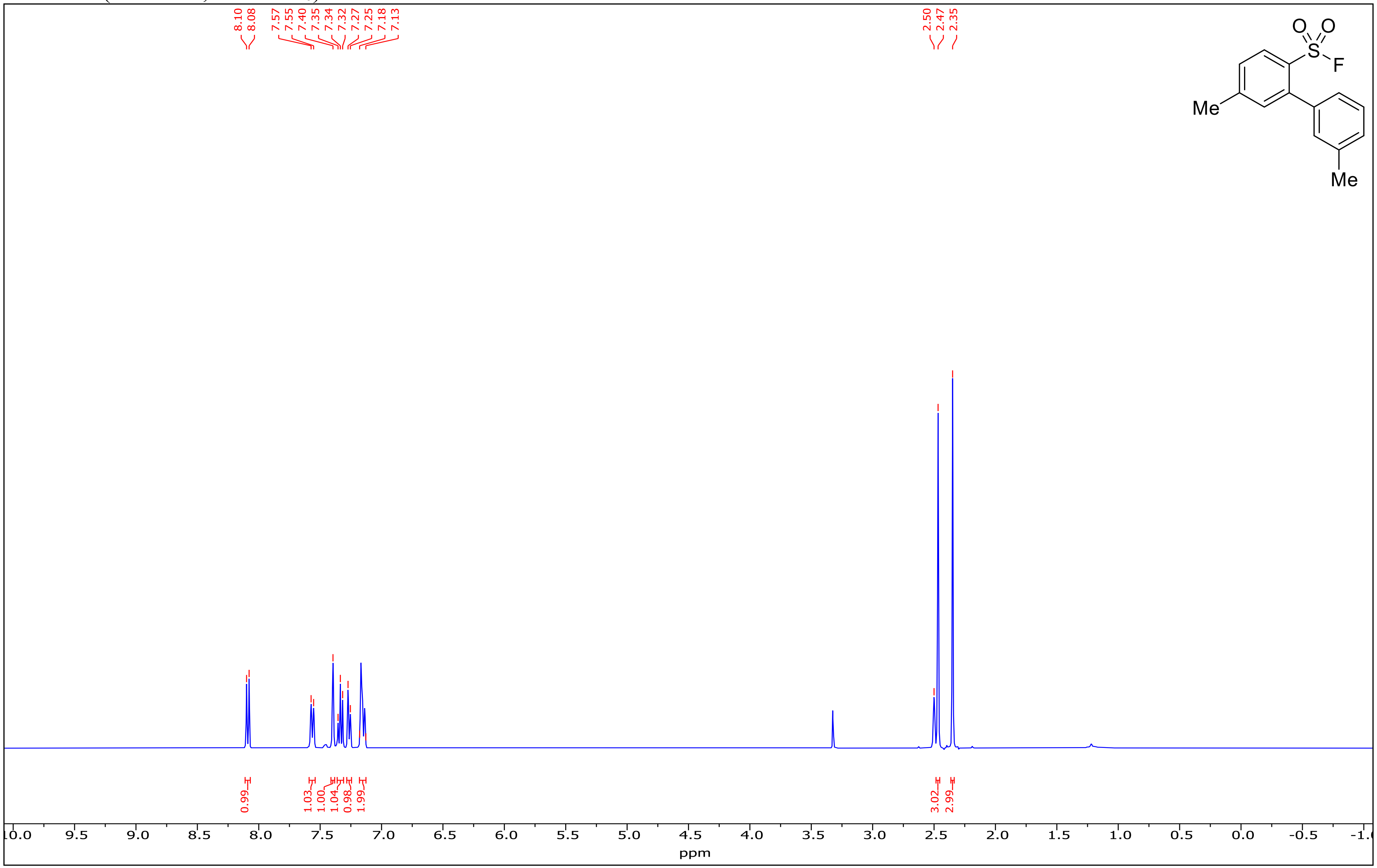

3b ^{13}C NMR (101 MHz, $CDCl_3$)

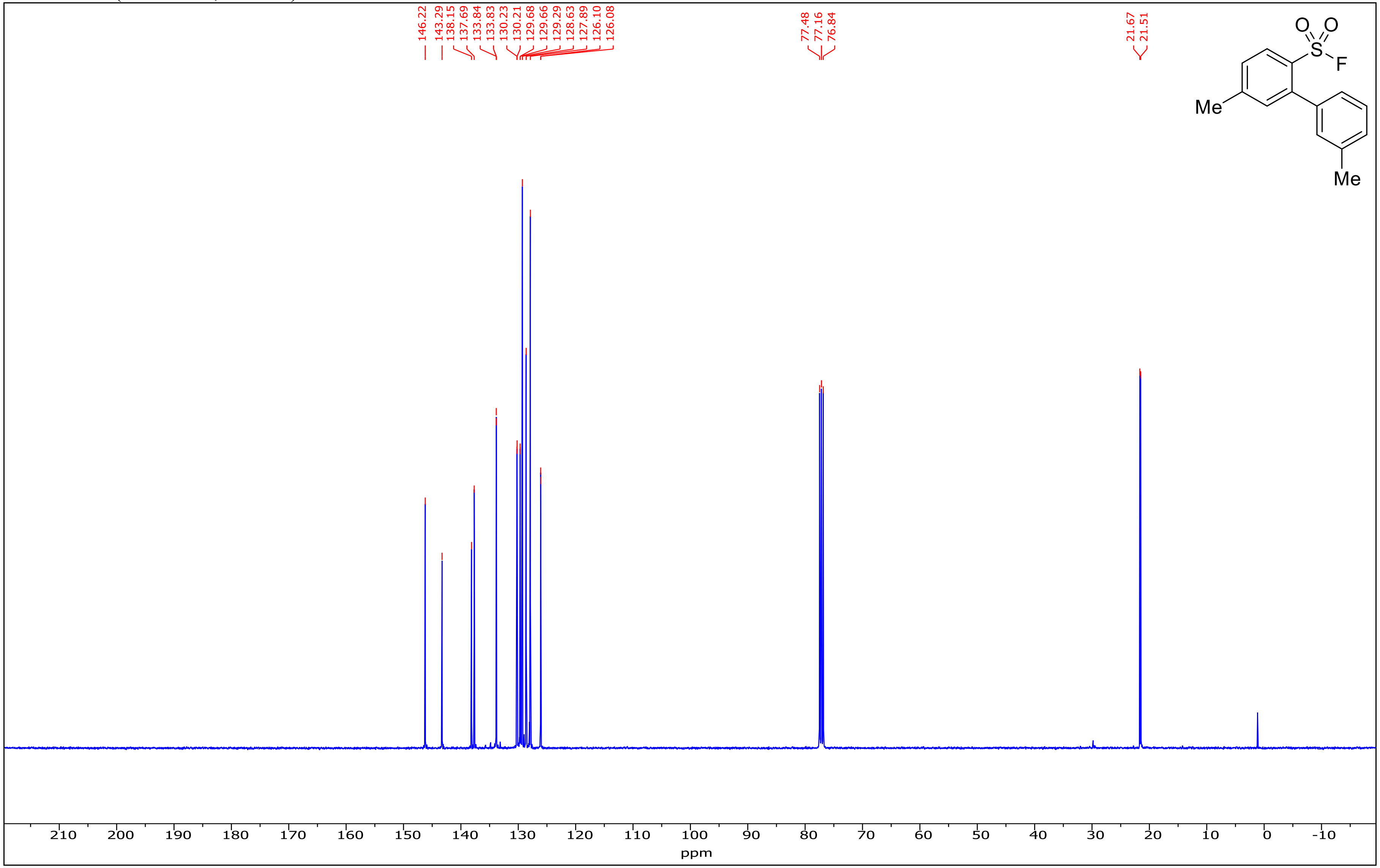

3b ^{19}F NMR (376 MHz, CDCl$_3$)

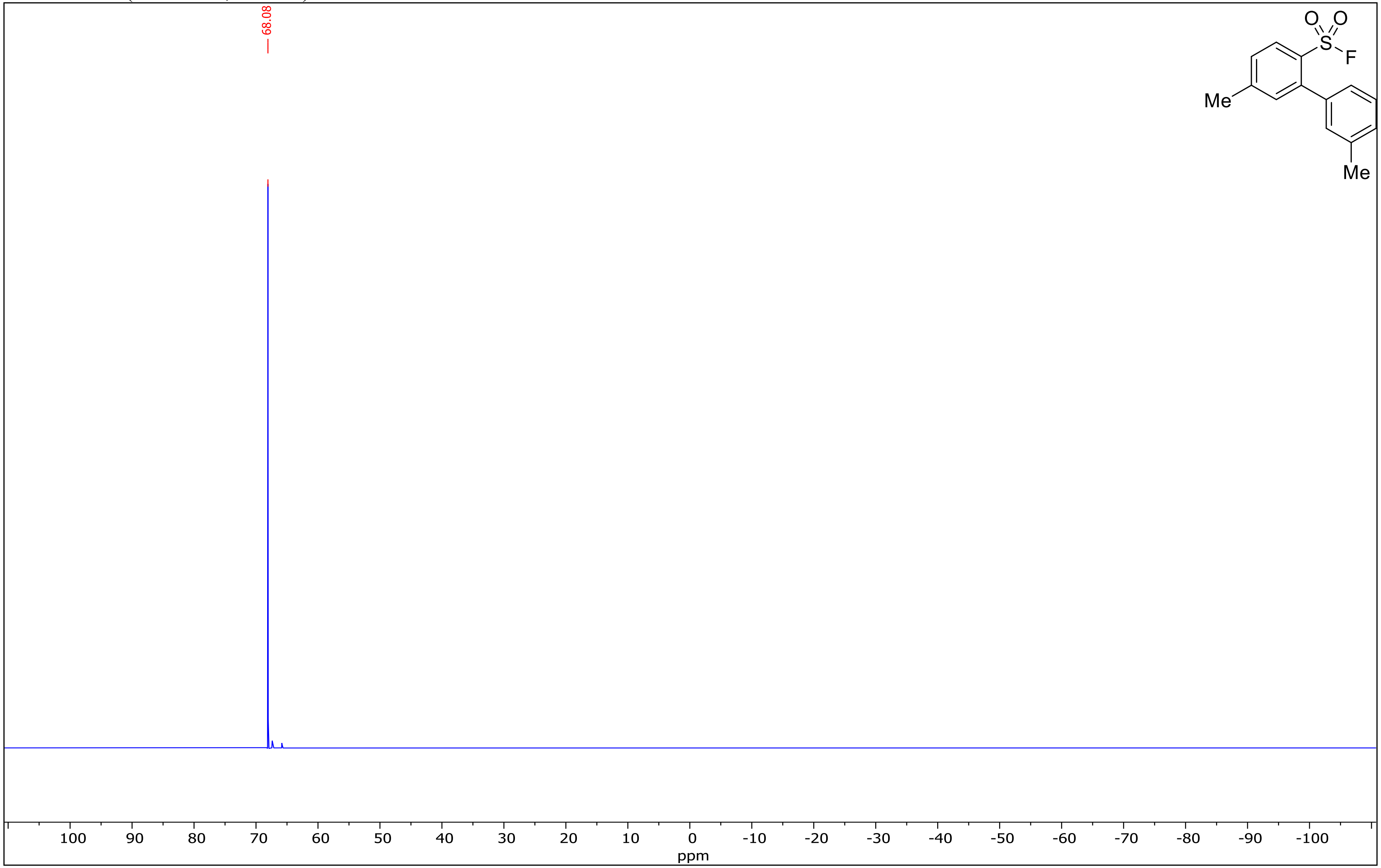

3c ^{1}H NMR (400 MHz, $CDCl_3$)

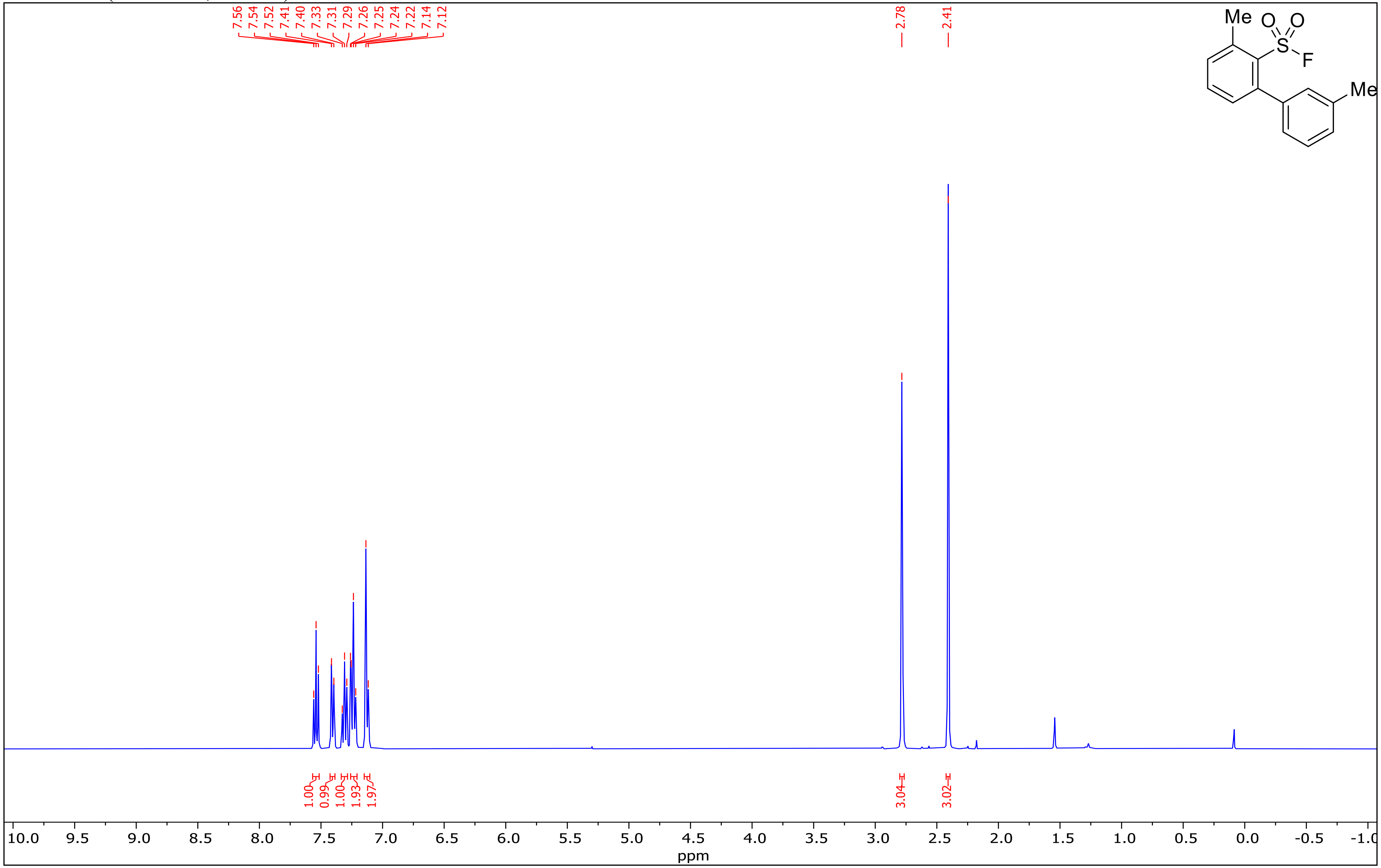

3c ^{13}C NMR (101 MHz, $CDCl_3$)

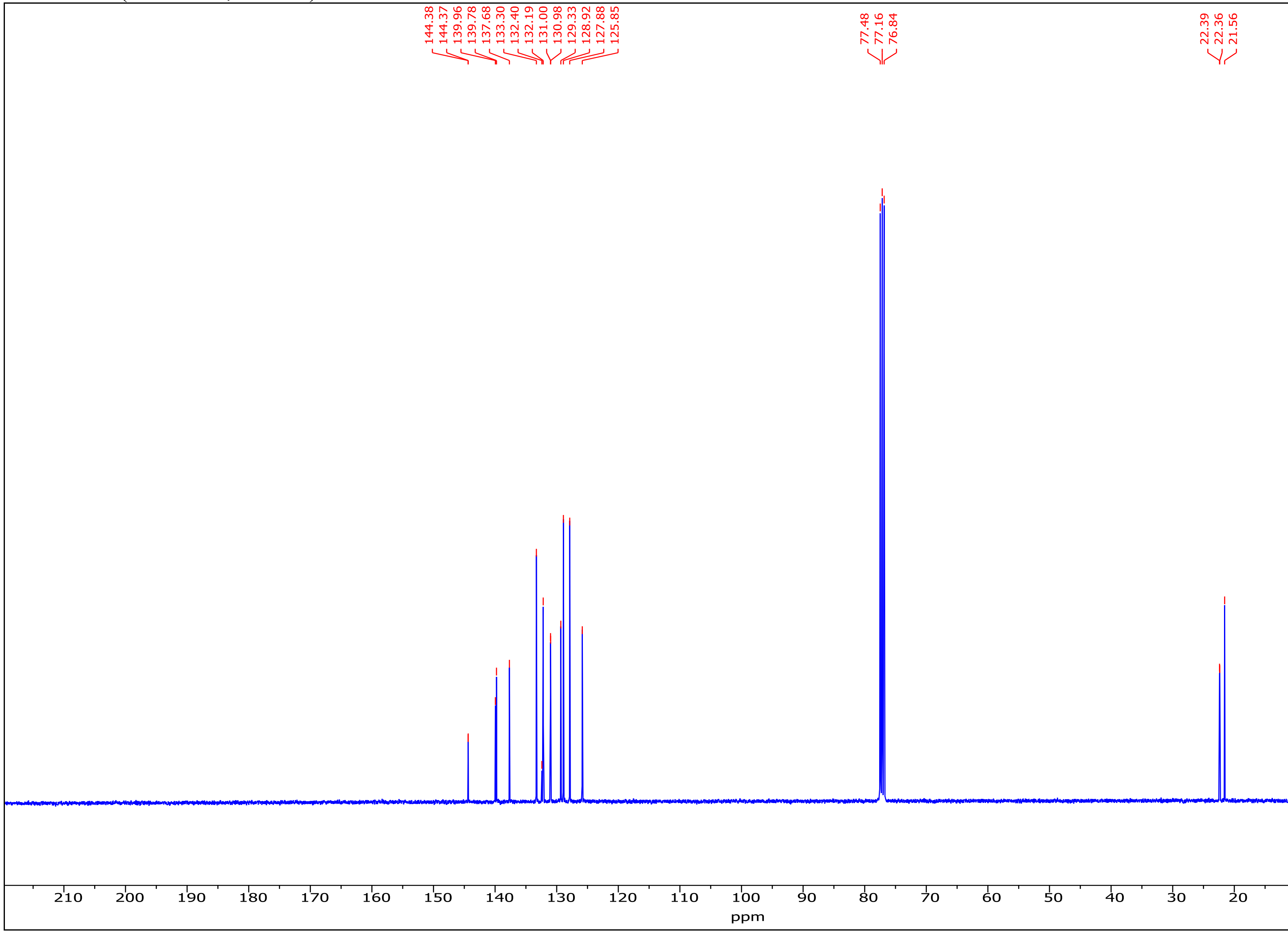

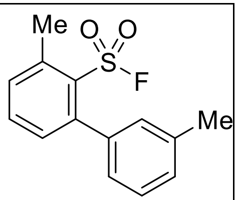

3c ^{19}F NMR (376 MHz, $CDCl_3$)

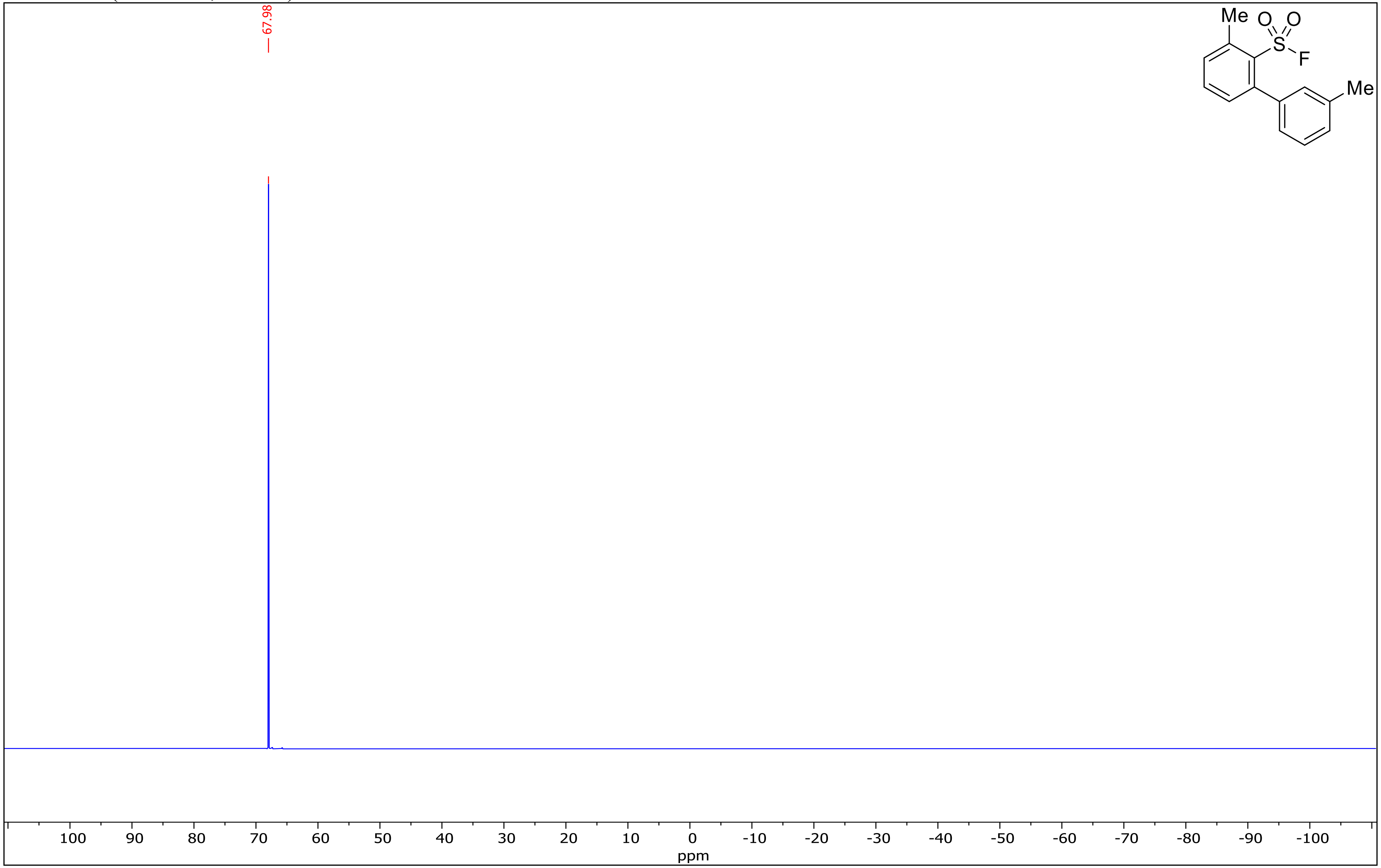

3d ^{1}H NMR (400 MHz, $CDCl_3$)

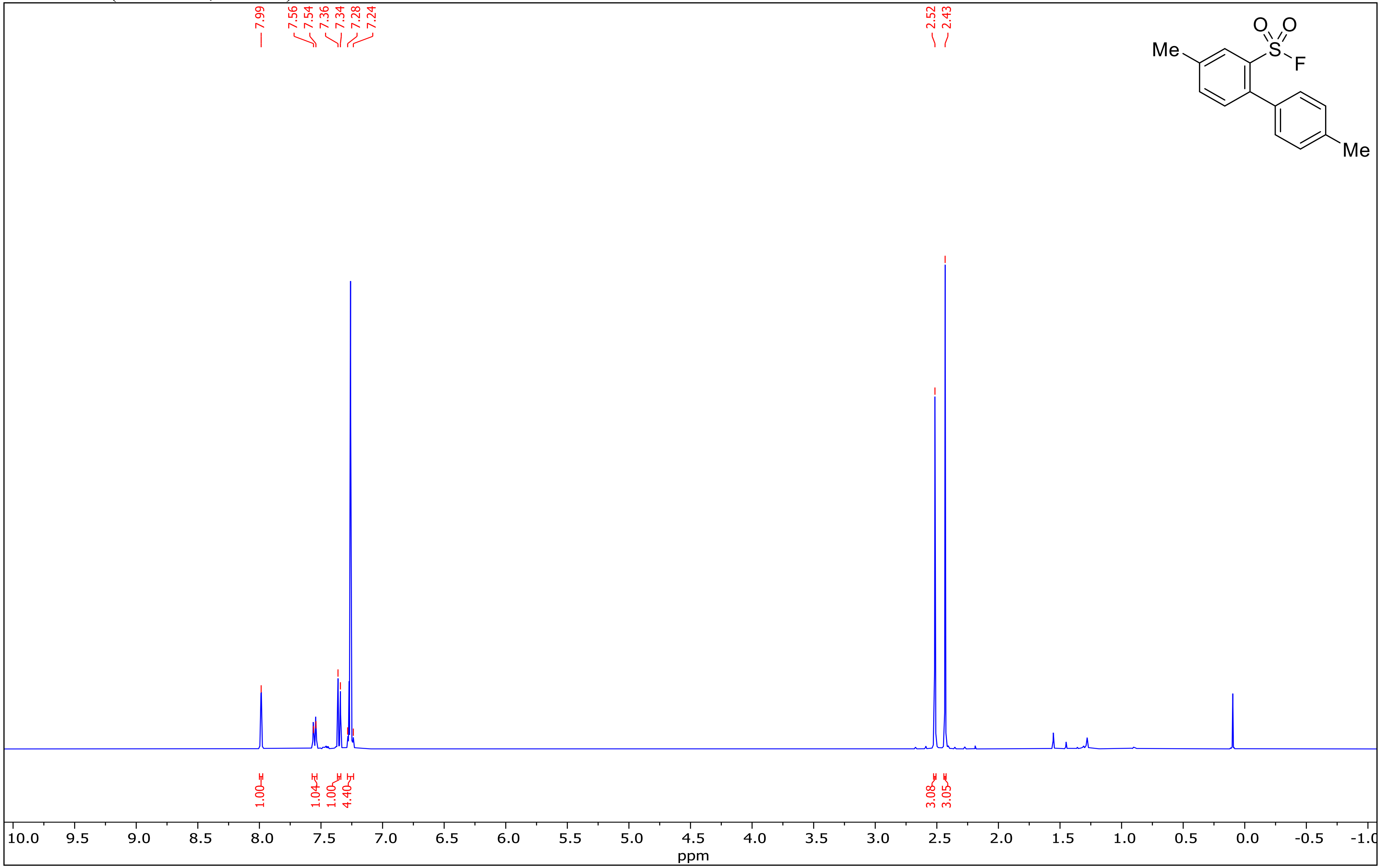

3d ^{13}C NMR (101 MHz, $CDCl_3$)

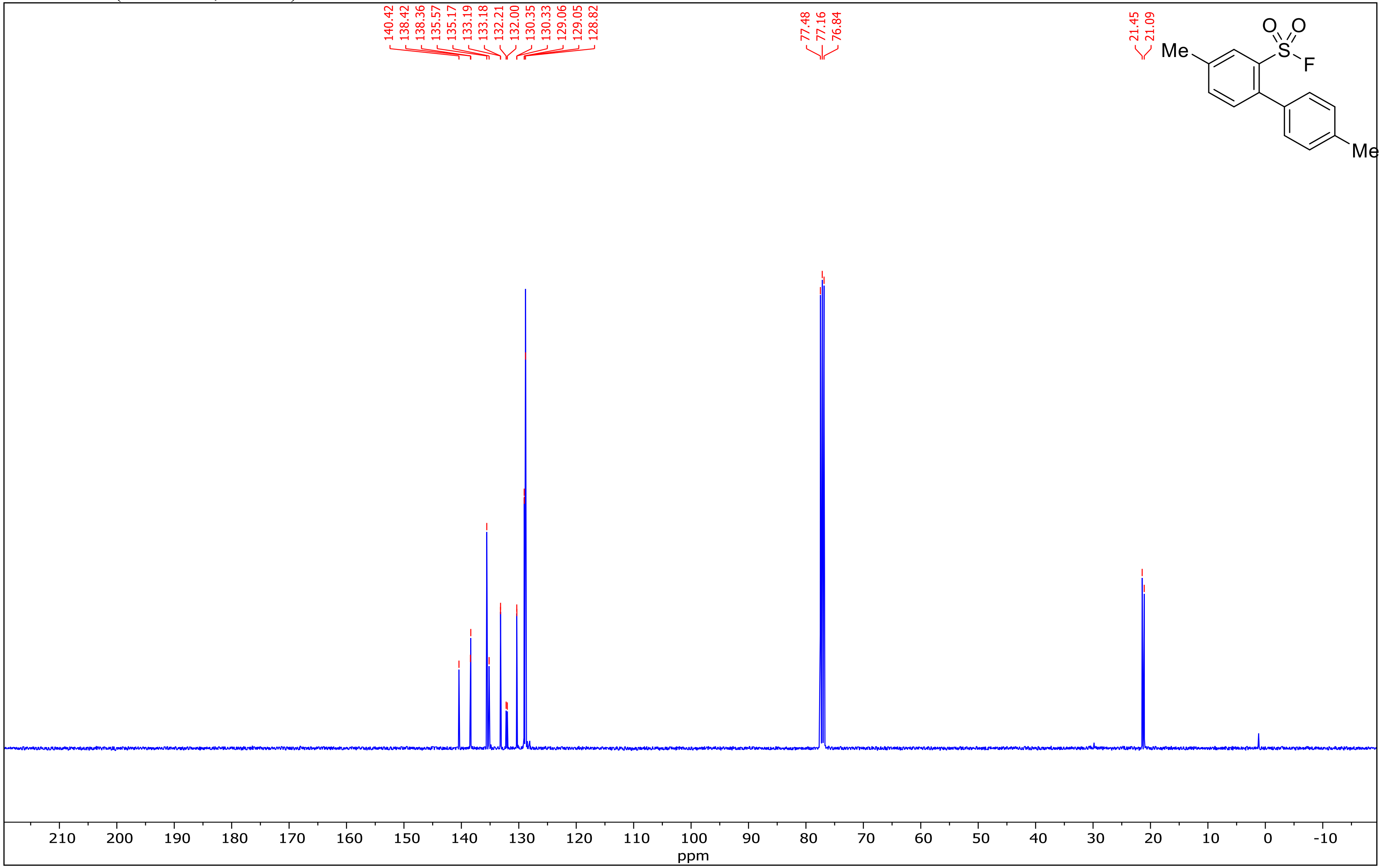

3d ^{19}F NMR (376 MHz, $CDCl_3$)

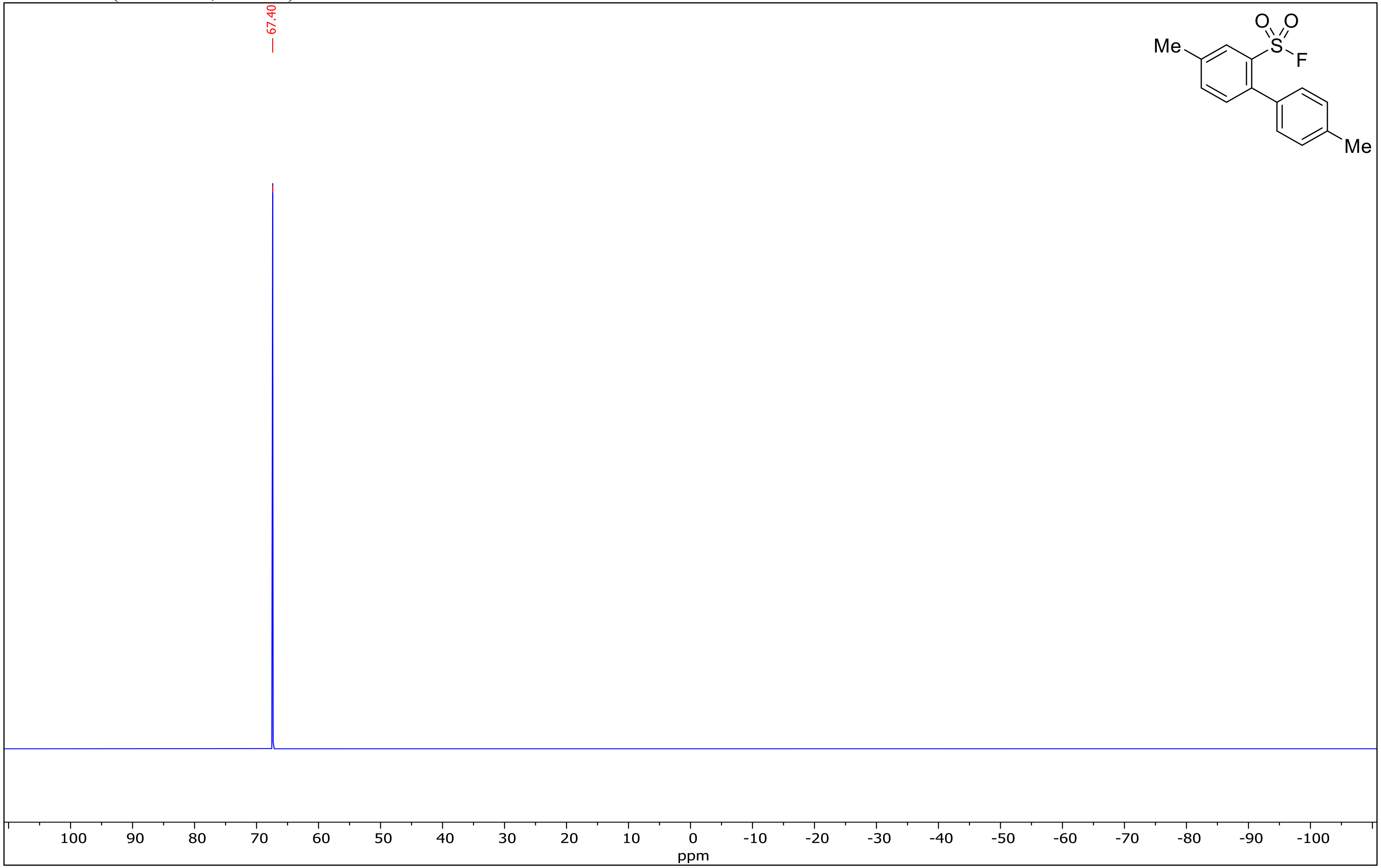

3e ^{1}H NMR (400 MHz, $CDCl_3$)

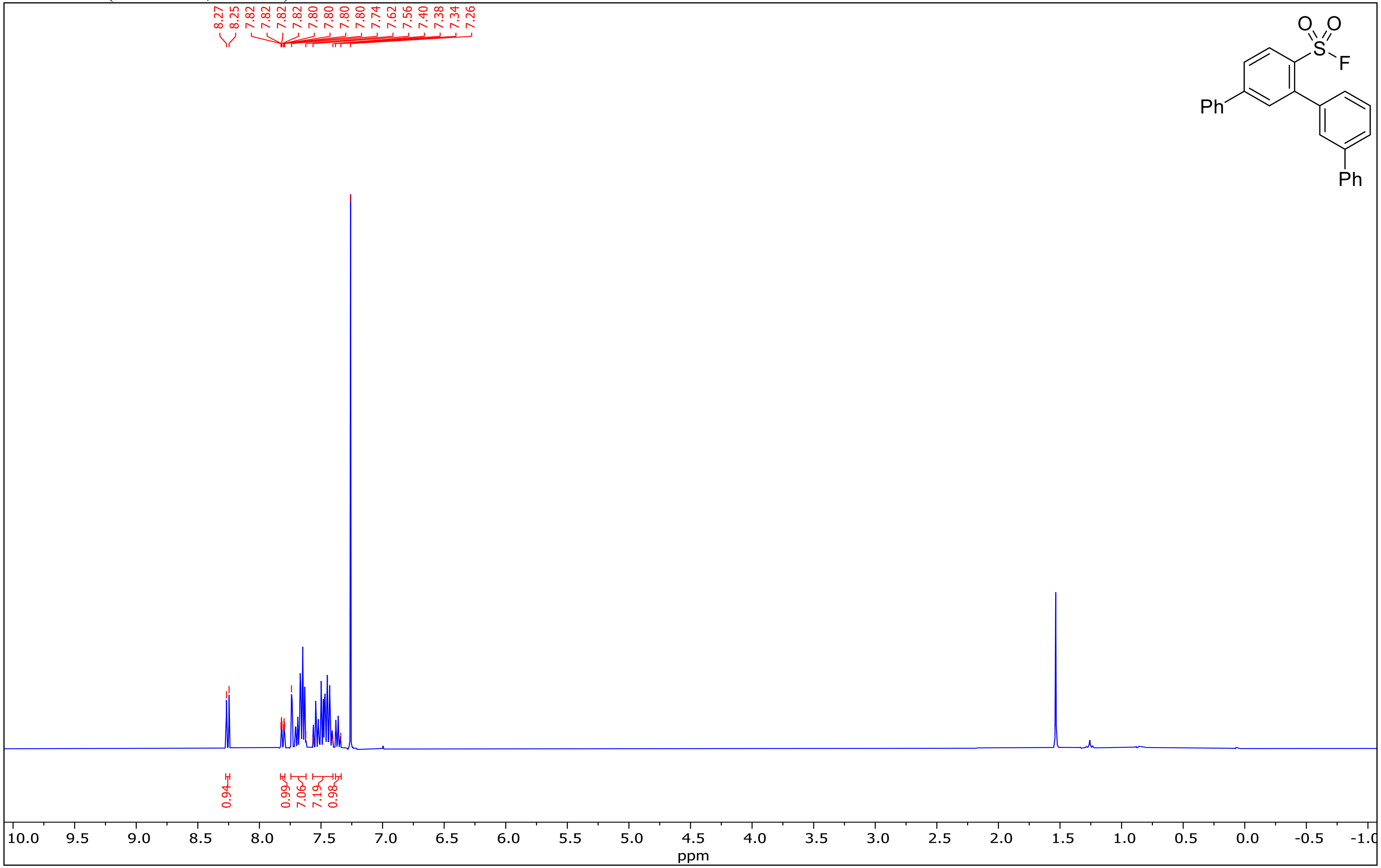

3e ^{13}C NMR (101 MHz, $CDCl_3$)

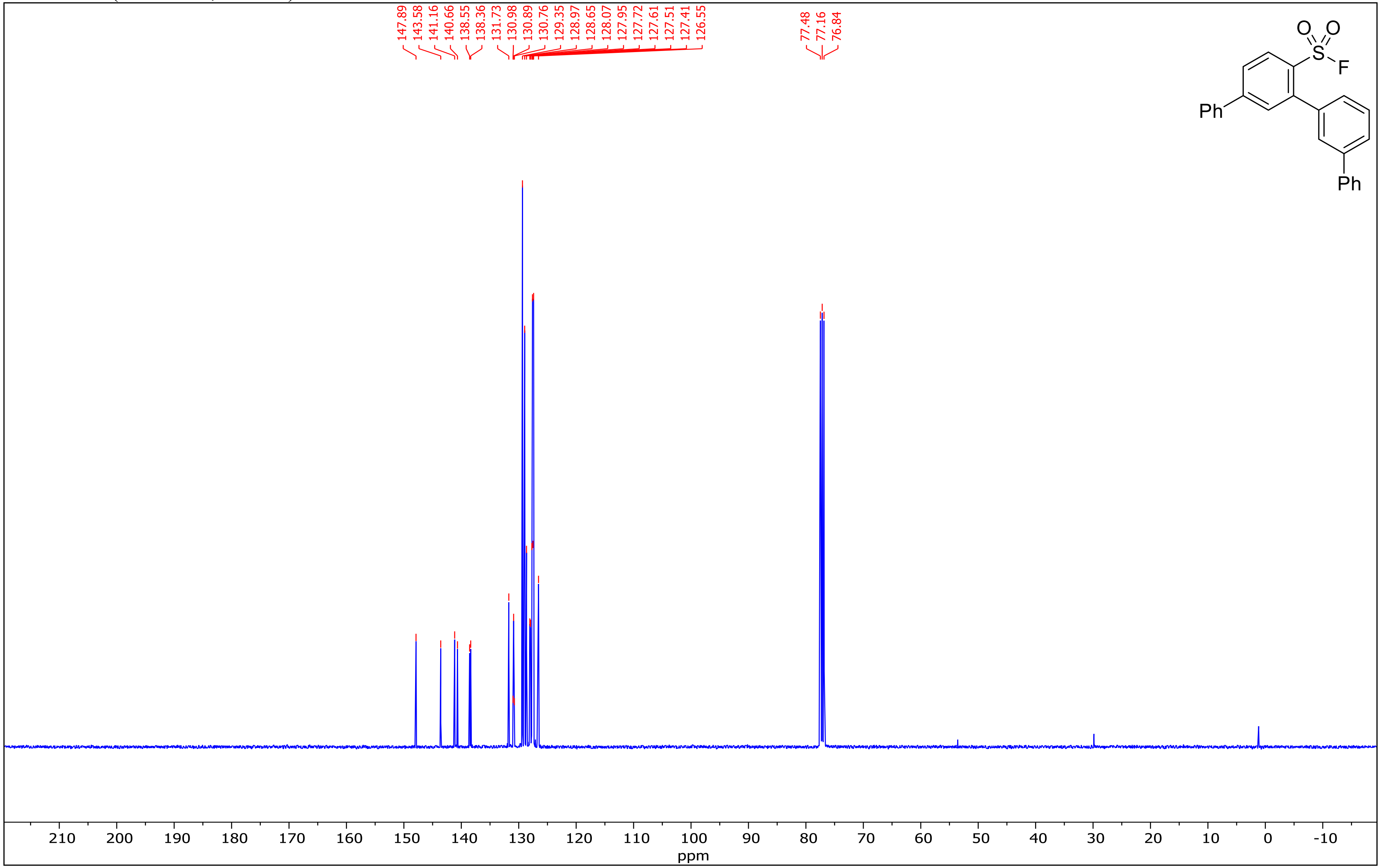

3e ^{19}F NMR (376 MHz, $CDCl_3$)

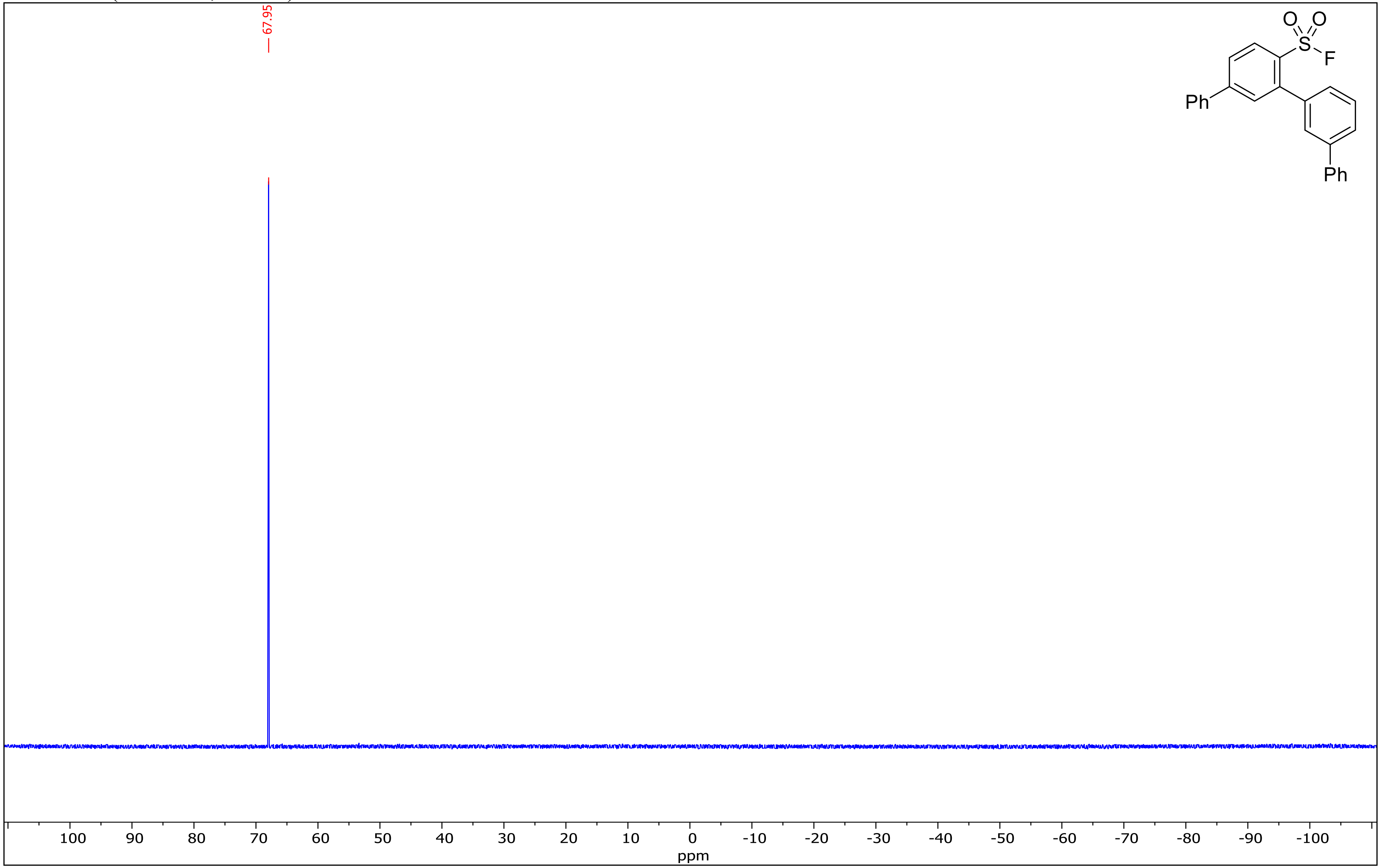

3f ^1H NMR (400 MHz, $CDCl_3$)

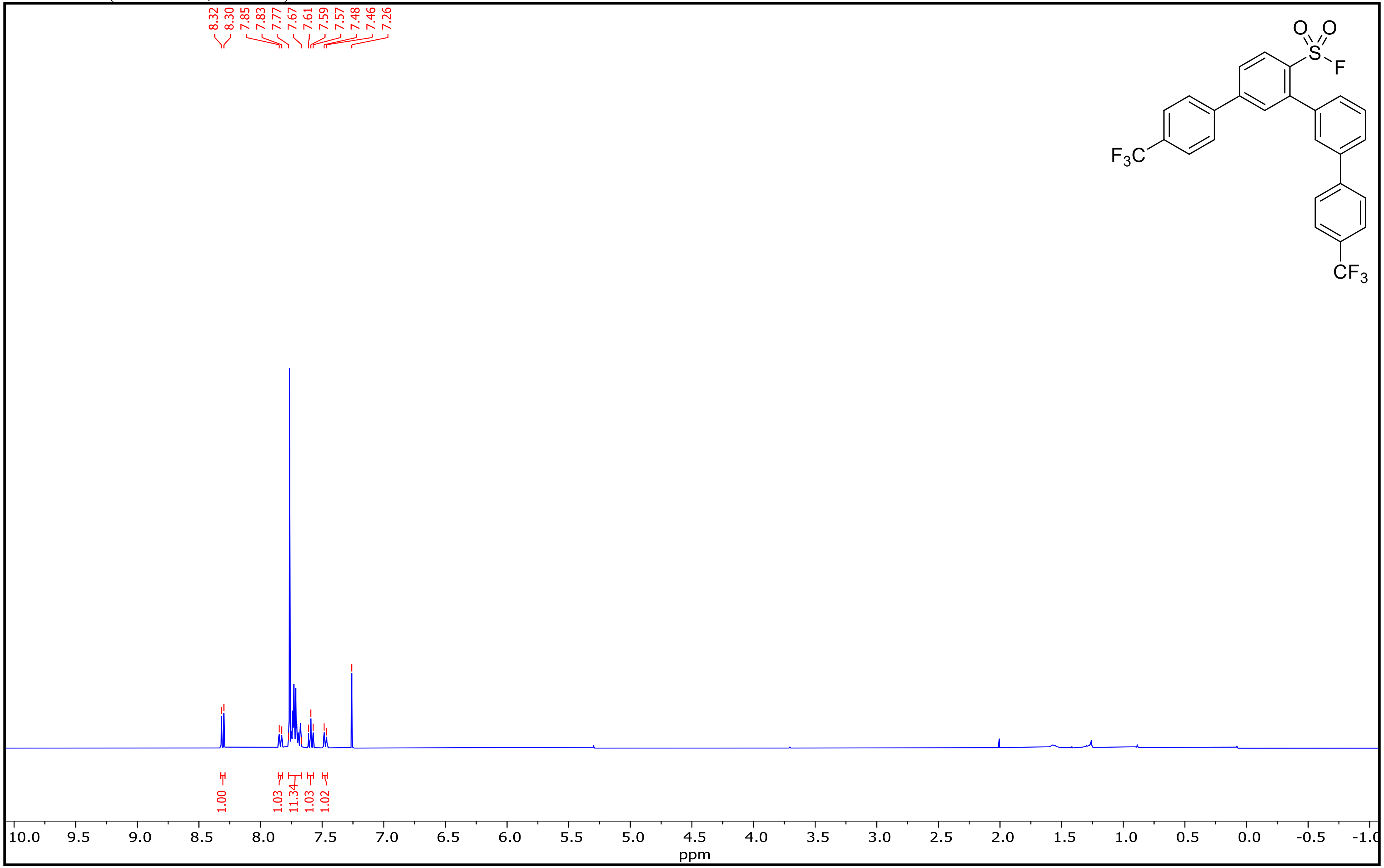

3f ^{13}C NMR (101 MHz, $CDCl_3$)

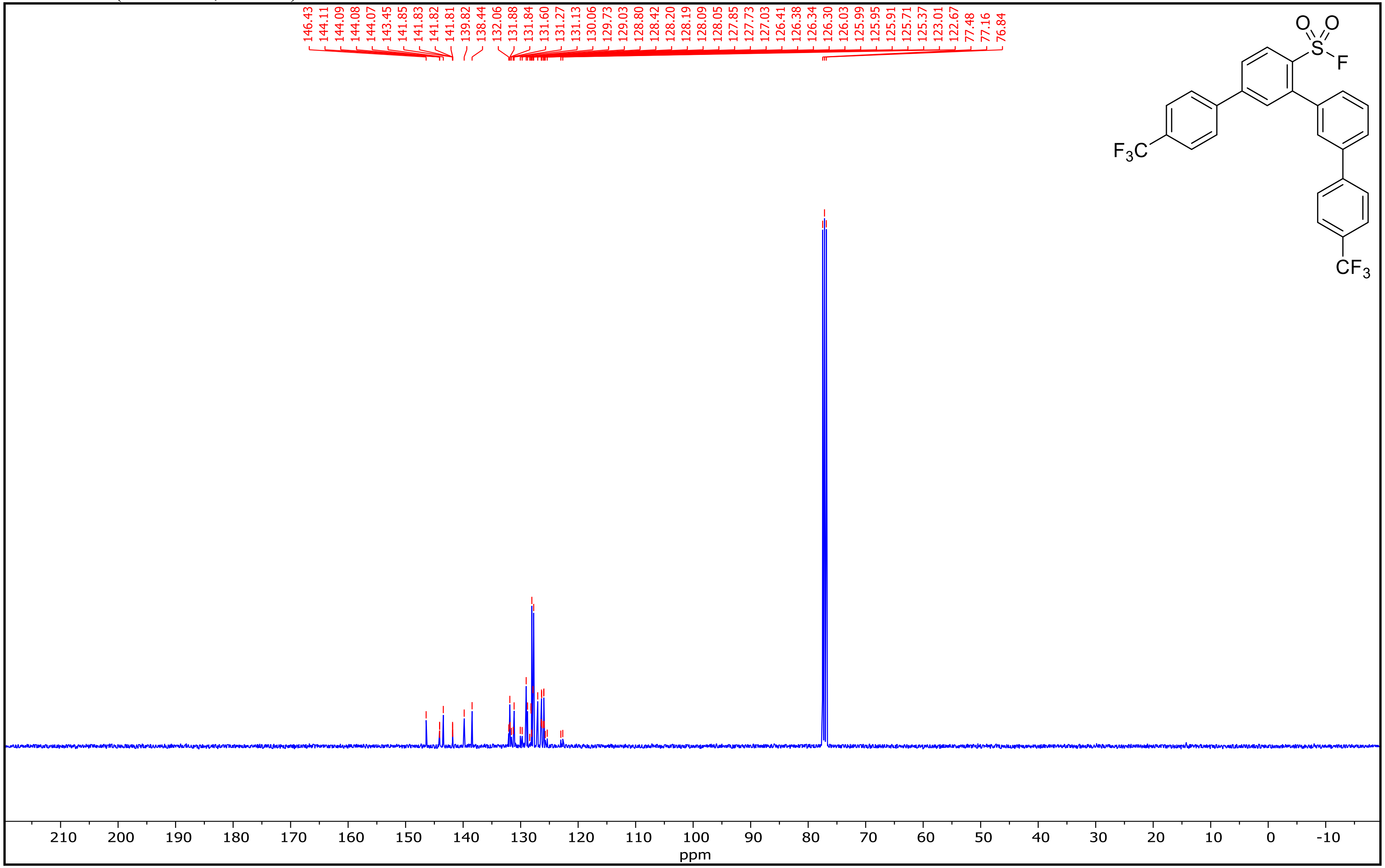

3f ^{19}F NMR (376 MHz, $CDCl_3$)

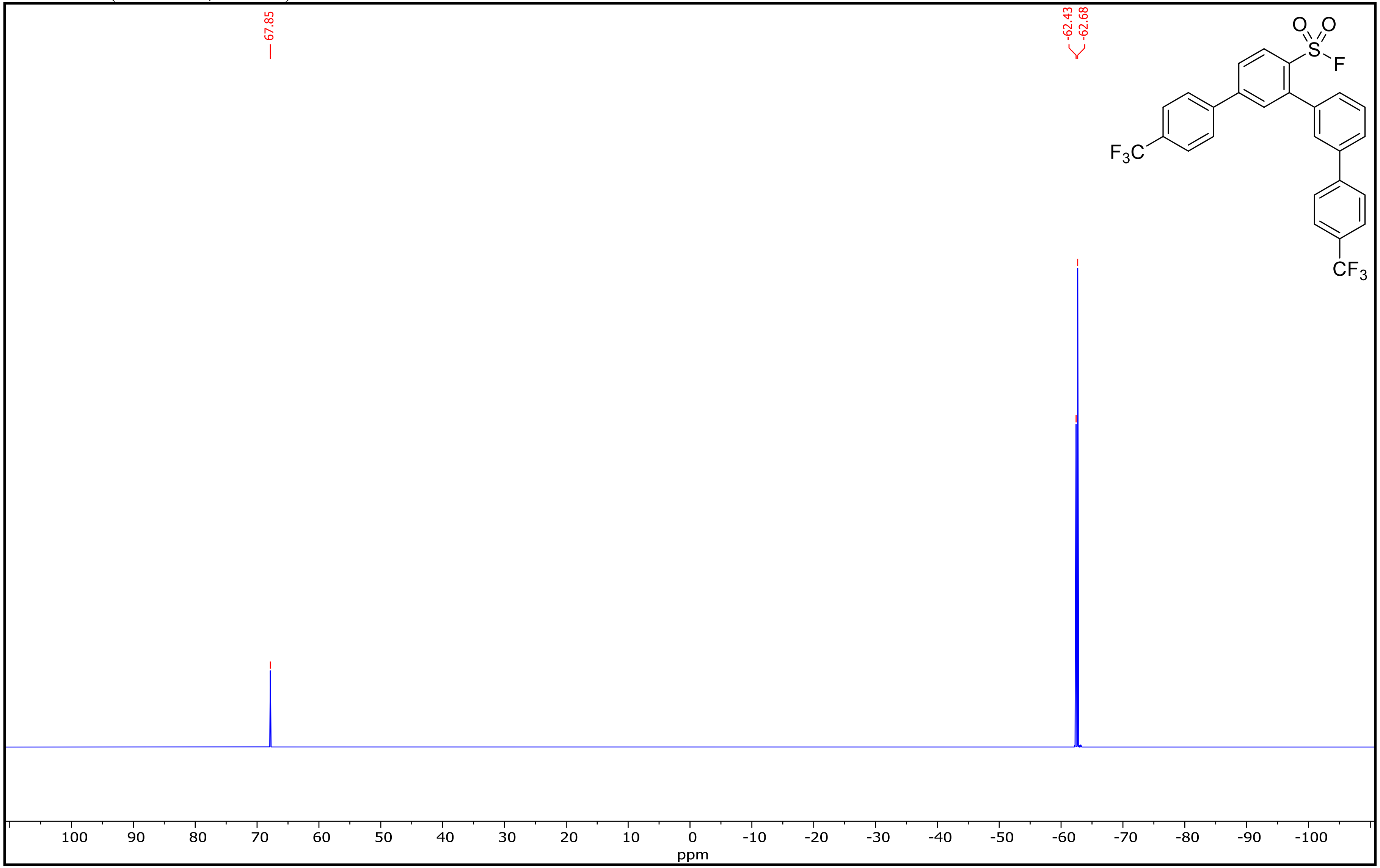

3g ^{1}H NMR (400 MHz, $CDCl_3$)

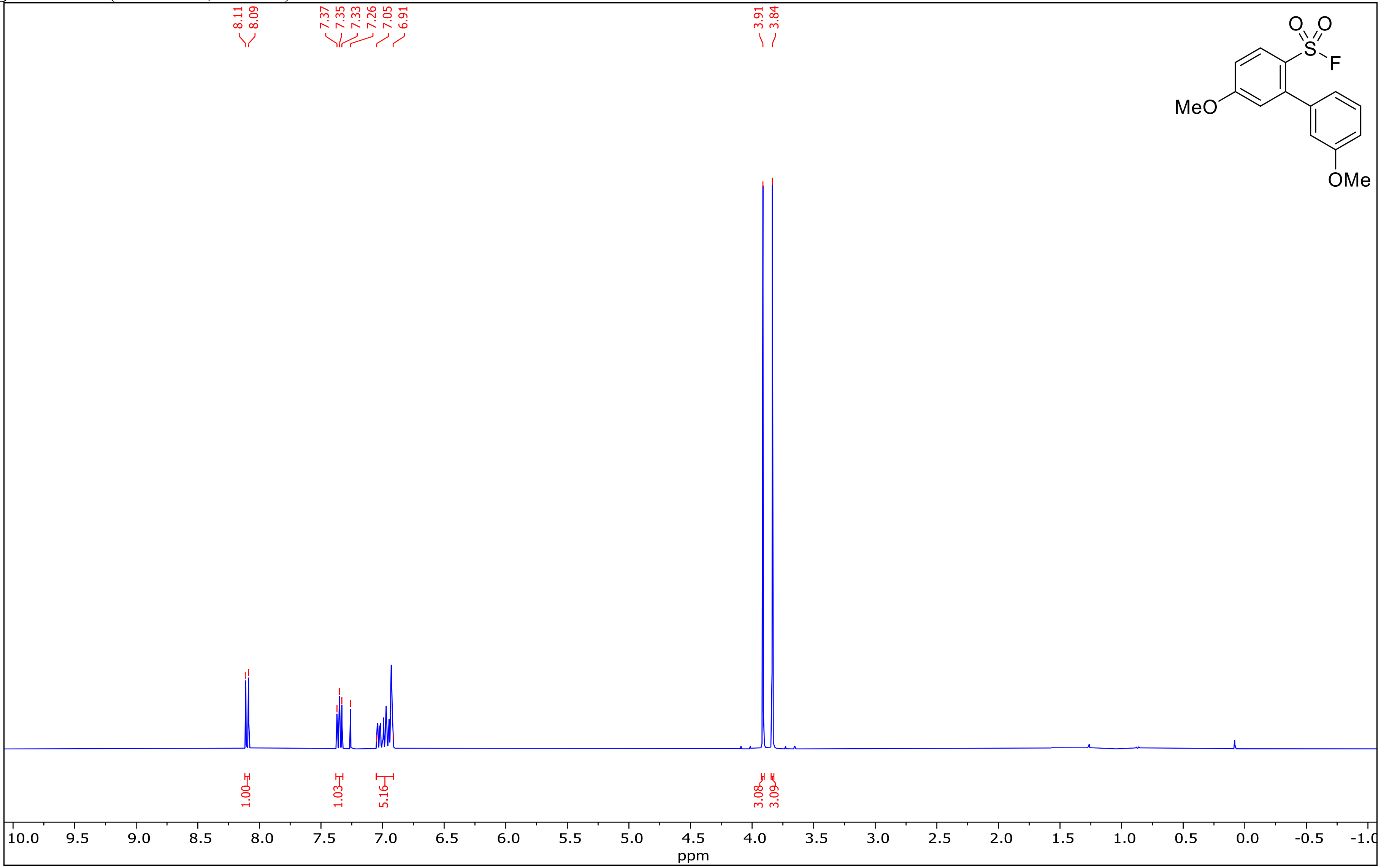

3g ^{13}C NMR (101 MHz, $CDCl_3$)

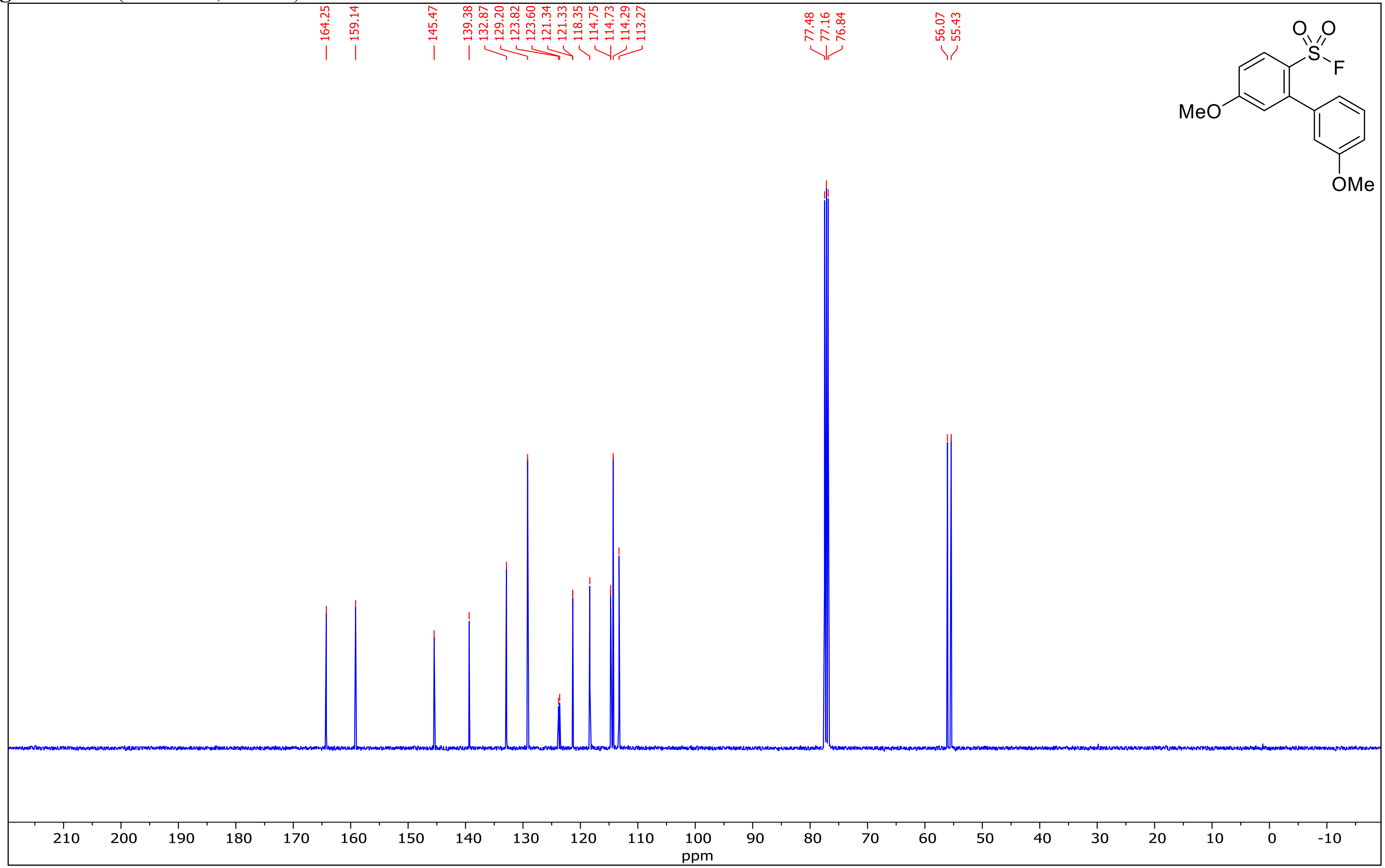

3g ^{19}F NMR (376 MHz, $CDCl_3$)

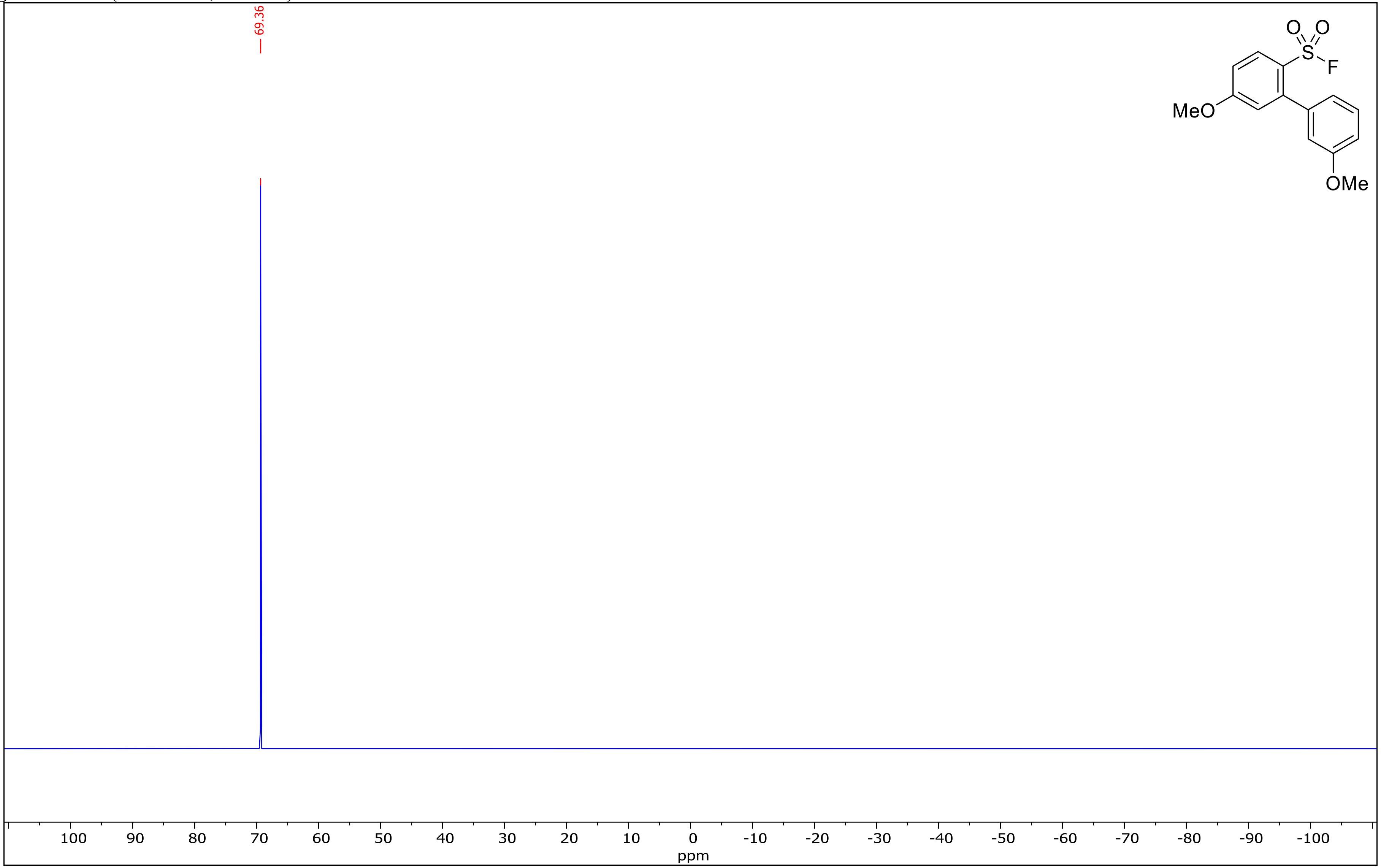

3h ^{1}H NMR (400 MHz, DMSO-d_6)

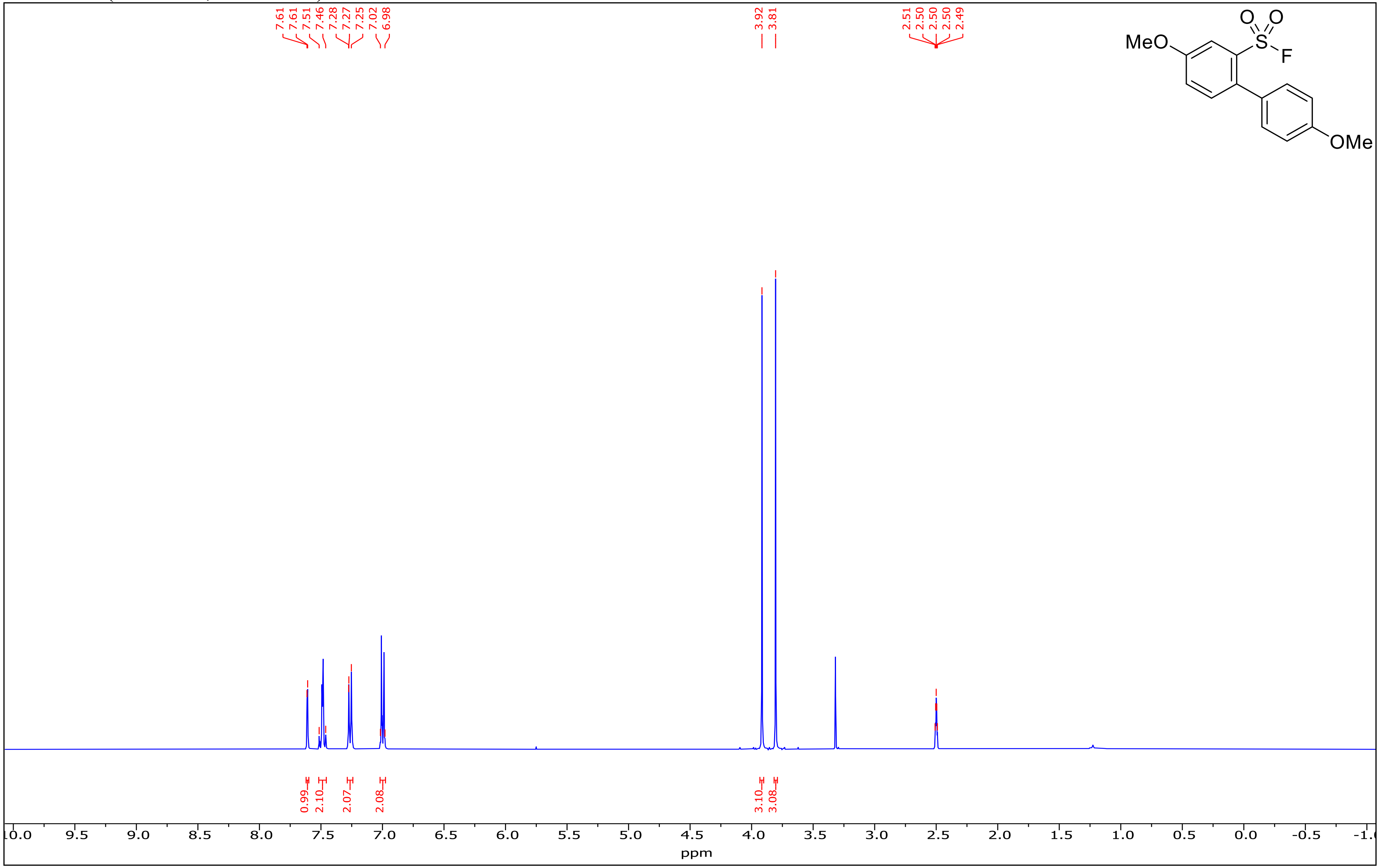

3h ^{13}C NMR (101 MHz, $CDCl_3$)

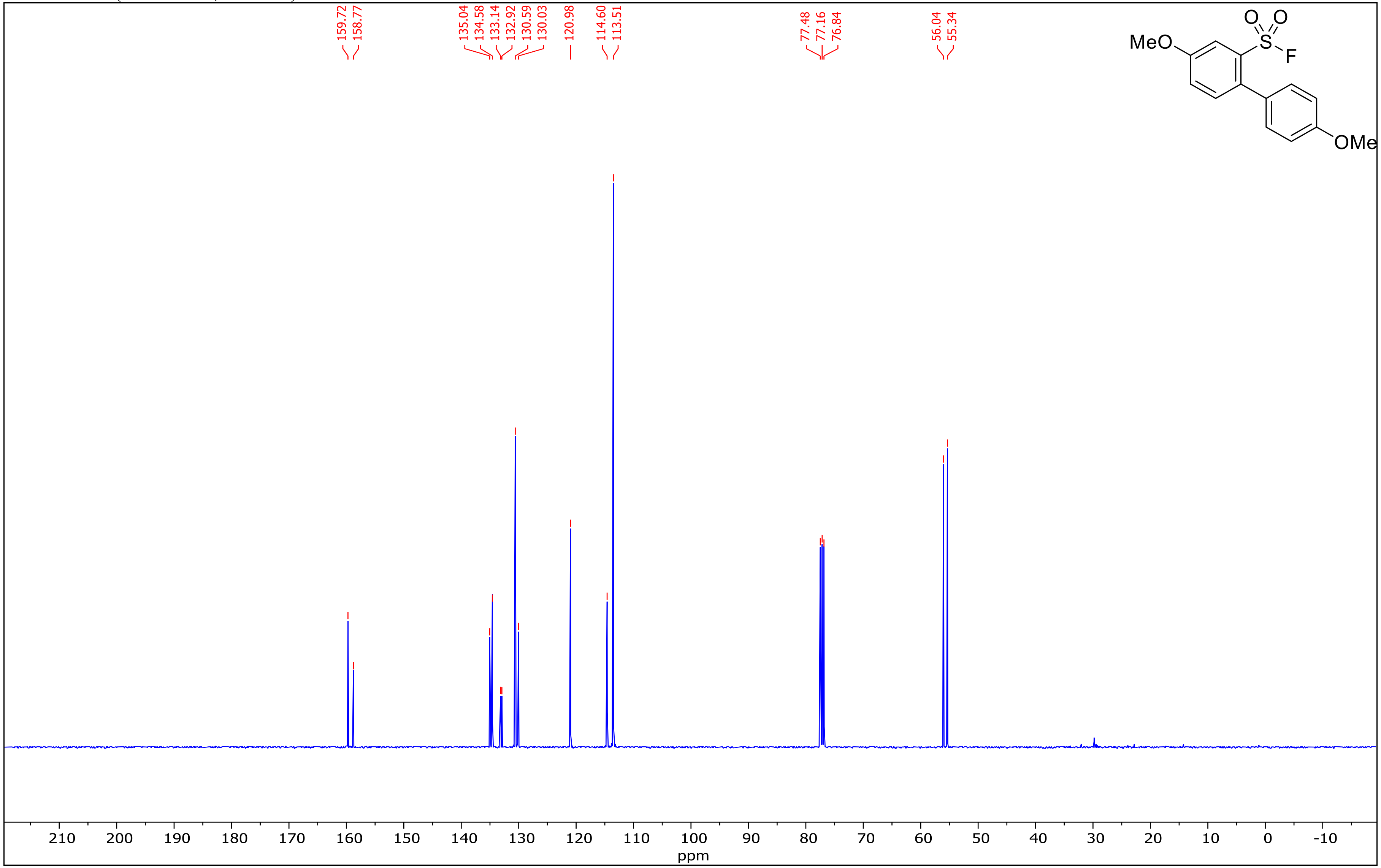

3h ^{19}F NMR (376 MHz, $CDCl_3$)

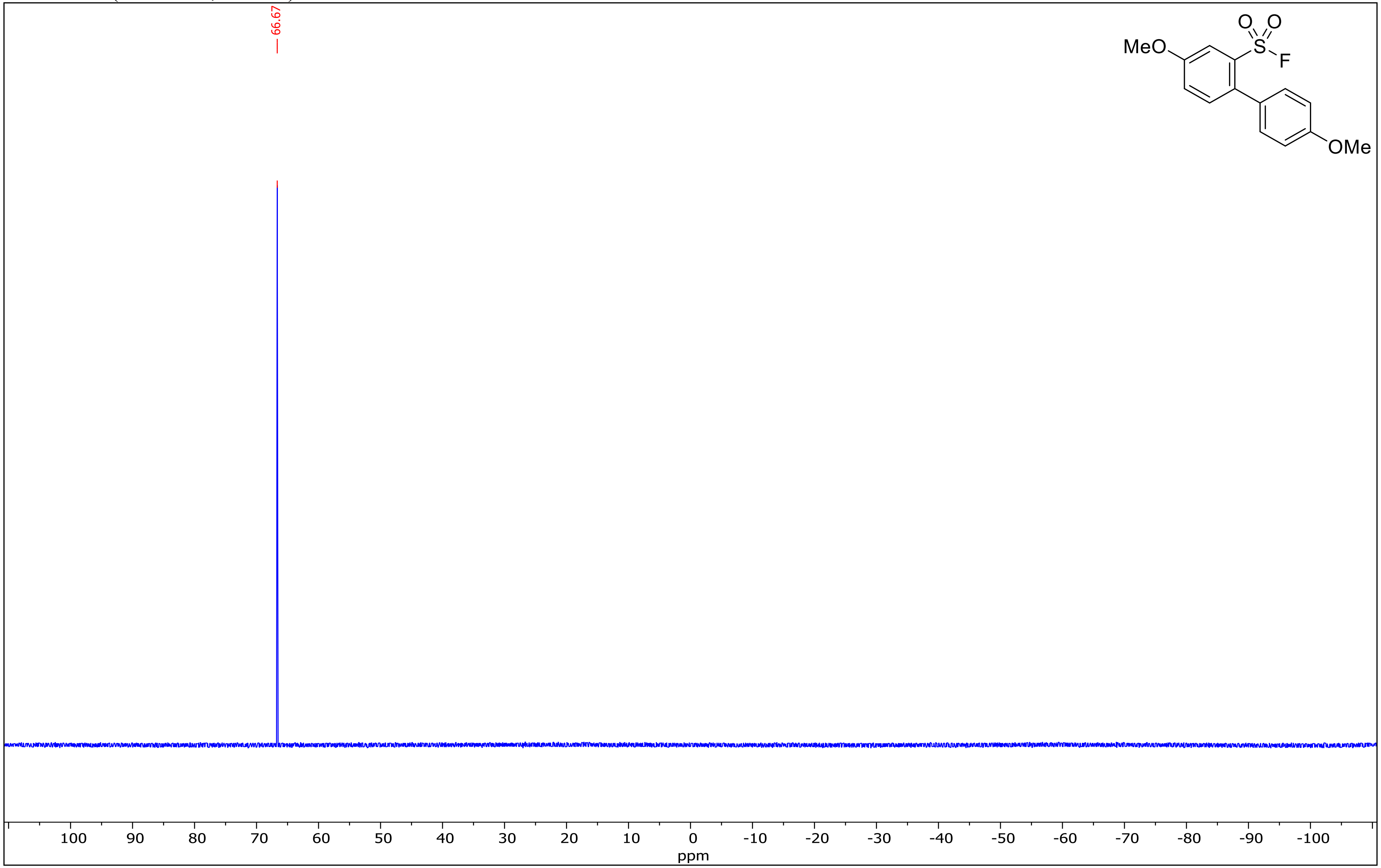

3i ^1H NMR (400 MHz, $CDCl_3$)

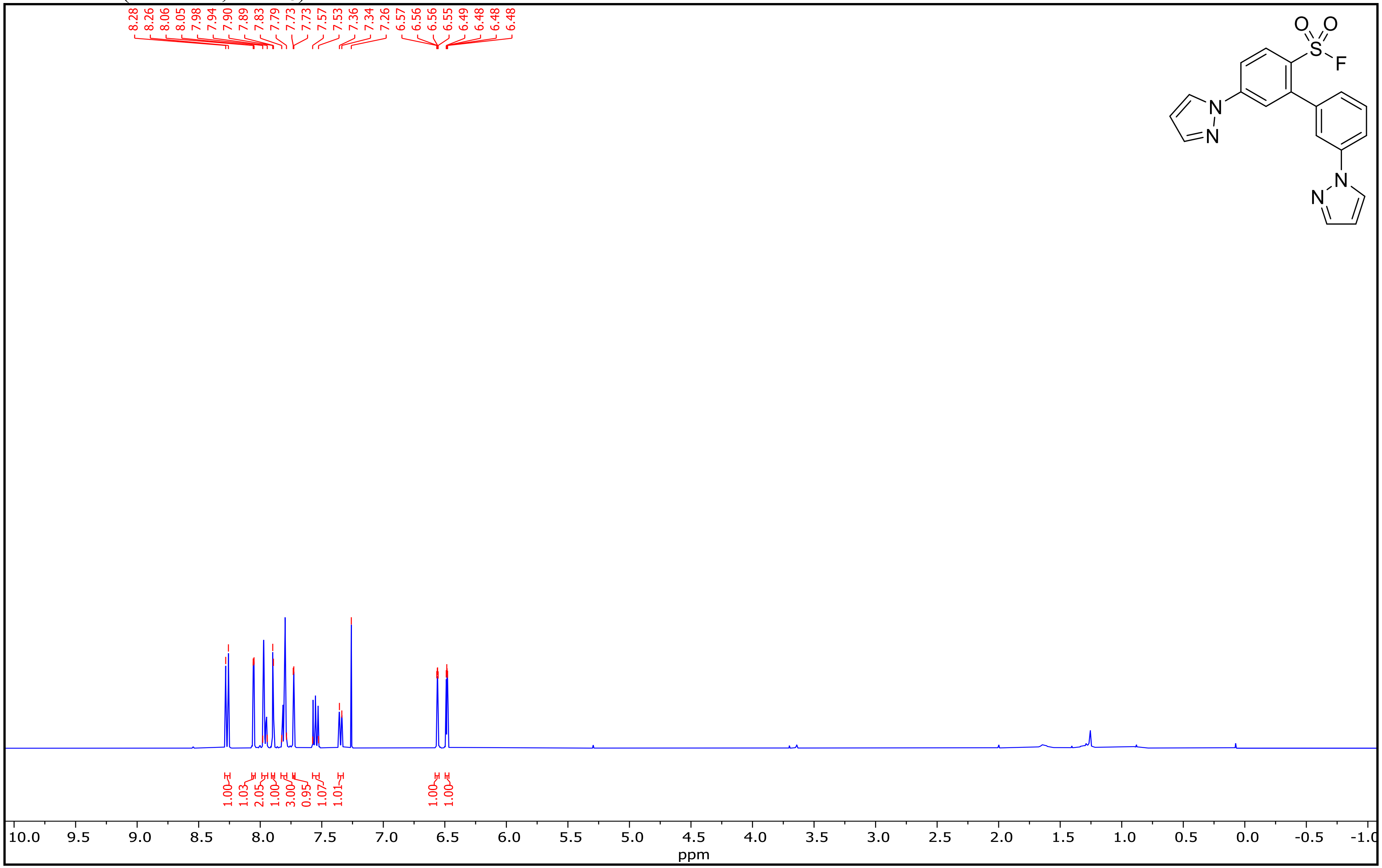

3i ^{13}C NMR (101 MHz, $CDCl_3$)

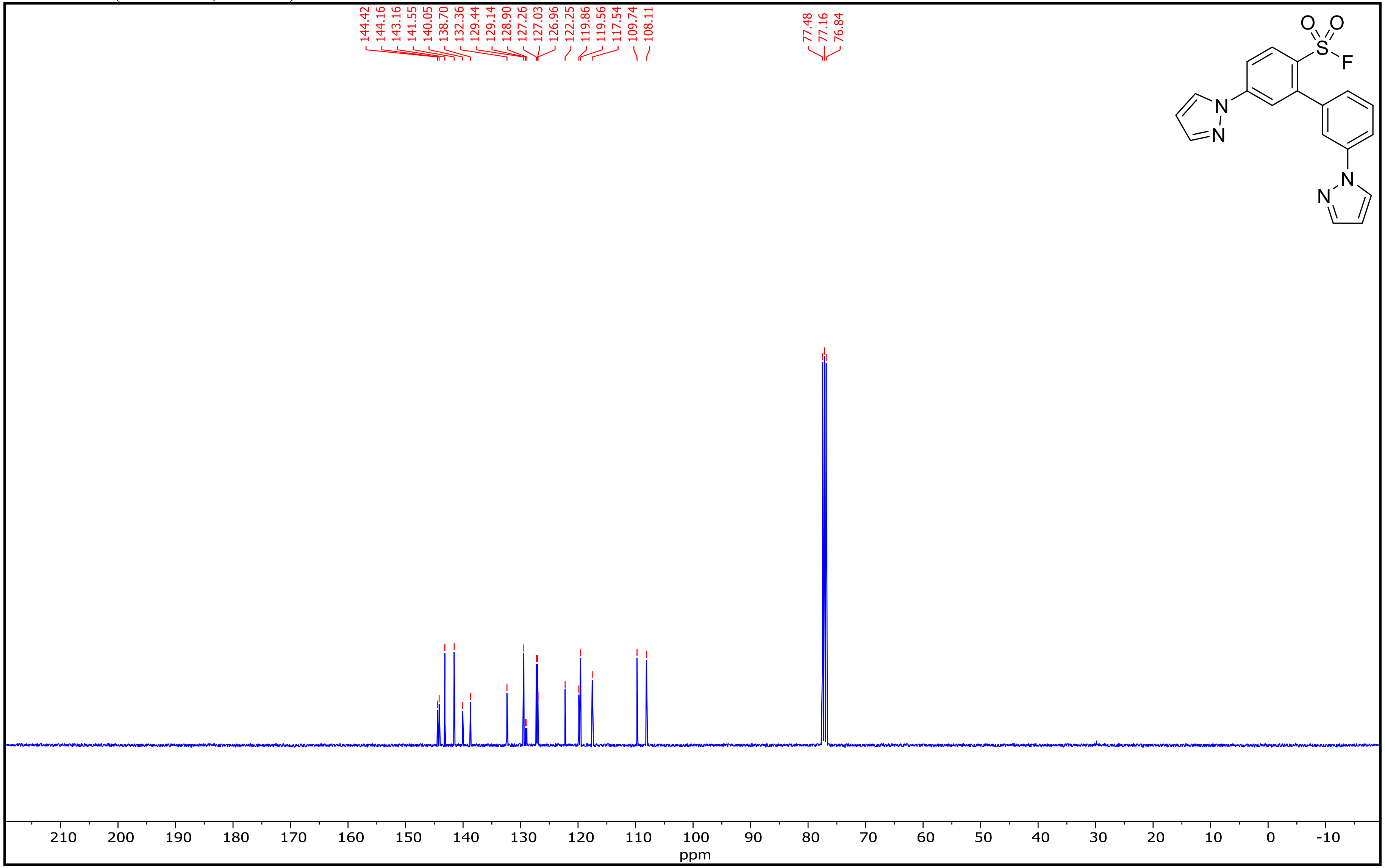

3i ^{19}F NMR (376 MHz, CDCl$_3$)

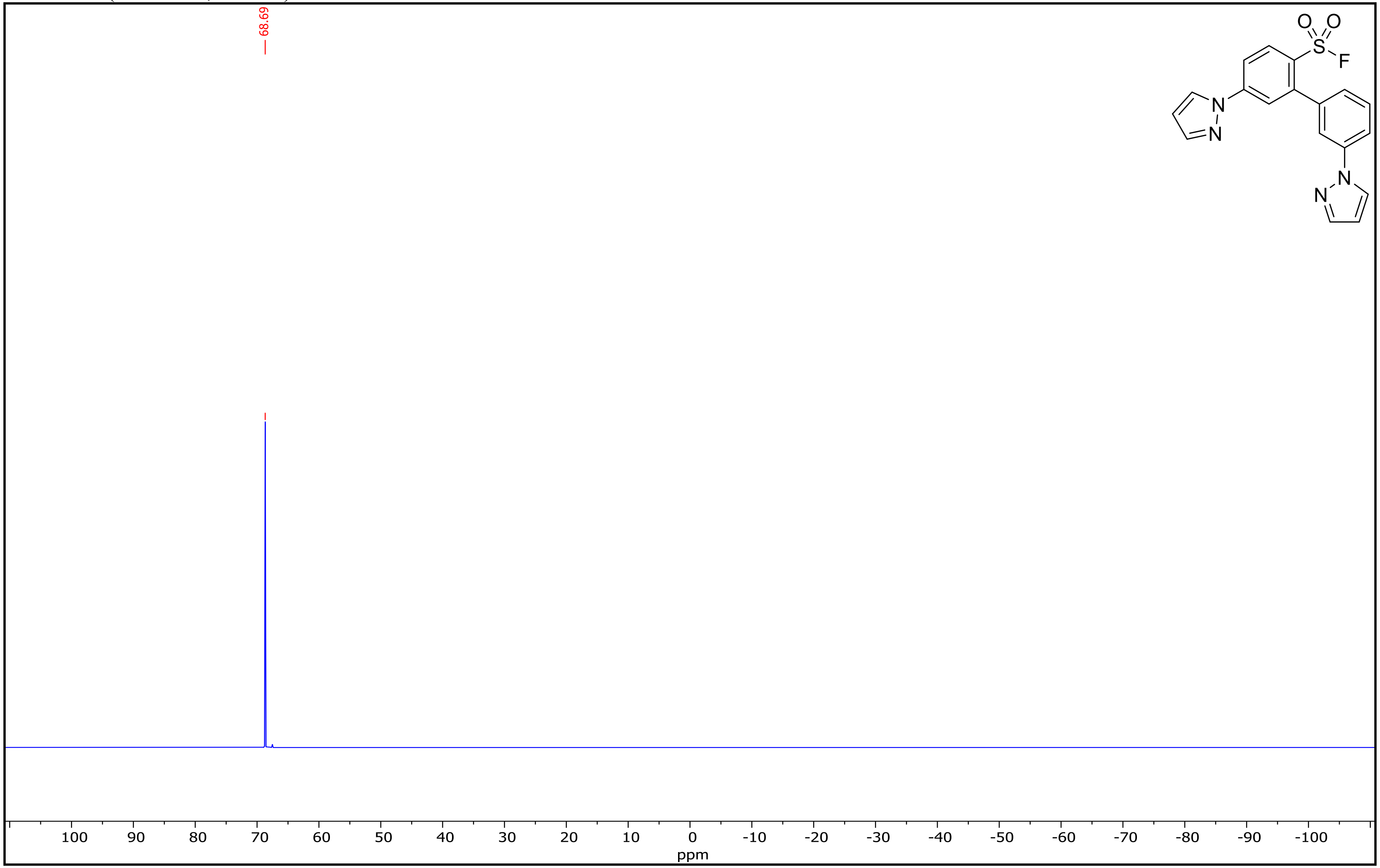

3j ^{1}H NMR (400 MHz, $CDCl_3$)

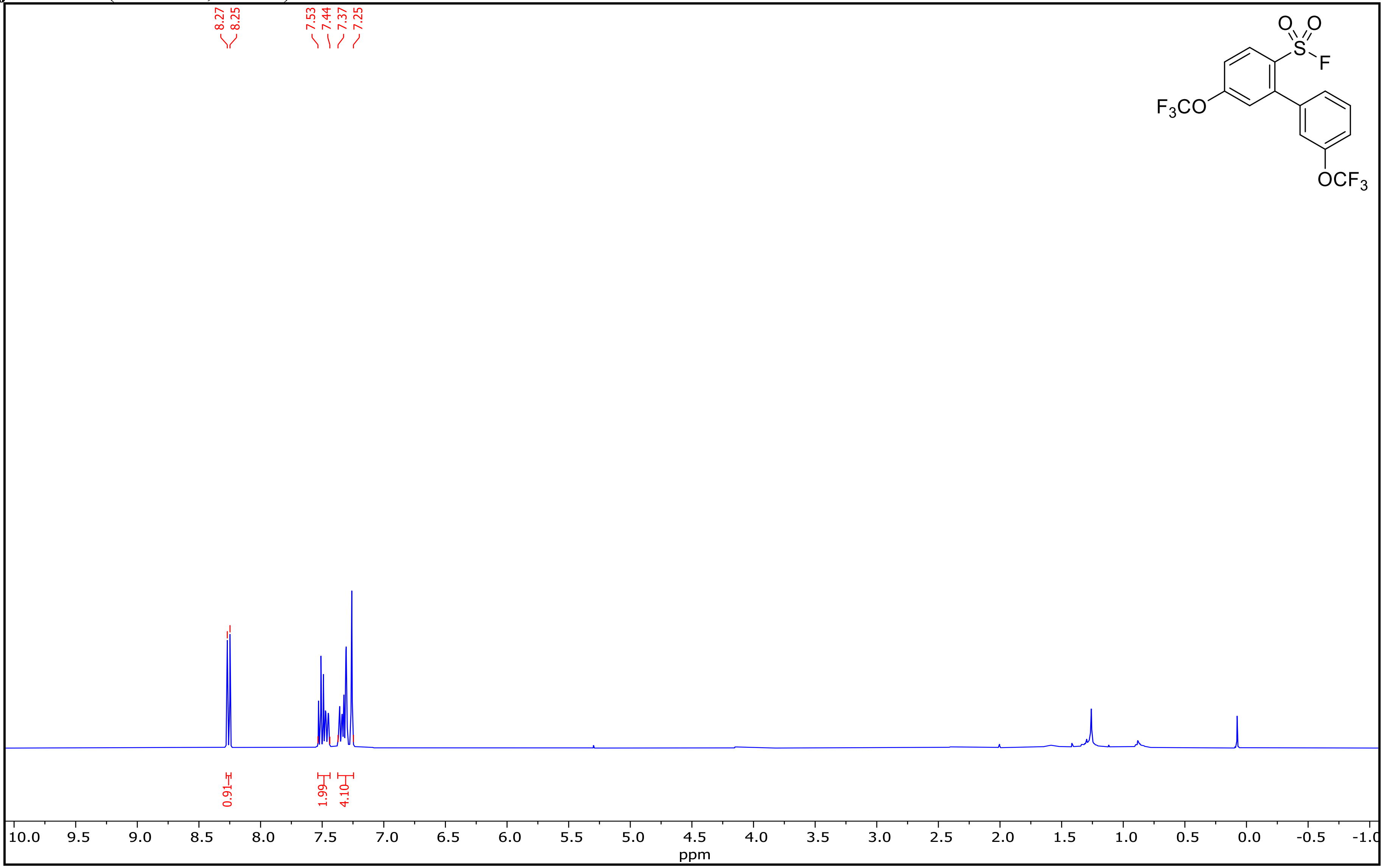

3j ^{13}C NMR (101 MHz, $CDCl_3$)

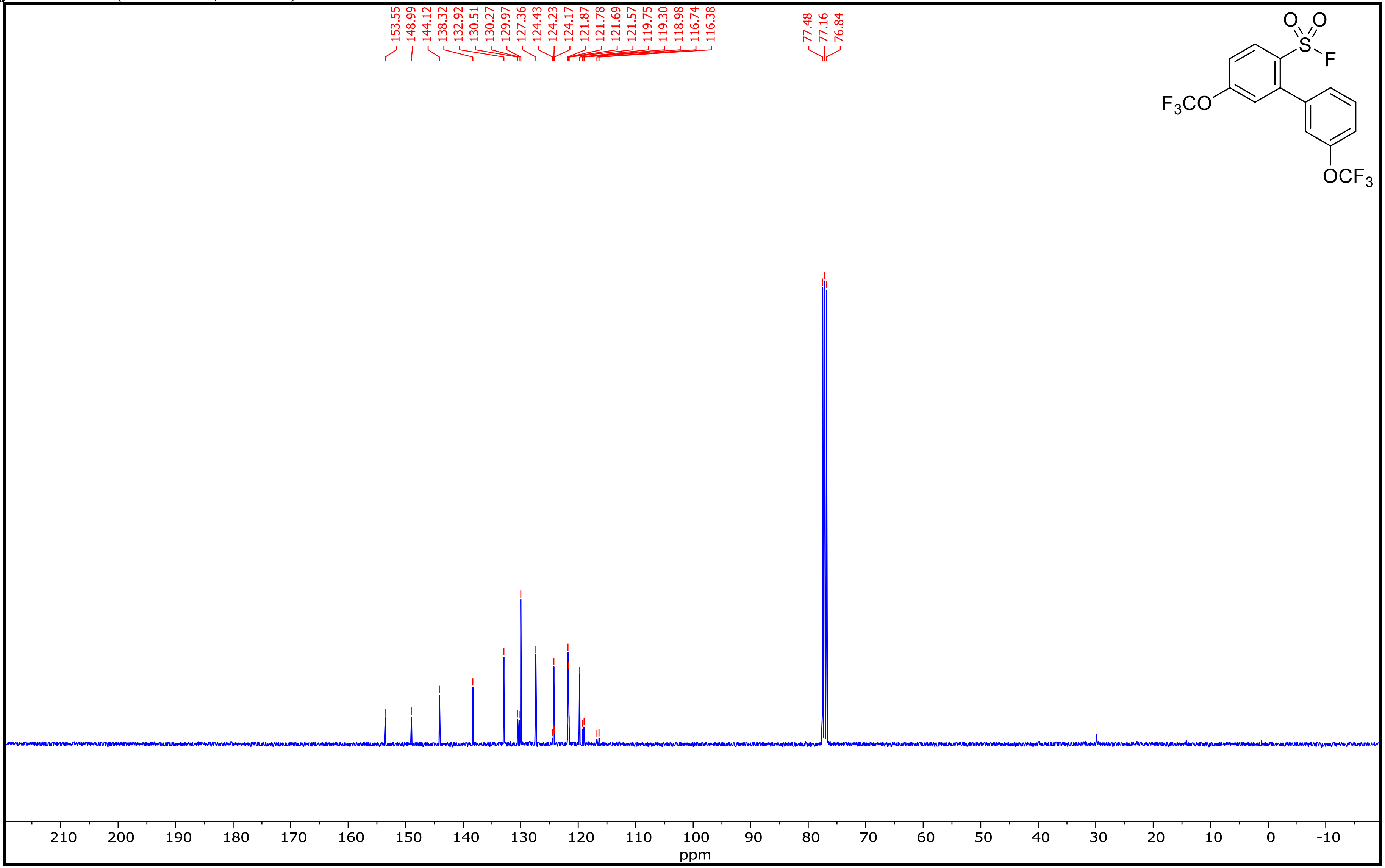

3j ^{19}F NMR (376 MHz, $CDCl_3$)

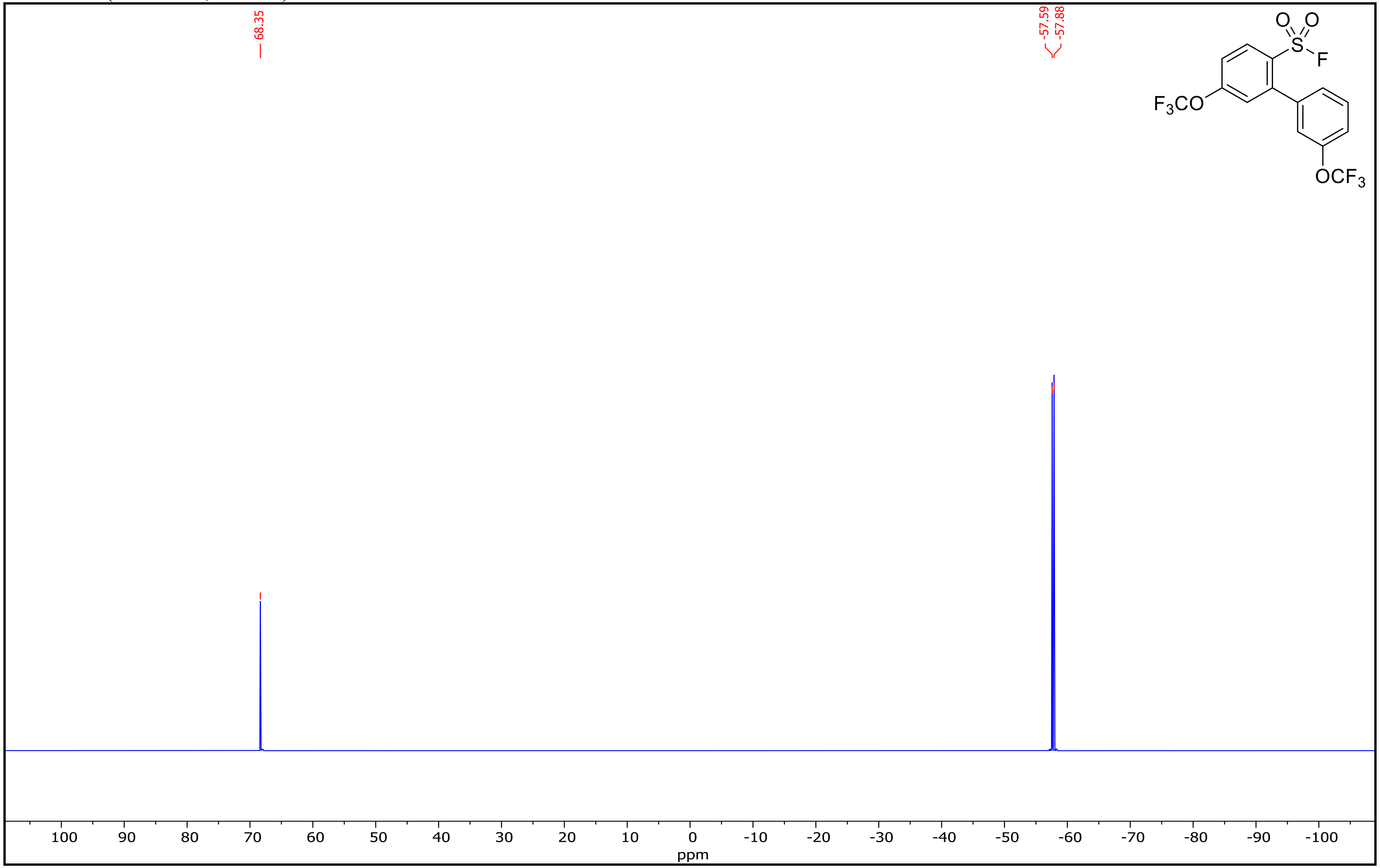

3k ^{1}H NMR (400 MHz, $CDCl_3$)

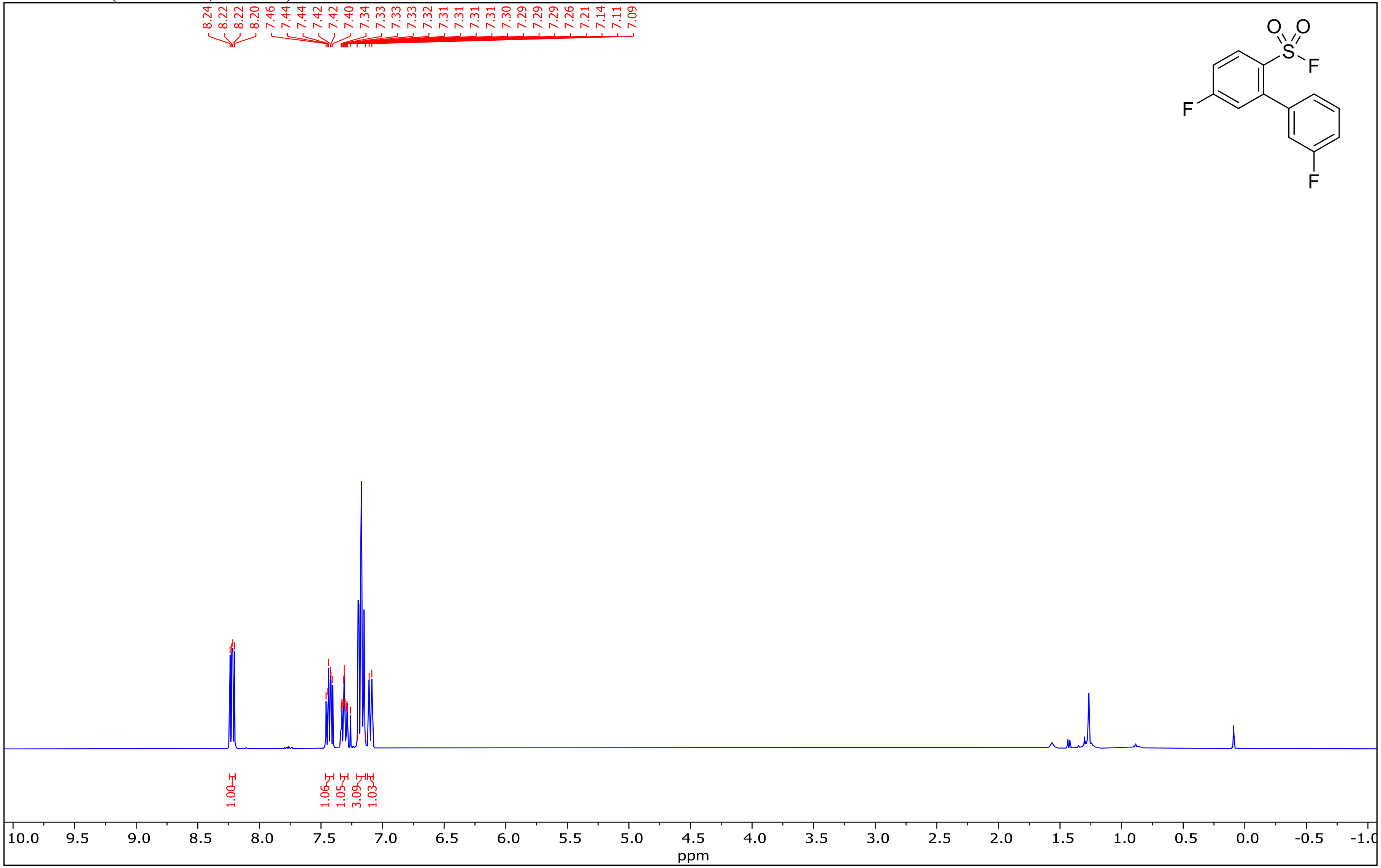

3k ^{13}C NMR (101 MHz, $CDCl_3$)

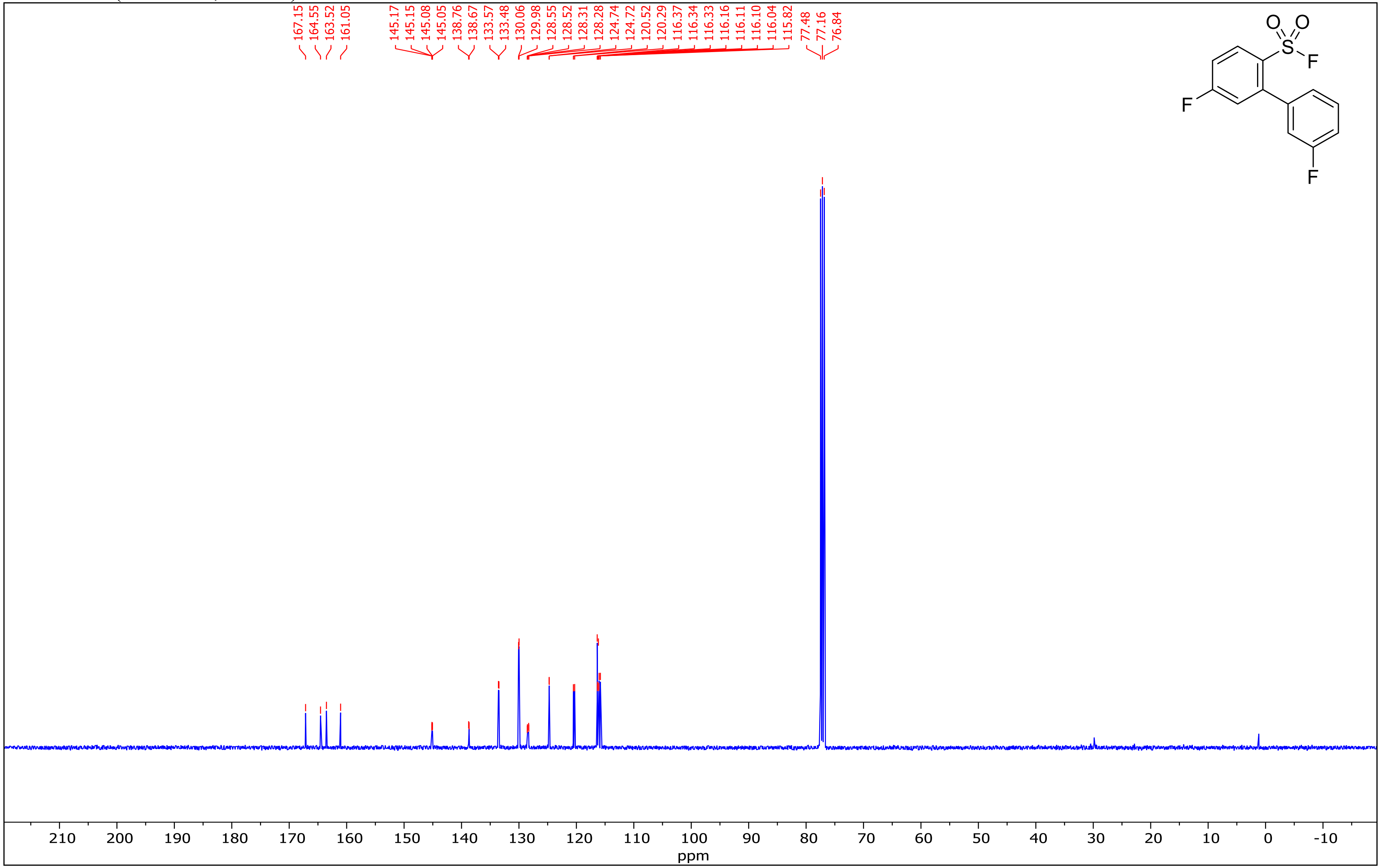

3k ^{19}F NMR (376 MHz, $CDCl_3$)

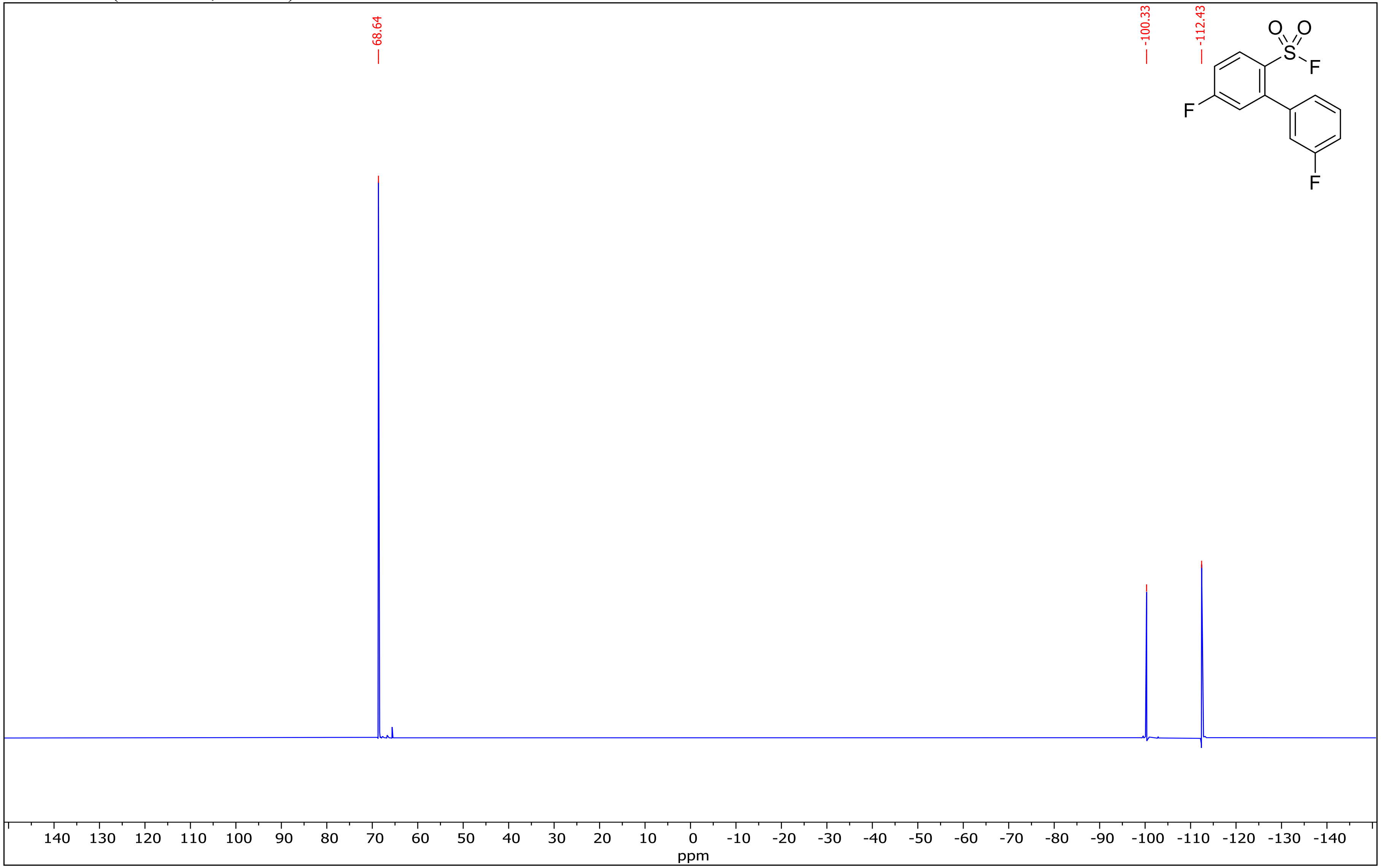

3m/3m' ^{1}H NMR (400 MHz, $CDCl_3$)

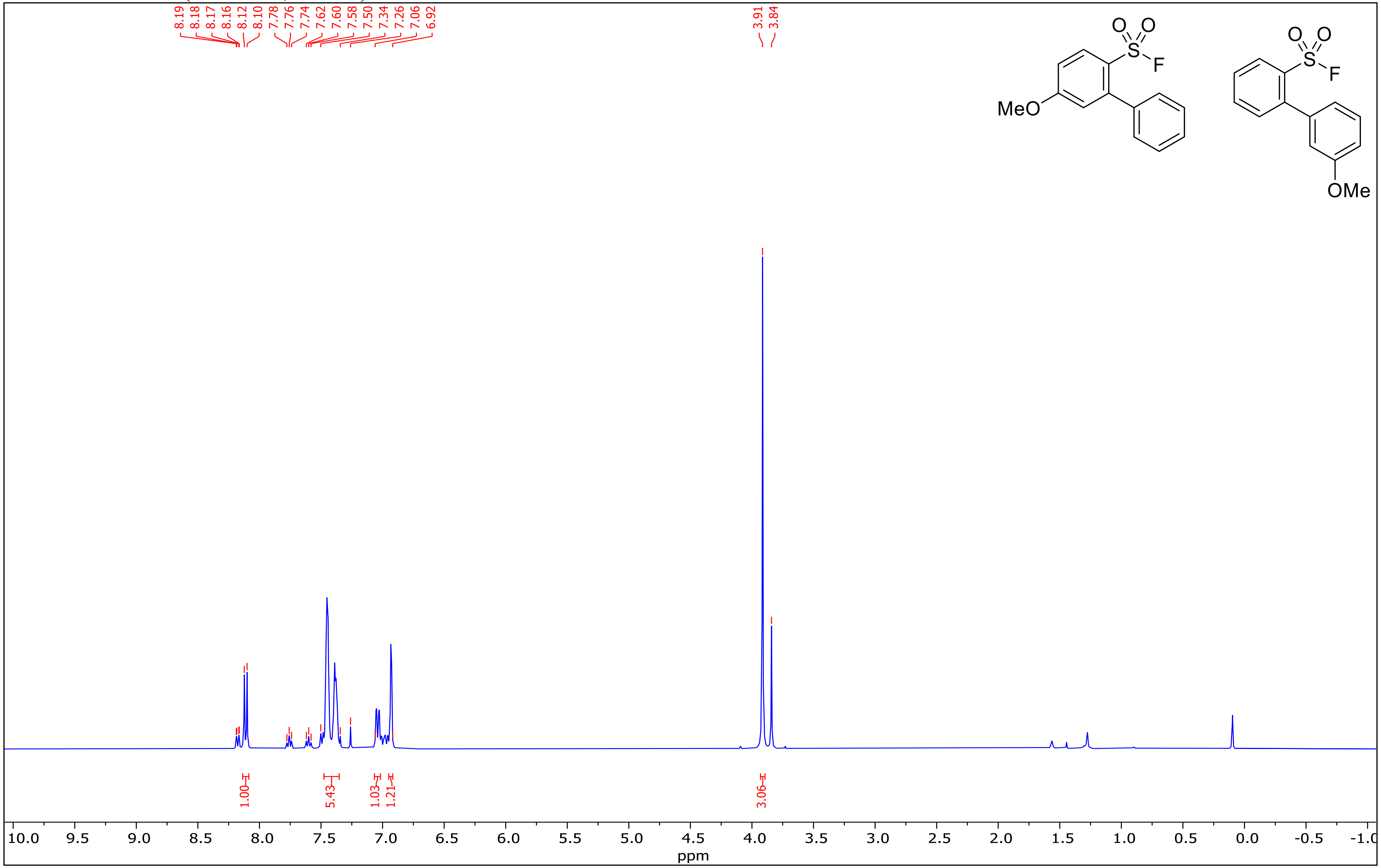

3m/3m’ ^{13}C NMR (101 MHz, $CDCl_3$)

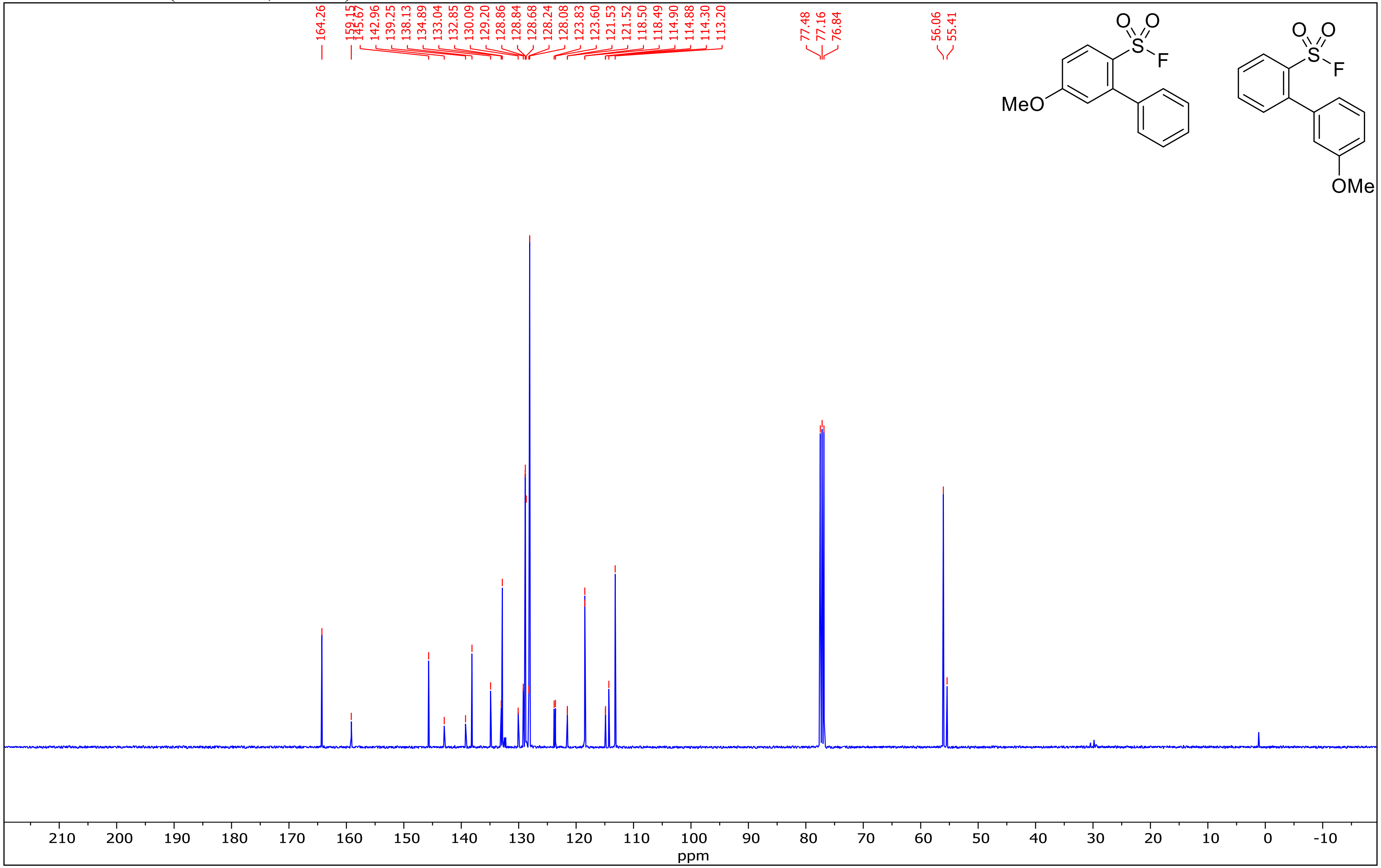

3m/3m' ^{19}F NMR (376 MHz, $CDCl_3$)

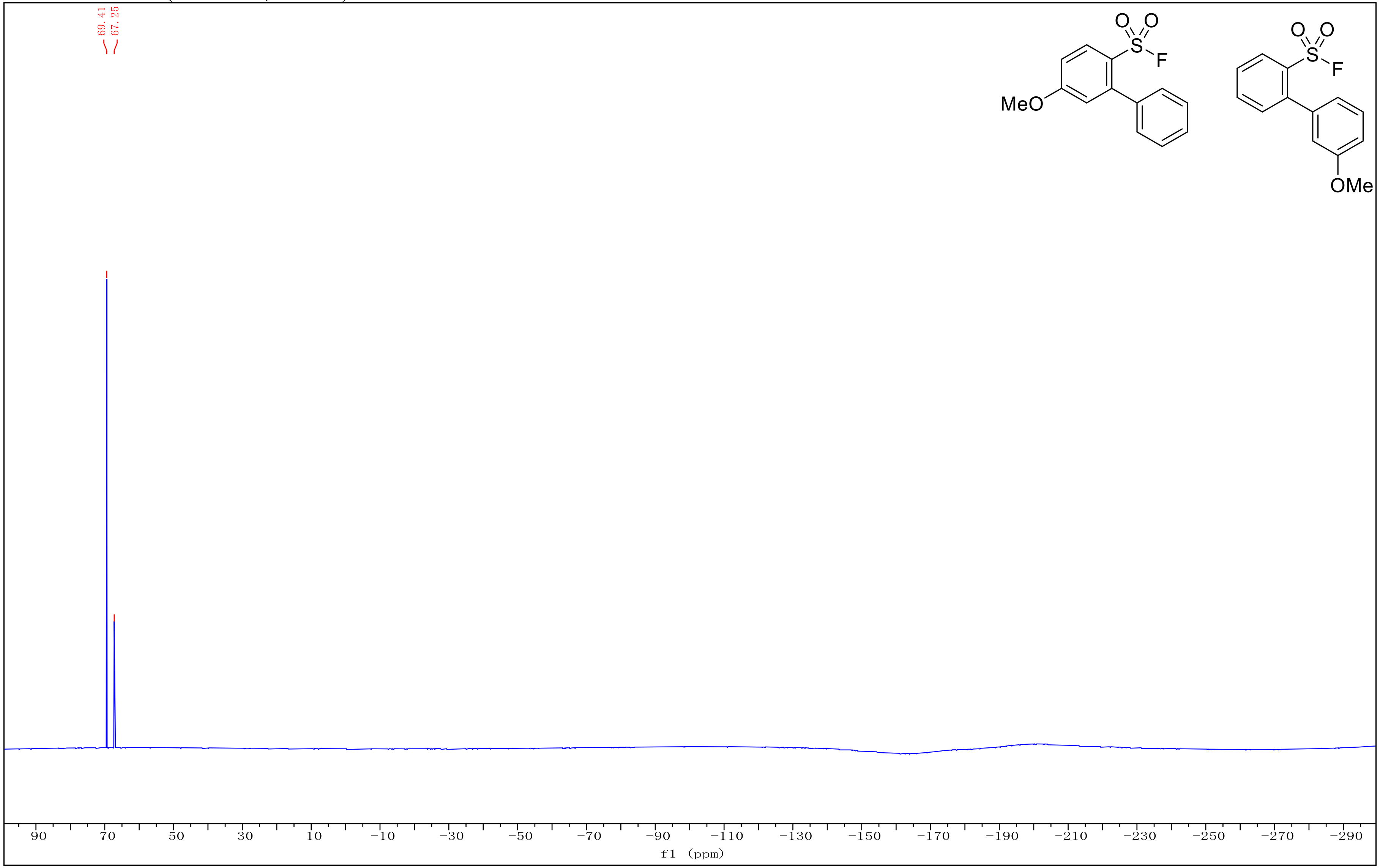

3n ^{1}H NMR (400 MHz, $CDCl_3$)

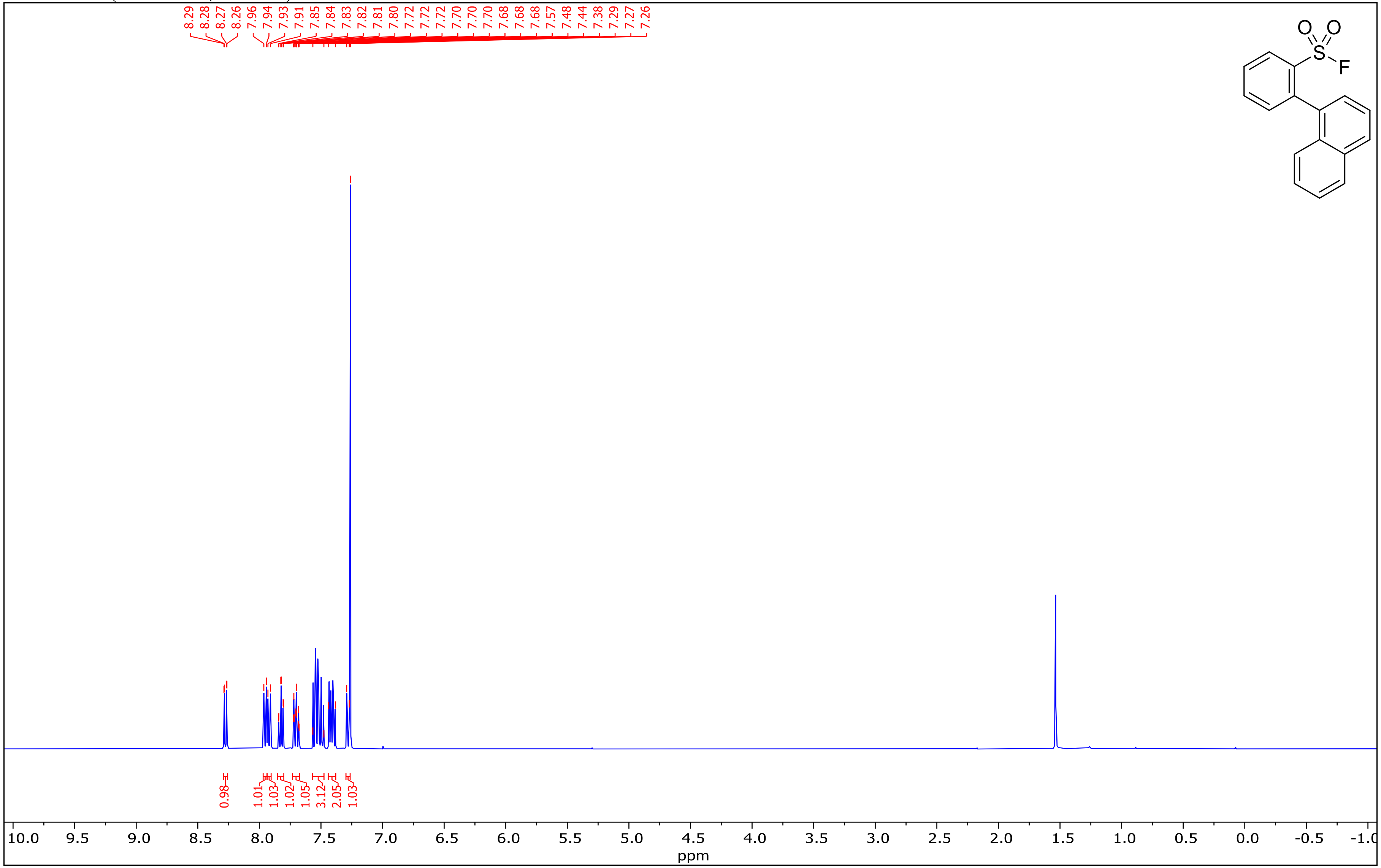

3n ^{13}C NMR (101 MHz, $CDCl_3$)

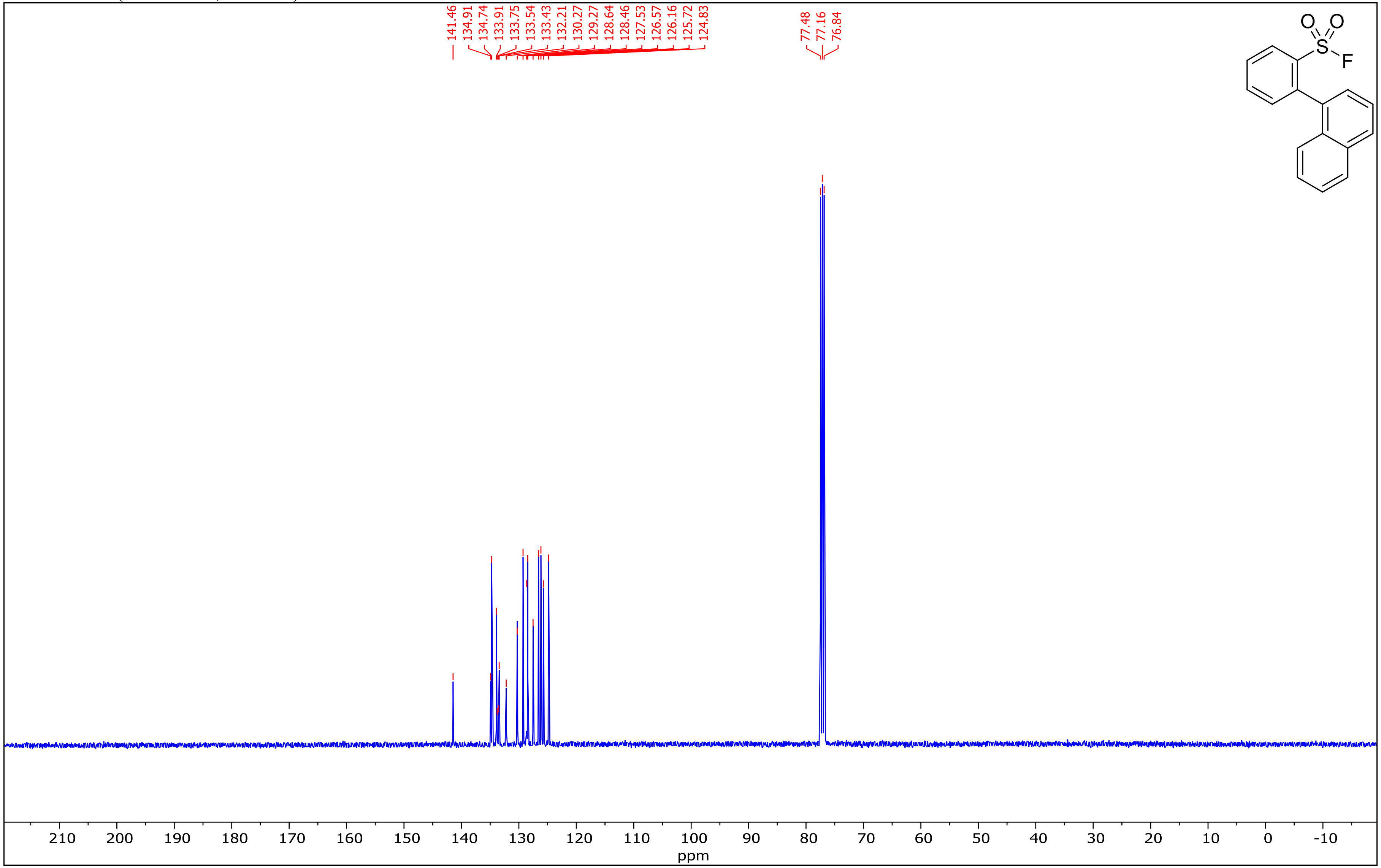

3n ^{19}F NMR (376 MHz, $CDCl_3$)

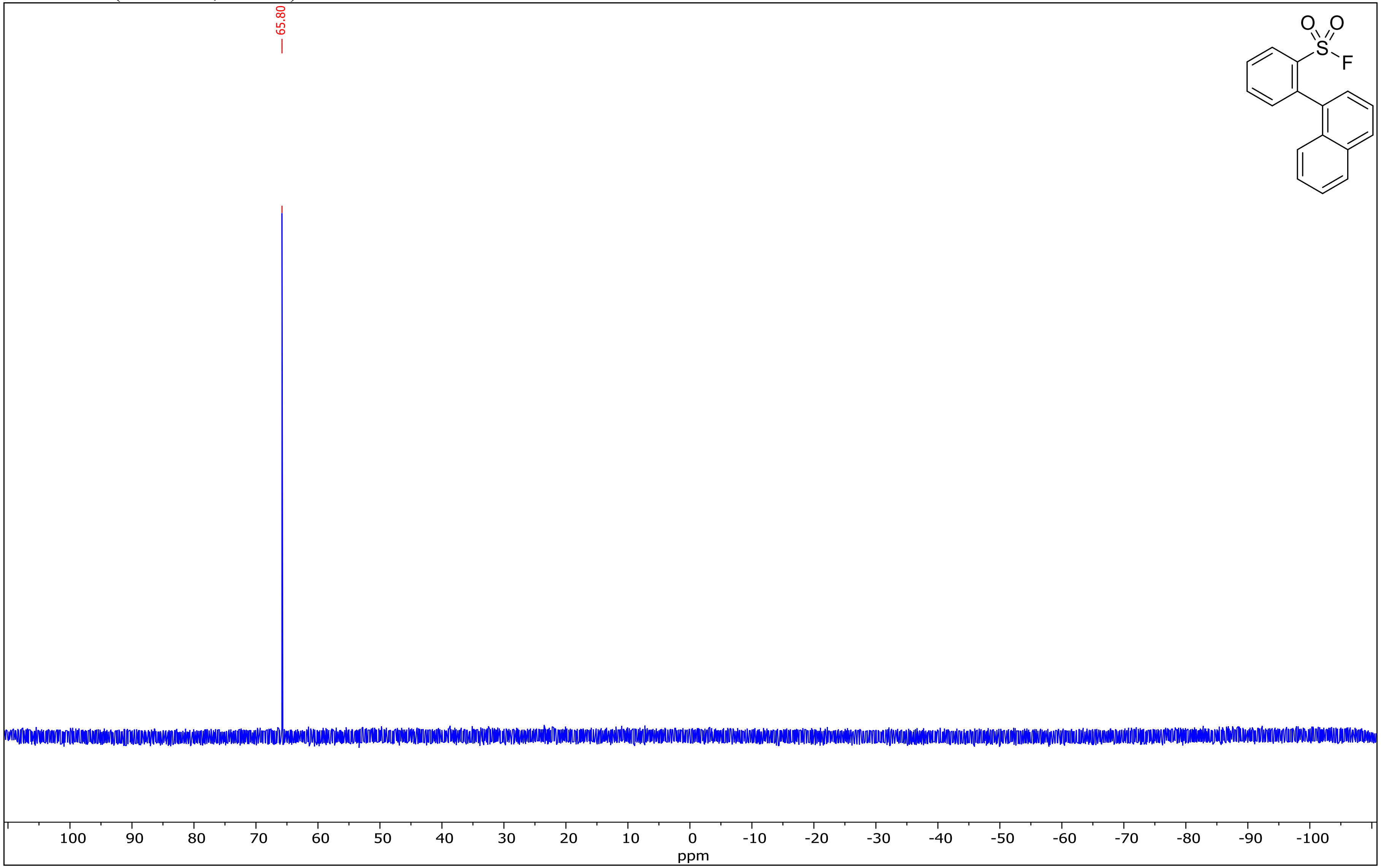

4a ^{1}H NMR (400 MHz, $CDCl_3$)

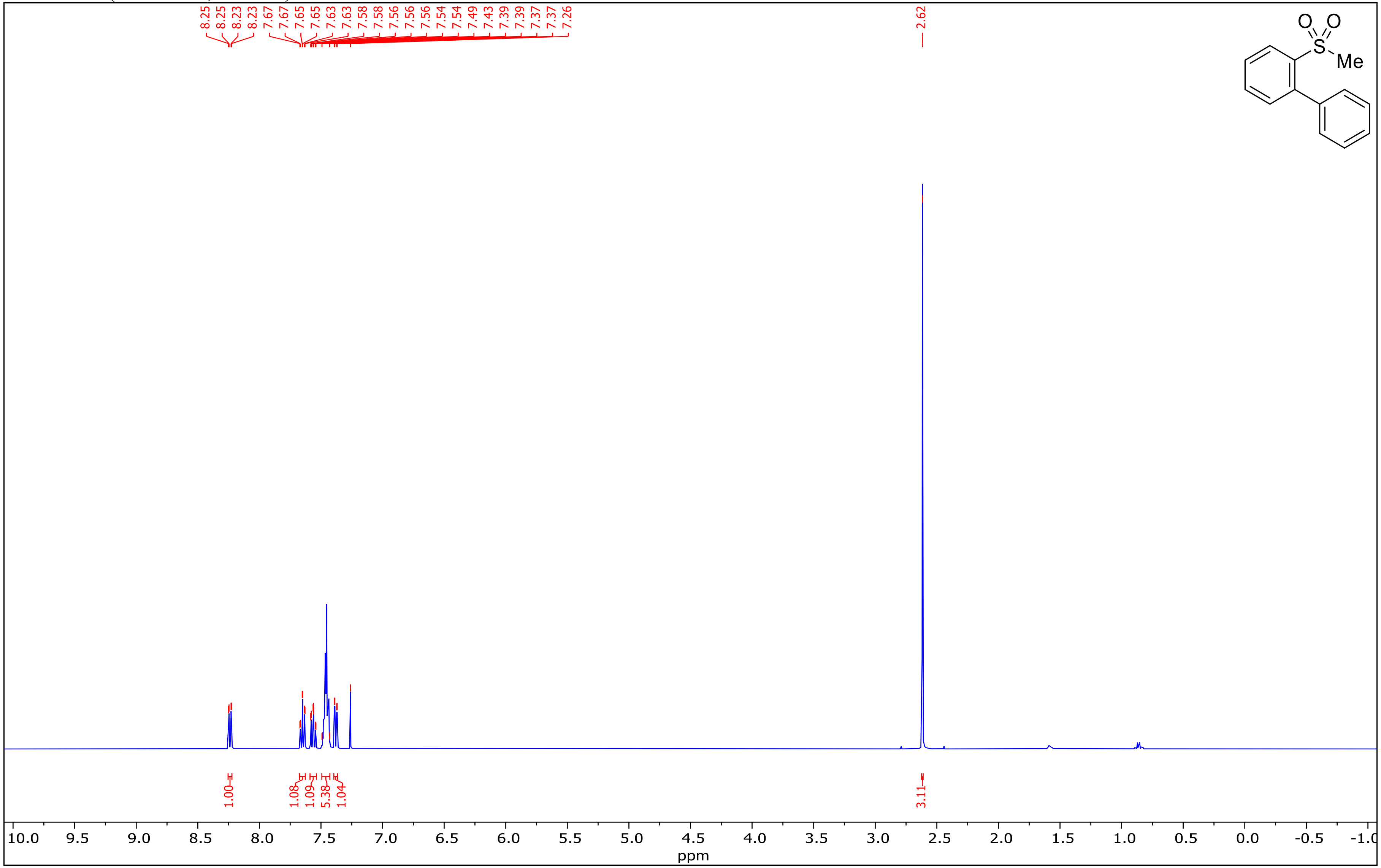

4a ^{13}C NMR (101 MHz, $CDCl_3$)

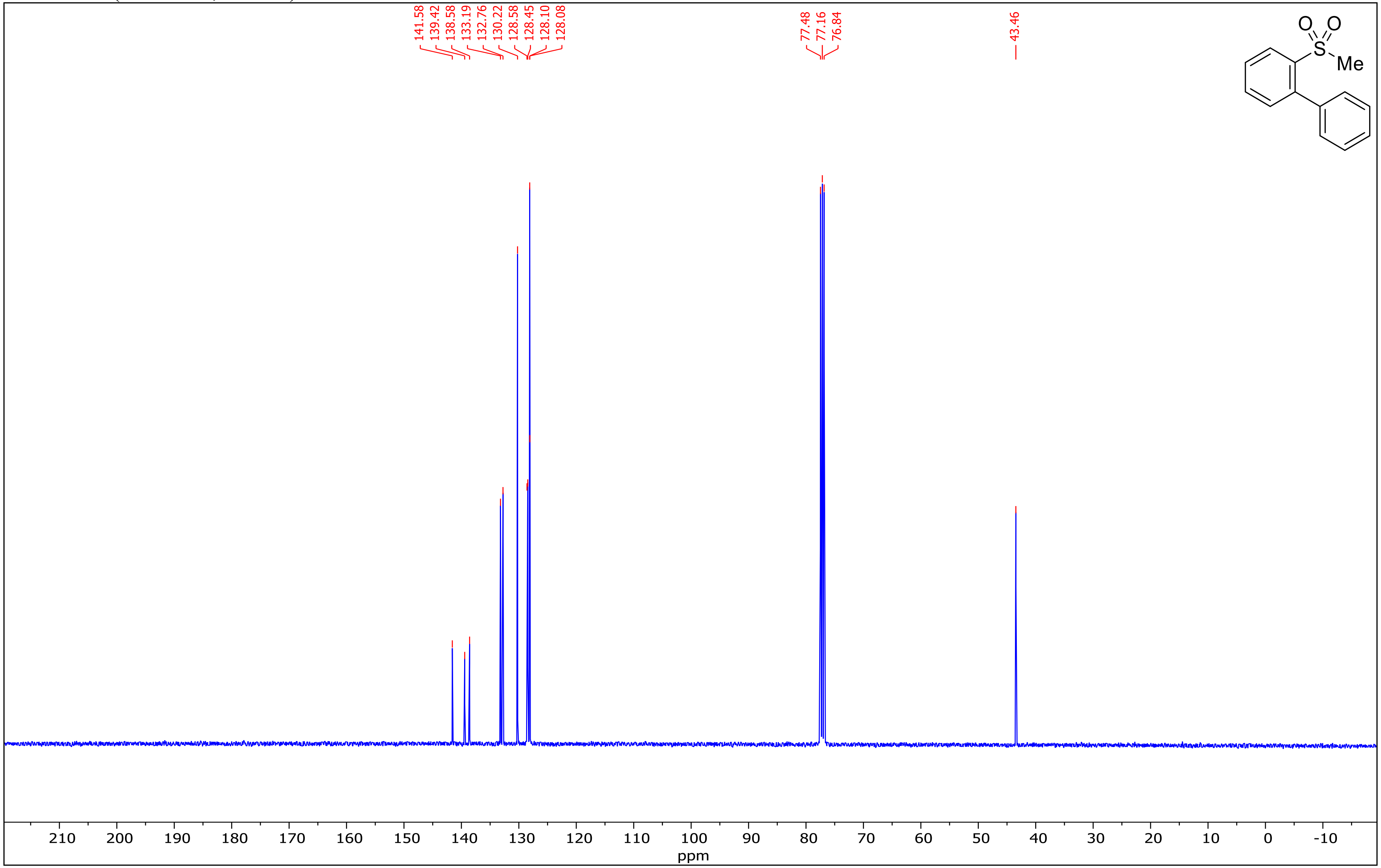

5a ^1H NMR (400 MHz, $CDCl_3$)

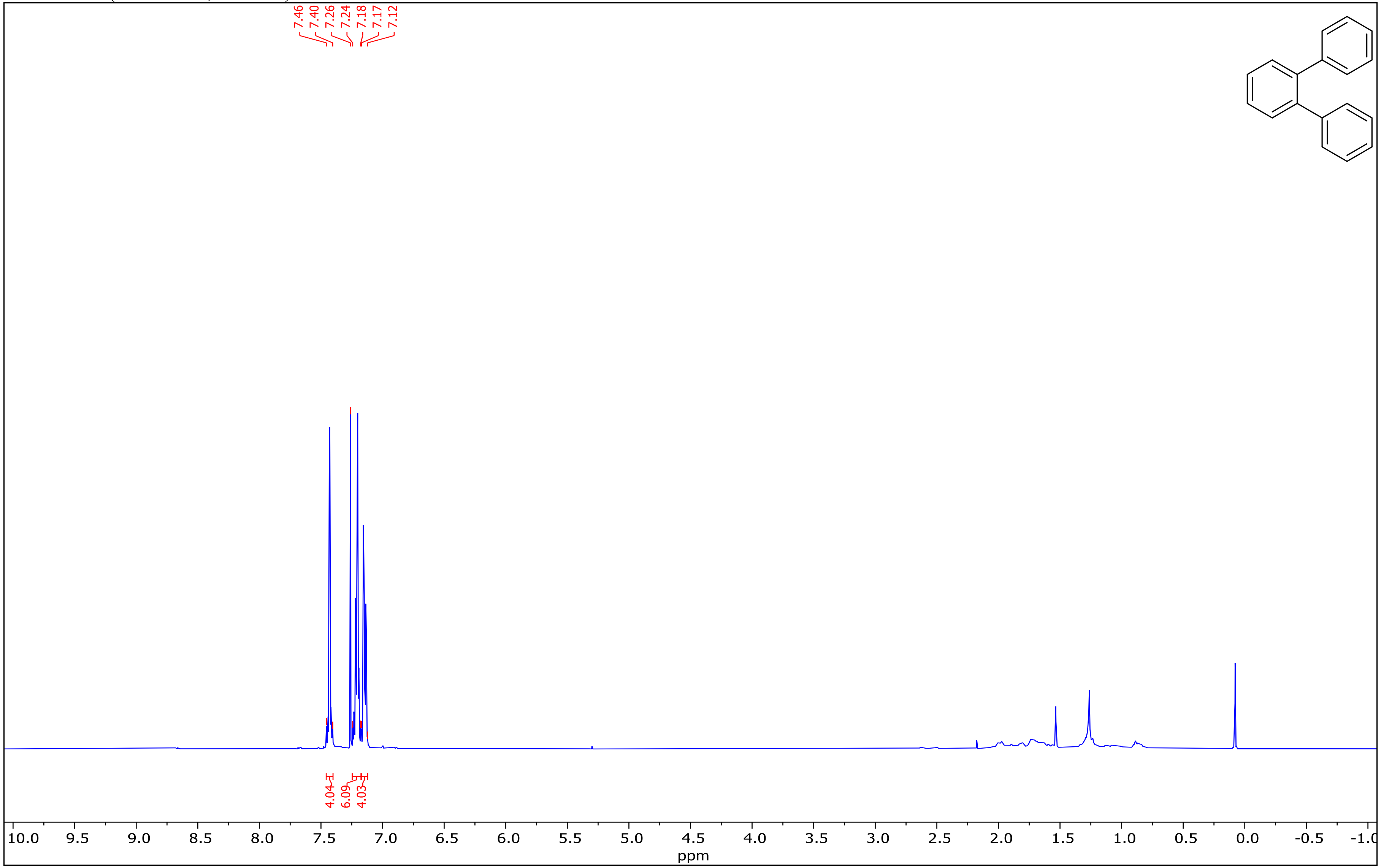

5a ^{13}C NMR (101 MHz, $CDCl_3$)

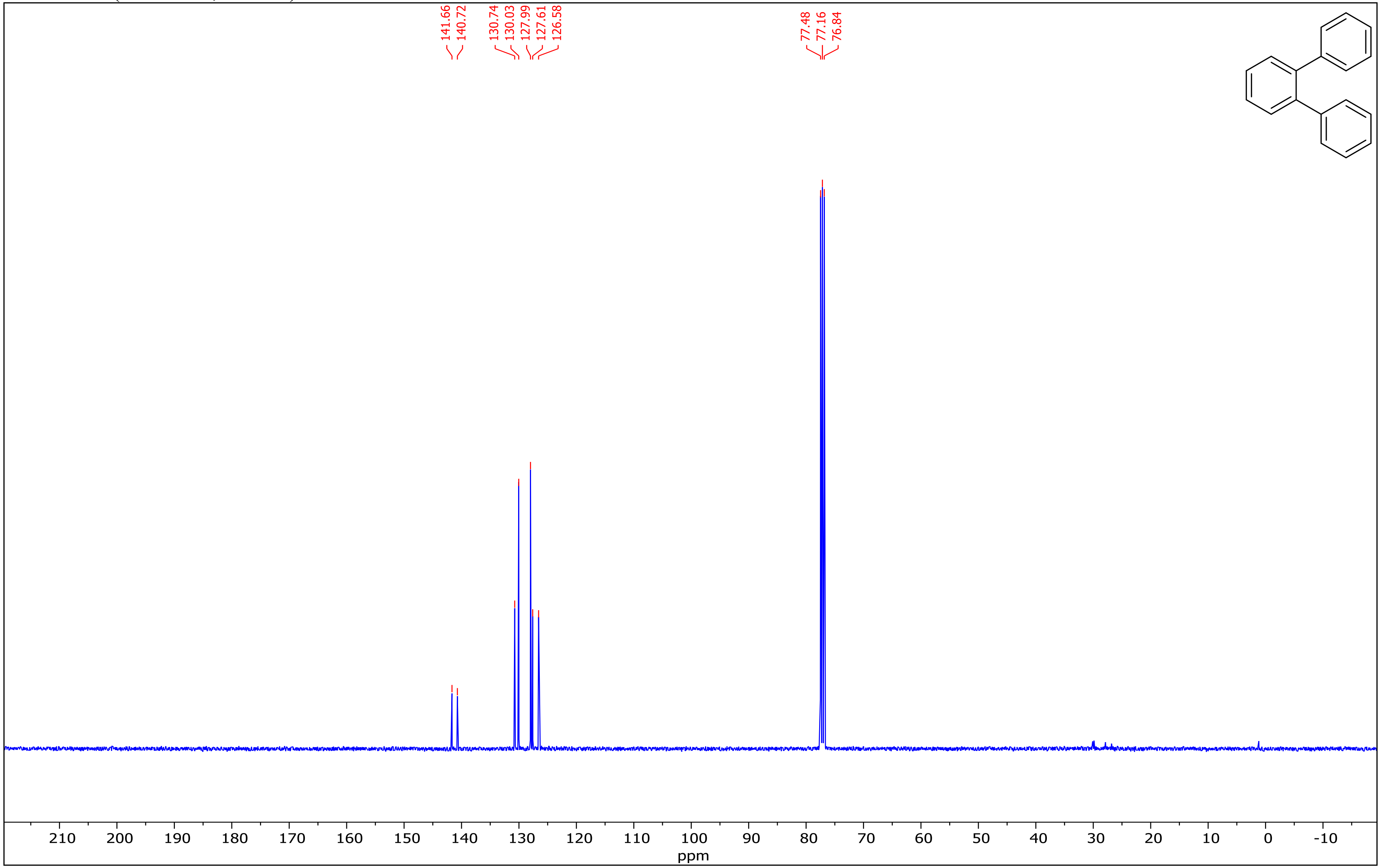

25) References

1) A. F. Burchat, J. M. Chong and N. Nielsen, *J. Organomet. Chem.*, 1997, **542**, 281–283.

2) H. Sirringhaus, R. H. Friend, C. Wang, J. Leuninger and K. Müllen, *J. Mater. Chem.*, 1999, **9**, 2095–2101.

3) X. Gao, C. Shu, C. Zhang, W. Ma, S.-B. Ren, F. Wang, Y. Chen, J. H. Zeng and J.-X. Jiang, *J. Mater. Chem. A*, 2020, **8**, 2404–2411.

4) K. Sato, M. Hyodo, M. Aoki, X.-Q. Zheng and R. Noyori, *Tetrahedron*, 2001, **57**, 2469–2476.

5) R. S. Nobuyasu, J. S. Ward, J. Gibson, B. A. Laidlaw, Z. Ren, P. Data, A. S. Batsanov, T. J. Penfold, M. R. Bryce and F. B. Dias, *J. Mater. Chem. C*, 2019, **7**, 6672–6684.

6) M. Bhanuchandra, K. Murakami, D. Vasu, H. Yorimitsu and A. Osuka, *Angew. Chem. Int. Ed.*, 2015, **54**, 10234–10238.

7) M. Yadav, R. Singh Jat, S. Kumari, P. Vijaya Babu, P. Roy and M. Bhanuchandra, *Tetrahedron Lett.*, 2023, **119**, 154430.

8) J. S. Ward, R. S. Nobuyasu, A. S. Batsanov, P. Data, A. P. Monkman, F. B. Dias and M. R. Bryce, *Chem. Commun.*, 2016, **52**, 2612–2615.

9) M. Bhanuchandra, H. Yorimitsu and A. Osuka, *Org. Lett.*, 2016, **18**, 384–387.

10) A. Sacristán-Martín, D. Miguel, A. Diez-Varga, H. Barbero and C. M. Álvarez, *J. Org. Chem.*, 2022, **87**, 16691–16706.

11) A. Liang, M. Luo, Y. Liu, H. Wang, Z. Wang, X. Zheng, T. Cao, D. Liu, Y. Zhang and F. Huang, *Dyes Pigm.*, 2018, **159**, 637–645.

12) M. Wang, S. Chen and X. Jiang, *Org. Lett.*, 2017, **19**, 4916–4919.

13) F. Ruighi, E. Fabiano, L. Franco, A. Agostini, S. Zatta, G. A. Corrente, A. Beneduci, A. Cardone, G. Accorsi and A. L. Capodilupo, *Dyes Pigm.*, 2023, **219**, 111582.

14) L. Hu, J. Liang, C. Liu, Y. Cheng, T. Guo, B. Xiao, L. Ying and R. Yang, *Org. Electron.*, 2023, **117**, 106780.

15) M. Feofanov, V. Akhmetov, R. Takayama and K. Y. Amsharov, *J. Org. Chem.*, 2021, **86**, 14759–14766.

16) Q. Huang, S. Fu, S. Ke, H. Xiao, X. Zhang and S. Lin, *Eur. J. Org. Chem.*, 2015, **2015**, 6602–6605.

17) S. Kawano, T. Hamazaki, A. Suzuki, K. Kurahashi and K. Tanaka, *Chem. Eur. J.*, 2016, **22**, 15674–15683.

18) C. W. Cheung and S. L. Buchwald, *Org. Lett.*, 2013, **15**, 3998–4001.

19) S. Panda, R. S. Jat, A. Fayaz, J. Saha, R. Thirumoorthi, T. K. Roy and M. Bhanuchandra, *New J. Chem.*, 2020, **44**, 8944–8951.

20) T. Umemoto, B. Zhang, T. Zhu, X. Zhou, P. Zhang, S. Hu and Y. Li, *J. Org. Chem.*, 2017, **82**, 7708–7719.

21) E. Neofotistou, C. D. Malliakas and P. N. Trikalitis, *Chem. Eur. J.*, 2009, **15**, 4523–4527.

22) G. Wang, K. Leus, S. Couck, P. Tack, H. Depauw, Y.-Y. Liu, L. Vincze, J. F. M. Denayer and P. Van Der Voort, *Dalton Trans.*, 2016, **45**, 9485–9491.

23) T. S. Lou, Y. Kawamata, T. Ewing, G. A. Correa-Otero, M. R. Collins and P. S. Baran, *Angew. Chem. Int. Ed.*, 2022, **61**, e202208080.

24) S.-R. Guo, Y.-Q.Yuan, *J. Chem. Res.*, 2009, **12** 745–749.

25) Z. Li, D. Rong, L. Yuan, Z. Zhao, F. Dai, L. Chen and Y. Xie, *Org. Biomol. Chem.*, 2024, **22**, 4559–4567.

26) V. V. Pavlishchuk and A. W. Addison, *Inorg. Chim. Acta*, 2000, **298**, 97–102.

27) (a) A. J. N. Price, A. J. Capel, R. J. Lee, P. Pradel and S. D. R. Christie, *J. Flow. Chem.*, 2021, **11**, 37–51. (b) A. Nightingale, K. Rankin and R. Leeder, 2024, preprint, DOI: 10.31224/4104.

28) (a) A. D. Becke, *J. Chem. Phys.*, 1993, **98**, 5648–5652. (b) C. Lee, W. Yang and R. G. Parr, *Phys. Rev. B*, 1988, **37**, 785–789.

29) Gaussian 16, Revision C.02, M. J. Frisch, G. W. Trucks, H. B. Schlegel, G. E. Scuseria, M. A. Robb, J. R. Cheeseman, G. Scalmani, V. Barone, G. A. Petersson, H. Nakatsuji, X. Li, M. Caricato, A. V. Marenich, J. Bloino, B. G. Janesko, R. Gomperts, B. Mennucci, H. P. Hratchian, J. V. Ortiz, A. F. Izmaylov, J. L. Sonnenberg, D. Williams-Young, F. Ding, F. Lipparini, F. Egidi, J. Goings, B. Peng, A. Petrone, T. Henderson, D. Ranasinghe, V. G. Zakrzewski, J. Gao, N. Rega, G. Zheng, W. Liang, M. Hada, M. Ehara, K. Toyota, R. Fukuda, J. Hasegawa, M. Ishida, T. Nakajima, Y. Honda, O. Kitao, H. Nakai, T. Vreven, K. Throssell, J. A. Montgomery, Jr., J. E. Peralta, F. Ogliaro, M. J. Bearpark, J. J. Heyd, E. N. Brothers, K. N. Kudin, V. N. Staroverov, T. A. Keith, R. Kobayashi, J. Normand, K. Raghavachari, A. P. Rendell, J. C. Burant, S. S. Iyengar, J. Tomasi, M. Cossi, J. M. Millam, M. Klene, C. Adamo, R. Cammi, J. W. Ochterski, R. L. Martin, K. Morokuma, O. Farkas, J. B. Foresman, and D. J. Fox, Gaussian, Inc., Wallingford CT, 2016.

30) S. Grimme, J. Antony, S. Ehrlich and H. Krieg, *J. Chem. Phys.*, 2010, **132**, 154104.

31) A. V. Marenich, C. J. Cramer and D. G. Truhlar, *J. Phys. Chem. B*, 2009, **113**, 6378–6396.

32) Y. Zhao and D. G. Truhlar, *Theor. Chem. Acc.*, 2008, **120**, 215–241.
